# A fast-growing basal troodontid (Dinosauria: Theropoda) from the latest Cretaceous of Europe

**DOI:** 10.1038/s41598-021-83745-5

**Published:** 2021-03-01

**Authors:** Albert G. Sellés, Bernat Vila, Stephen L. Brusatte, Philip J. Currie, Àngel Galobart

**Affiliations:** 1 Institut Català de Paleontologia Miquel Crusafon, Edifici Z, C/ de les columnes s/n, Campus de la Universitat Autònoma de Barcelona, Cerdanyola del Vallès, Barcelona, Spain; 2 Museu de la Conca Dellà, 25650 Lleida, Isona Spain; 3 School of GeoSciences, University of Edinburgh, Edinburgh, EH9 3FE UK; 4 University of Alberta, CW-405 Biological Sciences Building, Edmonton, AB T6G 2E9 Canada

**Keywords:** Evolution, Palaeontology, Phylogenetics, Taxonomy

## Abstract

A characteristic fauna of dinosaurs and other vertebrates inhabited the end-Cretaceous European archipelago, some of which were dwarves or had other unusual features likely related to their insular habitats. Little is known, however, about the contemporary theropod dinosaurs, as they are represented mostly by teeth or other fragmentary fossils. A new isolated theropod metatarsal II, from the latest Maastrichtian of Spain (within 200,000 years of the mass extinction) may represent a jinfengopterygine troodontid, the first reported from Europe. Comparisons with other theropods and phylogenetic analyses reveal an autapomorphic foramen that distinguishes it from all other troodontids, supporting its identification as a new genus and species, *Tamarro insperatus*. Bone histology shows that it was an actively growing subadult when it died but may have had a growth pattern in which it grew rapidly in early ontogeny and attained a subadult size quickly. We hypothesize that it could have migrated from Asia to reach the Ibero-Armorican island no later than Cenomanian or during the Maastrichtian dispersal events.

## Introduction

During the latest Cretaceous (ca. 77–66 million years ago) in the run-up to the end-Cretaceous mass extinction, Europe was a series of islands populated by diverse and distinctive communities of dinosaurs and other vertebrates. Many of these animals exhibited peculiar features that may have been generated by lack of space and resources in their insular habitats. These include dwarf sauropod and ornithischian dinosaurs with slow growth rates^1–4^, anatomically bizarre small theropods^5,6^, and mammals with reduced brain sizes^7^. Furthermore, these faunas were notably different from those on the closest mainlands (Asia and North America). The characteristic European ‘island fauna’ consisted of titanosaurian sauropods, rhabdodontid iguanodontians, nodosaurid ankylosaurians, basal hadrosauroids, lambeosaurine hadrosauroids, and some abelisauroid and maniraptoran theropods^8,9^. As more fossils of these animals are discovered, they are becoming increasingly important for understanding how dinosaurs and other vertebrates changed before the end-Cretaceous asteroid impact, and for testing hypotheses of rapid^10^ vs. gradual extinction^8,11^.

There are still large gaps in our understanding of these island faunas, in terms of their composition and their evolution over time. Relatively little is known about the theropod dinosaurs, which were the top predators on the mainland during this time, and probably also on the islands (although they may have shared this role with gigantic pterosaurs^12^). Theropods also diversified into omnivorous and herbivorous species^13^. Based on what is currently known, the non-avian theropod faunas were dominated by small-sized (< 50 kg) dromaeosaurids, whose fossils have been found in latest Cretaceous (Campanian and Maastrichtian) formations of the so-called Ibero-Armorican region (including the current areas of Portugal, Spain, and southern France), and in Transylvania (Romania)^5,8,9,14,15^. Frustratingly, they are nearly exclusively represented by isolated teeth^14^ and eggshell fragments^15–17^. Skeletal remains are extremely scarce^18–21^. Some exceptions include the partial skeleton of *Balaur bondoc*^5,6^ and the more fragmentary specimens of *Pyroraptor olympius*^22,23^ and *Variraptor mechinorum*^20–23^.

Knowing more about the island-dwelling theropods would provide considerable insight into the paleoecology and paleobiogeography of these insular ecosystems^8^. During the terminal Cretaceous, several paleogeographic, climatic, and biotic changes occurred worldwide^24–28^. In the European archipelago, there was a turnover amongst the dinosaur faunas in the ‘middle’ Maastrichtian^9^. Perhaps related to some of these changes, nodosaurids disappeared and more primitive rhabdodontids gave way to larger and more derived hadrosauroids^9,29^. It is unclear, however, whether theropods were affected by turnover. It is also uncertain where the theropods of the Ibero-Armorican land mass ultimately came from, and whether they were island dwarves or otherwise affected by their island habitat. Any new theropod fossils have the potential to shed light on these issues.

We here describe a newly discovered theropod bone from the very end of the Cretaceous (within 200,000 years of the mass extinction) of the Ibero-Armorican island. Its unique combination of anatomical characters allows its identification as a new species. Furthermore, its histology shows a growth pattern in which it grew fast in early ontogeny but reached subadult size quickly. The new species appeared within the faunal turnover on the island in the early Maastrichtian, which helps define the origins and timings of the migratory waves that brought the newcomers to the European archipelago.

**Figure 1:**
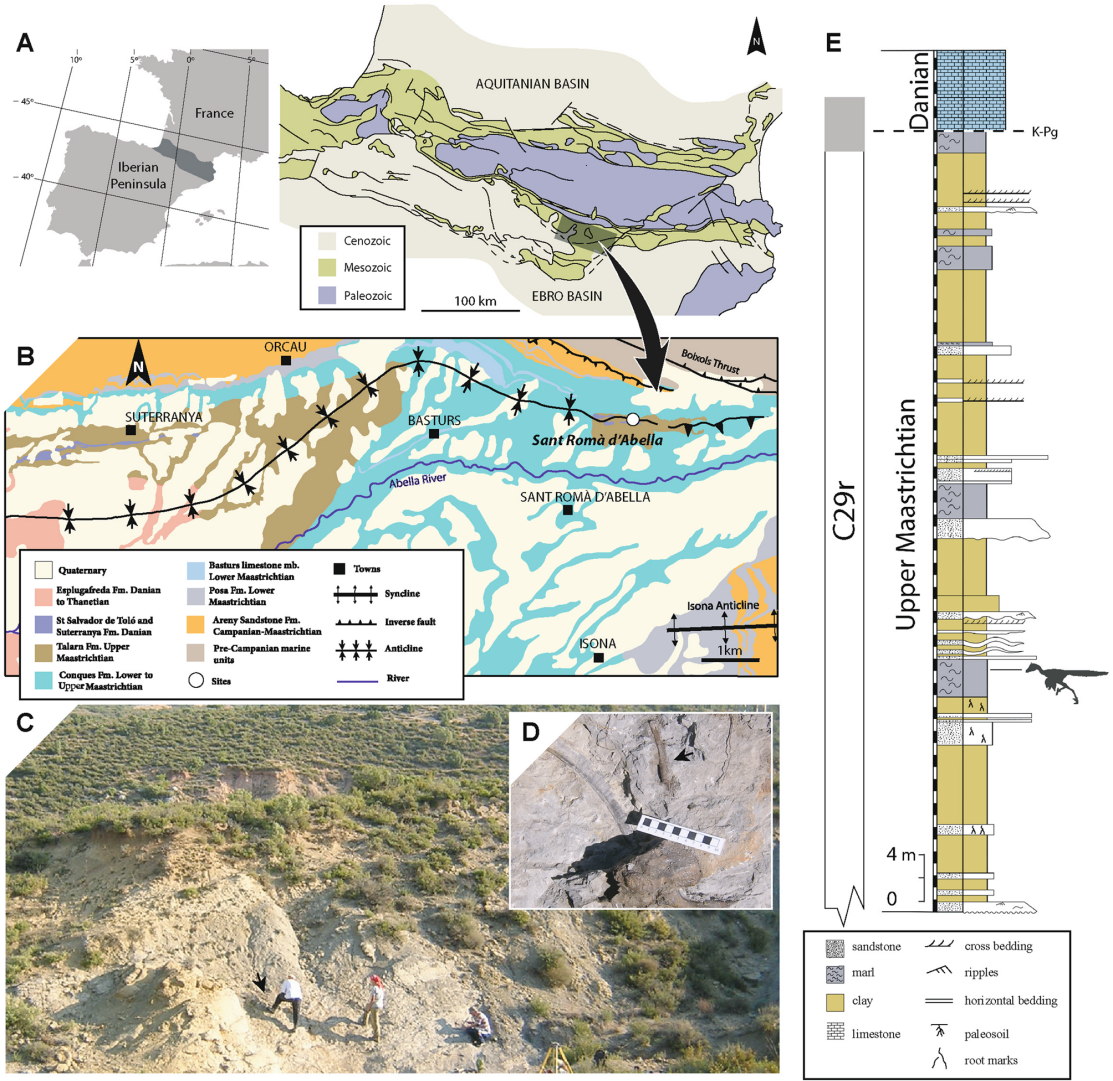
Geographic and geological location of Sant Romà d’Abella site. Geographic location of the Tremp Syncline in the southern Pyrenean region (**A**). Geological map of the eastern part of the Tremp Syncline with the location of the Sant Romà d’Abella site (**B**). Landscape view of the fossil site (**C**) with a detailed picture showing the close spatial relationship between the troodontid metatarsal (MCD-7073) and axial elements of *Pararhabdodon isonensis* (**D**). Stratigraphic section at Sant Romà d’Abella area with the precise position of the fossiliferous Sant Romà d’Abella site (**E**).

The specimen was discovered in September 2003 by a team of palaeontologists from the Museu de la Conca Dellà (MCD) at the Sant Romà d’Abella site (Fig. 1). According to the most recent stratigraphic data^15^, the Sant Romà d’Abella site falls within the upper part of the fluvial Talarn Formation of the Tremp Group (Fig. 1B). The fossil-bearing horizon of the Sant Romà d’Abella site is located in a 1.5-m-thick greyish marl layer (Fig. 1C), which is part of a 18 m-thick sedimentary sequence related to the development of a fluvial floodplain^30^. The fossiliferous horizon has yielded abundant plant remains, and the type specimen of the tsintaosaurin lambeosaurine *Pararhabdodon isonensis*^31–33^. The theropod specimen was found a few decimetres from bones of *P. isonensis* (Fig. 1D) and is the only other vertebrate remain in the site*.* Based on magnetostratigraphic, and biochronologic calibrations, the Sant Romà d’Abella site falls within the C29r magnetochron (latest Maastrichtian), thus placing the site within the final 200 ka before the K-Pg boundary^15^ (Fig. 1E).

## Results

### Systematic palaeontology

Dinosauria Owen, 1842

Theropoda Marsh, 1881

Coelurosauria Huene, 1914

Maniraptora Gauthier, 1986

Troodontidae Gilmore 1924

? Jinfengopteryginae Turner, Makovicky, and Norell2012

*Tamarro insperatus* gen. et sp. nov.

urn:lsid:zoobank.org:act:ADDD1AB2-BC87-4A17-A5D9-78B42B29AAD2

urn:lsid:zoobank.org:act:88919F32-2AE8-4587-AB8F-5D5852FA7FF5

*Etymology*. *Tamarro*, Catalan word referring to a small and elusive fantastic creature from the regional (Pallars county) folklore. *Insperatus,* from the Latin word for “unexpected”, in reference to the unexpected discovery of the specimen.

**Figure 2:**
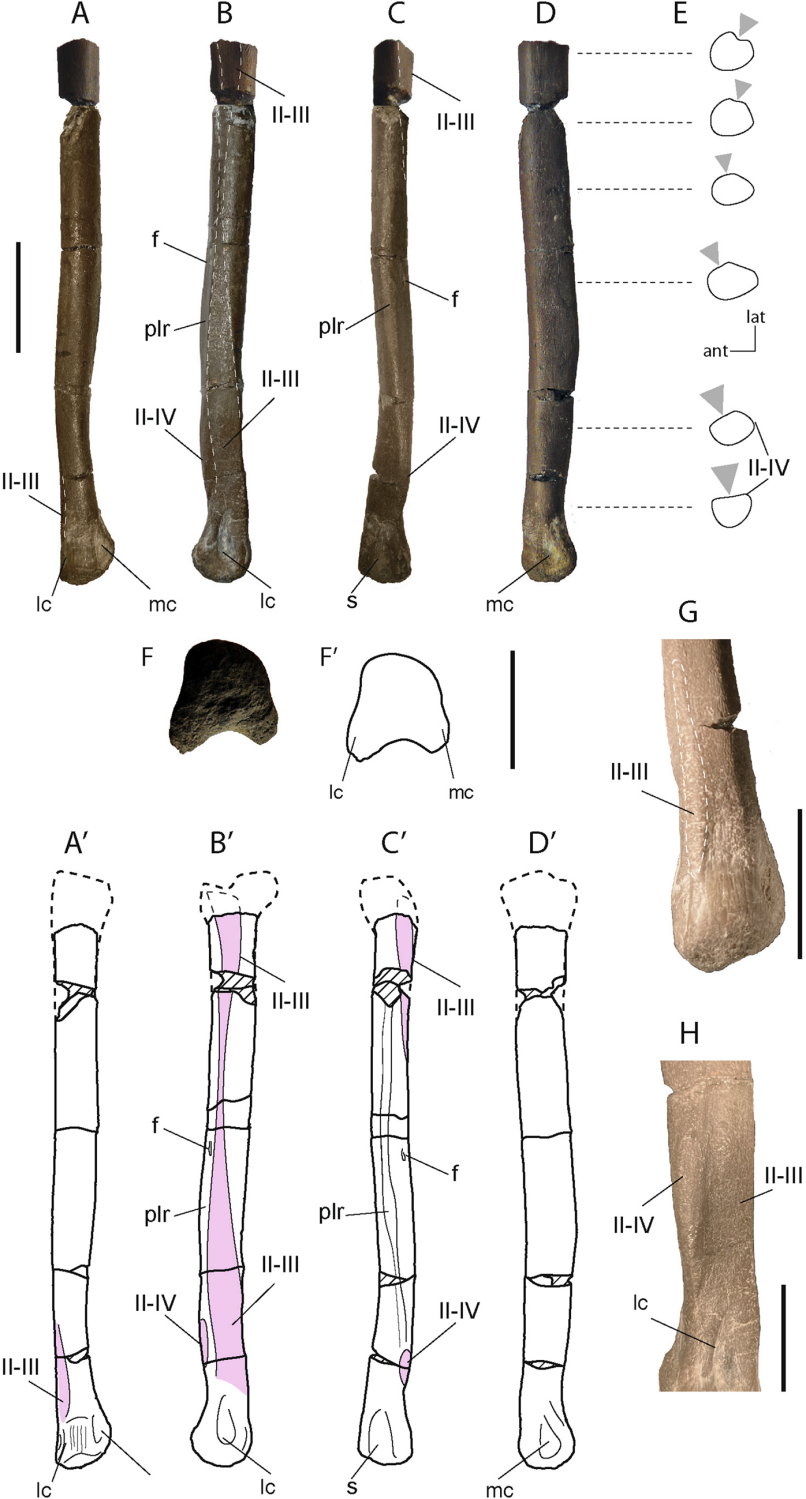
The European troodontid *Tamarro insperatus* (MCD-7073) gen. et sp. nov. from the uppermost Maastrichtian of the Tremp Group, Tremp Basin, Southern Pyrenees. Photographic and interpretative ilustrations of the right second metatarsal in anterior (**A-A’**), lateral (**B-B’**), posterior (**C–C’**), medial (**D-D’**), and distal (**F**-**F’**) views. Scheme illustrating the gradual transition of the articular surface–grey arrow–for the accommodation of the metatarsal III (**E**). Detail of the distal end of the metatarsal II in anterior view showing the anterior contact surface of the metatarsal III (**G**). Detail of the articular surface of the metatarsal IV in the posterodistal part of the second metatarsal (**H**) *ant* anterior; *f* foramen; *lat* lateral; *lc* medial condyle; mc, lateral condyle; *II–III* contact between the second and third metatarsals; *II–IV* contact between the second and fourth metatarsals; *plr* plantar ridge; *s* sulcus.

*Holotype* MCD-7073, a partial right metatarsal II (Fig. 2).

*Diagnosis Tamarro insperatus* is a mid-sized basal troodontid distinguished by the following unique combination of characters (* marks potential autapomorphies): metatarsal II with marked plantar ridge; small foramen on the lateral surface of the plantar ridge of the metatarsal II*; sub-arctometatarsalian condition with the metatarsal III restricted to the plantar margin on its proximal part.

*Type locality and age* Sant Romà d’Abella locality (Pallars Jussà, Catalonia); upper part of the Talarn Formation of the Tremp Group. The site is correlated with the C29r magnetochron^14^, latest Maastrichtian age.

*Nomenclatural acts:* The electronic version of this article in Portable Document Format (PDF) will represent a published work according to the International Commission on Zoological Nomenclature (ICZN), and hence the new names contained in the electronic version are effectively published under that Code from the electronic edition alone. This published work and the nomenclatural acts it contains have been registered in ZooBank, the online registration system for the ICZN. The ZooBank LSIDs (Life Science Identifiers) can be resolved and the associated information viewed through any standard web browser by appending the LSID to the prefix http://zoobank.org/. The LSID for this publication is: [urn:lsid:zoobank.org:pub:4EA36DCA-EF96-4826-8167-BCF1357FE445]. The online version of this work is archived and available from the following digital repositories: PubMed Central and CLOCKSS.

### Description

MCD-7073 is a right metatarsal II that lacks its proximal end (Fig. 2). Its bone surface is textured with a pattern of interweaving striations oriented parallel to the long axis of the element. In anterior view (Fig. 2A), it has a long, slender, and nearly straight diaphysis, which is moderately constrained mediolaterally on its distal half. While the lateral surface of the bone remains nearly straight and flat, the medial one is slightly convex and bows medially to the distal end. At shaft mid-length, the cross-section of the metatarsal is mediolaterally compressed to oval in shape, with the long axis oriented anteroposteriorly (Fig. 2E). The distal articulation facet is asymmetrical and inclined mediolaterally relative to the long axis of the shaft. This geometric configuration is produced by the differential proximodistal development of the distal condyles, in which the lateral one is larger than the medial one. The anterior surface of the distal articulation lacks the characteristic extensor sulcus of a ginglymoid joint. Instead, the medial and lateral condyles are connected anteriorly by a convex surface (Fig. 2A,H) textured with longitudinal striations. A subtle rugose lip is located on the lateral margin of the distal end of the metatarsal, which is here interpreted as the anterodistal contact with the metatarsal III (Fig. 2F). This structure indicates that the third metatarsal partially covered the anterior surface of the metatarsal II.

Laterally, the shaft of the metatarsal increases gradually in its depth distally until the mid-length of the diaphysis where it begins to taper towards the distal end. This results in a posteromedial ridge that occupies almost half of the preserved length. As a consequence, a posterolaterally-facing facet is exposed along the posterior side of the autopodium (Fig. 2B). A small foramen is observed adjacent to the upper part of the posteromedial ridge. The lateral surface of the metatarsal II possesses a smooth facet for articulation with the medial surface of the metatarsal III (Fig. 2B). This surface originates near the proximodistal side of the shaft as a shallow concave facet that quickly tapers distally above the level of the proximal end of the posteromedial ridge. Then, it continues as a narrow flat surface almost to the level of the nutritional foramen, when it expands anteroposteriorly until reaching the lateral condyle of the distal articulation (Fig. 2B). Near the distal end, a small posterolaterally-directed, oval facet is located between the posterior edge of the articular surface for the metatarsal III and the plantar ridge. This low-relief surface is interpreted as the posterior contact between the second and the fourth metatarsals (Fig. 2F). The distal articulation is well developed posteriorly, and there is an enlarged and deep collateral ligament fossa. In medial view, the metatarsal is slightly crescentic in shape, mainly because of the anteroposterior development of the posteromedial ridge. The medial collateral ligament fossa is well developed, having a teardrop-like shape, but it is smaller and shallower than the lateral ligament fossa.

The metatarsal is straight in posterior view (Fig. 2C), disrupted medially only by the edge of the posteromedial ridge, and distally by the medial deflection of the medial condyle. In this view, the facet for the metatarsal III twists from the mid-shaft of the bone toward the proximal end, and the articular surface for metatarsal is well exposed in the distal part of the bone (Fig. 2F). The distal articular surface is characterized by a deep flexor grove that separated the distal condyles. The articular facet for the metatarsal-phalangeal joint is markedly rounded anteriorly and concave posteriorly, bestowing a rounded horseshoe-like shape in distal view (Fig. 2H).

### Comparisons

Several taxa of small-sized theropods have been described from the upper Cretaceous formations of southwestern Europe, including the deinonychosaurians *Pyroraptor* and *Richardoestesia*, the enigmatic *Paronychodon*, and indeterminate velociraptorines^14^, all of which are represented by highly fragmentary remains, in some cases solely teeth. Other small theropods, notably the dromaeosaurid *Balaur*^5,6^ and the enigmatic *Elopteryx*^34,35^, *Heptasteornis*^36^*, and Bradycneme*^37^ are known from similar-aged deposits in south-eastern Europe (Romania). *Balaur* is represented by a relatively complete, associated, and well-preserved holotype skeleton, which provides the best glimpse at the anatomy of a latest Cretaceous European theropod^5,6^.

The second metatarsal from Sant Romà d’Abella (MCD-7073) can be compared to these, and other, small-bodied Cretaceous theropods. Although fragmentary, this metatarsal exhibits a series of distinctive anatomical features that allow us to assess its taxonomic affinity. These key features are: (1) the lack of a distal ginglymoid joint, (2) a sub-arctometatarsalian condition, (3) the straightness of the distal end (unbowed medial margin), (4) distal constriction of the shaft, (5) small distal articular joint with a horseshoe-like outline in distal view.

First, MCD-7073 lacks the characteristic ginglymoid joint end, which is considered an unequivocal dromaeosaurid synapomorphy, present even in the most basal members of the group, including in the European *Pyroraptor olympius*^23^. Only a few dromaeosaurids, such as *Imperobator*^38,39^, the unenlagiid *Neuquenraptor*^40^, and the microraptorine *Zhongjianosaurus*^41^, lack or show a very poorly developed ginglymus in Mt II^24^. Metatarsal II is also not ginglymoid in *Balaur*^5,6^ (but some authors disagree with the dromaeosaurid affinities of *Balaur*^42^). Although it cannot be completely ruled out that MCD-7073 is a rare and unusual dromaeosaurid without a ginglymoid metatarsal II, we consider this unlikely. Even if it was an aberrant dromaeosaurid, MCD-7073 could be distinguished from the contemporaneous European dromaeosaurid *Pyroraptor olympius* in that the second metatarsal of the latter is characterized by a concave posterior margin^22,23^, a feature not present in MCD-7073. Additionally, MCD-7073 is strikingly different from *Balaur*, which has much shorter and more robust metatarsals that are covered with bizarre textures^5,6^. Accordingly, and primarily based on the lack of a ginglymoid articulation, the second metatarsal from Sant Romà d’Abella cannot be confidently attributed to a dromaeosaurid deinonychosaurian.

The anatomical relationship between metatarsals II and III within the metatarsus can be inferred by scrutinizing the morphology of the interosseous contact along the lateral surface of MCD-7073. As described, the articular surface for the accommodation of the third metatarsal on the shaft of the second one extends from the posteroproximal to the anterodistal side of the lateral side, increasing its depth distally (Fig. 2B). This configuration indicates that the third metatarsal was posteriorly displaced near the proximal end of the metatarsus –but not necessarily excluded from the anterior face– whereas it was clearly exposed from the mid-length to the distal part of the tarsometatarsus. The preserved proximal part of MCD-7073 is laterally concave and further indicates that the medial margin of the proximal end of metatarsal III was convex, and not reduced to a thin bar of bone (Fig. 2E). Besides, the occurrence of a distal articular facet for metatarsals II–IV contact in the posterodistal part of MCD-7017 (Fig. 2G) suggests that metatarsal III was forced to reduce its contribution to the posterodistal surface of the pes. Overall, this anatomical arrangement resembles the sub-arctometatarsalia exhibited by basal troodontids (i.e. *Sinovenator changii, Sinornithoides youngi,* and MPC-D 100/140)^43,44^, and caenagnathid oviraptorosaurians (i.e. *Elmisaurus rarus* and *Citipes elegans*)^45^; but differs from the arctometatarsalian morphology of derived ornithomimosaurians and the hyper-arctometatarsalian nature of parvicursorine alvarezsaurians^43,46,47^. On the other hand, basal alvarezsaurians display the plesiomorphic condition on the arrangement of the metatarsals, and only *Achillesaurus* and *Alnashetri* show third metatarsals slightly appressed between metatarsals II and IV, but not restricted to the ventral part of the shaft as in MCD-7073 (see Fig. S1).

One of the most salient features of MCD-7073 is the straightness of the lateral side of the shaft, especially on its distal end. As observed in MCD-7073, the lateral condyle remains unbowed regarding the long axis of the metatarsal, while the medial one is medially deflected. This arrangement in the orientation of the distal condyles is present in troodontids (i.e. *Talos, Latinevenatrix, Philovenator*)^48–50^ and caenagnathids^45^, but differs from the condition observed in ornithomimosaurians (i.e.*, Aeopyornithomimus, Ornithomimus*, *Qiupalong*)^51–53^ and avimimids^54^, where the two distal condyles are medially tilted due to the mediolateral bowing of the distal fifth of the diaphysis of metatarsal II. In addition, most of derived alvarezsaurids have straight distal epiphyses (i.e. *Alvarezsaurus, Kol, Parvicursor, Nemegtonykus*), which resemble the condition of MCD-7073. But alvarezsaurids lack the medial deflection of the medial distal condyle and the constriction of the distal end that exhibits the studied specimen. Furthermore, as above stated, they also differ from MCD-7073 in the anatomical relationship of metatarsals (hyper-arctometatarsalia). Because of that, it seems unlikely that MCD-7073 could be referred to this particular group of maniraptorans (see Fig. S1).

MCD-7073 shows a marked anteroposterior development on its mid-shaft, resulting in a posteromedial plantar ridge. This ridge is an uncommon feature among coelurosaurs generally, and when it is present, it is usually not as developed as in MCD-7073. Caenagnathids are distinguished by strong and well-developed posterior ridges on both Mt II and Mt IV^45^, which are more developed and more distally located on the shafts than in MCD-7073. In part, the different location of the plantar ridge is enabled by the occurrence of a marked mediolateral constriction in the distal part of the diaphysis of MCD-7073, just above the distal condyles. In caenagnathids, the mediolateral width remains nearly invariable along the shaft, thus bestowing a more robust aspect to the second metatarsal than in MCD-7073. In addition, MCD-7073 differs from this family of oviraptorosaurians in the morphology of the distal articulation, which is rounded and nearly spherical in caenagnathids^45^, while it is horseshoe-shaped in MCD-7073 (see Fig. S1).

A posteromedial ridge on the second metatarsal is observed in some basal troodontids^44,50,55,56^, while in more derived taxa that have very thin second metatarsals, the posterior ridge is reduced^48,49,57^. In this regard, MCD-7073 resembles the basal condition within Troodontidae. Finally, the reduced size of the metatarsal II, relative to metatarsals III and IV, is characteristic of most troodontids. This constriction is not restricted to the diaphysis, but also to the distal articulation. As a consequence, in distal view, the articular surface of the distal joint is anteroposteriorly deeper than mediolaterally wide, giving it a horseshoe-shape as in MCD-7073 (see Fig. S1).

Summarizing these comparisons, MCD-7073 shares features with basal troodontids (straight shaft, sub-arctometatarsalian morphology, unbowed distal end, horseshoe-shaped distal articulation), and has notable differences with other clades of Cretaceous small theropods. Additionally, MCD-7073 possesses a potential autapomorphic feature not reported in any other troodontid, the occurrence of a nutritional foramen on the posteromedial surface of the second metatarsal, which supports its designation as a new taxon, *Tamarro insperatus gen. et. sp. nov.*

urn:lsid:zoobank.org:act:ADDD1AB2-BC87-4A17-A5D9-78B42B29AAD2

urn:lsid:zoobank.org:act:88919F32-2AE8-4587-AB8F-5D5852FA7FF5

### Phylogenetic results

**Figure 3:**
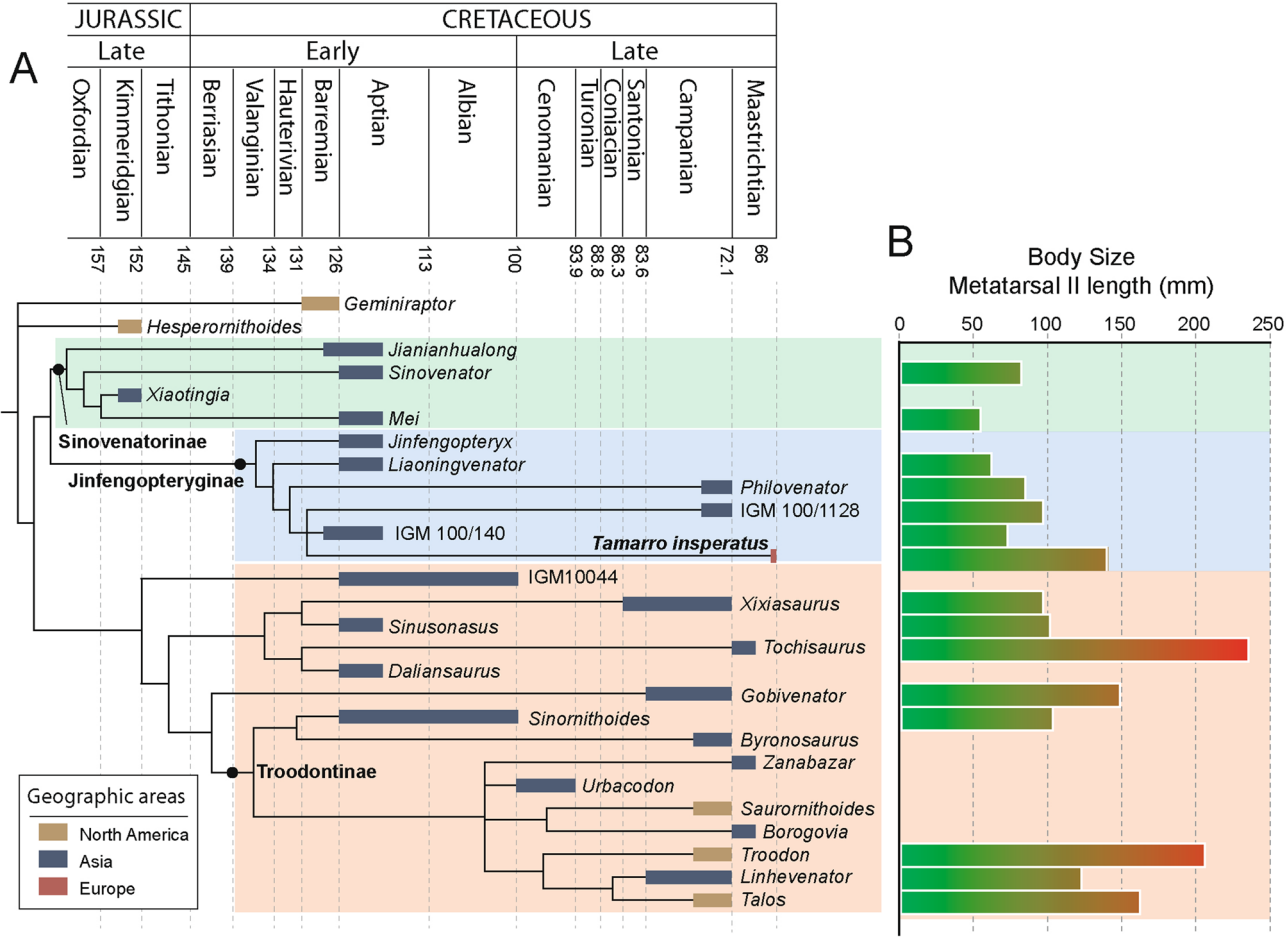
Phylogenetic relationships of *Tamarro insperatus* (MCD-7073) gen. et sp. nov. Simplified time-calibrate topology of the strict consensus phylogenetic trees (CI: 0.073, RI: 0.615) of 10 most parsimonious trees of 13,064 steps (**A**), and the evolution of body size using the length of the metatarsal II as a proxy (**B**). Additional data available in the Supplementary Table S2.

Our broad-scale coelurosaurian phylogenetic analysis based on Hartman et al.^58^ yielded 10 most parsimonious trees (MPTs) of 13,064 steps, with a consistency index (CI) of 0.073 and a retention index (RI) of 0.615. The strict consensus tree returned a well-resolved topology in which *Tamarro insperatus* was recovered in a polytomic relationship with the Mongolian troodontids MPC-D 100/1128 (sometimes referred as MPC-D 100/1126) and MPC-D 100/140 (Fig. 3). These three taxa are the most derived members of a group where *Jinfengopteryx*, *Liaoningvenator*, and *Philovenator* are recovered as consecutive taxa. This taxonomic grouping resembles that of Jinfengopteryginae defined by Turner et al.^23^. The identification of *Tamarro* as a jinfengopterygine seems to be supported by the plantar displacement of metatarsal III at its proximal end relative to the positions of metatarsals II and IV, a synapomorphic feature of this group of troodontids^23^.

Our phylogenetic results also agree with those of Hartman et al.^58^ in relating MPC-D 100/1128 and *Liaoningvenator*, but *Almas* (MPC-D 100/1323) is not recovered as a member of this sub-family, unlike Turner’s results^23^. In turn, Jinfengopteryginae is placed as the sister group of Sinovenatorinae and includes *Jianinhualong*, *Mei*, *Sinovenator*, and *Xiaotingian*. Despite some differences in the resulting topology, this relationship is similar to other previous phylogenetic results^23,58^. Perhaps, the most noticeable difference of our analyses is the location of *Philovenator curriei* as a jinfengopterygine; it is commonly considered the sister taxon of *Linhevenator tani,* and both are considered as derived troodontines^49,55,59–61^. Nevertheless, the recovery of *Tamarro insperatus* as a basal troodontid, most likely a jinfengopterygine, is in agreement with our anatomical comparisons.

### Histology

**Figure 4:**
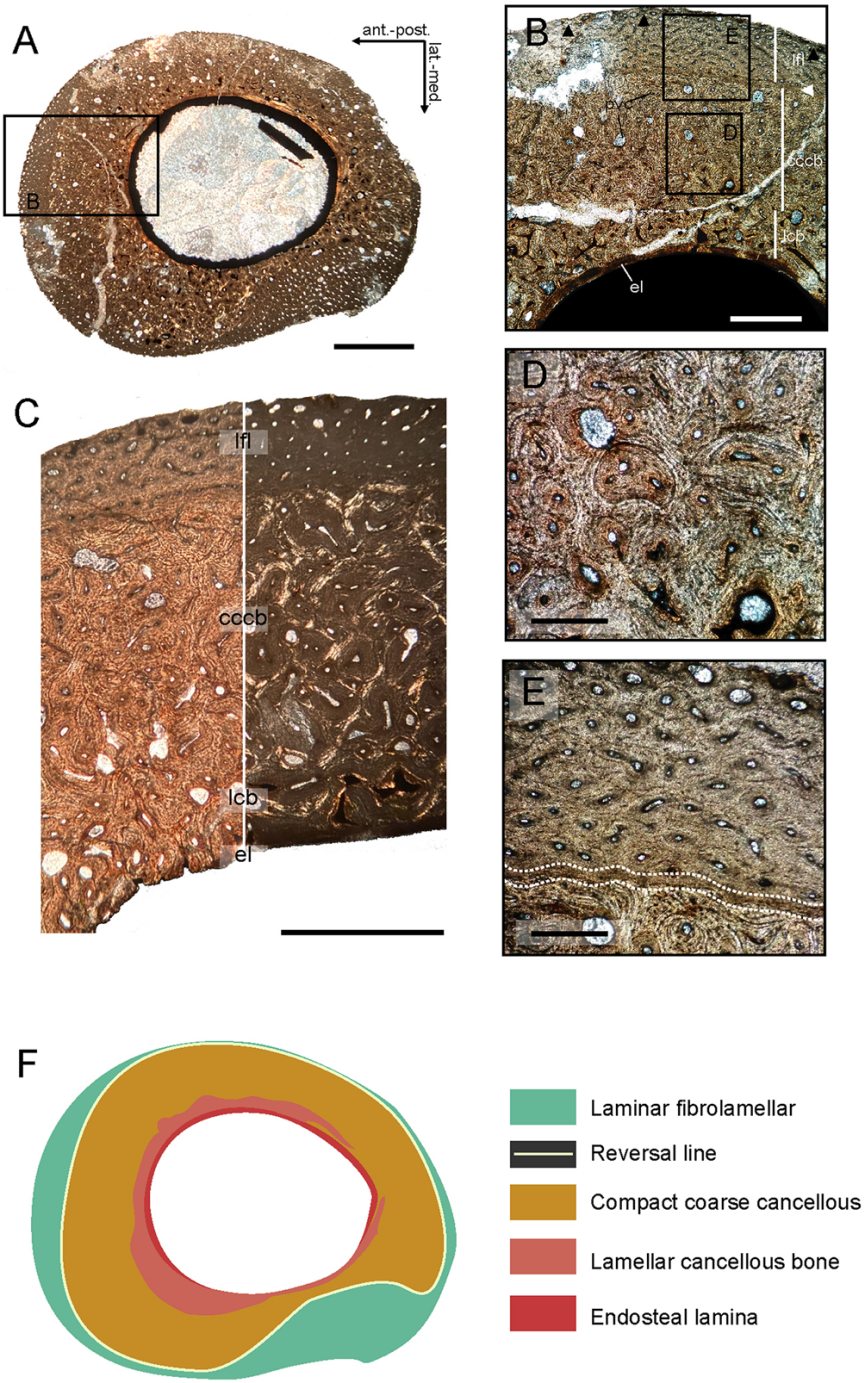
Osteohistology of *Tamarro insperatus* gen. et sp. nov. based on the osteohistological sample from the metatarsal II (MCD-7073). Full petrographic transverse thin section from the proximal mid-shaft of the Mt II (**A**). Detail of the cortex showing the predominance of primary lamellar and fibrolamellar bone tissues. Black arrows indicate the location of a LAG. White arrow shows the location of the reversal line (**B**). Outer cortex under transferred and polarized light displaying the distinctive arrangement of the bone tissues (**C**). Magnification of the inner cortex showing the infilling of primary cancellous spaces with lamellar bone (**D**). Osteohistological transition (white dashed lines) between the compact coarse cancellous bone of the inner cortex and the laminar fibrolamellar of the outer cortex (**E**). Type tissue map of areas representing different tissue types are colour coded for quantitative analyses (**F**). *cccb* compact coarse cancellous bone; *el* endosteal lamina; *lcb* lamellar cancellous bone; *lfl* laminar fibrolamellar; *pvc* primary vascular canal. Scale bar: 2 cm (**A**), 0.5 mm (**B**), 1 cm (**C**), and 0.2 mm (**D**,**E**).

Histological sectioning of the proximal shaft of MCD-7073 provides insights into the physiology of the new taxon. The cortex of the bone (133–185 μm thick) is primarily formed of primary lamellar and fibro-lamellar tissue, with predominantly longitudinal vascularity (Fig. 4A–C). A thin (16 μm) avascular layer of endosteal lamellar bone encircles the medullary cavity (Fig. 4B,C). The inner cortex consists of compact coarse cancellous bone (CCCB) with large primary longitudinal vascular canals (original cancellous spaces) filled with lamellar bone deposits (Fig. 4B–D); the inner cortex displays more cancellous vacuities close to the medullary cavity. The outer cortex is of vascularized laminar fibrolamellar bone with relatively dense longitudinal canals of small size (Fig. 4E). The transition between the inner and the outer cortex is demarcated by a reversal line (Fig. 4E). One LAG is observed in the outermost part of the periosteal region (Fig. 4B). Neither secondary osteons nor an external fundamental system (EFS) are observed. It is worth mentioning the asymmetric development of the second growth cycle, which is more pronounced on the anterior and the lateral sides of the bone (Fig. 4F).

## Discussion

The presence of troodontids in Europe has been debated for a long time, mainly because its record was entirely based on isolated teeth, until now. The oldest troodontid evidence in Europe dates back to the Early Cenomanian and is based on the discovery of one isolated tooth in western France^62^. Several troodontid-like and *Paronychodon* teeth (a *nomen dubium* taxa referred by some to Troodontidae)^63^ were recovered from the Campanian and Maastrichtian deposits of the ancient Hateg (Romania) and Ibero-Armorican (Portugal, France, and Spain) islands^8^. In Romania, the enigmatic fossils of *Bradycneme draculae* and *Elopteryx nopcsai* were referred to Troodontidae^63^, although later works ruled out this assignment^35,36^. Similarly, Torices et al.^64^ questioned the attribution of *Paronychodon* teeth to troodontids. Consequently, the discovery of *Tamarro insperatus* in the latest Maastrichtian deposits from southern Pyrenees represents the first unequivocal bone evidence of this group of small-sized non-avian theropods in Europe, and confirms the occurrence of troodontids in the theropod faunal assemblage of that continent.

### Ontogenetic stage, growth rate, and body size

According to the histological analysis, *Tamarro insperatus* shows some remarkable features among non-avian theropods. The primary bone of the specimen is mostly built on compact coarse cancellous and laminar fibrolamellar tissues with well-developed longitudinal vascularity, suggesting a high growth rate throughout ontogeny. This contrasts with the alternation of fibrolamellar and plexiform tissues observed in several coelurosaurians^65–68^. The occurrence of CCCB in *Tamarro* is likely because the histological sample was acquired near the proximal epiphysis of the bone^69^. The absence of EFS and Haversian structures suggests that *Tamarro* had not reached the somatic maturity at the time of its death. However, the occurrence of a well-developed endosteal lamina and the incorporation of compact coarse cancellous bone into the cortex indicate a mature stage of the individual and the cessation of the medullary expansion, and thus the attainment of subadult body size^69^. This interpretation is consistent with interweaving texture observed in the surface of the metatarsal II, a pattern associated with skeletal maturity in dinosaurs^65,70,71^.

**Figure 5:**
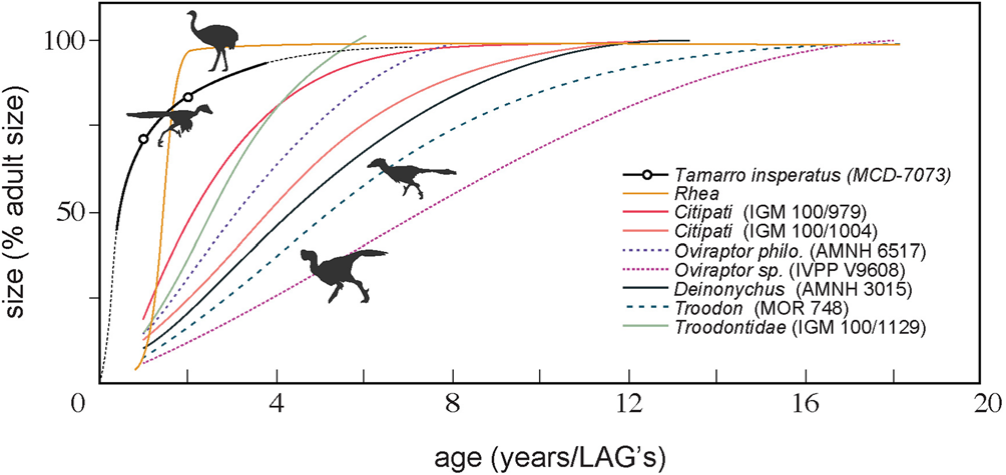
Growth curves of maniraptoran theropod dinosaurs. *Tamarro insperatus* outstands among other taxa for its fast initial growth, reaching subadulthood during the first year of life.

Our osteohistological observations suggest that *Tamarro* attained subadult body size much earlier than other non-avian theropods^67^ by its fast growth rate in early ontogeny, but similar to extant palaeognathid birds (Fig. 5). Although troodontids are known to be fast-growing^65,67^, similar rapid initial growth rates without bone remodelling as that of *Tamarro* has only been reported in *Mei long*, which attained somatic maturity at only two years^59^. *Liaoningvenator* reached that stage in four years^55^; while derived troodontids required more time to achieve adulthood (5–9 years; *Talos, Troodon*)^48,72^. In fact, the occurrence of laminar fibrolamellar tissues with well-developed longitudinal vascularity in *Tamarro insperatus* is more similar to the histological patterns of other basal troodontids (i.e. IGM 100/1129), than those of more derived ones (i.e. *Troodon*)^73^. If so, the histological evidence further supports our phylogenetic and anatomical results in interpreting *Tamarro* as a basal member of Troodontidae.

Troodontidae is a clade of small-bodied non-avian theropods that includes kiwi-size (i.e. 0.8 kg for *Mei long*)^74^ to rhea-size taxa (i.e. 47 kg for *Troodon formosus*)^74^. It is beyond the scope of this study to infer the full body size of *Tamarro*, but a preliminary approach can be performed by comparing the metatarsal II length among different troodontid taxa. The proximodistal length of metatarsal II of MCD-7073 was estimated at 140 mm, which is about the same size as *Gobivenator* (145 mm; MPC-D 100/86), and roughly similar to *Linhevenator* (120 mm; LH V0021), *Sinornithoides* (100.5 mm; IVPP V9612) and *Talos* (159 mm; UMNH VP 19,479). However, it is more than the double of *Mei* (51 mm; IVPP V12733), and nearly two times larger than its close phylogenetic relative (Fig. 2B and Table S2). If our phylogenetic interpretations are correct, *Tamarro* would be the largest, and one of the more derived jinfengopterygine yet discovered.

### Paleobiogeographic implications

Various biogeographic reviews on the Late Cretaceous faunas of Europe^8,75^ underscored the likely presence of troodontids in the archipelago, based exclusively on teeth. Thus, in addition to the Campanian and Maastrichtian reports from Portugal, Romania, and Spain, an early occurrence of the group was reported from the Cenomanian of France^62^. The fragmentary and disparate fossil material of European troodontids prevents a clear correlation between the Cenomanian and Maastrichtian taxa, but the phylogenetic cluster in which *Tamarro insperatus* is included supports the idea that the clade migrated to Europe from Asia (Fig. 3A). The timing of such dispersal that brings the Maastrichtian troodontids to eastern (tooth evidence from Hateg island, Romania) and western Europe (*Tamarro insperatus* and tooth evidence from Ibero-Armorican island, Spain and Portugal) is, however, difficult to determine precisely. The Maastrichtian troodontids could have first reached Europe in the Cenomanian and persisted on the islands until the end of the Cretaceous; or alternatively, they could have arrived in the two migratory waves that brought Asian faunas to the archipelago. More particularly, this dispersal would have taken place during the Coniacian–Santonian or in a series of events that occurred around the Campanian–Maastrichtian boundary, most probably in the early Maastrichtian, a time when new immigrants triggered a dinosaur turnover in western Europe (Ibero-Armorican island)^8^.

## Conclusions

The discovery of the new basal troodontid *Tamarro insperatus* at the very end of the Cretaceous in Europe represents the first evidence of bones of Troodontidae in Europe. Both phylogenetic and osteohistological results support the basal nature of the new taxon, most likely as a representative of the Asian clade Jinfengopteryginae. The histological analysis also has provided evidence for a mature individual with a high growth rate, which allowed the animal to attain subadult body size in a very short time. This growth strategy is similar to other basal troodontids, such as the Asian *Mei long*.

The fragmentary, yet controversial, record of troodontid-like teeth from Europe together with the likely Asian affinity of *Tamarro*, confirm that troodontids could have reached the ancient European Archipelago during the Late Cretaceous. Furthermore, the relatively large size of *Tamarro insperatus* compared with its closest phylogenetic relatives suggests it would be the largest among jinfengopterygines.

## Methods

### Material and terminology

Right metatarsal II (MCD-7073), housed at the paleontological collection of the Museu de la Conca Dellà (MCD) in Isona, Pallars Jussà, Catalonia, southern Pyrenees. The director of MCD, A.G.–which is also a co-author of the present study – granted access to the collection under his care and provided permission the study and graphic reproduction of the specimen.

### Thin section and histological analyses

A histological sample was taken from the proximal region of the shaft of the Mt II. The samples were exposed using a Buehler Isomet low-speed saw and subsequently polished on a glass sheet coated with carborundum powder, using decreasing particle sizes of 600, 800, and 1000 grit. The bone sample was fixed to a frosted glass slide using ultraviolet curing glue Loctite 358. The ground section was then prepared with a diamond saw (Buehler, PetroThin) to a final thickness of approximately 60 µm. The slice was dehydrated through a graded series of alcohol baths, cleared in Histo-Clear II for five minutes, and finally mounted in a DPX mounting medium. The thin section was observed under transmitted and polarized light using a petrographic microscope Nikon Eclipse E400 POL connected to a digital camera Nikon DS-FI3.

### Phylogenetic analyses

In order to establish the phylogenetic relationships of *Tamarro insperatus*, phylogenetic analysis of the new taxon was added to the large-scale theropod analysis of Hartman et al.^58^. MCD-7073 was coded using Mesquite 3.61^76^, comprising 502 species-level theropod taxa and 700 morphological characters, and analysed using TNT 1.1^77^ to find the most parsimonious trees (MPTs). For our exploration, we set the maximum tree saved in memory at 10,000 and used a traditional search, performing 10,000 replications of Wagner trees (using random addition sequences) followed by tree bisection reconnection (TBR) as swapping algorithm, saving 10 trees per replicate.

All figures, silhouettes, and drawings that accompany this study were created by the authors using Adobe Illustrator CC 2015 2.1.

## Supplementary Information

Supplementary Information.
