# Supplementary material for: A fast-growing basal troodontid (Dinosauria: Theropoda) from the latest Cretaceous of Europe
Source: Sci Rep. 2021 Mar 1;11:4855. doi: 10.1038/s41598-021-83745-5 (PMC7921422; doi:10.1038/s41598-021-83745-5)
Supplement: Supplementary file 1 — Supplementary Information. [file 41598_2021_83745_MOESM1_ESM.pdf]

Supplementary Information

A fast-growing basal troodontid (Dinosauria: Theropoda) from the latest Cretaceous of Europe.

Albert G. Sellés, Bernat Vila, Stephen L. Brusatte, Philip J. Currie, Àngel Galobart

Table S1. Measurements (mm) of the second metatarsal (MCD-7073) of *Tamarro insperatus*. Estimated values are indicated between brackets.

Maximum length of the metatarsal II	10.52 (14.0)
Maximum depth (proximodistal) of the mid-shaft of metatarsal II	8.89
Maximum width (lateromedial) of the mid-shaft of metatarsal II	6.17
Maximum depth of the distal articulation of metatarsal II	9.85
Maximum width of the distal articulation of metatarsal II	9.55

Table S2. Measures (mm) of the length of the second metatarsal in selected Troodontidae taxa used as a proxy of relative body size. Data was primarily extracted from the Theropod Database developed by Mickey Mortimer (www.therpoddatabase.com 11/10/20).

Sub-groups	Taxa	Specimen	MtII length
Sinovenatorinae	*Sinovenator changii*	IVPP V 12615	77
Sinovenatorinae	*Sinovenator changii*	IVPP V12583	79
Sinovenatorinae	*Mei long*	DNHM D2514	42.5
Sinovenatorinae	*Mei long*	IVPP V12733	51
Jinfengopterigine	*Jinfengopteryx elegans*	CAGS-IG-04-0801	59
Jinfengopterigine	*Liaoningvenator curriei*	DNHM D3012	82
Jinfengopterigine	*Philovenator currei*	IVPP V10597	94
Jinfengopterigine	Unnamed	IGM 100/1128	71.8
Jinfengopterigine	***Tamarro insperatus* gen. et sp. nov.**	**MCD-7073**	**140**
Derived troodontidae	*Sinusonasus magnodens*	IVPP V11527	93.5
Derived troodontidae	*Daliansaurus liaoningensis*	DNHM D2885	98.1
Derived troodontidae	*Tochisaurus nemegtensis*	PIN 551-244	233
Derived troodontidae	*Gobivenator mongoliensis*	IGM 100/86	145.2
Derived troodontidae	*Sinornithoides youngi*	IVPP V9612	100.6
Derived troodontidae	*Troodon formosus*	MOR 748	202.4
Derived troodontidae	*Linhevenator tani*	LH V0021	120
Derived troodontidae	*Talos sampsoni*	UMNH VP 19479	158.86

Figure S1. Morphological comparison of the metatarsal II in maniraptoran theropods. Each taxon is represented in dorsal, medial, and distal view. Main anatomical features that allow the taxonomic classification of *Tamarro insperatus gen. et sp. nov*. are indicated for each selected species if present. The arctometatarsalia condition and its variations are illustrated as the spatial configuration of the contact surfaces of Mt III and IV upon the medial surface of Mt II. All silhouettes were created by the authors using Adobe Illustrator CC 2015 2.1.

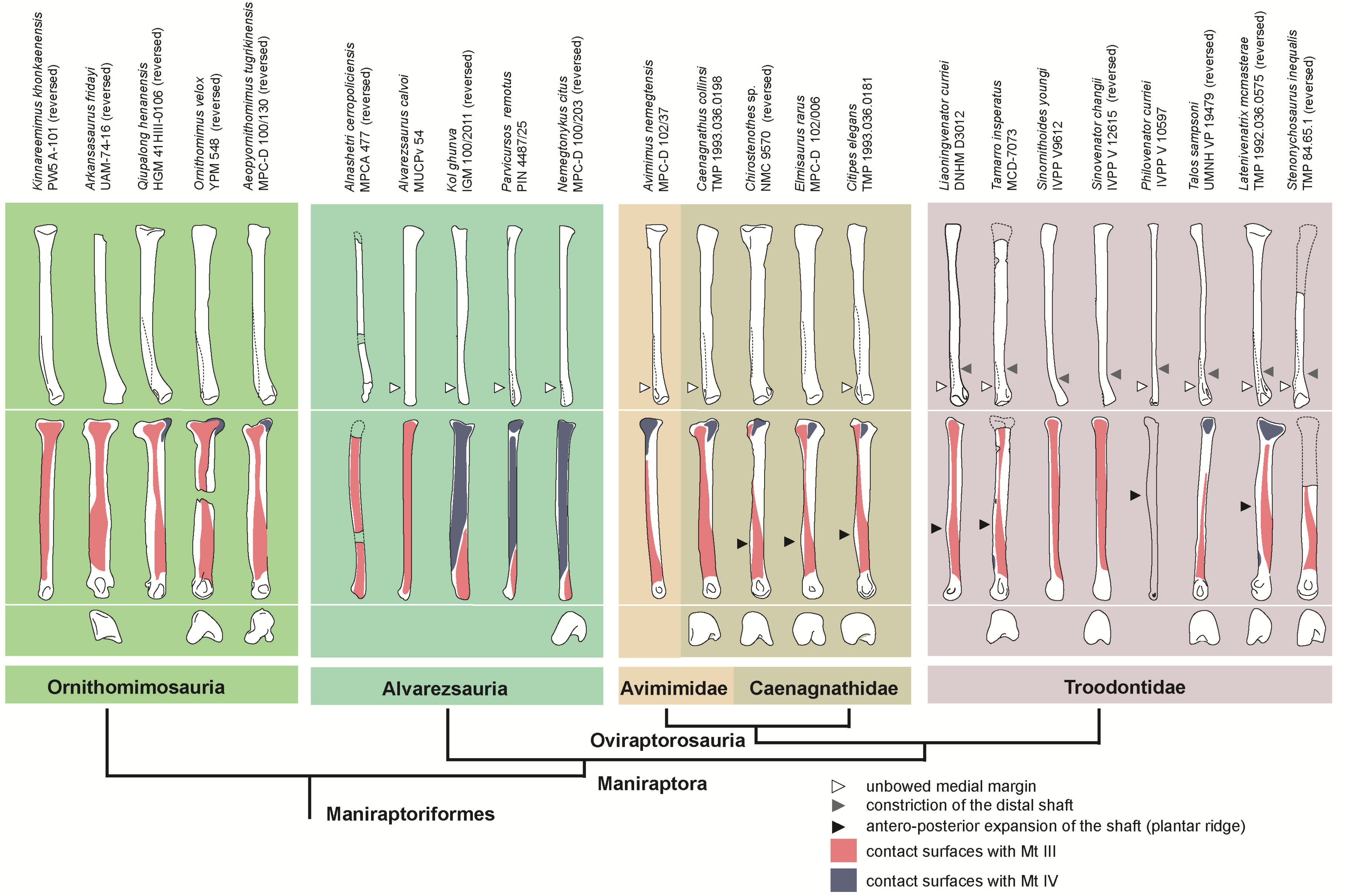

Supplementary Text 1. Nexus file used in the phylogenetic exploration of *Tamarro insperatus gen. et. sp. nov*. The matrix is based on Hartman et al.

```
#NEXUS

BEGIN TAXA;
	TITLE Taxa;
	DIMENSIONS NTAX=502;
	TAXLABELS
		Dilophosaurus Sinosaurus Cryolophosaurus Tachiraptor Berberosaurus Genyodectes Ceratosaurus
Eoabelisaurus Rugops Rajasaurus Genusaurus Arcovenator Majungasaurus Dahalokely Rahiolisaurus
MCF_PVPH_237 Xenotarsosaurus Ilokelesia Ekrixinatosaurus Skorpiovenator Viavenator Carnotaurus Aucasaurus
Abelisaurus Noasaurus Velocisaurus Masiakasaurus Elaphrosaurus Afromimus Limusaurus Deltadromeus Gualicho
Angeac_taxon Chuandongocoelurus Monolophosaurus Pandoravenator Piatnitzkysaurus Condorraptor Marshosaurus
Poekilopleuron Duriavenator Megalosaurus Torvosaurus Wiehenvenator Dubreuillosaurus Afrovenator Leshansaurus
Eustreptospondylus Cristatusaurus Baryonyx Ichthyovenator Irritator Spinosaurus_sensu_lato Kaijiangosaurus
Erectopus Xuanhanosaurus Yangchuanosaurus Shidaisaurus Metriacanthosaurus Sinraptor_dongi hepingensis
Siamotyrannus MNNGAD_Kryptops_postcran Allosaurus Saurophaganax Neovenator Acrocanthosaurus Labocania
Shaochilong Concavenator Kelmayisaurus Carcharodontosaurus iguidensis Tyrannotitan Mapusaurus Giganotosaurus
Datanglong Gasosaurus Lourinhanosaurus Chilantaisaurus Fukuiraptor Australovenator Megaraptor Murusraptor
Orkoraptor Aerosteon Kileskus Proceratosaurus Guanlong Mirischia Sinotyrannus Yutyrannus Dilong Juratyrant
Xiongguanlong Eotyrannus Alectrosaurus Appalachiosaurus Bistahieversor Raptorex Gorgosaurus Alioramus
Tyrannosaurus Coelurus Tanycolagreus Aristosuchus Bicentenaria Xinjiangovenator Zuolong Zuni Ornitholestes
Sinocalliopteryx Huaxiagnathus Juravenator Aniksosaurus Archaeornithoides Scipionyx Tugulusaurus Sinosauropteryx
Santanaraptor Haplocheirus Compsognathus Sciurumimus NGMC2124 Aorun Naze_dromaeosaur Deinocheirus
Hexing Shenzhousaurus Harpymimus Nedcolbertia Arkansaurus Archaeornithomimus_asiaticus GIN960910KD
Beishanlong Garudimimus Aepyornithomimus Sinornithomimus Ornithomimus Cerro_del_Pueblo_orn Tototlmimus
mongoliensis MNAPl1762A sedens_holotype_only Rativates Struthiomimus Gallimimus_bullatus Timimus Anserimimus
Qiupalong Dromiceiomimus Kinnareemimus Fukuivenator Nqwebasaurus Pelecanimimus Patagonykus Xiyunykus
Bonapartenykus Bannykus Alvarezsaurus Achillesaurus Alnashetri Albertonykus Ceratonykus Qiupanykus Albinykus
Xixianykus Linhenykus Heptasteornis Bradycneme Parvicursor Tugrik_parvicursorine Shuvuuia Mononykus
Martharaptor Falcarius Jianchangosaurus Beipiaosaurus Alxasaurus Enigmosaurus zheziangensis Suzhousaurus
Neimongosaurus Erliansaurus Therizinosaurus_minus_hindlimb Nanchao_embryos Nanshiungosaurus_brevispinus
bohlini AMNH6368 Segnosaurus Erlikosaurus Nothronychus_mckinleyi graffami Protarchaeopteryx Similicaudipteryx
Ganzhousaurus Caudipteryx Shanyangosaurus Kol Avimimus Machairasaurus Caenagnathasia_dentaries_only
Hagryphus Apatoraptor Leptorhynchos_gaddisi Elmisaurus_rarus elegans Chirostenotes_sensu_lato Anzu Luoyanggia
Incisivosaurus Microvenator Beibeilong Gigantoraptor Tongtianlong Oviraptor Banji Yulong Huanansaurus Wulatelong
Corythoraptor NXMV Nomingia Rinchenia Nankangia IVPPV9608 IGM10042 Citipati Anomalipes Shixinggia Khaan
Conchoraptor Jiangxisaurus Nemegtomaia Heyuannia yanshini Scansoriopteryx Yi Epidexipteryx Pedopenna Caihong
Serikornis Anchiornis Aurornis Eosinopteryx Ostromia Archaeopteryx Shanag BYU2023 ISMDVP09 Halszkaraptor
Mahakala Ningyuansaurus Austroraptor Buitreraptor Pamparaptor Ornithodesmus Pyroraptor Rahonavis
Unenlagia_sensu_lato Dakotaraptor Mei Xiaotingia Sinovenator Jianianhualong IGM100140 IGM100972_100974 Almas
Jinfengopteryx Liaoningvenator IGM1001128 IGM10044 Daliansaurus Tochisaurus Hesperornithoides Sinusonasus
Xixiasaurus Byronosaurus Sinornithoides IVPPV11119 Gobivenator Urbacodon_mandibular_only Geminiraptor
Borogovia Saurornithoides Zanabazar Troodon_sensu_lato Talos Tamarro_insperatus Philovenator Linhevenator
Tianyuraptor Zhenyuanlong Acheroraptor Bambiraptor Variraptor Sinornithosaurus Microraptor Zhongjianosaurus
osmolskae NGMC91 Changyuraptor Graciliraptor Yurgovuchia Deinonychus Utahraptor Achillobator Yixianosaurus
Pneumatoraptor Saurornitholestes_langstoni Atrociraptor IGM10022_10023 Itemirus_holotype_only Linheraptor
Tsaagan Dromaeosaurus Hulsanpes IGM100980 Velociraptor_mongoliensis Luanchuanraptor Adasaurus DGBU_78
Zhongornis Hesperonychus_holotype_only Balaur Bauxitornis Sapeornis Zhongjianornis jianchangensis Jeholornis
Jixiangornis Yangavis Eoconfuciusornis Proornis Confuciusornis_sanctus dui chuonzhous Elopteryx Changchengornis
Chongmingia Yandangornis Jinguofortis Wyleyia Protopteryx Paraprotopteryx Orienantius Eopengornis Pengornis
Elsornis Otogornis Yungavolucris Noguerornis Iberomesornis LP_4450_IEI Concornis Cruralispennia Bohaiornis
Parabohaiornis Longusunguis Shenqiornis Sulcavis Zhouornis Fortunguavis Dunhuangia Yuanjiawaornis Eoenantiornis
Huoshanornis Grabauornis Jibeinia Hebeiornis Gobipipus Gobipteryx Nanantius Gurilynia Holbotia Evgenavis
Enantiophoenix Parvavis Monoenantiornis chabuensis Junornis Eocathayornis Houornis aberransis
Cathayornis_yandica Sinornis Linyiornis Gracilornis Alethoalaornis Cuspirostrisornis Dapingfangornis Largirostrornis
Longchengornis Microenantiornis Piscivorenantiornis Pterygornis Dalingheornis GMV2158 GMV2159 Liaoxiornis
Avisaurus Gettyia Mirarce Soroavisaurus Neuquenornis Intiornis Enantiornis_pectoral_wing_only
Elbretornis_holotype_only Martinavis_cruzyensis vincei saltariensis minor whetstonei Cratoavis Qiliania Feitianius
CAGS_IG_02_0901 CAGS_IG_04_CM_007 CAGS_IG_04_CM_023 Boluochia Longipteryx Shengjingornis
Shanweiniao Longirostravis Rapaxavis Halimornis Flexomornis Alexornis Xiangornis Eoalulavis Liaoningornis Vorona
Lectavis Hollanda Aberratiodontus Bellulornis Archaeorhynchus Patagopteryx PMO228.582 Hongshanornis
Archaeornithura Jianchangornis Schizooura Chaoyangia Changmaornis guozhangi Changzuiornis Yanornis_martini
Yixianornis Songlingornis Tianyuornis Parahongshanornis Longicrusavis Iteravis Gansus Ambiortus Yumenornis
Dingavis Juehuaornis Piscivoravis Mystiornis Ichthyornis Gargantuavis Potamornis Enaliornis Chupkaornis
Pasquiaornis Baptornis Bmongoliensis baileyi varneri Fumicollis Parahesperornis Hesperornis Xinghaiornis Horezmavis
Maaqwi Jiuquanornis Eogranivora Qinornis Iaceornis Palintropus Apsaravis Lithornis Palaeotis Limenavis Struthio
Tingmiatornis Chauna Polarornis Vegavis Anatalavis Presbyornis Teviornis Anas Gallinuloides Paraortygoides
Sylviornis Meleagris Cerebavis Foro Columba
	;
```

END;

BEGIN CHARACTERS;
TITLE 'Matrix in file "Hesperornithoides_NEXUS_matrix.nex"';
DIMENSIONS NCHAR=700;
FORMAT DATATYPE = STANDARD RESPECTCASE GAP = - MISSING = ? SYMBOLS = " 0 1 2 3 4 5 6 7 8 9 N";
CHARSTATELABELS
1 'Primary and secondary remiges - width of leading vane ' / longer_or_subequal_to_trailing_vane__ shorter_than_trailing_vane_,
2 'External naris - posterior extent with maxilla ventral border horizontal ' / ends_anterior_to_antorbital_fossa__ extends_posterior_to_anterior_edge_of_antorbital_fossa_,
3 'Nasal - dorsolateral surface posterior to external naris ' / solid_ pneumatized_via_fossae,
4 'Maxilla - lateral surface of antorbital fossa anterior to antorbital fenestra ' / solid_ 'with small maxillary fenestra entering maxillary antrum, greatest diameter <15% of orbit+jugal height ' 'with large maxillary fenestra 15-27% of orbit+jugal height' 'with large maxillary fenestra >27% of orbit+jugal height',
5 'Maxilla - lateral surface at ventral margin of antorbital fossa ' / fully_visible_laterally_ external_surface_projects_dorsally_to_form_a_lip_that_overlaps_ventral_edge_of_antorbital_fossa_,
6 'Maxilla - anteroposterior position of maxillary fenestra ' / 'close to anterior edge of antorbital fossa (distance between them <11% of orbit+jugal height)' 'far from anterior edge of antorbital fossa (>10%)',
7 'Maxilla - lateral surface at anterior margin of antorbital fossa ' / solid_ with_promaxillary_fenestra_entering_promaxillary_recess,
8 'Orbit - length compared to height ' / '>66% ' '<67%',
9 'Postorbital - shape of anterior margin of ventral process ' / with_projecting_orbital_process_ smoothly_curved_or_straight_,
10 'Jugal and postorbital - convexity of posterior edge of postorbital bar ' / 'concave to minimally convex (0-5% of orbit+jugal height) ' 'strongly convex (>5%)',
11 'Suborbital fenestra - size ' / 'greatest diameter >24% orbital height ' '<25%',
12 'Braincase - separation of anterior tympanic and subotic recesses ' / separated_by_a_surface_which_is_not_bounded_by_walls_and_depressed__ both_within_a_common_lateral_depression_bounded_on_all_sides,
13 'Basisphenoid - number of paired large foramina in basisphenoid recess ' / none_ at_least_one_pair_separated_at_midline_,
14 'Opisthotic - lateral extent of interfenestral crest ' / confluent_with_lateral_surface_ depressed_from_lateral_surface,
15 'Exoccipital - convexity of posterior surface containing vagus (X), accessory (XI) and hypoglossal (XII) nerve foramina' / flat_to_convex 'concave, forming paracondylar recess',
16 'Basisphenoid - pneumatic invasion of dorsolateral surface of basipterygoid process ' / flat_ excavated_by_an_extension_of_the_anterior_tympanic_recess_,
17 'Basisphenoid - pneumatic invasion of surface ventral to otic recess ' / 'excavated by subotic recess, an extension of the anterior temporal recess ' flat,
18 'Basioccipital and basisphenoid - position of basisphenoid recess ' / between_basisphenoid_and_basioccipital_ entirely_within_basisphenoid_,
19 'Basisphenoid - pneumatic invasion of ventral surface ' / pneumatic_median_recess_present_ flat_to_convex,
20 'Parasphenoid - transverse expansion of base of cultriform process ' / 'absent, process rodlike or vertically platelike ' present,
21 'Basisphenoid - transverse angle of basipterygoid process ' / less_than_51_degrees_laterally_from_vertical__ '>50 degrees',
22 'Basisphenoid - length of basipterygoid process ' / '>7% of median occipital condyle height ' '<8% but present' absent,
23 'Basisphenoid - pneumatic invasion of interior of basipterygoid process ' / 'absent, process solid ' 'present, process hollow',
24 'Prootic - pneumatic invasion of lateral surface ' / flat_ excavated_by_shallow_depression_for_dorsal_tympanic_recess excavated_by_posterolaterally_directed_cavity_for_dorsal_tympanic_recess,
25 'Opisthotic - pneumatic invasion of surface dorsal to interfenestral crest ' / flat_ excavated_by_extension_of_posterior_tympanic_recess_,
26 'Opisthotic - pneumatic invasion of lateral surface of paroccipital process or inside otic recess ' / flat_ excavated_by_main_diverticulum_of_posterior_tympanic_recess_,
27 'Opisthotic - position of main entrance of posterior tympanic recess ' / on_anterior_surface_of_paroccipital_process_ opens_into_otic_recess,
28 'Premaxilla - length of subnarial process' / does_not_contact_nasal__ 'contacts nasal, but does not extend posterior to external naris' extends_posterior_to_external_naris_,
29 'Premaxillae - cross section of combined dorsal process ' / transversely_compressed_or_not_compressed__ dorsoventrally_compressed,
30 'Premaxillae - angle of symphysis in dorsal or ventral view ' / 'low, so that snout is pointed ' 'high, so that snout is rounded ',
31 'Premaxilla - shape of ventral edge ' / smooth_ crenulated,
32 'Maxilla - posterior extent of palatal shelf' / 'anterior edge of choana placed <18% down maxillary length ' '>17%',
33 'Maxilla- ventral projection of posteromedial portion of palatal shelf ' / absent_ 'present, forming ''tooth-like'' process ',
34 'Jugal - length of dorsal process ' / '>34% of orbit+jugal height, measured from lower edge of orbit' '<35% ',

35 'Postorbital - length of ventral process ' / 'height of orbit taken up by ventral process / orbital height <80% ' '>79%',

36 'Jugal - cross section of posterior process ' / transversely_compressed__ dorsoventrally_or_not_compressed,

37 'Jugal - pneumatization of anterodorsal surface near antorbital fossa ' / 'present, surface invaded by pneumatic diverticulum of antorbital sinus ' 'absent, surface solid',

38 'Jugal - medial surface below postorbital process ' / penetrated_by_foramen_ solid,

39 'Quadratojugal - length of posterior process (defined as area posterior to intersection of ventral edge with line drawn along posterior edge of dorsal process)' / '<36% of length of rest of quadratojugal ' '>35%',

40 'Quadratojugal - contact with jugal ' / sutured_ fused,

41 'Lacrimal - projection of dorsal surface ' / unprojected_ 'with lateral ridge or boss projecting dorsally, forming lacrimal horn ',

42 'Lacrimal - lateral projection at dorsal edge ' / unprojected_or_minimally_projected__ strongly_projected_,

43 'Lacrimal - surface at posterodorsal corner of antorbital fossa ' / solid_ pneumatized_via_foramen,

44 'Lacrimal - length of posterior process measured from anterior orbital edge' / '<15% of ventral process length, measured from internal corner' '15-30%' '>30%',

45 'Lacrimal - length of anterior process measured from internal corner' / '<131% of ventral process length, measured from internal corner ' '>130% ',

46 'Prefrontal - size ' / 'large, greatest length >34% of orbit+jugal height ' 'small, <35% of orbit+jugal height ' absent_or_fused_to_another_element,

47 'Nasals and frontals - angle of sutures from median to lateral edge of contact ' / 'anteriorly convex <60 degrees ' anteriorly_convex_60_degrees_or_more_to_posteriorly_convex,

48 'Frontal - convexity of supratemporal fossa edge ' / completely_posteriorly_concave_to_straight_ with_posteriorly_convex_portion_,

49 'Frontal - shape of orbital margin ' / straight_to_smoothly_curved__ abruptly_angled_at_postorbital_process,

50 'Frontal - shape of anterior edge of nasolacrimal articular surface ' / smooth_ notched_anterolaterally_,

51 'Postorbital - curvature of anterior process ' / decurved_to_straight__ upcurved,

52 'Parietals - median proximity of supratemporal fossae ' / 'far, leaving flat dorsal surface between them ' 'close, separated by ridge forming sagittal crest ',

53 'Parietals - median contact ' / sutured_ fused,

54 'Squamosal - angle of ventral process ' / '<60 degrees from posterior quadrate edge ' '>59 degrees',

55 'Squamosal - lateral surface ' / smooth_ 'horizontal ridge over ventral process, extends to posterior process ',

56 'Quadratojugal - extent of squamosal contact ' / contacting_ 'non-contacting',

57 'Quadrate - divergence of otic articular surface ' / 'if present, articular surface confluent with shaft and squamosal head ' distinct_otic_head_caused_by_intercotylar_incisure,

58 'Quadrate - orientation of posterior edge compared to ventral margin of jugal + quadratojugal' / 'inside angle <106 degrees ' '>105 degrees ',

59 'Quadrate - width from the paraquadrate foramen medial edge to the lateral edge of the dorsal quadrate ' / '<30% of width across quadrate condyles ' '>29%',

60 'Quadrate - pneumaticity ' / solid_ invaded_by_diverticulum_of_the_tympanic_airsac_,

61 'Foramen magnum - dorsoventral elongation ' / transversely_elongate_or_height_and_width_equal_ height_greater_than_width_,

62 'Exoccipital and basioccipital - lateral margin of occipital condyle ' / posteromedially_sloped_ constricted_forming_condylar_neck_,

63 'Exoccipital - length of paroccipital process ' / 'height halfway out from lateral edge of occipital condyle / length from lateral edge of occipital condyle to tip of process <71% ' '>70%',

64 'Exoccipital - orientation of paroccipital process ' / 'long axis angled ventrally <41 degrees from horizontal ' '>40 degrees',

65 'Exoccipital - torsion of paroccipital process ' / untwisted_ twisted_so_that_distal_ends_face_posterodorsally,

66 'Ectopterygoid - ventromedial surface ' / flat_ excavated_by_a_pneumatic_fossa_,

67 'Ectopterygoid - dorsal surface ' / flat_ excavated_by_pneumatic_fossa_,

68 'Pterygoid - size of posterolateral flange anterior to basipterygoid articulation' / projects_ventrolaterally_from_element unprojected,

69 'Palatine - development of jugal process ' / projected_to_form_posterior_concavity_between_it_and_main_palatine_body__ unprojected,

70 'Ectopterygoid - palatine contact ' / absent_ present,

71 'Dentary - medial curvature of symphyseal region ' / none_ low high,

72 'Surangular - shape of dorsal margin ' / smooth_or_with_small_coronoid_eminence__ with_prominent_coronoid_eminence__,

73 'Dentary - amount of anterior taper ' / 'anterior depth at symphysis <60% of maximum posterior depth' '>59% ',

74 'Dentary - curvature in lateral view' / upcurved straight_ downcurved,

75 'Dentary - lateral surface of posterior dentary ' / roughly_flat__ with_horizontal_shelf_,

76 'Dentary - length of posterodorsal process ' / absent_ '<8% mandibular length, measured parallel to long axis of mandible ' '>8% mandibular length',

77 'Dentary - position of lateral nutrient foramina ' / on_flat_surface__ inside_longitudinal_groove,

78 'Surangular - shape of external mandibular fenestra border ' / straight_to_evenly_convex with_distinct_process_,

79 'Prearticular - concavity of anteroventral margin ' / convex_to_straight concave,

80 'Surangular - size of posterior foramen ' / absent_ small_foramen enlarged_fenestra,
81 'Splenial - lateral exposure ' / absent_or_narrow__ broad,
82 'Coronoid - size' / large_ small absent,
83 'Articular - dorsal surface of medial retroarticular process ' / on_level_with_rest_of_element_ projected_dorsally_into_distinct_process_,
84 'Articular - retroarticular process elongation ' / taller_than_long_to_equidimensional_ longer_than_tall_,
85 'Articular - dorsal surface of glenoid ' / concave_to_flat_in_lateral_view__ convex,
86 'Premaxilla - presence of teeth ' / toothed_ toothless,
87 'Premaxilla - size of second premaxillary tooth compared to third and fourth premaxillary teeth ' / smaller_to_equal_in_size_ much_larger_,
88 'Maxilla - presence of teeth ' / toothed_ toothless,
89 'Lateral teeth - serration extent ' / present_on_mesial_and_distal_carinae__ absent_on_mesial_carinae_but_present_on_distal_carinae_ absent_on_mesial_and_distal_carinae_,
90 'Dentary - number of teeth ' / '1-11' '12-24' '25-30' '>30',
91 'Lateral teeth - largest crown height ' / '>15% of orbit + jugal height ' '5-15%' '<5%',
92 'Teeth - serration size ' / '<26 on distal carina ' '>25',
93 'Teeth - distal serration shape ' / symmetrically_rectangular_to_rounded__ hooked_apically,
94 'Teeth - crown root transition ' / 'constricted mesial and/or distal edges ' undifferentiated_mesial_and_distal_edges_,
95 'Dentary teeth - size of anterior teeth ' / smaller_than_posterior_two_thirds_of_teeth 'similar to posterior third of teeth, leaving teeth in the middle third the largest' larger_than_posterior_two_thirds_of_teeth,
96 'Dentary teeth - difference in spacing between anterior and posterior teeth ' / anterior_more_widely_spaced_ subequal_spacing_ anterior_more_closely_spaced_,
97 'Premaxilla, maxilla and dentary - medial bone between teeth ' / 'undifferentiated from interdental septa and/or main body of element ' forms_distinct_interdental_plates_,
98 'Dorsal vertebrae - separation of postzygapophyses ' / joined_into_median_lamina__ separated_over_neural_canal_,
99 'Premaxillary teeth - convexity of lingual surface ' / subequally_or_more_convex_compared_to_labial_surface much_flatter_to_concave,
100 'Cervical vertebrae - number ' / ten_or_less_ eleven_or_more_,
101 'Cervical vertebrae - posterior extent of epipophyses on axis ' / does_not_extend_past_postzygapophyses_ extend_past_postzygapophyses_,
102 'Axis - development of spinopostzygapophyseal lamina' / 'deep, broadly connecting neural spine with postzygapophyses ' 'narrow to absent, so that neural spine and postzygapophyses are separated',
103 'Cervical vertebrae - anteroposterior position of epipophyses ' / partly_over_postzygapophyseal_facets anterior_to_postzygapophyseal_facets,
104 'Cervical vertebrae - posterior extent of centrum on cervicals 3-4 ' / completely_under_neural_arch_ extending_posteriorly_past_neural_arch,
105 'Cervical vertebrae - anteroventral surface of cervicals 9-10 ' / convex_or_flat__ transversely_concave_due_to_carotid_processes_,
106 'Cervical vertebrae - anterior central articular surface dimensions of cervicals 3-4 ' / less_than_1.71_times_wider_than_tall__ over_1.70_times_wider_than_tall_,
107 'Cervical vertebrae - neural spine anteroposterior length of cervicals 3-5 ' / 'length / neural arch length excluding epipophyses >29% ' '<30%',
108 'Cervical vertebrae - number of pneumatic foramina in each centrum ' / one_pair_anteriorly_located_behind_parapophyses_ two_pairs,
109 'Cervical vertebrae - convexity of anterior articular surface ' / flat_or_concave_ convex 'convex dorsoventrally, concave horizontally ',
110 'Dorsal vertebrae - convexity of anterior articular surface of centra 1-3' / flat_or_concave_ convex 'convex dorsoventrally, concave horizontally ',
111 'Dorsal vertebrae - hypapophysis size on dorsals 1-3 ' / hypapophyses_absent_or_all_less_than_25%_of_posterior_central_articular_surface_height_ at_least_one_hypapophysis_more_than_25%_of_posterior_central_articular_surface_height,
112 'Dorsal vertebrae - lateral projection of parapophyses on dorsals 6-13 ' / 'parapophysis width measured from ventromedial corner <20% of centrum width ' '>19%',
113 'Dorsal vertebrae - orientation of medial postzygapophyseal edge - undifferentiated from postzygapophyseal facet ' / undifferentiated_from_postzygapophyseal_facet__ bent_ventrally_to_form_hyposphene_which_articulates_with_hypantrum_on_previous_vertebra_,
114 'Dorsal vertebrae - extent of central pleurocoels in series' / absent_ only_present_anteriorly present_in_anterior_and_posterior_dorsals_,
115 'Dorsal vertebrae - lateral extent of transverse processes on dorsals 1-4 ' / project_far_beyond_prezygapophyses_laterally_ 'project slightly past prezygapophyses laterally (distance from midline to transverse process tip / distance from midline to prezygapophysis tip <180% for d1, <200% for d2, <215% for d3 and <250% for d4)',
116 'Dorsal vertebrae - transverse width of neural spine distal tip ' / '<151% of minimum neural spine width ' '>150%',
117 'Sacral vertebrae - number' / less_than_6_ 6 7 8 9 10 '11-14' '>14',
118 'Sacral vertebrae - ventral surface of posterior centra ' / transversely_rounded__ transversely_flattened_with_median_groove_on_some_ with_median_keel_on_some_,
119 'Sacral vertebrae - number of vertebrae with central pneumatic foramina' / none_ at_least_one_vertebra_but_not_present_in_all_ all,
120 'Sacral vertebrae - convexity of posterior articular surface of last sacral centrum ' / concave_to_flat__ convex,

121 'Sacral vertebrae - dorsal connection of zygapophyses ' / each_zygapophyseal_articulation_distinct_from_that_of_adjacent_vertebrae_ zygapophyseal_articulations_joined_to_form_ridge,
122 'Caudal vertebrae - number with transverse processes of any size ' / eleven_or_more__ seven_to_ten_ less_than_seven_,
123 'Caudal vertebrae - transverse section of proximal centra ' / rounded_ rectangular,
124 'Caudal vertebrae - ventral surface of centra 1-2' / 'convexly rounded, flat or grooved ' with_a_median_keel_,
125 'Caudal vertebrae - dorsal outline of neural spines in caudals ~5-30 ' / convex_or_flat__ concave_to_form_accessory_anterior_spine_,
126 'Caudal vertebrae - transverse convexity of dorsal midline in distal caudals ' / narrowly_convex_to_form_neural_spine__ flat_with_no_spine_or_groove_ concave_to_form_median_groove,
127 'Caudal vertebrae - length of longest prezygapophyses in distal caudals ' / '<33% of centrum length ' '33-100% of centrum length ' '>100% of centrum length ',
128 'Caudal vertebrae - number ' / '>40' '25-40' '21-24' '<21',
129 'Chevrons - anteroposterior length compared to dorsoventral depth ' / '<76% through chevron 10 ' '>75% by chevron 10 ',
130 'Chevrons - transverse convexity of anterior ventral edge in distal elements ' / convex_to_straight_ concave_to_form_bifurcated_anterior_ventral_process_,
131 'Cervical ribs - length ' / greater_than_centrum__ equal_or_less_than_centrum_,
132 'Uncinate processes - ossification ' / unossified_or_absent__ ossified,
133 'Sternal ribs - ossification ' / unossified_or_absent__ ossified,
134 'Gastralia - length of lateral segments ' / shorter_than_medial_segment__ longer,
135 'Sternum - fusion of sternal plates and ventral projection of median surface ' / 'sternal plates unfused, without ventral keel ' 'sternal plates fused, without ventral keel ' 'sternal plates fused, with low ventral keel' 'sternal plates fused, with tall ventral keel ',
136 'Sternum - shape of lateral edge posterior to rib articulations ' / evenly_rounded_or_straight__ distinct_posterolateral_process_present_,
137 'Sternum - surface of coracoid articulation ' / concave_dorsoventrally_forming_groove__ convex,
138 'Furcula - size of hypocleideum ' / absent_ small_ridge_or_tubercle_ large_process_,
139 'Scapula - eversion of dorsal edge of acromion ' / 'absent, acromion completely dorsally to medially projected ' 'present, edge of acromion laterally projected ',
140 'Humerus - length ' / less_or_equal_to_scapula__ greater_than_scapula_,
141 'Coracoid - transverse convexity of posterior edge (anteroposterior convexity of lateral edge in Aves)' / convex_to_flat__ concave_forming_subglenoid_fossa_,
142 'Scapulocoracoid - form of contact between components' / sutured_or_freely_articulated fused,
143 'Coracoid - proximodistal elongation ' / 'proximodistal depth <91% of anteroposterior length from anterior edge to glenoid (mediolateral length from medial edge to glenoid in Aves)' '91-200%' '>200% ',
144 'Coracoid - angle formed in posterior (lateral in Aves) view at level of coracoid tubercle ' / '<31 degrees' '>30 degrees ',
145 'Scapula - orientation of glenoid ' / mostly_on_ventral_edge__ wraps_onto_lateral_surface,
146 'Humerus - angle between distal deltopectoral crest edge at apex and posterior edge of shaft at that point ' / '>85 degrees ' '46-85 degrees' '<46 degrees',
147 'Humerus - proximodistal placement of deltopectoral crest apex ' / '<30% of element length' '30-50%' '>50%',
148 'Humerus - depth of deltopectoral crest ' / 'width of humerus at deepest part of deltopectoral crest <301% minimal shaft depth ' '>300%',
149 'Humerus - lateral surface of distal deltopectoral crest ' / flat_ with_longitudinal_ridge,
150 'Ulna - length of olecranon process ' / 'proximal tip to apex of coronoid process compared to length of element <15%' '15-25%' '>25%',
151 'Ulna - transverse convexity of distal end ' / rather_straight_to_concave highly_convex,
152 'Ulna - separation of proximal cotylae' / single_cotyla separate_cotylae,
153 'Manual phalanx I-1 - shape of proximoventral edge ' / convex_to_flat_ concave_due_to_flexor_tubera,
154 'Distal carpals I and II - form of contact' / unfused_ fused,
155 'Distal carpal I - form of contact with metacarpal I ' / unfused fused,
156 'Distal carpal II - form of contact with metacarpal II' / unfused_ fused,
157 'Distal carpals I and II - combined transverse width' / 'covers >74% of proximal transverse width of metacarpals I and II ' '<75%',
158 'Metacarpal I - length compared to metacarpal II ' / '<50% ' '50-65%' '66-80%' '>80%',
159 'Manual digit III - number of phalanges ' / four_ three two one none,
160 'Manual unguals - proximodistal position of flexor tubercle apex' / '<25% down length of ungual (measured on the ventral curve, excluding the tubercle) ' '>24%',
161 'Manual ungual I - curvature, measured as maximum distance between ventral edge and line drawn between proximodorsal and distoventral tips divided by length of latter line ' / '>16% ' '10-16%' '<10%',
162 'Manual ungual II - curvature, measured as maximum distance between ventral edge and line drawn between proximodorsal and distoventral tips divided by length of latter line ' / '>16% ' '10-16%' '<10%',
163 'Manual ungual III - curvature, measured as maximum distance between ventral edge and line drawn between proximodorsal and distoventral tips divided by length of latter line ' / '>16% ' '10-16%' '<10%',
164 'Manual unguals - depth of flexor tubercles ' / '>32% of articular facet height ' '<33%',
165 'Manual ungual I - size compared to manual ungual II ' / smaller_ equal_to_three_times_larger_ over_three_times_larger,
166 'Manual ungual I - shape of proximodorsal margin ' / smoothly_convex_ with_lip_rising_dorsally,

167 'Manual ungual II - shape of proximodorsal margin ' / smoothly_convex__ with_lip_rising_dorsally_,

168 'Manual ungual III - shape of proximodorsal margin ' / smoothly_convex__ with_lip_rising_dorsally_,

169 'Ilium - ventral expansion of preacetabular process ' / 'expansion (measured as distance preacetabular tip extends ventrally past highest point at ventral edge of preacetabular process / height of preacetabular process at that point) >18% ' '<19% but present' absent,

170 'Ilium - length of preacetabular process, measured from notch below process ' / '<50% of postacetabular length, measured from posterior edge of ischial peduncle base' '50-120%' '>120%',

171 'Ilium - angle of anterior edge of preacetabular process ' / less_than_11_degrees_posteroventrally_ '10-70 degrees posteroventrally ' 'more than 70 degrees posteroventrally, often being the anteroventral edge of a round process ',

172 'Ilium - dorsal margin of acetabulum in dorsal/ventral view ' / markedly_convex_due_to_well_developed_supracetabular_crest_ slightly_convex_due_to_poorly_developed_supracetabular_crest straight_or_concave_due_to_absence_of_supracetabular_crest_,

173 'Ilium - lateral surface at dorsal edge posterodorsal to acetabulum ' / flat_ developed_into_supratrochanteric_process_,

174 'Ilium - shape of postacetabular process ' / quadrangular_posteriorly__ triangular_posteriorly,

175 'Ilium - dorsal postacetabular edge angled posterolaterally' / '<11 degrees between right and left ilia ' '>10 degrees',

176 'Ilium - depth of brevis fossa ' / deep_ shallow absent,

177 'Ilium - lateral projection of antitrochanter ' / undifferentiated_from_supracetabular_area_and_postacetabular_process_ projected_further_laterally_,

178 'Ilium - posterior extent of m. cuppedicus fossa over pubic peduncle in lateral view ' / over_at_least_half_of_peduncle__ over_less_than_half_,

179 'Ilium - ventral surface on preacetabular process for m. puboischiofemoralis internus (iliofemoralis internus and iliotrochantericus cranialis in Aves)' / forms_cuppedicus_fossa_which_is_overhung_laterally__ forms_flat_surface_or_laterally_angled_fossa_which_is_not_overhung_laterally muscles_attach_to_undifferentiated_blade_of_ilium_,

180 'Ischium - proximodorsal edge ' / unprojected_ projected_into_low_process_separated_from_ilial_peduncle_by_shallow_concavity projected_into_tall_process_and_separated_from_ilial_peduncle_by_notch,

181 'Ischium - distal curvature ' / curved_dorsally straight curved_ventrally,

182 'Ischium - dorsoventral convexity of lateral surface ' / concave flat_or_convexly_rounded ridged,

183 'Ischium - proximodistal position of obturator process apex ' / '<34% down shaft' '34-66%' '>66%',

184 'Ischium - contact between obturator process and pubis ' / absent_ present,

185 'Ischium - development of ischial apron' / notch_proximally_between_obturator_process_and_pubic_peduncle_ notch_enclosed_fully_forming_obturator_foramen_ no_ventral_projection_or_foramen_in_ischium_ventral_margin,

186 'Ischium - ventral edge of pubic peduncle ' / flat projected_as_obturator_tuber_,

187 'Ischium - length compared to pubis ' / '>100% ' '66-100%' '50-65%' '<50%',

188 'Ischium - form of median contact ' / fused_ unfused_but_contacting_ no_contact,

189 'Ischium - dorsoventral expansion of distal end ' / wider_than_shaft_ narrower_or_equal_to_shaft,

190 'Pubis - orientation of proximal half in lateral view ' / '<67 degrees from anteriorly horizontal' '67-120 degrees' '121-150 degrees' '>150 degrees',

191 'Pubis - anteroposterior length of anterior boot, measured as flare from prior curvature of pubis' / '>3% of pubic length ' '<4%',

192 'Pubis - anteroposterior length of posterior boot, measured as distal depth of pubis excluding anterior boot ' / '>29% of pubic length ' '15-29%' '6-14%' '<6%',

193 'Pubis - anteroposterior position of apron ' / in_middle_or_at_posterior_edge_of_shaft__ at_anterior_edge_of_shaft,

194 'Pubis - curvature in lateral view ' / anteriorly_concave_ straight_or_sigmoid posteriorly_concave,

195 'Pubis - proximal extent of symphysis, measured to median edge ' / '>41% of pubic length ' '<42% but present' absent,

196 'Femur - medial surface of head ' / flat_to_convex__ concave_to_form_capital_ligament_fossa,

197 'Femur - separation of anterior and greater trochanters ' / 'depth of the intertrochanteric notch (from the tip of the anterior trochanter) >65% the minimum anteroposterior diameter of the femur ' '<66% but still present ' 'absent, trochanters fused forming trochanteric crest',

198 'Femur - section of anterior trochanter in proximal view ' / 'anteroposteriorly elongate, forming aliform trochanter ' equal_to_transversely_elongate_,

199 'Femur - posterolateral surface at proximal end ' / flat_ 'posterior trochanter for insertion of m. ischiotrochantericus (m. ischiofemoralis in Aves) present but separate from more anterior m. iliofemoralis externus insertion (trochanteric shelf homolog)' posterior_trochanter_present_and_congruent_with_trochanteric_shelf,

200 'Femur - posterior surface of midshaft ' / 'with convexity for insertion of M. caudofemoralis longus, forming fourth trochanter ' flat_or_depressed_,

201 'Femur - convexity of anterior edge of anterior trochanter in lateral view ' / convex_or_straight__ 'concave, forming accessory trochanter distal to it ',

202 'Femur - anteromedial surface of distal end ' / uniformly_convex_transversely with_proximodistal_ridge_forming_medial_epicondyle,

203 'Femur - distal enclosure of popliteal fossa between distal condyles ' / distally_open__ distally_closed,
204 'Fibula - contact with proximal tarsals ' / contact_present_ contact_absent,
205 'Fibula - concavity of proximomedial surface ' / flat_to_convex_ shallow_concavity_ deep_concavity_,
206 'Proximal tarsals - depth of concavity between medial and lateral condyles in distal view' / '<20% of anteroposterior depth of deepest condyle ' '>19%',
207 'Tibia - anteromedial surface of proximal portion ' / flat_ with_proximodistal_ridge_forming_medial_cnemial_crest_,
208 'Astragalus - height of ascending process, from distal tibiotarsal edge to ascending process apex' / '>84% of astragalocalcanear width ' '<85%',
209 'Astragalus - mediolateral width of ascending process ' / 'measured halfway up, <58% width of astragalocalcaneum ' '>57%',
210 'Astragalus - concavity of medial edge of ascending process ' / convex_to_slightly_concave 'markedly concave, at least comparable in depth to Shuvuuia and Mononykus',
211 'Astragalus - confluence of condyles and ascending process ' / confluent_ separated_by_groove,
212 'Astragalus - convexity of anterior surface at base of ascending process ' / flat_compared_to_rest_of_ascending_process distinctly_depressed_as_a_fossa_,
213 'Astragalus - form of contact with tibia ' / unfused_ fused,
214 'Astragalus - form of contact with calcaneum ' / unfused fused,
215 'Distal tarsals - form of contact with metatarsals ' / unfused_ fused,
216 'Metatarsals II-IV - extent of fusion ' / unfused_ 'at least two fused proximally, but not distally' 'fused proximally and distally, but not distal to distal vascular foramen between III and IV ' fusion_encloses_distal_vascular_foramen,
217 'Metatarsal II - depth of distal articular groove ' / absent_or_shallow__ deep,
218 'Metatarsal III - depth of distal articular groove ' / absent_or_shallow__ deep,
219 'Metatarsal III - proximal transverse constriction ' / anterior_exposure_wider_or_subequal_to_metatarsals_II_and_IV_ 'anterior exposure significantly narrower than II and IV but still present at proximal end, subarctometatarsal ' 'hidden anteriorly at proximal end by contact between II and IV but still exposed on proximal surface, arctometatarsal ' 'does not extend to proximal surface, hyperarctometatarsal ',
220 'Metatarsal IV - transverse width at midshaft ' / '<117% of depth at midshaft ' '>116% of depth',
221 'Metatarsal I - presence ' / present_ absent,
222 'Metatarsal I - proximodistal position' / 'distance between distal ends of metatarsals I and II when articulated >30% length of metatarsal II ' '18-30%' '6-18%' '<6%',
223 'Metatarsal I - proximal extent ' / ends_distal_to_tarsus__ extends_to_contact_tarsus_,
224 'Pedal ungual II - length on straight line compared to ungual III ' / '<141% ' '>140%',
225 'Pedal ungual II - curvature' / 'depth of the dorsal arc <30% of ungual length (measured in a straight line from the proximodorsal tip to the distal tip) ' '>29%',
226 'Metatarsal IV - transverse width at midshaft compared to metatarsal II' / '<66%' '66-165%' '>166%',
227 'Skull - depth of snout ' / 'height of preorbital snout one fourth from tip >39% of orbit+jugal height ' '<40%',
228 'Premaxilla - angle between anterior subnarial and ventral margins ' / '>34 degrees ' '<35 degrees ',
229 'Premaxilla - subnarial height compared to alveolar length ' / '>180%' '101-180%' '50-100%' '<50',
230 'Premaxilla and maxilla - space between toothrows of each element ' / similar_to_that_between_teeth_in_each_element_ 'greater, forming diastema between elements ',
231 'Maxilla- shape of promaxillary fenestra ' / 'minimum diameter >35% of maximum diameter ' '<36%',
232 'Nasal - convexity of dorsal edge in lateral view' / convex_or_straight__ concave,
233 'External naris - length ' / 'longest axis <49% of orbit+jugal height ' '49-80%' '>80%',
234 'Postorbital - length of posterior process ' / shorter_or_equal_to_ventral_process__ longer,
235 'Frontal - dorsal surface of supratemporal fossa ' / flat_ with_pit,
236 'Parietal - minimum width ' / less_or_equal_to_minimum_frontal_transverse_width__ more,
237 'Parietal - dorsal outline ' / concave_to_straight convex,
238 'Quadrate - proximodistal position of pterygoid process apex ' / '>45%' '<46% ',
239 'Basioccipital - shape of notch between basal tubera ' / rounded_ angled,
240 'Laterosphenoid - lateral surface dorsal to exit for oculomotor (III) nerve' / flat_to_convex excavated_by_fossa_,
241 'Dentary - posterior depth compared to length ' / '<15% ' '15-50%' '>50%',
242 'Teeth - DSDI' / '<1.19 ' '>1.2',
243 'Dorsal vertebrae - length of column ' / '>119% of femoral length ' '<120%',
244 'Dorsal vertebrae - centrum proportions of dorsals 6-13 ' / 'length <83% of posterior height ' '83-130%' '130-200%' '>200%',
245 'Dorsal vertebrae - anteroposterior expansion of neural spine apex in dorsals 6-13 ' / '<116% of minimum anteroposterior spine length ' '>115%',
246 'Sacral vertebrae - transverse width of ancestral neotheropod sacral centra 1 and 2 ' / '>69% of ancestral sacral centrum 3 width ' '<70%',
247 'Caudal vertebrae - distal anteroposterior width of transverse processes on caudals 1-4 ' / tapered_ subequal expanded,
248 'Caudal vertebrae - length of caudals 10-15 ' / shorter_than_caudal_1 '100-200% longer' '>200% longer',
249 'Sternal ribs - number which contact dorsal ribs ' / none_to_three__ at_least_four,

250 'Furcula - size ' / 'length of arm from omal tip to base excluding hypocleidium <36% of scapular length ' '>35%',

251 'Coracoid - surface just distal to coracoid tuber ' / solid_ pierced_by_supracoracoid_fenestra,

252 'Scapula - contact between acromion and coracoid ' / present_to_near_anterior_tip__ 'absent, scapula-coracoid contact limited to short area near glenoid ',

253 'Scapulocoracoid - length of glenoid edge formed by scapula, with glenoid edge of each bone simplified to a straight line ' / '<160% the length of glenoid edge formed by coracoid ' '>159%',

254 'Humerus - length compared to femur ' / '<38% ' '38-69%' '70-100%' '>100%',

255 'Femur - distal extent of lateral condyle past medial condyle ' / '<11% of distal femur width' '>10% of distal femur width ',

256 'Radius - midshaft anteroposterior diameter ' / '>71% of ulnar midshaft anteroposterior diameter ' '51-71%' '<51%',

257 'Manual phalanx I-1 - length compared to metacarpal II ' / '<70%' '70-100%' '>100%',

258 'Manual phalanx III-2 - length compared to III-1 ' / '>105%' '60-105%' '<60%',

259 'Manual phalanx III-2 - proximoventral heel, prominence measured as angle between dorsal and ventral edges of element ' / '<11 degrees ' '>10 degrees',

260 'Manual phalanx III-3 - dorsoventral depth of shaft just proximal to distal condyles ' / similar_to_rest_of_shaft__ much_shallower_than_rest_of_shaft_,

261 'Ilium - length ' / '>63% of femoral length ' '<64%',

262 'Ilium - anteroposterior diameter of external acetabulum (measured between lateral surfaces of peduncles, excluding any medial walls or angled anterior and posterior acetabular surfaces)' / '>18% of ilial length ' '<19%',

263 'Ilium - angle main axis of pubic peduncle projects, compared to line drawn between bases of peduncles' / anterior_to_vertical_ posterior,

264 'Pubis - lateral surface of mid shaft ' / smooth_ with_lateral_projection_,

265 'Ischium - dorsal process at midshaft ' / absent_ present,

266 'Tibia - surface just posterior to fibular crest ' / flat_ longitudinally_grooved_,

267 'Pedal phalanx II-2 - length compared to II-1 ' / '<60% ' '60-82%' '83-100%' '>100%',

268 'Pedal phalanx II-2 - height of distal condyles compared to length (excluding posterior lips)' / '<61% ' '>60%',

269 'Metatarsal IV - convexity of posterior surface in section ' / flat_or_broadly_convex__ sharply_keeled,

270 'Pedal phalanx IV-4 - length compared to IV-3 ' / '<101% ' '>100%',

271 'Metatarsal V - length compared to metatarsal IV ' / '<41% ' '>40%',

272 'Pedal ungual I - depth of the dorsal arc compared to ungual length (measured in a straight line from the proximodorsal tip to the distal tip) ' / '<23% ' '>22%',

273 'Pedal unguals III and IV - depth of the dorsal arc compared to ungual length (measured in a straight line from the proximodorsal tip to the distal tip) ' / '<16%' '16-21%' '>21%',

274 'Pedal unguals - cross section of III and IV' / 'expanded ventrally, triangular ' subequal_in_transverse_width_dorsally_and_ventrally,

275 'Feathers - presence ' / 'absent, skin naked or scaled ' 'present, unbranched or branched filamentous integument',

276 'Feathers - arrangement of barbs ' / unbranched_or_with_basally_radiating_barbs_ with_barbs_arranged_in_a_vane_,

277 'Feathers - length of feathers along metatarsus ' / absent_to_comparable_to_body_feathers_ elongated,

278 'Pubis - angle of posterior pubic boot to shaft ' / '>79 degrees ' '<80 degrees',

279 'Chevrons - transverse convexity of posterior ventral edge in distal elements' / convex_to_straight__ concave_to_form_bifurcated_posterior_ventral_process_,

280 'Metacarpal I - proportions in dorsal/ventral view ' / longer_than_wide_ wider_than_long,

281 'Manual phalanx I-1 - minimum transverse width ' / less_than_or_equal_to_minimum_transverse_width_of_radius_ greater,

282 'Angular - posterior extent at ventral mandibular edge ' / 'extends to reach >94% down mandibular length from symphysis ' '<95%',

283 'Surangular - dorsolateral surface just anterior to glenoid ' / unprojected_ dorsally_projected_as_everted_ridge,

284 'Metacarpal I - convexity of distal end in dorsal view ' / deeply_concave_due_to_well_developed_ginglymus_ slightly_concave_ flat_to_convex,

285 'Metacarpal II - convexity of distal end in dorsal view ' / concave flat_to_convex,

286 'Dentary - convexity of anterodorsal edge ' / concave_to_roundly_convex__ with_abrupt_ventral_deflection,

287 'Quadrate - lateral exposure of head ' / covered_by_squamosal__ visible_laterally_,

288 'Ilium - ventral projection of medial brevis ridge ' / at_least_partly_ventral_to_lateral_brevis_fossa_edge__ reduced_to_not_be_visible_laterally_,

289 'Femur - development of insertion for M. iliofemoralis externus on proximolateral surface' / unprojected_to_low_mound__ forms_longitudinal_ridge_ forms_trochanteric_shelf,

290 'Dentary teeth - extent ' / over_most_of_alveolar_edge__ restricted_to_anterior_tip_ absent,

291 'Coracoid - depth of subglenoid notch ' / 'anteroposterior length from anterior coracoid edge to posterior tip of coracoid glenoid (mediolateral length from medial coracoid edge to lateral tip of coracoid glenoid in Aves) <106% of depth from anterior coracoid edge to subglenoid notch ' '>105%',

292 'Articular - curvature of retroarticular process ' / straight_or_decurved__ upcurved,

293 'Scapula - lateral surface dorsal to glenoid ' / flat_ with_dorsoventral_ridge_forming_supraglenoid_buttress,

294 'Basioccipital - transverse width across basal tubera ' / '>164% of occipital condyle transverse width ' '<165%',

295 'Metatarsal II - length' / '<98% of metatarsal III ' '>97%',

296 'Basioccipital - posterior surface ventrolateral to occipital condyle ' / flat_ excavated_by_subcondylar_recess,
297 'Humerus - transverse width of entepicondyle ' / narrow_or_absent_ 'wide, >15% of distal humeral width',
298 'Ilium - convexity of ischial peduncle articular surface ' / concave_to_flat__ 'convex, articulates with socket on ischium',
299 'Teeth - labiolingual compression of roots of lateral teeth ' / 'labiolingual width <70% FABL ' '>69%',
300 'Ilium - lateral deflection of preacetabular process ' / lateral_edge_of_preacetabular_process_less_than_60_degrees_from_dorsal_postacetabular_margin__ '60-90 degrees' more_than_90_degrees,
301 'Teeth - recurvature ' / significant_ slight_or_absent,
302 'Dentary and maxilla - method of tooth implantation ' / all_in_distinct_sockets__ some_in_confluent_groove,
303 'Pubis - extent of symphyseal contact of aprons ' / continuous interpubic_fenestra,
304 'Ilium - convexity of dorsal edge of postacetabular process ' / convex_or_straight_ concave,
305 'Ilium - posterolateral edge of postacetabular process in dorsal view ' / ventral_edge_does_not_project_laterally_compared_to_dorsal_edge_ ventral_edge_has_lateral_projection_at_posterior_end_of_ilium,
306 'Pedal phalanx II-2 - length of proximoventral heel compared to height of distal condyles ' / absent 'present but <44% ' '>43%',
307 'Ischium - obturator notch distal to process ' / present_ absent,
308 'Ischium - angle between edges of obturator process ' / '>29 degrees ' '<30 degrees',
309 'Metatarsals II and III - topology of dorsal surface of shaft ' / no_tubercle_developed_on_either_metatarsal_ tubercle_for_insertion_of_m._tibialis_cranialis_on_center_or_medial_edge_of_metatarsal_II tubercle_for_insertion_of_m._tibialis_cranialis_on_lateral_edge_of_metatarsal_II_or_on_metatarsal_III_,
310 'Maxilla - concavity of anterodorsal margin at base of dorsal process ' / 'depth of concavity <4% of orbit+jugal height ' '>3%',
311 'Maxilla - length of anterior ramus, measured from point of maximum anterodorsal concavity ' / '<150% of maxillary height at that point ' '>149%',
312 'Nasal - extent of antorbital fossa on lateral surface ' / absent_ present,
313 'External mandibular fenestra - length compared to mandibular length ' / '>18%' '12-18%' '1-11%' absent,
314 'Anterior teeth - texture of carinae ' / serrated_ unserrated,
315 'Maxilla - posterior extent of teeth ' / posterior_to_anterior_orbital_margin__ restricted_anterior_to_anterior_orbital_margin_but_extend_over_half_of_maxillary_length restricted_to_less_than_anterior_half_of_maxilla_,
316 'Metacarpal IV - presence' / present_ absent,
317 'Ilium - ventral extent of pubic peduncle compared to ischial peduncle ' / equal_or_less_ greater,
318 'Pubis - extent of puboischiadic plate proximally ' / obturator_foramen_pierces_plate__ obturator_notch_formed_when_foramen_opens_ventrally_ obturator_notch_reduced_so_that_posterior_edge_of_ischial_peduncle_does_not_project_distally,
319 'Femur - proximal extent of anterior trochanter ' / completely_distal_to_head__ extends_to_lower_half_of_head_ extends_at_least_to_upper_half_of_head_,
320 'Astragalus - anteroposterior depth of ascending process' / deep_and_blocky_ shallow_and_laminar,
321 'Maxilla - dorsoventral position of maxillary fenestra ' / 'distance from ventral antorbital fossa rim <11% of orbit+jugal height ' '>10%',
322 'Maxilla - depth of posterior process ' / 'minimal depth <16% orbit+jugal height ' '>15%',
323 'Maxilla - convexity of lateral surface surrounding maxillary fenestra ' / flat_ 'concave, forming fossa ',
324 'Maxilla - size of external portion of dorsal process' / robust_ reduced absent,
325 'Maxilla - height of antorbital fossa portion of dorsal process ' / broadly_contacts_external_portion_of_dorsal_process__ separated_from_external_portion_,
326 'Nasal - contact with antorbital fossa ' / absent_ present_but_does_not_contact_antorbital_fenestra present_and_contacts_antorbital_fenestra_,
327 'Premaxilla - contact with antorbital fossa ' / absent_ present,
328 'Jugal - contact with antorbital fenestra ' / absent_ present,
329 'Splenial - concavity of posterior margin ' / convex_to_slightly_concave__ strongly_concave,
330 'Premaxillary teeth - height of first tooth' / 'less than twice height of premaxillary teeth 2-4 ' 'more than twice height of premaxillary teeth 2-4 ',
331 'Postorbital - angle of anterior process ' / '<96 degrees from long axis of ventral process ' '>95 degrees',
332 'Jugal - lateral exposure of posterior process ' / much_of_process_exposed__ most_of_process_hidden_by_quadratojugal_,
333 'Frontal - shape of suture with nasal ' / roughly_straight_or_curved__ 'W-shaped',
334 'Cervical vertebrae - height of neural spines ' / taller_than_anteroposteriorly_long__ lower_than_anteroposteriorly_long_,
335 'Caudal vertebrae - fusion of distal caudals ' / absent_ 'present, fused into pygostyle',
336 'Coracoid - anterior projection of anterodistal margin (medial projection of mediodistal margin in Aves) ' / posterior_to_or_on_level_with_anterior_margin_of_scapulocoracoid_boundary_ 'anterior to anterior margin (medial to medial margin in Aves) of scapulocoracoid boundary ',
337 'Skull - length compared to femur ' / '>125% ' '80-125%' '61-79%' '<61%',

338 'External naris - dorsoventral placement' / 'distance between ventral edge and alveolar edge <51% of orbit+jugal height ' '>50%',

339 'Maxilla - posterior extent of medial wall of antorbital fossa ' / 'length of antorbital fossa anterior to antorbital fenestra / antorbital fenestra length <46% ' '>45%',

340 'Maxilla - elongation of maxillary fenestra ' / 'height >50% of length ' '25-50%' '<25%',

341 'Nasals - median contact ' / 'sutured or non-contacting ' fused,

342 'Nasal - surface texture ' / smooth_ rugose,

343 'Jugal - minimum suborbital height ' / '>30% of orbital height ' '20-30%' '<20%',

344 'Nasal - length compared to frontal ' / longer_or_equal_ shorter,

345 'Skull - ventral deflection of snout ' / 'angle between maxilla and jugal+quadratojugal ventral margins <30 degrees ' '>29 degrees',

346 'Maxilla - length of ventral margin ' / '>125% of orbit+jugal height ' '<126%',

347 'Frontal - level of lateral edge of dorsal surface ' / equal_or_depressed_compared_to_rest_of_frontal__ raised_rim,

348 'Parietal - length compared to frontal ' / shorter_ longer,

349 'Laterotemporal fenestra - greatest anteroposterior length ' / '>30% of orbit+jugal height ' '<31%',

350 'Exoccipital and basioccipital - transverse width of foramen magnum ' / '<101% of occipital condylar width ' '>100%',

351 'Dentary - depth of Meckelian groove ' / deep_ shallow,

352 'Dentary - posterior extent of ventral dentary ' / ends_prior_to_or_partly_overlapping_external_mandibular_fenestra__ extends_past_external_mandibular_fenestra_,

353 'Surangular - lateral surface ventral to glenoid ' / flat_ with_horizontal_ridge_,

354 'Premaxillary teeth - height of longest compared to longest maxillary tooth ' / '>70% ' '<71%',

355 'Dentary teeth - presence at tip of dentary ' / present_ absent_for_space_of_more_than_two_FABLs_from_dentary_tip,

356 'Cervical vertebrae - elongation' / centrum_length_less_than_centrum_height_ equal_to_twice_as_long length_more_than_twice_height_,

357 'Dorsal vertebrae - height of neural spines of posterior dorsals (6-13)' / '>149% taller than long' '>75% of posterior central height' '<76%',

358 'Dorsal vertebrae - posterior extent of postzygapophyses on mid and posterior dorsals (4-13) ' / end_above_centrum__ extend_posterior_to_centrum_,

359 'Chevrons - length of proximal chevrons compared to caudal centrum length ' / '>149% ' '<150%',

360 'Furcula - interclavicular angle, measured from center of median to tips of arms ' / '>69 degrees ' '<70 degrees',

361 'Scapula - angle of posterior edge of acromion ' / '>80 degrees compared to dorsal edge of shaft just proximal to it' '80-20 degrees' '<20 degrees',

362 'Scapula - angle of anterior edge of acromion ' / '<106 degrees compared to dorsal edge of shaft just posterior to it' '>105 degrees',

363 'Scapulocoracoid - shape of anterior edge at suture ' / notched smooth,

364 'Scapula - expansion of posterior end ' / '>184% of minimum shaft width ' '<185%' tapered,

365 'Coracoid - position of coracoid tuber ' / distal_to_scapular_articulation__ 'proximal to scapular articulation, forming acrocoracoid process',

366 'Humerus - minimum width in anterior view compared to length ' / '>12% ' '<13%',

367 'Ulna - shape of posterior margin ' / 'straight, sigmoid or concave ' convex,

368 'Distal carpals I and II - shape of combined proximal surface ' / flat_or_irregular__ strongly_and_smoothly_convex_,

369 'Metacarpal I - height of medial condyle ventrally compared to lateral condyle ' / higher lower,

370 'Metacarpal II - medial expansion of proximal end ' / 'much greater than distal width, >119%' 'subequal or less than distal width, <120%',

371 'Metacarpal III - length compared to metacarpal II ' / '>79% ' '<80%',

372 'Metacarpal III - distal extent of contact with metacarpal II ' / extensive_intermetacarpal_space_ metacarpals_in_contact_for_almost_all_of_their_length_,

373 'Metacarpal III - position of proximal end ' / mostly_lateral_to_metacarpal_II__ mostly_ventral_to_metacarpal_II_,

374 'Manual phalanx II-2 - length compared to II-1 ' / '<101%' '101-119%' '120-200%' '>200%',

375 'Manual phalanx II-1 - expansion of lateral edge ' / fully_concave_ 'with low convexity developed on at least portion of shaft, maximum shaft width <161% of minimum width of metacarpal II' 'with deep convexity, >160% of minimum width of metacarpal II',

376 'Manual digit II - length of II-1 and II-2 compared to metacarpal II ' / '>100% ' '<101%',

377 'Manual phalanx I-1 - ventral curvature ' / straight_ bowed,

378 'Manual ungual I - dorsal projection of dorsal curve ' / 'with articular surface oriented vertically, dorsal edge of at least mid portion of ungual does not project dorsally over the level of the proximodorsal ungual corner' dorsal_edge_of_at_least_mid_portion_of_ungual_does_project_dorsally_over_the_level_of_the_proximodorsal_ungual_corner_,

379 'Manual ungual II - dorsal projection of dorsal curve ' / 'with articular surface oriented vertically, dorsal edge of at least mid portion of ungual does not project dorsally over the level of the proximodorsal ungual corner' dorsal_edge_of_at_least_mid_portion_of_ungual_does_project_dorsally_over_the_level_of_the_proximodorsal_ungual_corner_,

380 'Manual phalanx III-3 - length compared to combined lengths of III-1 and III-2 ' / '<93% ' '93-109%' '>109%',

381 'Ilium - height of preacetabular process, dorsal extent over line drawn between bases of peduncles, compared to height at base of postacetabular process ' / '<90% ' '90-150%' '>150%',

382 'Ischium - minimum anteroposterior depth of shaft distal to obturator process compared to pubic shaft ' / '<80%' '80-130%' '>130%',

383 'Tibia - length compared to femur ' / shorter_or_equal_ longer,
384 'Tibia - anterior constriction at base of lateral condyle in proximal view ' / lateral_condyle_does_not_laterally_overlap_incisura_tibialis_ overlap_present,
385 'Metatarsus - length compared to femur ' / '>49%' '37-49%' '<37%',
386 'Metatarsal II - depth at midshaft ' / less_or_equal_to_transverse_width_at_midshaft__ greater_than_transverse_thickness_,
387 'Metatarsus - distal extent of metatarsal II-IV contacts ' / shafts_separated__ shafts_appressed,
388 'Metatarsus - minimum transverse width distally compared to proximal width ' / '>59% ' '<60%',
389 'Metatarsal I - torsion ' / absent_ 'present, so that hallux faces medially or posteriorly ',
390 'Pedal ungual I - length on straight line compared to ungual III ' / '<90% ' '>89%',
391 'Manual ungual III - length on straight line compared to ungual II ' / '<96%' '>95% ',
392 'Metatarsus - transverse slenderness at midshaft ' / '>50% of length ' '30-50%' '13-29%' '<13%',
393 'Ilium - anteroposterior length of pubic peduncle, measured halfway down' / over_twice_as_long_as_ischial_peduncle_measured_halfway_down 'less than twice, but still longer than ischial peduncle ' equal_or_shorter,
394 'Pedal phalanx III-1 - slenderness ' / 'length >139% transverse width ' '<140%',
395 'Humerus - transverse width of distal end ' / '<200% minimum shaft width ' '200-269%' '>269%',
396 'Humerus - transverse extent of ectepicondyle ' / '<20% of distal humeral width ' '>19%',
397 'Metatarsal I - transverse width of distal condyle ' / '<81% of transverse width of distal condyle of metatarsal III ' '>80%',
398 'Metacarpal II - length compared to humerus ' / '>32%' '<33%',
399 'Jugal - dorsoventral expansion of anterior process ' / 'expansion dorsal to ventral orbit margin <6% of orbit+jugal height' '>5%',
400 'Chevrons - anteroventral process on distal chevrons ' / absent_ present,
401 'Metacarpal III - depth of distal articular groove ' / absent_ present,
402 'Scapula - dorsoventral depth of acromion ' / '>20% of total scapular depth ' '<21%',
403 'Metatarsus - medial extent of metatarsal IV posterior to III ' / 'extensive, often leading to L-shaped metatarsal III ' slight_to_absent,
404 'Nasals - transverse convexity of dorsal surface ' / concave_to_slightly_convex__ 'highly convex, forming median crest',
405 'Ectopterygoid - anteroposterior position ' / posterior_to_palatine__ lateral_to_palatine_,
406 'Pterygoid - orientation of palatal process ' / 'within 20 degrees of ventral jugal edge, palatal process completely dorsal to ventral margin of jugal ' 'projects strongly downward (>20 degrees), palatal process mostly ventral to jugal',
407 'Articular - contact with surangular ' / sutured_ fused,
408 'Cervical ribs - contact with vertebrae ' / mobile_or_sutured_ fused,
409 'Caudal vertebrae - pneumaticity of caudal centra 1-5 ' / absent_ pleurocoels_present,
410 'External mandibular fenestra - anteroposterior placement ' / 'length of mandible posterior to fenestra <140% orbit+jugal height ' '>139%',
411 'Manual ungual II - presence ' / present_ absent,
412 'Ilium - convexity of anterodorsal edge ' / convex_or_flat with_concavity,
413 'Basioccipital - basal tubera size' / present_ absent,
414 'Postorbital - length of anterior process ' / shorter_than_ventral_process_ equal_or_longer_than_ventral_process_but_without_thin_extension_along_orbit 'longer than ventral process, with thin extension along orbit ',
415 'Postorbital - angle between ventral and posterior processes (measured with vertex equidistant between orbit, supratemporal and laterotemporal fenestrae, through tips of processes)' / '<120 degrees ' '>119 degrees ',
416 'Scapula - medial curvature' / 'present, anterior end placed medially to blade ' absent,
417 'Scapula - sectional shape of posterior half ' / thin_blade_ 'ventrally thick, drop-shaped ',
418 'Coracoid - convexity of medial (posterior in Aves) surface ' / flat_ with_fossa,
419 'Ulna - height of coronoid process ' / prominent_ low_or_absent,
420 'Ulna - height of proximomedial process ' / low_or_absent_ prominent,
421 'Ulna - anterior surface of proximal third ' / smooth_ with_longitudinal_ridge_,
422 'Ulna - proximal extent of distal articular surface up posterolateral side ' / slight_ over_half_of_ulnar_depth_,
423 'Ulna - position of thickest portion of distal end' / near_medial_margin_ near_middle_or_lateral_margin,
424 'Ulna - anterolateral surface distally ' / convex_to_shallowly_depressed_ well_defined_radial_sulcus_extending_to_distal_articulation_,
425 'Ulna - proportions of distal end ' / transversely_compressed_to_equilateral__ 'transverse width greater, but less than 201% ' transverse_width_greater_than_200%_,
426 'Radius - dorsal projection of distal end ' / absent_ 'present, forming flange',
427 'Radiale - size' / '<90% of distal ulnar depth ' '>89%',
428 'Ulnare - convexity of distal edge' / convex_to_straight_ 'slightly concave (1-15% of ulnare depth measured perpendicular to line across tips of rami), forming metacarpal incisure to make ulnare heart-shaped' 'deeply concave (>15%), making ulnare V-shaped',
429 'Ilium - ventral extent of postacetabular process ' / completely_dorsal_to_ischial_peduncle_tip_ extends_ventral_to_ischial_peduncle_,
430 'Sternum - posterior expansion of posteromedian process' / absent present_but_posteriorly_convex present_and_posteriorly_concave__,
431 'Pubis - distal extent of posterior median groove ' / 'proximally restricted, posterodorsal edge of pubic boot rounded ' 'distally extending between most of pubic boots, posterodorsal edge of pubic boot sharply ridged forming hypopubic cup ',
432 'Tibia - midshaft transverse diameter compared to femur ' / '>80% ' '<81%',

433 'Femur - posterior surface of femoral head ' / transversely_flat_to_convex_ with_vertical_groove_near_medial_margin,
434 'Manual phalanx I-1 - convexity of distoventral surface proximal to condyles ' / convex_to_flat_ concave_to_form_flexor_fossa_,
435 'Tibia - angle of anterior edge of cnemial crest ' / '>10 degrees from anterior shaft edge ' '<11 degrees',
436 'Tibia - orientation of cnemial crest in proximal view ' / mostly_anterior_ mostly_lateral,
437 'Tibia - proximal extent of fibular crest ' / separate_from_lateral_condyle__ continuous_with_lateral_condyle_,
438 'Braincase - prominence of crista prootica ' / slight_convexity_to_small_ridge_ well_developed_ridge prominent_overhanging_ala_,
439 'Astragalus - contact between ascending process and fibula ' / absent_ present,
440 'Basisphenoid - expansion' / unexpanded_ 'expanded ventrally, posteriorly and laterally due to extensive pneumatization ',
441 'Supraoccipital - posterior median surface ' / pronounced_sagittal_crest__ concave_to_slightly_convex,
442 'Vomer - posterior extent ' / ends_anterior_to_subtemporal_fenestra__ extends_posterior_to_anterior_edge_of_fenestra,
443 'Dentary - angle of symphysis ' / '>45 degrees from dorsal dentary margin ' '<46 degrees',
444 'Dentary teeth - labiolingual compression of teeth 1-2' / 'labiolingual width <90% of mesiodistal length ' '>89%',
445 'Teeth - labiolingual compression of lateral tooth crowns ' / 'labiolingual width <80% of FABL ' '>79%',
446 'Cervical vertebrae - transverse convexity of ventral centrum surface' / convex_or_flat__ concave,
447 'Dorsal vertebrae - height of neural arch in mid dorsals (4-6)' / distance_from_neurocentral_suture_to_prezygapophysis_less_than_height_of_anterior_articular_surface__ greater,
448 'Dorsal vertebrae - diameter of parapophysis on mid dorsals (4-6)' / '<66% height of anterior articular surface' '>65% ',
449 'Dorsal vertebrae - division of infrazygapophyseal fossae in mid dorsals (4-6)' / undivided_ accessory_centrodiapophyseal_laminae_present,
450 'Dorsal vertebrae - inclination of diapophysis in mid dorsals (4-6)' / inclined_or_horizontal_ declined,
451 'Dorsal vertebrae - anterior bifurcation of neural spine in dorsal view ' / absent_ present,
452 'Dorsal vertebrae - posterior bifurcation of neural spine in dorsal view ' / absent_ present,
453 'Sacral vertebrae - fusion of neural spines ' / all_separate at_least_some_fused_into_a_continuous_lamina_,
454 'Furcula - curvature of arms ' / 'straight or curved laterally to form V-shape ' 'curved medially to form U-shape ',
455 'Scapula - dorsal edge of scapular blade ' / straight_or_evenly_curved__ with_flange_,
456 'Humerus - size of bicipital crest' / 'low, projected <45% of minimum humeral width from humeral head' 'moderate, projected 45-100% of minimum humeral width ' 'prominent, projected >100% of minimum humeral width ',
457 'Humerus - angle of bicipital crest in proximal view ' / '<45 degrees from long axis of humeral head' 'angled laterally/posteriorly 45 degrees or more from long axis of humeral head',
458 'Humerus - separation of bicipital crest from humeral head ' / confluent_ 'separated laterally (posteriorly in Aves) by capital groove',
459 'Humerus - shape of bicipital crest' / with_convex_outer_edge_ with_straight_to_concave_outer_edge_,
460 'Humerus - posteromedial surface of midshaft ' / flat_ with_crest__,
461 'Humerus - anterior surface proximal to ectepicondyle ' / flat_ with_tuberosity_,
462 'Humerus - angle of torsion between long axes of proximal and distal ends ' / '>32 degrees ' '<33 degrees ',
463 'Humerus - anterior flexure of distal end ' / significant_ absent_or_slight_,
464 'Humerus - shape of entepicondyle ' / hemispherical_ 'proximodistally elongate, crest-like',
465 'Humerus - anteromedial surface proximal to entepicondyle ' / flat_ with_longitudinal_groove,
466 'Metacarpal I - ventrolateral surface ' / unprojected_ with_flange_underlying_metacarpal_II,
467 'Metacarpal III - convexity of lateral margin ' / straight_or_concave_ convex,
468 'Manual ungual II - length compared to metacarpal II ' / shorter_or_equal_ longer,
469 'Ilium - distance between dorsal edge of mid ilium and sacral neural spines ' / 'marginal, ilia almost contact neural spines ' significant,
470 'Ilium - lateral surface of posterodorsal corner of postacetabular process ' / flat_ with_small_rugosity_ with_large_tuber,
471 'Ilium - transverse compression of pubic peduncle distal surface' / more_than_twice_as_long_as_wide__ 'length 50-200% of width ' less_than_half_as_long_as_wide,
472 'Ilium - angle pubic peduncle distal articular surface projects ' / '>17 degrees anteriorly' 17_degrees_anteriorly_to_9_degrees_posteriorly '>9 degrees posteriorly',
473 'Ilium - ventral extent of antitrochanter' / ends_proximal_to_articular_surface_of_ischial_peduncle_ extends_to_ventral_edge_of_ischial_peduncle,
474 'Ilium - shape of antitrochanter ' / with_flattened_ventral_side__ hemispherical,
475 'Ischium - proximal enclosure of obturator notch ' / absent_ 'present, process on ventral edge of pubic peduncle curves distally',
476 'Ischium - proximodorsal position of mid dorsal process'' apex' / '<67% down ischium ' '>66%',
477 'Pubis and ischium - shape of subacetabular contact ' / straight_or_simply_curved sinuous,
478 'Pubis - anteroposterior expansion of distal shaft ' / less_than_twice_minimum_shaft_depth_ at_least_twice_,

479 'Femur - angle of femoral head to shaft ' / declined_to_perpendicular__ inclined,
480 'Femur - anteroposterior constriction of femoral head in proximal view ' / at_most_constricted_to_80%_of_medial_head_width_ 'constricted to <80% of medial head width ',
481 'Femur - anteroposterior width of greater trochanter ' / narrower_than_or_equal_to_width_of_femoral_head_ wider,
482 'Femur - ventral constriction of femoral head ' / present absent,
483 'Tibia - depth of incisura tibialis compared to proximal width of tibia' / '>15%' '<16%',
484 'Tibia - transverse compression of proximal end ' / longer_than_wide_ equal_or_wider_than_long_,
485 'Tibia - distal extent of fibular crest ' / 'ends at <45% of tibial length ' '>44%',
486 'Fibula - angle of proximal edge ' / '<16 degrees from perpendicular to long axis of shaft ' '>15 degrees anteroproximally',
487 'Fibula - proximodistal position of iliofibularis tubercle apex' / 'placed <41% down length of fibula ' '>40%',
488 'Fibula - transverse width of posterior half in proximal view ' / equal_to_or_wider_than_anterior_half_ 'narrower, forming posteriorly tapered surface ',
489 'Tibia - distal exposure of lateral condyle ' / 'absent, covered by astragalocalcaneum ' present_due_to_reduction_in_astragalus_,
490 'Femur - convexity of proximal surface between head and greater trochanter ' / convex_to_slightly_concave strongly_concave,
491 'Maxillary teeth - position of largest teeth' / in_anterior_third_larger in_middle_third_ in_posterior_third_,
492 'Tibia - anterior exposure of mediodistal corner of tibia ' / covered_by_astragalus exposed,
493 'Caudal vertebrae - separation of tip of distal caudal prezygapophyses ' / unseparated bifurcated,
494 'Braincase - confluence of otic recess and lateral depression ' / separate within_common_depression,
495 'Postorbital and jugal - minimum anteroposterior length of postorbital bar ' / less_than_or_equal_to_minimum_anteroposterior_length_of_lacrimal_ greater,
496 'Lacrimal - separation from postorbital dorsal to orbit ' / wide narrow absent,
497 'Premaxillary teeth - labiolingual compression ' / longest_axis_mesiodistal_ longest_axis_labiolingual,
498 'Premaxillary teeth - lingual surface ' / flat with_central_basoapical_ridge_,
499 'Infratemporal fenestra - posterior constriction caused by squamosal and quadratojugal ' / 'greatest anteroposterior width of posterior intrusion into fenestra / greatest anteroposterior width of fenestra if protrusion were absent <10% ' '>9%',
500 'Nasals - convexity of dorsolateral surface ' / 'concave to right-angled ' 'sharply convex, forming paired ridges or crests',
501 'Ilium - lateral surface dorsal to acetabulum ' / flat_or_slightly_rounded__ with_well_defined_vertical_ridge,
502 'Nasal - lateral surface along or inside antorbital fossa ' / solid_ pierced_by_at_least_one_pneumatic_foramen,
503 'Articular - transverse width of retroarticular process ' / equal_to_narrower_than_mandible_anterior_to_glenoid_ wider_than_mandible_anterior_to_glenoid_,
504 'Basioccipital and basisphenoid - transverse width across basipterygoid processes ' / equal_to_less_than_anteroposterior_length_between_anterior_edge_of_basipterygoid_processes_and_posterior_edge_of_basal_tubera__ greater,
505 'Ischium - transverse convexity of acetabular surface ' / convex_or_flat__ concave_due_to_sagittal_groove,
506 'Paraquadrate foramen - dorsoventral size ' / '<18% of quadrate height ' '>17%',
507 'Paraquadrate foramen - composition of lateral edge ' / formed_at_least_partially_by_quadrate__ formed_completely_by_quadratojugal_,
508 'Axis - dorsal transverse expansion of neural spine ' / tapered_to_equal_to_base_ 'expanded dorsally, resulting in epineurapophyseal processes ',
509 'Ilium - extent of supracetabular crest along pubic peduncle ' / present_on_most_of_peduncle__ present_on_minority_of_peduncle_if_at_all_,
510 'Scapula - convexity of dorsal acromial edge ' / convex_to_straight_ concave,
511 'Basisphenoid - lateral walls of basisphenoid recess ' / unmarked_ have_muscle_scars_,
512 'Quadratojugal - convexity of external surface ' / flat_ 'flexed at posterior border, so that distinct posterior face is present ',
513 'Nasal - convexity of lateral edge ' / convex_or_straight__ 'concave, so that paired nasals are hourglass-shaped',
514 'Metatarsal III - shape of medial edge when articulated, taking into account curvature of shaft' / distally_concave_to_slightly_convex_ with_deep_convexity_localized_distally_,
515 'Ilium - convexity of pubic peduncle articular surface ' / convex_with_angled_anterior_and_posterior_components__ convex_and_rounded_ flat_or_concave_,
516 'Ilium - distal anteroposterior expansion of pubic peduncle ' / unexpanded_ expanded,
517 'Axis - lateral surface of centrum ' / solid_ pneumatized_by_pleurocoel,
518 'Maxilla - medial wall of maxillary antrum ' / solid_ pneumatized_by_medial_antral_fenestra,
519 'Surangular - dorsoventral depth at intersection of dentary and external mandibular fenestra ' / '>50% mandibular depth at that level ' '<51%',
520 'Pedal ungual II - convexity of posteroventral edge between articular facet and flexor tubercle' / convex_or_straight_ concave_due_to_notch_at_base_of_tubercle_,
521 'Sternum - ossification ' / unossified_in_adult__ ossified,
522 'Pedal digit II - length compared to pedal digit IV, excluding metatarsals and unguals ' / '>79% ' '<80%',

523 'Metatarsal III - transverse medial and lateral bulges on shaft just proximal to articular condyle' / absent_ present,
524 'Metatarsal II - length compared to metatarsal IV' / 'much shorter, <98% of length of metatarsal IV' 'subequal, lengths within 2% of each other ' 'much longer, >102% the length of metatarsal IV',
525 'Metatarsal IV - projection of lateral surface dorsal to lateral flexor ridge on distal end' / unprojected_ with_longitudinal_ridge_,
526 'Maxilla - posterior extent of postantral wall ' / no_greater_than_interfenestral_wall__ extends_posterior_to_interfenestral_wall_,
527 'Teeth - texture of labial and lingual surfaces ' / smooth_ fluted_basoapically_,
528 'Dorsal vertebrae - anterior projection of interspinal ligament scars on neural spine ' / evenly_projected_along_spine_ 'more projected dorsally, forming beak-like process ',
529 'Maxilla - lateral exposure of promaxillary fenestra ' / covered_by_lateral_lamina_of_dorsal_process__ at_least_partially_visible_laterally__,
530 'Antorbital fossa - proportions ' / longer_than_tall equal_to_taller_than_long,
531 'Jugal - length of posterior process ' / '>15% of total jugal length ' '<16%',
532 'Furcula - cross section of omal ends of arms ' / posteriorly_convex_to_flat_ posteriorly_concave,
533 'Cervical vertebrae - post-axial central pneumatization ' / pleurocoels_present pleurocoels_absent,
534 'Caudal vertebrae - relationship of zygapophyses ' / prezygapophyses_articulate_ventral_to_postzygapophyses prezygapophyses_articulate_dorsal_to_postzygapophyses,
535 'Uncinate processes - articulation to dorsal ribs ' / unfused fused,
536 'Ulna - posterior projection of dorsal condyle ' / unprojected_or_minimally_projected_ distinctly_projected_as_a_semilunate_ridge_,
537 'Ischium - distal extent of pubic contact ' / limited_to_acetabular_area_and_sometimes_obturator_process 'extensive, along most of ischial shaft',
538 'Nasals - transverse convexity of combined dorsal surface ' / relatively_flat_to_concave strongly_convex,
539 'Jugal - convexity of anterior tip ' / convex concave_due_to_bifurcation,
540 'Radius - length compared to humerus ' / '<51% ' '50-90%' '91-110%' '>110%',
541 'Premaxillae - form of contact at symphysis ' / unfused fused_anteriorly_but_not_posteriorly completely_fused,
542 'Dentaries - form of contact at median ' / unfused_ fused,
543 'Dentary - anteroventral surface of tip ' / smooth_ with_groove_extending_anterolaterally_from_ventral_corner_of_symphysis__,
544 'Premaxilla - length of ventral margin ' / '<91% of orbit+jugal height ' '>90%',
545 'Premaxilla - length of dorsal process ' / 'from tip of snout, extends <61% of preorbital length ' '>60%',
546 'Vomer - extent of contact with pterygoid ' / broad_ narrow absent,
547 'Palatine - extent of contact with pterygoid ' / 'long, overlapping ' 'short, vertical',
548 'Palatine - contact with premaxilla ' / absent present,
549 'Vomer - contact with premaxilla ' / present absent,
550 'Basisphenoid - anteroposterior position of basipterygoid processes ' / located_basally_on_basisphenoid_body located_anteriorly_on_parasphenoid_rostrum_,
551 'Basisphenoid - orientation of articulation with pterygoid' / anteroventral mediolateral dorsoventral,
552 'Pterygoid - convexity of articulation with basisphenoid ' / concave flat_to_low_and_convex 'highly convex, forming stalk ',
553 'Pterygoid - angle between palatal process and posterior end in lateral view' / 'present, posterior end kinked dorsally' 'absent, element straight ',
554 'Interorbital septum - ossification' / unossified ossified,
555 'Mesethmoid - anterior extent ' / barely_surpasses_frontals_if_at_all_ extends_far_anterior_to_frontals,
556 'Basisphenoid - lateromedial position of ossified eustachian tubes ' / lateral medial_but_widely_separated medial_and_adjacent_or_median_and_confluent_,
557 'Basisphenoid - ventral surface anterior to basisphenoid recess ' / solid perforated_by_paired_foramina_or_medain_foramen_for_ossified_eustachian_tubes,
558 'Squamosal - length of ventral process ' / projects_noticeably_ventral_to_quadrate_notch_ absent,
559 'Quadrate - form of articulation with pterygoid ' / pterygoid_broadly_overlaps_medial_surface_of_pterygoid_process restricted_to_anteromedial_edge_of_process_or_body_of_quadrate_,
560 'Quadrate - position of articulation with pterygoid ' / extends_to_tip_of_pterygoid_process_ 'extends to pterygoid process, but ends before tip ' restricted_from_pterygoid_process,
561 'Quadrate - convexity of pterygoid articulation ' / flat_or_slightly_convex highly_convex,
562 'Quadrate - anterolateral surface on dorsal portion of pterygoid process ' / flat with_tubercle,
563 'Quadrate - form of articulation with quadratojugal ' / planar condylar trochlear,
564 'Quadrate - proximal articulation with prootic and/or opisthotic ' / absent present,
565 'Quadrate - number of distal condyles on posterior edge' / two_or_less 'three, with posterior condyle between lateral and medial condyles ',
566 'Articular - pneumaticity ' / solid pneumatized_by_diverticulum_of_the_tympanic_airsac_,
567 'Splenial - anterior extent ' / does_not_extend_close_to_mandibular_symphysis_ 'extends close to, but does not participate in mandibular symphysis ' participates_in_mandibular_symphysis_,
568 'Dentaries - transverse convexity of posterior symphysis ' / concave flat_or_convex,
569 'Dentaries - dorsal surface of fused symphysis ' / solid with_one_or_more_foramina_,
570 'Dentaries - arrangement of symphyseal foramina ' / median paired,

571 'Dentaries - position of symphyseal foramina ' / on_posterior_edge_of_symphysis_ on_dorsal_surface_of_symphysis,
572 'Splenial - medial overlap of Meckelian groove ' / 'absent, Meckelian groove exposed ' 'present, Meckelian groove hidden ',
573 'Jugal - contact with postorbital ' / present absent,
574 'Frontal - contact with parietal ' / unfused fused,
575 'Cervical vertebrae - convexity of posterior articular surface ' / flat_or_concave convex 'concave dorsoventrally, convex horizontally ',
576 'Dorsal vertebrae - size of hypapophyses on dorsals posterior to third ' / absent_or_small large_on_at_least_some,
577 'Dorsal vertebrae - number ' / more_than_12 12 11 10_or_less,
578 'Dorsal vertebrae - anteroposterior position of parapophyses ' / anterior_to_diapophyses_ at_same_level_as_diapophyses_,
579 'Dorsal vertebrae - transverse compression of centra ' / length_less_than_midlength_width_ length_equal_to_or_greater_than_midlength_width_,
580 'Dorsal vertebrae - convexity of lateral centrum surface ' / convex_to_slightly_depressed_ 'highly concave due to deep fossa, which may be dorsoventrally compressed to form a groove ',
581 'Dorsal vertebrae - connection of adjacent transverse processes ' / 'absent, processes separate' processes_connected_by_ossified_ligaments_,
582 'Dorsal vertebrae - fusion ' / no_dorsals_fused_together_ at_least_two_anterior_dorsals_fused_to_form_notarium,
583 'Sacrum - length of ancestral avepod thirteenth dorsal and first two sacral centra ' / at_least_one_longer_or_equal_to_more_anterior_and_posterior_centra all_shorter_than_surrounding_centra at_least_one_adjacent_centrum_also_shorter_than_surrounding_centra_,
584 'Caudal vertebrae - length of transverse processes in caudals 1-5' / equal_to_or_longer_than_centrum_width shorter_than_centrum_width_,
585 'Caudal vertebrae - number fused into pygostyle ' / more_than_five_ three_to_five two,
586 'Gastralia - ossification ' / ossified unossified,
587 'Sternum - extent of ventral median keel ' / restricted_to_the_posterior_part_ extends_from_close_to_anterior_edge_to_posterior_part restricted_to_the_anterior_part_,
588 'Sternum - pneumatization of dorsal surface ' / solid invaded_by_one_or_more_diverticula_of_the_clavicular_airsac,
589 'Sternum - pneumatization of lateral edge between sternal rib articulations ' / solid invaded_by_diverticula_of_the_clavicular_airsac_,
590 'Sternum - distance between coracoid sulci ' / wide narrow sulci_overlap,
591 'Sternum - number of sternal ribs that contact sternum ' / two_or_less three four five six seven_or_more,
592 'Sternum - topology of ventral surface lateral to midline ' / smooth 'with longitudinal ridge, an intermuscular line between the m. supracoracoideus and m. pectoralis ',
593 'Sternum - shape of posterior edge near midline ' / unprojected_without_fenestra_ with_free_posteromedial_processes 'with posteromedial processes fused distally with posteromedian process, forming paired fenestrae ',
594 'Clavicles - median fusion ' / fused_to_form_furcula_ unfused,
595 'Furcula - shape of omal tips in dorsal/ventral view ' / flat_to_rounded_ pointed,
596 'Furcula - shape of ventral edge ' / convex flat_to_concave,
597 'Scapulocoracoid - convexity of mobile articulation ' / 'concave on scapula, convex on coracoid' flat_on_both_bones_ 'convex on scapula, concave on coracoid ',
598 'Coracoid - topology of anterior (medial in Aves) edge near level of scapular articulation ' / unprojected with_procoracoid_process_,
599 'Coracoid - convexity of posterior (lateral in Aves) margin of distal portion ' / concave_or_straight convex,
600 'Coracoid - pneumatization of proximal area, near scapular articulation or coracoid tuber ' / solid invaded_by_diverticula_of_the_clavicular_airsac_,
601 'Coracoid - pneumatization of mediodistal (posterodistal in Aves) area, near sternal articulation ' / solid invaded_by_diverticula_of_the_clavicular_airsac_,
602 'Coracoid - shape of posterior (lateral in Aves) edge just proximal to sternal articulation ' / confluent_with_more_proximal_part_of_edge_ projected_into_lateral_process_,
603 'Coracoid - topology of posterior area of lateral surface (lateral area of anterior surface in Aves)' / smooth 'with ridge extending distally from coracoid tuber to sternal articulation, an intermuscular line between origins of the m. supracoracoideus and m. coracobrachialis posterior ',
604 'Coracoid - shape of acrocoracoid process ' / straight_or_curved_laterally_ curved_medially,
605 'Coracoid - position of supracoracoid nerve ' / 'passes through coracoid, resulting in coracoid foramen' 'does not penetrate coracoid, coracoid foramen absent ',
606 'Coracoid - convexity of external surface surrounding coracoid foramen ' / concave flat_or_convex,
607 'Coracoid - size of portion proximal to biceps tuber ' / 'present, forming distinct protrusion or area of the bone' 'absent, biceps tuber at same level as scapular articulation ',
608 'Scapula - curvature of shaft in lateral view ' / straight_or_dorsally_curved_ ventrally_curved,
609 'Scapula - curvature of acromion ' / lateromedially_straight_or_medially_curved_ anterior_portion_curved_laterally,
610 'Humerus - convexity of proximal edge of humeral head when viewed perpendicular to long axis' / concave_to_flat_ convex,
611 'Humerus - closure of capital groove ' / groove_open groove_closed,
612 'Humerus - convexity of anterior edge of head in proximal view ' / straight_to_convex_ concave,

613 'Humerus - separation of head from more distal humerus on anterior surface ' / confluent separated_by_transverse_groove_for_acrocoracohumeral_ligament_,
614 'Humerus - orientation of deltopectoral crest in proximal view ' / in_line_with_long_axis_of_humeral_head angled_from_long_axis_of_humeral_head_,
615 'Humerus - convexity of proximolateral/posterior surface of deltopectoral crest ' / convex_or_flat_ concave,
616 'Humerus - surface of deltopectoral crest ' / solid perforated_by_fenestra_,
617 'Humerus - surface of bicipital crest ' / smooth_ scar_or_fossa_for_m._scapulohumeralis_posterior,
618 'Humerus - position of m. scapulohumeralis posterior insertion ' / 'medial (anterior in Aves) surface of bicipital crest ' distal_surface_of_bicipital_crest_ 'lateral (posterior in Aves) surface of bicipital crest ',
619 'Humerus - medial (anterior in Aves) projection of bicipital crest ' / absent moderate highly_convex,
620 'Humerus - proximal pneumaticity ' / solid 'invaded by diverticulum of the clavicular airsac near base of lateral (posterior in Aves) surface of bicipital crest ',
621 'Humerus - orientation of ectocondyle ' / mostly_proximodistal_ mostly_transverse,
622 'Humerus - shape of entocondyle ' / rounded 'ridge-like',
623 'Humerus - angle of distal edge ' / perpendicular_to_shaft_or_angled_so_that_ectocondyle_is_further_distal_ angled_so_that_entocondyle_is_further_distal_,
624 'Humerus - anterior surface just proximal to condyles ' / flat concave_for_origin_of_M._brachialis_inferior,
625 'Humerus - size of entocondyle ' / long_axis_shorter_than_long_axis_of_ectocondyle_ equal_or_longer,
626 'Humerus - lateral side of posterior surface on distal end ' / flat with_longitudinal_groove_for_passage_of_m._scapulotriceps_,
627 'Humerus - medial side of posterior surface on distal end ' / flat 'with longitudinal groove for passage of m. humerotricipitalis, confluent with olecranon fossa ',
628 'Ulna - separation of lateral and medial cotylae ' / confluent_or_only_separated_by_ridge_ separated_by_groove_,
629 'Ulna - convexity of lateral cotyla ' / concave_or_flat_ convex,
630 'Ulna - anteroproximal surface ' / flat_ scar_for_the_insertion_of_the_M._biceps_brachii_ tubercle_for_the_insertion_of_the_M._biceps_brachii_,
631 'Ulna - anteromedial surface of proximal end ' / flat_ scar_for_insertion_of_m._brachialis_,
632 'Radius - posterolateral surface ' / flat_ with_long_scar_for_m._pronator_teres_ with_long_groove,
633 'Ulnare - length of medial (ventral) ramus ' / shorter_than_lateral_ramus__ subequal_to_lateral_ramus_ longer_than_lateral_ramus_,
634 'Metacarpal III - distal contact with metacarpal II ' / 'absent, contacting or sutured ' fused,
635 'Metacarpal III - minimum transverse width compared to minimum transverse width of metacarpal II ' / '>49% ' '<50%',
636 'Metacarpal I - development of medial process for insertion of m. extensor metacarpi ulnaris' / unprojected_or_slightly_convex__ 'greatest length of extensor process (measured medially from proximal metacarpal articular surface and lateralmost part of medial edge of metacarpal I) 8-20% of transverse width between medial edge of distal articular surface of metacarpal I and lateral edg' '21-30%' '>30%',
637 'Metacarpal I - proximodistal convexity of medial edge ' / concave_or_straight_ convex,
638 'Metacarpal II - proximal part of ventral surface ' / flat_ pisiform_process_for_retinaculum_flexorum,
639 'Distal carpal I - ventral surface ' / flat_ with_infratrochlear_fossa_for_ventral_radiocarpometacarpal_ligament,
640 'Metacarpal III - distal extent ' / proximal_to_or_subequal_to_metacarpal_II__ distal_to_metacarpal_II,
641 'Metacarpal II - dorsolateral surface near middle of shaft ' / smooth_ scar_for_insertion_of_m._extensor_carpi_ulnaris_longus__ intermetacarpal_tubercle_or_process_for_insertion_of_m._extensor_carpi_ulnaris_longus,
642 'Manual phalanx II-1 - projection of laterodistal margin ' / does_not_extend_distally_past_articular_surface_ extends_past_surface_as_internal_index_process_,
643 'Pubis - contact with ilium ' / articulated_ fused,
644 'Ischium - contact with ilium ' / articulated_ fused,
645 'Pubis - contact with ischium ' / articulated_ fused,
646 'Ischium - contact between mid dorsal process and ilium ' / absent_ present,
647 'Ischium - contact between proximodorsal process and ilium ' / absent_ present,
648 'Ilium - position of antitrochanter ' / mostly_posterior_to_acetabulum mostly_dorsal_to_acetabulum,
649 'Pelvis - development of pubic tubercle on anterolateral surface of proximal pubis or pubic peduncle of ilium' / undeveloped_or_a_scar_ 'process for attachment of inguinal ligament, M. obliquus abdominus and/or M. ambiens ',
650 'Ilia - contact between dorsal edges of preacetabular processes or between dorsal edges and sacral neural spines' / absent_or_freely_contacting_ fused,
651 'Ilium - overlap with last dorsal rib ' / absent present,
652 'Ilium - orientation of postacetabular process in section ' / mostly_vertical mostly_horizontal,
653 'Ilium - internal surface of postacetabular process ' / flat with_renal_fossa_for_posterior_portion_of_kidney,
654 'Pubis - transverse compression of midshaft ' / anteroposteriorly_compressed_or_equal_ transversely_compressed_,

655 'Femur - convexity of anterior edge of distal end' / convex_to_slightly_concave_ 'markedly concave, forming deep extensor or patellar groove ',

656 'Femur - confluence between lateral condyle and ectocondylar tuber ' / separated_by_notch_ joined_into_tibiofibular_crest_,

657 'Femur - posterior projection of posterior surface just proximal to lateral condyle ' / in_line_with_medial_surface_ distinctly_more_posteriorly_projected_than_medial_surface_,

658 'Femur - transverse position of lateral edge of ectocondylar tuber or tibiofibular crest ' / 'far medial to lateral edge of lateral condyle, <90% of distance across condyles, measured from medial edge ' 'roughly in line with lateral edge of lateral condyle, 90-100% of distance across condyles' 'lateral to lateral edge of lateral condyle, >100% of distance across condyles',

659 'Astragalocalcaneum - anterior projection of medial condyle ' / much_greater_than_lateral_condyle 'slightly greater to less than lateral condyle (measuring from a line across the posterior tip of each condyle, medial condyle extends anteriorly <110% as much as lateral condyle)',

660 'Tibiotarsus - lateral side of anterior surface just proximal to distal condyles ' / flat with_open_longitudinal_extensor_canal_for_ligament_of_m._extensor_digitorum_longus_ ossified_ligament_crossing_extensor_canal_to_form_supratendinal_bridge_,

661 'Astragalus - anterior surface of ascending process ' / concave_or_flat_ with_tubercle_for_attachment_of_retinaculum_bridging_m._tibialis_cranialis_and_m._digitorum_longus_,

662 'Astragalocalcaneum - transverse width of medial condyle ' / '>110% transverse width of lateral condyle (measured from point of greatest constriction between condyles) ' '110-90%' '<90%',

663 'Astragalocalcaneum - convexity of anterior edge of medial condyle ' / highly_convex straight_or_almost_straight_,

664 'Tibia - proximal extent of trochlea for the tibial cartilage on the posterior surface of the distal end ' / proximal_extent_less_than_51%_of_distal_tibiotarsal_transverse_width_ '>50%',

665 'Tibia - definition of lateral and medial edges of trochlea for the tibial cartilage on the posterior surface of the distal end ' / undifferentiated_from_adjacent_bone_or_low_rounded_ridges_ defined_by_sharp_crests_continuous_with_distal_condyles_,

666 'Tibia - distal transverse expansion ' / 'transverse width just proximal to distal condyles >150% of midshaft transverse width ' '<151%',

667 'Metatarsal V - presence ' / present absent,

668 'Tarsometatarsus - proximal surface ' / flat with_central_raised_intercotylar_eminence_,

669 'Tarsometatarsus - proximal part of posterior surface ' / flat raised_hypotarsus_with_flat_posterior_surface raised_hypotarsus_with_grooved_posterior_surface_for_flexor_tendons_ raised_hypotarsus_with_perforated_posterior_surface_for_flexor_tendons_,

670 'Metatarsus - dorsal and ventral surface near proximal end between metatarsals III and IV ' / solid with_vascular_foramen_,

671 'Metatarsus - dorsal and ventral surface near proximal end between metatarsals II and III ' / solid with_vascular_foramen_,

672 'Metatarsal I - posterior curvature ' / 'absent, shaft straight in medial view ' 'present, shaft J-shaped',

673 'Metatarsal II - surface for articulation of metatarsal I ' / flat_or_convex slightly_concave well_developed_fossa,

674 'Metatarsal II - transverse width of trochlea ' / '<80% of metatarsal III trochlea ' '80-130% of metatarsal III trochlea' '>130% of metatarsal III trochlea ',

675 'Metatarsal IV - transverse width of trochlea ' / '<60% of metatarsal III trochlea ' '60-200% of metatarsal III trochlea ' '>200% of metatarsal III trochlea ',

676 'Metatarsus - distal surface between fused metatarsals III and IV ' / solid_ with_vascular_foramen,

677 'Metatarsal III - proximal extent of lateral trochlear edge on posterior surface ' / less_than_or_equal_to_medial_trochlear_edge greater_than_medial_trochlear_edge_,

678 'Chevrons - length of posterodistal process on distal caudals ' / shorter_than_centrum_ longer_than_centrum_,

679 'Exoccipital - length of metotic strut, measured from base of paroccipital process to tip of basal tuber ' / '<35% of height from tip of basal tuber to skull roof ' '>34%',

680 'Basisphenoid - lateral surface ventral to trigeminal (V) nerve foramen and anteroventral to otic recess' / flat_ excavated_by_main_portion_of_anterior_tympanic_recess_,

681 'Basisphenoid - position of anterior tympanic recess ' / extends_posterior_to_basipterygoid_processes__ restricted_to_above_or_anterior_to_basipterygoid_processes_,

682 'Pedal phalanx II-2 - height of distal end' / greater_than_79%_height_of_proximal_end less_than_80%,

683 'Ulna - size of lateral cotyla' / smaller_than_medial_cotyla equal_or_larger_than_medial_cotyla,

684 'Quadratojugal - size ' / 'length of ventral margin >29% of jugal+orbit height ' '<30%',

685 'Frontal - surface of articulation for postorbital ' / smooth_or_slightly_notched__ deeply_notched,

686 'Furcula - cross section of center ' / compressed_dorsoventrally_to_slightly_compressed_anteroposteriorly highly_compressed_anteroposteriorly,

687 'Furcula - width of epicleidial ends ' / equal_or_narrower_than_arms_ broader_than_arms_,

688 'Furcula - width of arms ' / equal_or_narrower_than_center_and_epicleidia_ broader_than_center_and_epicleidia_,

689 'Furcula - anterior surface of hypocleidium ' / flat_or_rounded keeled,

690 'Furcula - symmetry ' / asymmetrical nearly_symmetrical,

691 'Furcula - robusticity ' / 'width halfway down arms <20% of arm length ' '>19%',

692 'Distal caudal vertebrae - separation of postzygapophyses ' / separated_posterior_to_centrum joined_by_lamina_past_centrum,

693 'Metatarsal III - dorsal surface proximal to articular area and extensor fossa ' / flat_to_convex_ concave_forming_fossa_,
694 'Astragalocalcaneum - proximodistal height of lateral condyle ' / '<115% height of medial condyle ' '>114% height of medial condyle ',
695 'Astragalus - anterior surface at base of ascending process ' / without_pitting__ one_or_more_pits_present_,
696 'Coracoid - lateral (anterior in Aves) surface for origin of M. biceps brachii ' / convex_tuber_ convex_ridge flat,
697 'Manual ungual I - exclosure of proximoventral vascular grooves ' / open_ enclosed_forming_proximoventral_foramina,
698 'Maxilla - lateral surface ventral to antorbital fossa ' / flat_ horizontal_ridge_present_,
699 'Pubis - abruptness of pubic boot anteroposterior expansion ' / abrupt_ gradual_over_distal_portion_of_pubis,
700 'Astragalus - anterior surface of center of body ' / proximodistally_convex_to_flat_ with_transverse_groove ;

MATRIX

Dilophosaurus ?0000-1100??0?10?000000??1?000000001?0N1?02?110-
?0010?1001000000????00010110?10{0 1}0000000101?121(0 1)00010000001(0 1)00111?(0
1)0100000001100?0???????000100121000?00NNN1(0 1)0(0 1)00011000110000100-2021(0 1)020100012?0001(0
1)000100100100000NN0000?0200?100310010??000?1(0 1)0(1 2)(0 1)0?{0
1}??0001000000000000110?01??0???0?000000100(0 2)0000101000{0 1}000(0 1)000001012(0 1)100010-1-
001010000?(0 1)?0100-001000?000?0100(1
2)000?10000100000000000000200000000011010001?1010??N100000000??00??0?100?0??11?001?100?0(0
1)000???(0 1)(0 1)0?010000000000001010--0-00010010000100 1?0?110?110100000000??1001000??0010-0(0
1)100?00?0000100000???00??0?-00????0?0?0?----01000010?000-0?????????-010?0?-0100?0-111000-
001000000--20{0 1}?N0000?1101N0---0N010000?00001000000{0 1}--0?11-??11??-0???????100000000

Sinosaurus ?0(0 1)00-1110?-0?1110001000?0-00?0000001?001?1(1 2)011001001(0
1)00?0??01?00????00000(0 1)00?{0 1}0{0 1}1000000101011110?010000?00110111??0??0?0?????{0
1}0?0???????000100?11012???????{1 2}{0 1 2 3}?????????110000100-2{0 1}10{0 1}0{1
2}020001112??10?001002?01??0100000000???0?100(1 2)10010000?0010010?0{0
1}??00?1?0????000001??0?1??????1?00?0{0 1}?00020?00100000000100?0?{0 2}101(0 2)0(0 1)0?010-1-
00100000011?0100-00(1 2)000000101001110?1?10-00??????{1 2 3}?????{0
1}010?1?00???1??10??0??01000?000?00000????????????0???1?0012?00000000??0????0{0 1}??00?00???0?1{0
1}{1 2}0--0-?00?0?000?01??0??-(0 1)1??(0 1)101?0???0?01??0010?1???00?-00100?00??00?{0
1}10000?00?00??0?-00?????????0---?000?0??0???0???????????-0???0?-01-
00????100?????0?1?????????00???????000-?-0000??00?????????00??--??{0 1}{0 1}-
??010??00??????????2?100

Cryolophosaurus
??0????110???01????0???10????????0001?00111001000?00?000000?0?000??????0??????????100??0{0 1}?{0
1}1?1??00?????????00??11{0 1}???2{0 1}00{0 1}0?????0?0??????????????????{0
1}?0???????????????????00??{0 1}???01100??{0 1}00{0
1}??1??0100001001??1000100???????????????????0000000??0(1
2)??????????00????????1????????????????00?????2??0?1?0?00?0?????0????1{1
2}?1??000???0?1?0??00????{1 2}???0010??00?0?01??1?1??{1 2}?????0??????????????2????????????{1
2}???1????00000?0??000???0?????????0??01???12100???00?????????1?10?????0??????{0
1}???00?0001??????00?0??11??110?0??00????0??1??????????????00?00??000?????0????????0???0??????0
0??????0?0??010?????????????????????????????10?0???0-0010??100--{0 1}00??????????0000-
?1??0??0000?00??00??????????????0???-0?????????0?1?1?0

Tachiraptor
??
???1{0
1}?2????????????????????0??0100??0???0?????????
????????????????????0????????00???????????0???
??????????{0
1}?0??001?1???????????????????????????????????????
???????000???0????????????????0???????????????????????????????0????????????????????????????????
???0????????????????0????0
?????????????????????????????????

Berberosaurus
???0??1???
?????????0??10????????0{0
1}0????????????????????????????????????0??????????????????????????????????????0100011?02??100??
0?????????????????????????????0??0???(0
2)?????????????????????????????10??????????????0?????????????????????1?????????????0???????????
????????????????????????0?????????????????????????1???????????00??????????????????????????????
?0?00??000?00?0????????????????????????????????????0??
???0???1??0?????????????????0???000?
?????????????????????????????????

Genyodectes
??????????????????????????000??0??0??0????????0000{0
1}0101110?0???
??010???????????10??????????????????????????????????????
???????0???0????????0?00???????????0???????????????0???????0?????????????1??00?????????????????
??000??????????????????????
??????????????????????{1
2}?????00?????????????????????????????0??????????????0000?????????????????????????0?----

??
?????????????????????????????????
Ceratosaurus ?0001011100-01101000000101?0100000(0 1)0100011100010-101100000-01(0
1)0000000000000000?10{0 1}0?00000101?112000?100(0 1)000(0 1)(0 1)00110012100?00000000-
0???????00010012010110?----1{0 1 2}?????????0{0 1}?00?000-2111002010000{1 2}1(1 2)001000(0 1)1(0
1)02?0100(0 1)(0 1)01000000?????10010?000000000100(0 1)0020??001100???00000(0
1)??0?????0??1?001000010(0 2)00?01000100000?0?0000001000?00001000100000000?01010111000-00000110(0
1)010?10110(0 1)0-00000?0?????11001010???1{1 2}?10?1101011000(0 1)00??0000?100001010--
???01?0012100000000010001?01?1000110110?1?1{0 1}--0-00000010001?00100-01000000?1?0-?00101??11(0
1)0????20-(0 1)0000?00?0010100000000?0?0010-0000-00?0??0---?0000{0 1 2}0101?00?0?????????-010000-
0100010?11000-1011(0 1)1100--100-00?00-?1?111---0000?0000(0 1)100??0(0 1)0??1--??00--0010?-00????????0(0
1)0?(0 1)0(0 1)
Eoabelisaurus ???????100????10?000?00??????????00???0?101201?00?10100100-
010100?00?0??????????????????00??1?1??00??1?0???010?0111?01?00?00?0??{0
1}0??????????0?100?210?21?000010100?01{0 1}???0{0 1}?00?1?0-202100201{0
1}0112000010000?002?0??0011100000000??1???????0000000??010?0(0 1)???0?1?00---
0?00010?0?0?10???0?00??10?112???01010?00?00??00?0??????01011???????????1??0?0{0 1
2}?????0????000?????1010?11?1?01??10002{0 1}1???-
01011?10???21000011??01?00?00???000???0????????????0??000???00????0???0????00??1???1??00?0?{1
2}1???-?00?0000??01?0??0?11????0????0-??11??0?1??????01???1????00??0??0????????????01???000-
???????????010??0?01????????????????-???????0??0?1?????0??{0 1}?1(0 1)0?0????????00000?0?0111--
?1?0??????????????????0??--??0{0 1}-??1????0????????0??00?0?
Rugops ?0001011????????????????????110??????????10120010-
??11???????0????????????????????????0?00?11????0?1??
???0000?00?000??
??00???????100?00?????01000101????1?
???00?10?0?0-
0?????1???0???????????????????????????????????????
??0???1????2???1?0??????????1????????????-
??11???????0??0??00?????????????????????????????0???
??1?????????????1??
Rajasaurus
???????????????1????????0??????????????????????11???11???????0??????????????????????????????
??0?????????10???1{0 1}??100???0??1???{0
1}0????????????????????????00?1??100??0?000?{0 1
2}????????????????00???????1?0????????0???????0??????????????????????????0?????????1??????????{0
2}???????0{0 1}?1????????????????????????????1?0?????{0 1}??????????????????????????????1?????{1
2}1?????????????????0??????????????????????????????2??0????0000??????????????????????11--
???????????0?1????????????????????0?????10????????????????0?????????????????????????????????
????100?0?0??0???00???-
0?????00?0?0?????0??????0??????10???0???????????????
Genusaurus
??
???01{0 1}000?0?-
2????0?????0???0??11?011??2?0???000??1???????
???????????????02??????????{0 1}???1?????????????1{0
1}1??0??0????????1??????????????????
????????????????????????001??????????????????????????????????0{0
1}0??????0????0000??????????????0??????????????10??????????????????????0???????????????????????????
??11????0??????0???
???
Arcovenator ????????0??-?1101??010????????????????????????10-
?01100?????0??0?????????????????????????0??1?1????????????????????????????0?????????????????????
??02??10???0?????????????????????0000?1
0?(0 1)?????{0
1}?????????????????0???????????????????????????0?1????0??????????????????????????????0??????????
?????-
0?0????????????????????????????????0????????????????????????0???00????????????????????0012?01?
??100?0?0???0-
?2???????1??????0????????????????0??????0????????????????0???10??0?????????????????1?????????0?????
??0????00??????????????110???
1???????????????
Majungasaurus ?0001-11000-01101100000000-0110000001000101102 11-101100100-
0011010000010100110111{0 1}00000001110111001010000000(0 1)000111001?{0 1}?0000001{0
1}0?00?????010110012200110?----2{2 3}???-????-11100010010?????????????????{0
1}?00?100210100010100000?0????10010?0000000001(0 1)01102100001 1000---000??0{0
1}?00??10????00000200000000000000010000?000??010100000??1-1-0010100000(0 1)?0100-110000-00010100(0
1)11001001000-100000010?--0?001?00?0-12100011?00100000001?00100001101000--
0????00012100?000000100(0 1)10000000011-000-1012--???????00?0010?100-020010 0101?0-
10011101 1101??0000-01000100?1?10000000?00?000011-0000-0000000----010001100000?0????0??000-000000-
000001-0(0 1)1000-001101000--101-00000-00000???-?0000??0?01001000?0?{0 1}--?011--?110?-01100-
1010100?0?0
Dahalokely
??
??0??1?0???01000111(0 1)(0
1)??

???????????????????????????????????01???
??????????????????????????????1?????????????????????201??
????????0??????????????????????????????????????000000(0
1)??1????0????????????
??????????????????????????????0??010???
??
Rahiolisaurus
??????????????????????????????00??00????1???
?0?0?10???0?-0????{0 1}??200???0????????????????0?01?002?0?????????????????????101000?0010?2?{0
1}????00011?100110001002?????1?01010?0?0????1?0{0 1 2}????????????-
?????????????1??????00000????????????0?????????02?0?0?0??0?00011????0????0??101???????????0???????
???????????????????1????20?1-
????????????????0??0?100???11?????????11????0???????000???????????0???1?001???????00?????????0{0
1}0?????????????010--?-
?10100?0000??0??????00??0???????0????0201?????0???0?????1???0???0??0???????????????????????????
???0???????????????????????-?0??1?-??-0?1?011?0??00???????????????????????10?-?-
00?0????0??0?????0???-0?000-?????????????????0??2??01
MCF_PVPH_237
??
??0???????????????11{0 1}??{1 2}?0???{0 1}1?{0 1}01?{0
1}?1{0 1}1?1{0 1}0{0 2}?{0 1
2}0?0??120??1?????????????????????000??????????????????
??????????????????????00???0??????????0??1??????????????????
??????{0
1}???0??0?{0
1}?????0????????????????????????0???????0??????0?????????????????????0?????????????????????????????
????????????010??1?1?0?000??0??????
???
Xenotarsosaurus
??
??????????????00??{1
2}??010000100 2?0
1001?01?????????????????????????????????1???????????0???????????0????????????????????????2??????????????
?????????????????11??00??????????????
???????????????????????????????????01?001?1??000000000100?0
??0?0???????
???0010?0???000????????????????
???????????0?????1
Ilokelesia
????????0???0?????0????1?????????????????????????????????????
?????1?00?0?11??11{0
1}???????0?????????0???
?????????????????????????????0??????????1??2??????????????????????{0 1 2}??0??{1
2}0??0??1?????????????????????????1?1????????
????????????????????????????0???????????????0????000????????????????????????????????????0????????????????
??00????????????????????????
?????0??0????????0?0??010???0???
???1????????
Ekrixinatosaurus ???00-1?????????????0????????????????????????????10-
??11??????????????????????000?1???????????00?1?????0?????0????11????{1 2}????????0?????{0
1}???0???????????????????????????????????????011??0?{0 1}???0???0?????0?1?1?010?0?1?0???1001011?{0
1}??{0 1
2}?0?????0???0???000???1?000?20??????????????000????????????????0???????0?000???????????0??1?????00?
??{0 1}??0{0 1}?-1-00?0?????0???10?-??????-0??1???00?????????????????????????????1?0?1??????{1
2}1???0???????????00??0???????????????????0?????00??1???0?00???0??????????????????????????????0?????0?
0??0?{0 1}1???2??????????????????????????????-??1???0???0????01???????????10??0?????????00----?100{0 1
2}??????0???10??-
???0????0???0020??0??????????-??????????????????01??000
Skorpiovenator ?0001-1100?????????0?????????0?????1101?001??{1 2}0???-
?0??00??0?????0?????????00?????10??00??00?11?????????1??????????????????????{0
1}?????????00???????????????????{0 1}??????????????????????011??0?????????{0 1}0?????0???{0 1}??{0
1}??0?1?0???1010001?{0
1}??0????????00001000??????100???20?????????????0?0??????????????????????1????1????0?????????00?0?????
0-0100??0{0 1}1-1-00{1 2}011?10????200-?10001-?0?001?????0???????0???????????????{0 1}{0 1}0?{1
2}??????????????1????????0?0?0?00????????????????????????0???1?????00???0??????????????????????????????
?????0?00?0?100?020?11?????????????????????1??????-0?100???-?0?0??0?00??????????????0?????????0?----
0???{0
1}?????????0???1?????????00????0????
??????????????0??????????00??0?1
Viavenator ????????0??111100?00000100-?????????????????????10-
001100?????00000?????????????????????????????0??101???0??1?001110(0 1)00111(0 1)0?????00??00{0 1}0-
????????0?11001???00?????????????????2???????????????????????
????????0000?01?0?11?2???000???{0
1}0?00?0?????????????????????????????????????0?1(0 1)?0???????????-
0?0?????1010?10110???0?0?????00???000011??????????????????????2?00?
??00011011??1??????????????????????????????????????1????01?2????????1?????10????????????????0(0

1)????00???????????????00??10-00??????????????0100?0100??0?0??????????-010000-
01000???010???0??????????0???
?
Carnotaurus ?000101100?-?1101?00?0?1?0-1000?01001?0011110211-?01100?00-
001000????010000100?101000000?11???11001010000011?01120(0 1)200?0{0
1}0???????00??00??00110012200110?----220??11???0011100000-
2011002010001?1?01000110???0???????????????????0010?000000001?01002??000?000?---
0000??????????0??1?0?1021011000000?10??000010?00?0000?00001?1100020110000(0 1)?03000010001-
000?0100(0 1){0 1}10?1111-00-?0000?00???-11??????????0?00?11?00?0?0?000?0000??100?11000--0-
??0?0012?00?0?0000100(0 1)1?0001000110000?1010--0?100?01?00????01??-02??000????0-
0?00?0??01?1?1????-0(0 1)110000-0010000000?0??0{0 1}0010??000-???0?00----010020100000?0-0??0?0?1?-
010000-01-0010011100-001100100--{0 1}0{0 1}-0000?-0{0 1}?100?--0010?0?001????????????????????010?-
01?????0????2?00?
Aucasaurus ?000??1?????????????????????{0 1}??0?????????????????0-
??11?????????0?????????????????0??????????0??1??????????????????????????????0??????{0
1}0??0??????00??0??210?21??----3{0 1 2}?????????011?00???????2?{0 1}???{0
1}?0???????????????0???1????11(0 1)0000?000101000???0?100???{0 1}00???2(0
1)??00?0?0?1??0?0?0?0?00002???????0?????????????0?0?0???????0?{0
1}???00????0?0???1??0?0?????????01?0??1?00?-0??????????0?1??1?00-
?0?00??0????1?0?1??0?0?2?00001???01?????0??0???????0??110{0 1}0--
??????00?????????0??????????10??0?00?-0?0??0?{0 1}???-
???????0?00?????0??2????0???????????????????0?0?{1 2}?-??10????-
1???0???00??????????????????????????????1??{0 1 2}??????0????????????-00????-0??0?0-
????????0?11??00????0-00{0 1}??-0?????-???????????????????0001--0011-0???????0????????1????0?1
Abelisaurus ?0001-1100?-0?1?1?0010??00-0?10??00????010?1?{1 2}10-
?01100?00??01000???????????????????????0000?{0 1}(0
1)????0???
??0010100000000??0???????????????????????????????????????
??????1??????0?1?????????????00???{0 1}?????-?-00?0??0001????00-111000-
000???0????????001????????????????????????2?00?
??0???-12???0???1??????1?1????????????-
0?100??????1??{1 2}??00????0{0 2}??10??000-
???0????????01??
??????????????????????????111??0?????????????????
Noasaurus ???0?-
0?????????????????????????00???????????????????????????00?0?????????????????????????????????00?{0 1}(0
1)????0???1?00?1111?????????????????????????1????????????????????????????????-
0??1?0??00??????????0???????????0???1?????????????
??00??????{0 2}00???1?????-?-00?0???????1??{1 2}00-
?????0??????????2??????????????????????????0????????1?????{2
3}?????????????????0???01??
?????{0 1}????????????????????????????0????????-???0??0?????????????????????????????00-
00?0??????????0???
?????????????--??{0 1}??????????????????????????01??
Velocisaurus
???
???
??????{0
1}000?1????????????00000000?????2???11000??11????????????????0?
????0????????????0??0??????????{1
2}1??1?0111???3?0?????????1?????????
?????????????????????0??001??00???0??0????????????????
??????0????????0010???
??0??0??0000??1--??00--
????1???????????00??????0
Masiakasaurus ???000000??-111?1?00?0?11????101?0????????????{0
1}10000??????0?000?????????0?12010?0?0???00?0(0 1)(0 1){0 1}10(0 1)22(0 1)0101100001(0 1)000111?(0 1)11{0
1}0000?111{0 1}??0?????????0010002100???????????0???1?????1?000000??021{0 1}02?10001{0
1}1200100011002?010011111000{0 1}0?????{1 2}00?????00??10?1(0 1)020?20??00010?????00000???0???{1
2}1???1????????1?0000?11?1010000000?00{0 2}00?{0
1}01?00211?100?0?000?01???000???????1???1??00211??200?-1????????????????1?10011????{2
3}2000?????01????00???0010?0????????????0?0?1?001210??01000010001??00000011-1???1021--0-
10000000000100?00-?000??0????0-0000??010101???0?0-1(0 1)0???00??0???001??????????????????????0?0?00---
-0?00{0 1 2}110?????0???????????-00??10-01-0?1-1(0 1)1000-001100000????????????????000---
0001000001?01?0000?01--?0{0 1}{0 1}--??10???????????10012?100
Elaphrosaurus
???
??0?0??-1000-00011000100000000?0{0 1}{0 1}????????????0010002100??????0?????????????011000000-
20110020{1 2}000??11001100110021010011010000000?????2???????????????????02(0
1)0?0???0010?????000110?????????????????????0??00???0?0?01?0???10?10???????01?11????????????????1????
???????????????????221??10??-1???0???????????01100100???21000?1???01????(0
1)0??0???01??????????????0??01?001?0???????10000001??011100?100???1010--
001?000010000100?00????????0???0???0????001??????0????(0
1)????10??0???????????????????????????????????????00?010??0??????????????-?00?1?-01-??10011000-
001100000??????0???0??{0 1}?0000--1001?0000010010000??1--?00{0 1}--??????????????100?2???0

Afromimus
???
???????????????????????????0???11??
?????????????02??00?1101??????????0??1?0?????????????????
???????????????????????????????????1???
??001?1????????????????????????????????????
????????00?010??00??????????????????????????1??????????????0????????????????????????????????????
??0???00
0?????????????????????????1??1?????
Limusaurus ?111001010?????0?????0?????01000001?1?000?10000-
0?0110??00?1?10010?00?10120200?00{0 1}?1?1-121{0 1}--1110?-
01??100010?01?1?01000?00?02110?001?10?010000002000000-----02?-???--
??21000010001011001010001012 0?10{0 1}001002?01?00?0100000001000{1 2}0031-000??????1-030?0{0
1}??0001?00---00000?100000{0 1}????1?0-1?200?002000?0100000001001-{0 2}00-011010{1 2}1000000000-
00???03000002?01?00??0?012??001102010--0000001--?-011?01100002{1 2}000010?(0 1)010???1(0
1)000?01????0?????0--0????-00????0?000(0 1)???????010??0?011-00000010----000?0??0?000?01???100-100-
??????00????110?1010?10?01-00?0?-00?0(0
1)00000????????0???0???????????????000????????0?????????0??-00??00-0000?1??11?0??1?0110???--?10-
0001?-000000---10??0?00???????????0??--0011-?????0-0???????????0-100
Deltadromeus
???
??????????????????????????0??1?1{0 1}0-0?????????100000?0000?0????????????????????{0 1}???00??{0
1}11{0 1}??0?00?0?1???{0 1}000?1002?00??1101000000????01??????????????????????{0 1}??-001?{0
1}????00??0???000?????????????????00?0?0?0?????????????0??????????{1
2}1???11??-
??????????????????10?11???2??00???0?01????????????????????????????????000?????????????0????1{0
1}??????1?????????--?-
??1?0?00?00100??0????????????????0????0?????????12??????????0?????????????????????????????????????
???????????????0??????????-?0??0?-01-????0?10??????1?????--???????????????0?-?-
???00?1????????????0?{0 1}--??10-?0????-??????????0??2????1
Gualicho
???
??????????????????2???1(1 2)0?00010{0 1}{0 1}0?????????00010?100000????00?14(0 1)00-1100-
??????????????????????0101??10{0 1}0111?2?????????00000????001?????????????????03??21???000001---
?????010?0??10???1?01??00?????0?0???0?????0??0?????????????1????????????????????????????????????
???0?11?1-10100?10001001-???011????-{1 2}?000?1???01?????(0
1)?0????00???00?010?0??00??000?????????????????110000011-
0000??????????0??0?00?0?0?0??0??????????????????????1?????1?10??????????0?0???1???????????????????
???????????????????10???0?0??????????-?0??0?-01-001-1?1000-000000100??000-
?10000?00?????????????00?{0 1}???????????1--??0{0 1}--????0??????????0??20?0?
Angeac_taxon
???2???????????????-????--
??????0????10???0??????2???????00?
???????????????001???0??{0 1}00{0
1}0?????1???0???????0????????????????2????0??????-
?????????????????1??????????????1????????????????????-2????????????????????????????0011????{2
3}????????????????0???????????????????????????000??????-
?1???????????????????????????????????????000???0??0??????????????????????0????????00????????0??????
???????????????????????????????????0??
??????????????????????????????000???????00-????????????????????????
Chuandongocoelurus
???
??????????????????{0 1}??0??????1{0
1}????0{0 2}????{0
1}?????010?0?100200?0?1100000?0??????1??????????????????????2???????0??????00??????0??2??????????
????1{0
1}????????0??????????????????1011???0?
100??????200????????1?????0?????????????????????????1?00??????????????????????????????????????10??0?0
?000000?00?00?0????????0???????0????001??????0??????????????0???????????????????????????????????????
??000???0?????0?001??20000?
?{0 1}--??1{0 1}-??????????????????0?????0
Monolophosaurus
?001000100????1???0?00?10??00000?0100000100101110?101010000010000??00001100100010000000000101?11
110001100000010011110(0 1)0000??0???????00?????????????????????????????????????01{0
1}00000001010001 01{0 1}00?{0 1}110???????????????????????????????0020-020100001 1?0(1
2)00????????????????0000????????????????00??01(0 1)?0?0?1???00000100?1-
?1012?1?10??01100101101000??100011000000000000000(0 1)0(0 1)??????????????????????{0
1}0???????????1?????0????1?00000??000??????????????0????????0?00000000000?01????????????????1010----
00?????????????1???11001001?0001?0?000?01100??????00(0 1)-00?0?-?010?00000??0?00{0 1}?0???000-
0??0?0?----00000010000???000---
00?0?1?????????????????????????01???0??????????????00?
Pandoravenator
???
????????????????????????????????21???????????????0??1??
???????????100{0 1}001000100000000?0????{0
1}????????????????????????????0?0?????????????????0?????????????????????0?0?0??????????????????????

1???0???????????????????01????????0???1??0??????????
????????????????????????1????????????????????????0????????????????????????0??00???????????????
???????????????10??-
?????????0???????????????01?1??????????????????????????????00?{0 1}1001000?000--??{0 1}1-
????????????????11001????1
Piatnitzkysaurus ???00011???-0?11100000?1?0-????00????????????{0 1}00-
0?0????????01000?????0?????0??????????00?01?1{0 1}110??11000000(0 1)101110(0
1)010???0????????????????00000?121011???????????????????1{0 1}00??{0 1}?-2011102?11010{0 1}?1??{0
1}0001?001??0????0?00000??????10???????0?????{0
1}1010?0???00010?????000100???????????0???????0?0{0 1}00?0101?00?0?10??000?0???{0
1}?101111000?0?????10?0{1 2}??0???0?0-??00???0(1
2)01??100001 0??????????????110?0?00???21?10?????00????00??????0??0??0?0?????????1?0?12?0??0?00?0?
0??0?01?100?00?????1?11??0-001?00??0?0?0010?-?1????0??0???100???02100????01?-
0??0??00?10????000?????00????-0???????????---??000{0 1 2}010?????????????????-00???0-
0100?00???00???0??0??01??{0 1}?????????????000--?10????10?0?????00???{0 1}--??01-
???10???0???????0??1?00?
Condorraptor
???0??101?
??0????0?00?0(0 1)(0 1)0011???00?0?0??0???{0 1}????0-
2011{0 1}0??{0 1}10{0 1}??1?0????0?10???0???????00???0?????????????????????0?1?0?{0
1}???????????????0100??0????0???????????????0????????00?0????????????????10????????????????0???????
???????????????1?1????????????????????????1?0{0 1}??????{1
2}1???????????????00???????????????????????????001???????00000??????????????????????11--?-
0?????100?????????????????????????0?????{0
1}1?????????0?0?????00??0??00?010???0????????????????????????
???????????????????????????????????000---1????00000???????????--????-????????????????????????
Marshosaurus ???00?1????????0?10??0???0-{1
2}??010???0??0????????????0???0???000?00?????0?100?1??????0?0000101?1?1100?11?0?0001?0?11?00????
???00??0?10012{0
1}0??11001112 0?????????2??????????????????????0020??????????11?11?????????????????0000???????????
?1???????010{0 1}0?????1?00?0010??0??001?0{0 1}?11??1?000{1 2}0??0????????0?000???????00?1?0?0(0
1)?????{0 1}?????????????????{0
1}1???????????1?????1???????????????0????????????????0???????????????0?00?????????0????????1??????011??
?-?0?????????????{0 1}?????0??000?????10?????1100???????-
0110??0???0???0000?????????0??????????????0---
???0??0?0??000--
?0???0?????????????????????????????1???????????????????10?
Poekilopleuron
??
??????????????????????????0??10100??1?0?????0?????1100110000001?{0
1}???0??????????????????????????????????????020?10000000?????0??00{0 1}??????????????????????{0
1}?????{0 1}?0???????????{1 2}0?0??{1
2}0????00???0??????????????0?????????0??????????????1??{1
2}?0001??????????????????????0???0100???????????????????0??01001 0100??????1????1?????????????????{1
2}???0001011????????????????????????0??00????????????????????????????1???????????????000???1?????????
??????????????????????????????????0?0???????????????????????0??????1?0????1001100--
001???00????????????????????????000?000???????{0 1}{0 1}??????0-???????????10?????
Duriavenator ???00?1????????????????????{0
1}0??0???????????????????????????????????????0??1??00???0???0?00101012 11?0???????????????????????
??
??????????002?1?{1 2}???????{0
1}0???0???0?????????0?00???????10??0{0
1}????????00????0???????000?????0????1???0??
???????????????????????????????????000??0?????00?????
???????????????00??????-0?10??????????0000????????????????????????0---
??
???????????????????????????????????
Megalosaurus
???000?1?0??????????????????????00??00??0??????????????????????????????????0??1??0?????1?0??00101012
110??1?0???0??1?011??0000?{0 1}0????????????????00000011?01???????????????????010000000-202100{0
2}?{0 1}10{0 1 2}?{0 1 2}110010001100??0100??0?000000?????10???????????????10?1?(0 1)2{0
1}??00010?????000010??0??????????????00??0?0000?0????00000110?0?010???{0
1}?1?110?000?0????0???01??0??{0 1}?0?????1?0?01{0 1}1??1001-00???????????????0{0 1}?0?100???11?{1
2}???1??00???000??0???000001?000???0???1?001?????0000??????1?110??1?01??????010--
?0?10?00100???00000????????0?1?????(0 1)0???011?0????00?-0??00?00??0?0{0
1}?000?????????????????????????0?0---???0???10??00?????????????-000000-0(0 1)-
00??1?100??10???0??0??00?????????????00?0--?0?0?01000?0???000??0--?011--????????????????????2?0??
Torvosaurus ???0000110?????????????????000010??011?01?100?????0????000-
0??????????0??1??0????????0000{0 1}01012100??1100?0001100121(0 1)0210?0011??{0
1}0??????????00000121001101?00?1{0 1 2}00??1?0??01?000?00-
202100201100010000000011001001000000000000010????10011-?????????{0 1}0?11(0 1)(0 1
2)1??0001000?1?000001??0????????(0 1)001??000?0000?0?0?0000001(0 1)0?1-010??0{0 1}?101101000{0
1}00?000?0?0{0 1}000??00?0??0?1??001110?100{0 1}-00?10000???00??0(0
1)001100???01?10011?100???01????00??101101010??0?001?000?0???0?00(0 1)000001?111000011-000?1010--
0-000000100001001 00?0{1 2}0???(0 1)???00-100???0(1 2)110????020-00-

00?00?00?0000000?????????????????0?0??1---?0?00?010??00?0??????????-000001-01-??0-111100-101001100--111?01000?00?000---00?00011000000000??0--?011--?????-0???????100120001

Wiehenvenator ???00-1?1???????????????????{0 1}000??010???0101{0 1}??????0????????????????????0??{1 2}??0????????0000{0 1}01012?1?????????????????????????00?1??????????????????????????????????{0 1 2}??010?100?000????????????001???{1 2}0??????{0 1}0????2{0 1}??{0 1}????????0?00????????0??01????1-1-00?0??00?????{0 1}?0-?????0????????0??00???????????????????????????0?0??0-1??0?0?1?0?????????????????????????????-0?00???0??????0??0?????????????????????????????0??????????0?0???{0 1}0????????????????????0?????????0???0??

Dubreuillosaurus ???00011N0?-??1?10?0???0?0-0??0000??1?0N1??00110-00NN01??0???000010???0??10{1 2}1???0????000010101201?0????????011?0?1????{0 1}00{0 1}??110???0??????????N??0???????????????0?0?1??0?????????????????????????????{0 1}0????0?1?????????????????????002???{1 2}0000??010?1??2{0 1}??????????????????????????????????????0??{0 1}0??0??0??0?00???????10?{0 1}01?????1000010?10001???1000??{1 2}000000010??01???????{0 1}???????????????0??????????????0????1??????????00??01?????????????????????????0??0?0???0??????????????????????????????0??0????0???{0 1}?0-01001????????????0??????0???????-0?000?00??????NN00?????0???????0????0???0?----0N???0?0???0?0??????????-???0???????????0???????????1????0???????0?????0??

Afrovenator ???0001110?????????????????0?????01?1??01?100?????0??00000-0??????????????????0????????00?0101??10??11?0??00110?1100?????0??1?1{0 1}0-0??????????????21??????000?0{0 1 2}(0 1)00?11000?1??00?{0 1}?-2021000?1{1 2}001{1 2}?1??0000???01??10???0000??{0 1}?0?000?0??????0???0???00{1 2}???1??????1?{0 1}1??000000????0?0{0 1}????1?01?????00{0 1}?????0??00?0?10??00?10???1?1?1111100101??00?0??1000??0000??0??????10?0????????0????????000?0{0 1}0?{0 1}????00{1 2}10????10??0????0??0??00????????????0?0???1?0?????????000??0?????????????1????01011???-?00?0????0???00???01????0????0-10?????110????????-0??00?0???0?0???????????????????0???00?0???????0??0?010?????0????????????????????????????????0?????0????????????0??????0?000--?0?????1??????????????{0 1}--0???-?0?????0?????????0??0001

Leshansaurus ???????1????0?11??0000?????????00????????????110-0?00???????11000?????????????????????0??00?{0 1}1????1???1?00??00110?1{0 1}??000??????????????????????????????????????0??{0 1 2}00??110??1{0 1}1???????0??????????11{0 1}?0??{0 1}0?0????1???0???0??0??????????????????????000?1??00(1 2 3)?0?1??????0????00000??{1 2}?????00????????????????{0 1}??????0??0?0????0???10???1?10{1 2}1?0??0????????1???{0 1}0??0??0?000?0?????1{0 1}1???????????????????0??0????0?1???????00??????1??????0????0?????????????????????0????0??00???000000?????????????????0???{0 1}{0 1}????001?????00??00{0 1}0???{1 2}???????0??????1???11?0?????????????0??0???0??????????????00??0?-0??????????????????000{0 1}010??0??0???????0000???0??????????????????0???--????-??1?????0????????????0?0?

Eustreptospondylus ???00011N??-0?11100000?1?0-0?000?0??????10100100-?0N?00?00-0?1000?????0?11?{0 1}0????????000010101211000100000001100 11?000000000???{0 1}????????????0?N??02100????????????????????111001000-202100??1{0 1}0012010010001100100100 10NNNN0000???0?10020??{1 2}000?00?100(1 2)000(0 1)?????10?????000000100???{0 1}0???0????????000{0 1}0??01000000001000??010??01?111101000{1 2}0??00?1(0 1)??1000???0?0000010??0111????????0???????????????01001100???11010??????0????N0??00000????????????0?001?000200??0?00000000??110000001?0???1010--?-00000010000100?1?-01????0???00-00????01100????000-00?0??00??0???NN00????????????-00???00?0??0---?0N00{0 1}010?N00?????????????-??????????1?1-101000-001011100????????????????NNN---0N?000000000000000??0--?011-1?0101??????????000??001

Cristatusaurus ?0?0?-1?????????????????????001010??????????????0???????????0010???????????{0 1}??0??1????????00003010?21100???0????0110?11??0????0????0{0 1}0-0???????1?0000???10?2??????0??{0 1 2 3}0000(0 1)1000110??0?{0 1}???01?100?1?0102?1??10?01??01??10??100?????????????0031??2????????{0 1}?01(0 1)?20??00?1?0????000?0??????????????0????????0?0{0 1}0??????000??0?0??00?11??01?1111-1-00{1 2}0??0???1?0??0-???0?0????0??00{1 2}01001001?00????????????00?1100??????0?1?20???000?????00?010??????0?????0?1??0?0???000??????0?(0 1)00??????10010?1??????1?0?????1???-?0??????0000???0?0???0???0????10??0????111????????-?000?00???0??0{1 2}?0?0????????????????????0?0??????????????00??????0?0????????000-0??????-00-1?0??01?0??0?10011????{0 1}?{0 1}?0?????????00?--?0??0??1????????????0?????????????0????????101000????2001?

Baryonyx ???????????-??1110000000?0-00101?????????11100010-??1????00000?001??????1?100{1 2}1???00?1?00003010121100?110(0 1)000(0 1)1(0 1)001100?????00??00????????1????0000?1110?210??00?0{0 1 2 3}000?(0 1)100????0?1?01??011{0 1}?0?{0 1}?0{0 1 2}?{1 2}??01??0??1001????????0??????????????0030?02??00??010011??1??0?0110????00000????????????????01??{0 1}00?0{0 1}01101?0000?00?0??00?11?{0 1 2}0??1??1???????0??0??0(0 1)?010??10?0?0?0?00?100101??{0 1}0?{0 1}000?????000??00?1?????????????1?10????????1??00010???00?01?01000??0???1????2?01?0?(0 1)00010000?010000010-1?????11000-0?0100???00???0?0-?100?0???1?0010?????111?0?1?????(0

1)0?0??00?001?020010????0?0?????????00?0?00----?100?010?????0-????0????-?0??00-00-1?0-110000-
001000101--11{0 1}???00????0000--0???0?11000????????????????????111?-????????1???20???
Ichthyovenator
??
??0?????????????01{0 1}?0{0 1}?{0
1}???0???011000?011001100101101110101????????????????
???????????????????????????????11?1???????????????0000?????????????0??????????0?????????1?0??100?1
-
????????11???00?????????????????????11??????????1?????????
?????0???????????????????0?0???????????????????000????????????????01000--
00??????????????????????0???0???0?????11????????????0?????0??0??????????????????????????????????
??????010???0???000--
000?000???0?
Irritator ?00???11?0?-0111100?00?1?0-
0?101?0101?00000001?00?001?0?0???0?101??0???1???{0 1}?0???{0 1}0?00002?0--
1???
???{2 3}1?0?000??-0{0 1}-
??-?0??1?1????????110{1 2}?1??????1?00{0
1}00?000?????0??0000101000??1???1????1?00??0??100????
???????????????????1?000??1???{0 1}??-
01???0???1??????01?????1????????(0 1)??00??????1???0??0????0{1 2}000?-
00???????0??????00??
????????????????????????????111??00????????????????
Spinosaurus_sensu_lato ?0000-1??????????????????????011010????????????0????????????0?(0
1)0??????????0?110?0???0????000210--122000?100?0?0(0 1)11111100??0?000??00{0
1}0?0?????????????????1?????????????0???1????011100?0(0 1)-(0 1
2)0010???0?0110?0??1?001?00??0110??00??????0?000??031?00????0??1-021??1??????(0
1)?????000001?0?0?000???0???????(0 1)?000?????????1{0 1}(0 1)0?1001-{0 2}111?11?12{0 1}1-1-
0?10?00???0??{0 1}00-01?????????1??00(1 2)01???????0????????0??1??1110{0 1}0??10?{1 2}10{1
2}?0??????1???00??0???????????????????0?????000?????01(0 1)00010000??????0??1??????012--?-
?0????100?0?0?000???(0 1)0?000???00?0?????(1 2)(0 1)???0??????1(0
1)00???00??01??2000???0??????????????0000?00----
??0??010??00?0?????????????????????????????0?????????????????????????????000?--
00?0???1010??????00?????0?1{0 1}??????1?????????-????0?0?
Kaijiangosaurus
?????????0?????????????????????????000???00?01???
????????0??0?(0 1)1????????????????01????????????101000?11002?0??00?{1 2 3}{0 1
2}00??100??00000????011?????????????????0?2????{0
1}??0001???????????????0?0??2???????0???????????1?0?0?0???0??????????????????????????????????????(0
1)?0???????1??????????????1????100{0
1}000????00????00????0??0???01?010??1??00????0??0????00?00?01?0????????????1????????00????????11?
?0??10?0?0??????????????????10?????????0??????????????????0??????????0?0?0??????????????0???{0
1}??????????????????????????????????0?0????????????????????????-????0?-0101?0???100??{0 1}?10001??--
????00???????????????????????????????????{0 1}--??11-??????-??????????????10???
Erectopus
??00?0101?
?1??????????????????????{0 1}??????0????????????????????????????????0?0???{0 1 2}?????{0
1}0????????????????????????????????01000?100??0?00??0000000????????????????????????00?????????????0???
??0?????????????????????0???0????{0 1}????????????0????????0?0????0??21?????????????????{0
1}??????????????????????????????????0????0{0 1}?0??????010?????{1
2}?0??????????????????0????????????????0??0???1000?????????0??????????????????????1?0????????????0??010?
??100?0???????????????????????0?????????0???0??
???0?00????0?0???????????1000?????000???--?0??--
????????????????0???????
Xuanhanosaurus
??
??0?????????????10?11??????????????????????????10100?1????{0 1}???00000{0 1}?0???{0
1}0???1??????001{0
1}?111???????????????????????00??00?????0?0???0????????????????????0?????????????????????0????????????
?????????{0
1}1??100?001001000?00????????????????0???10?0??00???????????????????1??0?000???????????????????????0
?????1?00?010?000???0???1?????
??????????????????????????????????010??????????????????-01??00-??00?1-?1?????001?00100??{0 1}?{0
1}?0000100{0 1}0??10???
Yangchuanosaurus ?001011110??????????????????10?0??0000?001?1101??-
?0??000?0???????????0000010200010?0000000101??11??00100000000(0 1)0011000000000??{1 2}1{0
1}0?00????????0?0???(1 2)1?01?00000?10?0???{1 2}???010001100-2(0 1)1(0 1)(0 1)0(0 1)0100110110?10001?00{1
2}??1001000?0000?0?0???00000000??00??1?0(1 2)(0 1)0110??0?1001???0000(0 1)?????????????(0
1)?001?0000(0 1)00?(0 1)????00000?100?(0 1)0?101(0 1)01010(1 2)111100101000010??10(0 1)0011000-
?0??011010(0 1)0?1??(0 1)?100?0000?0??????0(1 2)0?2????????10(1
2)0?01?00?000000000?000??0??0?0?1??0???1?000?1?0?0?0000000?0?000?001010100?1011--
00000?00?0000?00100?110?1101?????000????111?1??????000100?0?-00?0100000????????0???0?0??????00---
-0?0000100000?0???????????-???????0??0?1??01?00-{0 1}?00001??--1???0000??0{0 1}00000--
0010?01010?0???0?0???--????-??????-0?????????0???001
Shidaisaurus ??????????????1??????????????????????????????{0 1}?0-
??01????????01000?????????????????????????0??1?????????10???????????1{0

1}??0?0????0???0100?0???-201000{0 1}?1?010{0
1}?1???????????????????????????????????????00?????001(0
1)??????????????????001????????????0?????????1?????????0??0???0?1?????????1???????????????????{0
1}?????????-
0?0??????01???????????????????????11??????????????????????????0???????????????????0???????????0?
??0??0????0????????????????0?1???000????????????????1????0??????00?????110??????????0????????0
???????????????????????????????????0?0{0 1
2}0?0??0??0??0000-
?00?0?????????????????????????????????0??????????????0?
Metriacanthosaurus
??
??0?????????0??0011?0??0??{0 1}???0??{0 1}?-2011{0
1}???100{0 1}0{0 1
2}1???0000??????0?????????????????????????????????????01?????????????????0?0?0?????????????0???????
??0{0 1}????????0????1??????????????1{0 1}{1
2}?????????????????????????????????????01??????????????????????02?0????????1??????????????00???
??????????????????0?1?000??????????????????????????????????10???-
?00?0?000????0??????????0???????0?????11???????????0????0???0??????????????????????????????????
???????10???00?--
??????01???0?
Sinraptor_dongi ?0010111000-0?101000001001011000000000100101100 10-100100000-
001000100000001101101 10{0 1}0000000101011110001000000(0 1)1(0 1)0(0
1)110000000?0???????00??20??0??0??0???????0?????{0 1}?????{0 1}??1110000000-
2020000010001111000000010020010001000000000?0001001000001000000100 11?(1 2)????0?{0
1}0?????000011100?00(0
1)0???1???100000000?00100?000001000000001101011111100010110001 0??1000001000-0000010010(0
1)??10?1?????0000??0????1200100000?110??0?1?1000000000000000??????????00001?0002100000000100
00?0????????????0?1010--1000000000000100 10?-1100110110000?0001001111101?00000(0 1)100?00-
?000?000000000000000-0000-0000000----000000100000?02?01000???-
??????????10????????????????????????01????00?0000--10?000100000010000000--0011-
??0110?00???????001??101
hepingensis ?001011100??1?1??00000?????{0 1}100??0000?101010001(0 1)-?001010?0-
?0?000???0??0100210?{1 2}0?0000000101?11{1 2}1000100000001(0 1)00110(0 1)00?0?000111{0 1}0-
00??????0?10000???????????????????????1100?0?01??01100011001101100 10?0010??????????????????????
?001100{0 1}0000???1?0(0 1)1??1??000?1?????00000????????????1???1???001{0
1}00000?0?0000?100?00?0010?1?1{0 1}1?11000101?000(0 1)0?0100?001000-0000010?(0
1)01??00010????????????????12???????????1?????10?0?0000000?00000?1??????????0??????????00?00000(0
1)(0 1)0001?1??????????????1{0 1}11101-000001???????01?0?120?11?1??000000?10?111?1??????00(0
1)100?00-?000?00000?000?00?0???00{0 1}-???0?00----000000100000????????????-01?00?-
0??00???????????????????????????????000--01?10?01000???????????????????01????00??????1???{0
1}??0?
Siamotyrannus
??
??0?????????????11{0 1}??000???001???0???????????????????????????????????????010000?{0
1}01002100?1{0
1}??101?1???0?0?0?????????????0001?????????????0??
???????1?????????0?0??100???????????11???
?????12??????????1???????????????0??0????????????????0???10
01--
1?01???????????????????????1??????????????11????????????????????0??????????????????????????????????
??????????0??0??0000--
0?10??0?
MNNGAD_Kryptops_postcran
??
????????????????01{1 2}??01{0
1}0??01001000?1102100??100100100?????????????????
????????????????????????????????0???????????????0001?????????????1?????????0???????????{0
1}??01??1?????????1{0
1}???????????????????????????????????????0??????????????????????10??????????0?????????????????
?????????????????0??????????????????00???01????????????????0{0
1}1???001??????????????????????0???????0?????11????????????0????????0???????????????????????????
???0100-
?0?1??0???0?
Allosaurus ?102001110000?1(0 1)(0 1)000000101?1110000101100101100 10-10010000000(0
1)10101000010110100010000000001010111001011000000(0 1)1(0 1)0(0 1)110(0 1)000000012100-0000---(0
1)000000011001010(0 1)0011000000100111000000 0-201000001101000000000011002001001(0
1)000000000?000100201010000 0011(0 1)00102{0 1}0000010021100000001100010(1
2)00??10010000001000000 1000100001000(0 1)0100110111111(0 1)1000100100010?0100000(1 2)000-000(0 1)(0
1)100(0 1)(0 1)10000000(0 1)0000(0 1)001000000110(0 1)11000001001000100000000(0 1)000(0
1)000001010000000000-0010000210000 10000000(0 1)100110000(0 1)00000010(0 1)1--0-01(0 1)00000000100100-(0
1)10011011000(0 1)1001011111100000(1 2)000(0 1)000100-?000100000000000000--0000-0000000----
000000100000?0----{0 1}--010-000000-000(0 1)00-111000-0010(0 1)110001001-010001000000---
001000101000000110000--0011-001101000100000(0 1)01010001
Saurophaganax
????????0???1?????????????????????????????????????{0
1}??1?????0????00?00011??11???0{0 1}???0?????0????????????0???1100?????00??{0 1 2}00??1?1?????{0

1}?????1{0 1}0??{0 1}?{0 1}????{0 1}0{0 1}?0??0000010?{1 2}?0100??0?00000?0?0?01?????????????????0???{0
1}???0?10???????0???{1 2}0?0??2??????0???00???{0
1}???0?0?0???0????0??????????1111??????????0?????????????????????????1????{0
1}????0???0?0?????0?????0??00???1{0 1}020???1?00????00?0??0?0????????{1
2}?????????000???????00?????????110000000?00???{0
1}1??0?001??000000?00??????????1???????0????111???1??020?0?????00????????????????????????????0?
0???????0?0???10????????????????-????????????{0 1}-??100??0?1001000??????0?00?????000?-
?????????????????00??0--0?11-?0???1?????????????????0?

Neovenator

?102001????????????????????010010??????????????????????????????????0?0??{0 1}0?0????????0000{1
2}01011100??110?0?1(0 1)1100121(0 1)?1{0 1}?000111110-
??????????01000???????????????????????????0?0000100200000100100100010001100 2?0000??0?000?00?????
10020001???????{0 1}0011?20??0?0?0?????0?000010001?(1
2)0???10??????0?0000?0????10000?100100101??{0 1}?11111110010??0???0??100001?0?0????0??10(0
1)01??10?00?????????????????001(0 1)100???10??????0?000???(0
1)0??????011??????????0?001?000?????000000 1001??0??????????????001--0-
0000000000100100???0??1?1??0??00???0?111??????0000(0 1)00??0????0??00000?????????????????????0-
--???000010?000?0??????????-???0?0-0100???????????????????????????????????000---???0010000?????0(0
1)00?0--?0?{0 1}-?0???0??????????1???1?00?

Acrocanthosaurus ?0010111000-11101?00000000-110010000000010100010-
?00101000110100010000001101101100000000010101220??0?1000?0(0 1)1100?2?1??1?00001??{0
1}0?0???????000(0 1)00(0 1)0101200000011{0 1}101?(0 1)100?????????????0110?00110{0 1}000(0
1)0000001100 1?0100??0000000?0?0??10020100000000010011021??00?010111????00?10?0?020???1?00110001
?000100001?0?0?1??00?0000001??121111001011000 10?01000001000-
0000010 0101??1000000001100?00000??00?1000?0?1?020011?00100000(0
1)00?0000?10001111100???01000?2?000000100 10?(0 1)1?11100101000000??????0-101000?0001100100-
020?11?101?001?0111???100??10??00(0 1)100?00?1000100000000?0?001--0000-00?000?----
010000100000?0??????????-00??00-0?0000-111000-{0 1}01001000--{0 1}0{0 1}-000010010000--?0????1100(0
1)???????00?{0 1}--0{0 1}11-??1110-00????????11?0?101

Labocania ????0????????????????????????????1??????????????{0 1}10-
1???????0?1???????????????1???????????0?00??1?1????1??????????????????????????????0???????????????
????????????????????????????????????1?1??{0 1}???????????????????????????????{0
1}???????????????????00?0???0??0???????????0?????1??
?????{0 1}??????1???????????1???{0 1}??????0?0-???0???{1
2}???????1???
??????????20??????????????0??????????????0?????????0???????????????????????????0????????0??????
??0?0?????????????????????????????
-????????0???????????????

Shaochilong ???00-1????-?11?1?0????200-{0 1}???1???????????????11-
1?01???000010?????????????????????????00?{0
1}1????0???110????0??????????????0??1??
??0???????001000?0?????{0
1}??0?1?????0????????0???1?????-1-00?0?????10???00-
0??0??-0?0?????{0
1}??0????????????????????????2??0???0????
???1?0-?2?????1???111????????1????????-
0?00???0????????????????????0-????{0
1}?00?0????????1??
??????????????????????????11????0???????1?????0??

Concavenator ?0000-110?????????????????????????00???0?1?000???-
?0???????0??????????????????1?????{1 2}????0??00?01???????01?00??0??{0 1}???{0 1}?00?{0 1}??0?01?100-
000?---??0?00??21??1????00?{0 1 2}{0 1 2}(0 1)?010{0 1}??0010000????{1 2}001{0 1}0??{1
2}?01???0?01??00??0???10001000000 0??????1002?00{0 1}???????{0 1}?01(0 1)??(0
1)??00?1?0???0000?0????00?2?0?0??00??????0??????0?11?0(0 1)??0????0101??1?11{0 1}?-1-
00100??0??0?0100-?10000-????????{0 1}010?100{0
1}?010???0??????1110??1?00??011100??10?0?000?00?0??0?0?10??0?0?0??0-
????????1??00??????0??0?01??1?0?0?1??0?0{0 1}1???-
??????????0?0?100?12????01??????00???021????001{1 2}?-0010??0?-
10?01??000000??????????????????????????01?000?0000??0----?--???-00?00?????1?0??11?0??{0
1}?10011????1?0?0?001????000--?0010???????00?0??000?--??11-?0?????0????????001??1?1

Kelmayisaurus

??0?110?1???????????001{0
1}???{1
2}10??
???1??
??????0????????0??0????????0???{0
1}????????0??????????????00????????????0???0??
?????????????????????????????????????000??
???00??????????????????????????0---
??
????????????????????????????????????

Carcharodontosaurus ?0000-1100????????0??????0-0???1?0000??010110210-
?101?????????????????????????????????00?0101??0????????0?01??????????????0???????????????????????
???????????????????????????????????0?00?{0 1}?????0{0 1 2}?0??10?01100{1
2}?????????????????????0???10?0010?0??0?????????????1????????0??????????????????????????????????1
0?(0 1)01???1??00???1???1?-1-00101??101???100-011000-0???????1??????????????????????????{0

1}0????????????????1????0???01????01?????????????????1?????1?0???0?????????????????????????????0
??01??1????10?11???12???0?1??????????1????1???????-
0?100?00??000????00?????????1???????????????????010???
????????????????????????0??-??????11??0??????????????????????1????0?????????????10?

iguidensis ????1?1????-1?111?0??0?0?0-{1 2}???????????????????0-
1?01????????0?10?????1?????1??????????00?010???0??
???{0 1
2}?????00??0??0??1???{0 1}?????0????00????????0???{0
1}????????0???????????????0???????-
0?00???0??0???????????????????????2?00?
0?0??1??-
?2?????????????????????????????0?1????????????00???????????0???????????{0 1}0---
??1???
????????????110???0?????????????0??

Tyrannotitan
???????1?????????????????????????0?00?00??????????????????????????????1??10?1???????????010101{
1 2}100??1?0????1110?12?10000??0??0?{0 1}???????????0010?0???????????????????????????????????{1
2}{0 1}000?{0 1}10??{0 1 2}?10010001?001??????????{0 1}0???????0???????????????100(0
1)10????0000?????????0???0?????????????????0??000?0?????0?(0
1)01??010???????????1????????1???0????1???????????0?0???0101??00??00???????????????00??0????????{
1
2}???1??0?????0?????????1????????????????????????0?0??01000??????????1?????????????01001???011
?0?????????????????????????????0??????00??0?00??0?0{0 1}?00????????????????????????0---
?0?0000?00?00????????????-?0????-
010?????????????????????????0??????????????????????0?0?0?0??????????????????????1?0???????????0???
?

Mapusaurus ??00?-11?0??????????????????1???1?00001?01?1{0 1}021?-
?1?????0?01??????????{1 2}?111?1???1?000??0010101??00??100??????1??12?0??0??001101{0
1}??????????0??0??1{0 1}{0 1}?1??????????0???0????200000?000{0
1}020000??10??????0100001?0201000??00000000?????10???00?0??????10?01?2{0 1}?????{0
1}0??????00?01???????????????????0?000??0???010?00?1??100001??{0 1}?0?11-1-00{1 2}0?1?00????{0 1}00-
01{1 2}000-???0?1???0???10???0???????????????0??????00??{1
2}1010??1??000??1?0?00??10?1???????0??0???1?001?????0?0???1?00???????01?1-1????{0 1}00--?-
??1?01?0000??0100??2???101???00010??1010?1????0??-00100??0???00??000?????????????????1???00----
0??0?0?????0?0????????????????0?01?00????1??????10?1000????0??????????00?---
???0??1110????1000??1--1?{0 1}?-0??????????????10????1?1

Giganotosaurus ?0000-110??0111?0?00???000-1????????????1?11?210-
0001???000101010?0???1?11??1????????0?00101??11001????????1110?12???????0?1???{0
1}0???????????01{0 1}?0???????????????????????010??0???-201?000?0{0 1}0100?1?0{0
1}0?011?02???????0?????????????00{0 1}?0??0000??0100{0 1}1??{0
1}????1???????010?0?????????????0???????0?0{0 1}00?01???10?0010??10??01??{0 1}?1101-1-
00?0???0?1???{0 1}?0-01?0?0-0?00???01000?????-???????????????100?????????{1
2}??????????0???00??0?01??????????????0???1?0??2?00?0?00????????????????????????01???-
?11?0?????????1??-?2????01???0????????111????????-0010??0???0???000??????????10??????00?0??0---
?01??????????????????????-????1?-01?0????????????????????????????????????000--
????0??1????????????????????????110???0?????????????10?

Datanglong
??
??0?????????????012??00000?00???01{0 1}0???10-
20???0?????{0
1}???1?00??????????????00????????????????????
????0?????????1?0????????????????1{1
2}??0?????????????????????????????????0????????????????0??
?????????????????0??1?01--
??????????????????????????0???????0??????1?????????????????0??0????????????????????????????????
??????????0??00???000?--
??10??

Gasosaurus
??
?????????????0???????00?0?????????????????????????1100???????????????????010{0 1}00?{0
1}?1201100000{1 2}000{0 1}?1??0??????0{1
2}???????0?00000?????????????????????????1?0???????10?????000?00???????????0?????????0{0
1}?????0?00?{0 1}???0??00{0 2}????????1{0
1}1??????????????????????????????????????1???????1??????????????01000?00???{0
1}1?11?????????????0??0????????????????0??1??001????????0??????0??1?10000000???0{0 1}?0??0-
001??0??000??1??????????0???????0????010??????0?????????????0????????????????????????????????
???0????0??00?????????????????????????10???100-{0 1}0?00?10????????????????000--
?10????????????????????--??1?-????????????????0?????0?

Lourinhanosaurus ???0?-
??0???????????????????????0?0{0
1}?????????????????101????{0 1}??0?00?0?01??{0 1}0?0??????????N??0???????????????????????0{0
1}000??0?120?1000?{0 1 2}??1??????1000100?2?0????????????????????????????????????0(1
2)??21?????????????000?0??????????????????????00???0????0????????00????????1011-?-
??????????0?????-???????????????1?10?1??????????????????{0
1}1?0?????????1????????0?????00???0???????????????0???1?00?????????0????????0???????????????11??
?-0?0?0?100001?0??????????0???????1?????11?????????-

??????0???0??????????????????????????????????????0???10??00?????????????????????????0?????????????
?????????????????????000--?0?????10?1???
Chilantaisaurus
??
??0100??????????00??0?0??1?{0 1}{0
1}?????10??????????????????0000?10???01?0??0?000001?????1???????????????????????????1????????0??
0??0????????????????????0?????0?0??0????????0?????????{1
2}1??1????????????1????001010???1??10??????0?????????
???????????????????0??000??????????????????1000000100?????{0 1}{0
1}??????1?0??0000??0?0????????0???????1????1????????110???
???1-101000-
001001100???????????????0???????????1000?????000??0--?011-0????????????????????0???
Fukuiraptor
????0??0?????1????????0?00?{0
1}101110?1??????????1????{0
1}?0?????????0??????????????0010?1101201???????000?0100?????????????????????????????110001100??0
0?0110000000?0?0??????????????????(0
1)01??????0??10??????????1??????????????????????000???1?0?0?00??????0????0????21???????????????
0{1
2}??????????????0???0?0???????010???????0??11????001??0??2?0100?????0???????0??????01?10?00?????
?01?010?1???000??????????10010?00-
0??????????????0100000????0??????00????????????????0????????0???0?????0??0???1?00?????????????????
????????0---???0???10?????????????????-000000-000??1-?11100-
001101100?000???????????0?????????????1001?00???0???1--001?--????????????????0?120??1
Australovenator
??0?000{0 1}0????????0??(0
1)1{0 1}101110?1???????????????????????????????????0????????????11001110001100000(0 1)1111???{0
1}??????????????????????????0101011002?00001000000000?0001???????????????10????????????1001??0?????(
0 1)1000?0(0 1)0?????01??000??00????0?000?00???0??0????0????21?????????????????{1
2}??????????????0???0???????????00110???1000000??010000000 2?01000????0???????0???????000000101???
?110000?????000??????????10000000-
00?????????????010000001?0?0????00?????????????????0?????0?00???0????????0???1?00??????????????????
????????0---????????????????0???????????????????????????0-101100-000001100000000?001000?{0
1}0?0??????????1000000000000?1--0011-0????11??????????0???0??1
Megaraptor ?001111????-
1?1?100????0?11010010?????????????11011?1????????0??00??????????????????????0101?{0
1}101??1?10110???0(0 1)100?121??1200000 1?????0???????0?1000011001?011000100000(0 1)1(0
1)00?????????????????????????0????00???02?????????00010?0????100{1 2}??02?0???0??-
?10?????0001?02110??????{1 2}0????????????01??00???0?1?01?10?0?001??0??0?10?010?1??10?0010??0??1(0
1)?0{1
2}00?0N?0?00??0???1?1110?00??0100001000000000?????110??01?01000??0000???01?0?0?????01?01010????
???000???00???000000??????0?000?100001??{0 1}?????0??????0??0???1??-
?100?0?0?????????1?10???1????0?000010??0??0???{1 2}N??00?????????????????????????????N00{0
1}0?0?N?0?0??????????-000?00-000????1010N0-00?00?100--{0 1}?{0
1}?0100000000?0???1?????????????????????--?00{0 1}-0?11?0-?0????????0??100??
Murusraptor ????????1??-11101000001100-?????????????0?120110-(0
1)010??00011100001?0?0?0??????11?{0 1}0000?00?0101???01?????????????0121(0 1)0?{1
2}?0?????????????0?????????????????????????{0 1 2}?????????010{0 1}00?{0 1}?10?1?{0
1}????101???????????????0??0?????00????????????????????0000100????1?2??????????????000??0??????????
?????00????1?{0 1}?0?1?0??0?0??1??1?????00??11?1????????????1?0???1?????????-0?0?011??{0
1}10???????????????????????10?1?????????0????????????0000?0?1001??????????????0?????0000?00???0??010
00??????????????????001????0?????000??????00-
?10???0?11?00?0?0???11??1???????00?????0??0??????????0??0?0010-0?00-
00?01??????00???0??????0?0???000???10?00???
???????000????????????010???0????????????????
Orkoraptor
????????1?????????????????????????????00???????????1????????????????????????????????0???????1??101?
???????????????????????????0?????0??
???????????????2?0??????????????????????????????????????-
????????????????????????0??????????????????????????????????0?0???????????????????????????????????1??????
??1?????????????????????????
??000????????0??000??????????{0
1}??????????????????????????????(0
1)??????0????????????????????????????0???
??
Aerosteon
????????1??????????????????????????????????????1????0?????0011????????????????????0?????????????????
??0??1?00??0(0
1)110012110?0????1??????0??????00?11000????????????????????????010100?0000??????????000?10??????
??02??0101?00?0??{0 1}????????????????0???1????00(0
1)????0000?????????000??????????????0????????01??0?0????0?0??100?????????????11?1????????????1??1?0{0
1
2}??????????????????????111?010010????????????????0????????????0????????00????01?01?01???????????????0?
????0?0?1????????0110?0000???????????????001--??00????????0??0??0???{0
1}????0????00?10????11????????{1

2}??00???00???0?????????????????????????00?0???????0?00?010???0?0???????010-00??00-
01000??????????????????????????????????000??-100000?????00?0??0???--????-?????????100-10??0?1??01
Kileskus
?1?3001??????????????????????1?1?1???1????00000?{0
1}101??1?1??0????????????????????????????
??????????????????????????????00?00?0?0???0010??{1
2}???????????????????????????????????0????10????????1?0?????????????0??????0???00??01?????10000?
???0??????{0
1}010?????0??????11????????????????0????0??????????????0??2????0?????????0???????????????????????
??????????????0???0????????0??
?0?10??????????0??0???
?????????????0???0??00???????????????????????????{1 2 3}--0???--????0??????????0????1??
Proceratosaurus ?10{1 2}0110100?0??1??0000?????1110100100?00?????{0
1}???????0?0?000?????1?00000000110?10?10000001010(0
1)021?1???
???????????????????????????????0??????00101?2?????0?1(0
1)???????????????????????????????????????00??0???0?0?1?0??0?00???????10?201?????10000?01?0?0???
??0101?2000?????0110???1????1000??0??0?????????????????
?????????0?0110??1???10??1???10?01???00??????0??????
?(0 1)?100??????10?0000?00000{0 1}00????000-?0?01?0---
?0??
??????????????1???0??????????????0??
Guanlong ?112011010?-
1110100000?10??01101?0100100001101100?01100000?00000010???0?000110?00???0000(0 1)?0101??1(0
1)100?0100000000110(0 1)00000?00?21???00??????0000{0 1}??010001000001101000001?11010001?00000210-
101101101(1 2)00101011002000011000000001001000010020?020001??0110(1 2)(0 1)?01??0??110111001000?(1
2)00000{1 2}0???1?0110010100{0 1}?0000111000010001-0111{0 1}0101111?0000{1
2}01?0001???10101010000000?0110(1 2)1(0 1)??{0 1}0?{0
1}?10101000100?100111011000020010001?00010??00001000???00000011(0 1)00?001000??100?0?0(0
1)0000001??10000000010001001----?0000000?00100110-00(0 1)01?11?0000?1?00?001??01?0010?0(0
1)000?00?0010100000??00000?0???00?-00?0??0---?000000100000????????????-??????????1?1-1?1000-
101000001--{0 1}0{0 1}-000000000000---101000001110000000000--00(0 1)1-??0??0-0????????0(0 1)??0101
Mirischia
??
????????????????11{0 1}?0?10?0???0?00?????10001{0
1}010{1 2}{0 1}?{0
1}10120?1000110?2?0??????????????????????????????????????0210?????????????????0001????????????1?????
?????0????????1?0??1???(0
1)0?????????01??????????????????????????????????????11???????????????????????0?0?????????0???????
???????????0???????????????????0???000??????????????000????????????????0?01??1-
100??0?0???1?0?????????????????????0?????01????????????1????????0???????????????????????????????????
???????100????0???000--
?00???0??1???0?
Sinotyrannus ?1?{2
3}001??????????????????????1???0??0??1????????0000?0101???
??????????????01{0
1}???0?0?0??1?11???1??010??????????{0
1}?????????????????????????????0??????001???{1 2}???????{0
1}0?11?????????????????00??????????????????????0???0????????0?00?00????00???{0
1}?12??1?000?0??????????010?????0????????0?01???????????????{0 1}????0?{0 1}???????????{0
1}???????????1??????0??????????????????0??????????????0?0???????????????????????????{0 1}0--
?????????????????1?????????1??????????????21?1????????0?10??????0???00?0??????????????????????0--
-????????0??000??-
???0???0??
Yutyrannus
?112001100??????????????????1?10??0000?001?120?0???1?110000?1????????0??0?1010-0100?00000010101{1
2}11?101000?0?00??0?{0 1}??0????0??001{0 1}?0000?---?0000000{1 2}1??0?0?00011000000110001 0{0
1}00?001001100001{1 2}0010?10?{0 1}0?0??002?0?????000000100?00010010?010??0???1?01(0 1)?(1 2){0
1}??0001?01101000000{1 2 3}0000?201??10000000??000000?0?1?0000?1??100001311111{0
1}1110001010000?0?01010110000-?1??-11?00?0?00010100?01002000000000?1110??011010?01100?100000-
01?01???0???????1?0-?0?10?0????0????0??0??1?00??0?01??0001?{0 1}{0 1}0--1-
?01??0?0000?00100??2??1011???0??10??10?11?-00001?000000?00-0011100000{0 1}00?????1???0?0??????0?--
--0?0?{0 1}??0???0?0----?--???-00??00-0001?1??11?0??{0 1}?00001??--??1?0000?1000000---
?0?00??????????00000?--??01-?0????-0????????0??0000?
Dilong ?103001010?-
0?1?100000??0??1110??0100?00?11101110?01100000?10100??????0010010-
?00??000000101011??1?0001?0001??0???0????????011{0 1}0?001?????0100000{1 2}0?0{0 1}????00?000000(0
1)10001010?0?0??001100001?0{0 1}10?100{0 1}000??00?000????000001000200?100100020001?0?110(2 3)(0
1)??(0 1)??00?1??20??0??00?100?0?{0
1}?11?1?0??010011?00000000?0000?10?1?0011301???1110000101?00001?010111000001000?-1102{1
2}?0?110001???01??200?0000{0 1}1001100?02?00??011?0?1?0?00-
0?010??1??????????0?????0????00?1100????????0??0?0000?0?0010?????-
?01???0?010?00100?11011111?0?0???01?10??1?-???01000?100?0???000200000??00000???-00???0000100----
000??01??????0??????????-00000?-??00??-??1000-000000000????0?01000?000000--??0?000000{0
1}000?000000?--??11-?0010??00???????0?00?10?

Juratyrant
??
??0????00??00{0 1}00?11??0(0 1 2)000?0?????00???01{0
1}00000010021000011000000??0?0?10???0?????0???????????????????????????????0110?1??????????????000
00????????????10????????00?????????1?0??100?10?????????1{0
1}?1??????????????1??????????????????????1{0
1}1????????????????????????0010????????0???????????????00???????????????????0?00??000????????0????
???????????????????1000--0-
00????000???0??0?????????1???1???0?????11?????????????????00??0???????????????????????????????????
???0??010??0??(0 1)00---
10?0001001?????000???????????????????????????????????0?
Xiongguanlong ?00?1????00?1?10?000000??????10100?00?0010120110-
?1110000000000010000???????????????0000?0101??1110(0 1)10000(0
1)000001110??{1
2}?10000010????????????????00000110?????????????????????????0010??{0
1}??0??0??0011??????????0?????010?????????????????????????010????0?0???00???0??????-
?11?1?2??1?00001?010?0??101?102000-
010???1?101????????????????????????1????????????1?????1????000?0?????0{0
1}???????????????0???1???????000??00(0 1)0100???????????????????00?{1 2}--????1?00????????01???1(1
2)11101-?1?111??110???11????????00?01?0????0??0??00?00000????-000?-?0?0????????0000{0
1}01000???0???-
?0?0??1000???????????????????????????0???????????????0??
Eotyrannus ?00{2 3}?00???0?????????????????{1 2}?101???00???N01{0
1}??1?????????????01?????????0?0011??0??1???000000{0 1}01011?0?1?00????00{0 1}????{0
1}????0??????1?????????????000N{0 1}0?1100???0NNN1{0 1
2}0000?0101?????0????????????????????????????????????0100??N?NN00{0 1}1?????10010-11????????{0 1}(0
1)01???1??0??{1 2}?20??00????0??0???0???????001?010???01?????0?0?00???????{0 2}001?0?????10??0?{1
2}0??0???1??{0 1 2}0??11?0?0????0?1?01????1??{0
1}01?000?0?20?0010???0?0?0???2?010?0??0000??NN??0????001??0??????????????1100?????0?00????????000
0000100000?1???????????????000???0??0????10?111????10????10??1?0???00??0?-
0??0????1??NN000???????????????0?-?0?0??0---???0???1(0 1)????????????????????00?10-1-00?1-101?N0-
001001100???0???00001??0??????????0????????????00???0--?021--????????????????0??00???
Alectrosaurus
??
???000??{0
1}??????????????????????????00??????00?1002000101100000200?0001??????????????????????????????0???
???????110100010???1????????????0?????0????????????0??0????????????1??????????????????????????????????
??????????????????????????????10011001?2?0??0?????0????????0???????????????????????0??000?1?????????
???????????????????????????????????0000010??0????????????????????????1???????0100??????????????????
??
??????????????????0000?0????000?0--0{0 1}11--????0???????????????0???00
Appalachiosaurus
?002011???0??????????????????????10????????1012??1????????????????????10?0?0??10?0???0????0?001{0
1}101????1????????????????????????????0??01???11100
0?1{1 2}010{0 1}000?{1 2}00001002000?01100000020?????10????0{0
1}????????1?????21???????0?????????001101???(1 2)0?????????????0??00????0?????00????1000--{1 2}01??{1
2}211?0000011????????100011?0?0??????0??0???????????????????????????????0(0
1)10111???2?0?????????000???0???????????????????????????0??000?1???0????????????????????????????????????-
?01???00000100 1?0???11?0?-???????????11???1????10000??0???????01???0000?00??000??????0?-??????00----
???-
0??????11?0?00?10000??0--?011-0???????????????1001??000
Bistahieversor ?0020110000-1?11100000?0?1010101001000001012 0110-
101110000?10110110?000001010 0020?100000010101??1?1??0??????????????????????0??1?1{0
1}0?????????????N??0??{0 1}00?{0
1}0?00000?002?0001??0N0000200?0??1001?10110001001??????(0 1)?????{0
1}?????????????????00???????1???10??00?00?0?001????00???????000-201???2101000001 1?001???1010112000-
000101?0???0?00?0???????????????????????0?{0 1}11001?{1
2}?0??0?11?0?00?0??0??000???????????????????00?0002?0??0??????????????0???????????????????????????01000?00
00?00100-121110?-1??1???01110????0??0010?0?100??0???10?00000????00??10-0000-?0?0100----
00??????????????0???????????-
??????????0??0?????0000?0-?0?11--
0?????01????????0????000
Raptorex ?002101N00?????????00????????{0 1}?101?0000?0N11120111-
??1??0????1?????????????010001??20??000000101?1111?101000?0011?0??2??0?1???0????????0N???????000N0
?0{1 2}{0 1}??00???N????10??100??010{0 1}00?{0 1}01{0 1}12100001??10{0
1}?1??0??0??00???010?1???N??2?0?????0010?00000????1?0{1
2}0??1??0000?0????000?0?1??????????????00110?0?0{0 1}0000???0??{0 1}0?100?10?00-
101?12210000000??0001(0 1)?010101N2000-?0???1101(0 1 2)???1100010??????20?00??00110?1??0?{2
3}0010??1??0?0??NN?001?00???0??0???0??0?????0?0?1???0?0?0?????N?01??0?011?01??00?1--0-
0?0??????0??0?11??1111100-??1??100??1021110????0?00000??0?-001?1NN000??????????????0????????????---
?0N000?????0??0????????????-01??00-010001???1????1?00110????{0 1}?????00????0NNN-0-
0?0???10?0?00?????????--????-1???????0???????????10?000
Gorgosaurus ?00211100(0 1)0-1?11100000100101110100100000101 20010-
101110000?10100110000001(0 1)01000200100000010101111010100000 1(0 1)000012?0002000000011000000---
0000000121001000000 1(0 1)4100-1110-0102000010010(0 1)0000111100010010001100200010110000002(0
1)0100010000101000000001001(0 1)01(0 1)?0001000(1 2)---00000?100000100??100110{0

1}000000000001?10000110010000(0 1)2011012101(0 1)00(0 1 2)01100010001010112000-
0001011000110000000000111020000?-1000001000-210000111-000000000010000001000?000000-
000?0002100001000??0001100?000011???000011--0-00101?00000100100-1(1 2)1010101(0
1)11110011111211110100100(0 1)0(0 1)100000-0010(0 1)000000001000010-0000-0010100----000000?00000-0----?--
0(0 1)0-000000-000101-?11100-{0 1}01001100--{0 1}0{0 1}-01000?0{0 1}0000-0-1010011000000{0 1}0000000--0001-
000100-01010-?0110000000
Alioramus ?003(0 1)(0 1)11000-0?111000001001?1110100(0 1)000001112011(0 1)-
10111000011100001100000000010?1200100000010101111 0?010000000(0 1)?0012?000{0 1}00?0?1?1{0
1}0?0??????????10?????????????????????????????0000010{0 1}1210000{0 1 2}{0 1}1??????(0
1)10001100?0?0101100N{0 1}00200??0?100000(0 1)000001001001??2{0
1}??????0?????0?0?00????00?????????10??00000?0?001?10?00?10?00?00-201???21010000011 0001(0
1)??1011111000-00000110(0 1)010??????????????????????{0 1}{0 1}1?01100??2{0 1}0??0?11?0?000N000??00(0
1)??????????????0??01???0210001?00????001????????????????00?{0 1}--0-1?1000??0???00100-1(1 2)1?10(0 1)-
110(0 1)110??111211?0???1{1 2}0100100?00??010?00000{0 1}0000?0?1???000-0010?00----
000??010???0?????????????-??0N-0-
???0??10?000000000000--0?11-1?010??01??????1000??1?0
Tyrannosaurus ?00(2 3)0011000-1111100000100101010100(0 1)00000101(1 2)0210-1(0
1)1110000111(0 1)00(0 1)10000000101001200100000010101111010100000 0(0 1)(0 1)000120(0 1)00100000101{0
1}000000---0000(0 1)000110020010001140 11-100?-010200001(0 1)01(1 2)100001111000(0
1)00000011002000101100000020010001000010100100001000(0 1)02(0 1)000000011---000000100010100??1000(0
1)000000001000010100001100102000201111210100010110001 0?0101011(0 1)000-
00010110001001000000000100200001-0001011000-110000111-000000000010000000000010100?0-
001?0002000000(0 1)00000011(0 1)000000011000000010100-1010000000 1001100-
121010101111110011112111 01001(0 1 2)000(0 1)000000-0010000000000000000010-0000-0010100----0000000(0
1)0000?0----{0 1}--00(0 1)-000011-010001-100000-000001100--101?01000000000(0 1)-0010100110101002000000 1--
0011--00100-01110-(0 1)(0 1)100(0 1)00000
Coelurus
??020?1?????????????{1
2}??????11????01?00(0 1)00001100?????000000{0 1}??????????????N??020110010NNN1?{0
1}?????1??????????????????????????0100200100 01?0010?10010NNNN?0{0 1}0?????????????????????{0
1}?020??{0
1}?????100110????0????1?????????1??1???0???00??0???0?????1???????????????111???????????????1??{1
2}??????????????0????211??{0 1}0???110?0???1000?????1?0???????{2
3}??2000???00????N0?0????00?01?00000?????01???0?1??????(0 1)????00???2000011001?????{0
1}?????110100??000??0??0?????????????????????????????????0??0????00?00??1????????????????????????
??????????00{0 1 2}010?????????????????-?????????????0-1?1?N0-00?01110011{0 1}1{0
1}?N???0??00N?N????????000???00?0000???-????1--?????0?????????????0???01
Tanycolagreus ????????0???????????????????010??00???0N1?1??{0
1}????0????0?000??????????????????????100000??0??(0 1)??011???????????0001{0
1}?0??0???0???1????????????000N0001{0
1}00001?1NN000000001100???????????????????????100200100011001000101 1NNNN00000?0001001????0???1?
???0(1
2)1??1??00110011?0???0?020000000???1?00??00???0?000?0?1?0?0?1??0??0??????0??21????????100??1?0?
0???N????????????1??11??1001011001100100010 1??1001?00002?01000??1000??N?0?0??0?001010000011????
01?000?1????????????00??0100000000 1000?????????1000000000100?00?1?0?????1??01??0?0?0?????1?100??
?0???????0???10??00?????????????????00?00??????0????010?????0??????????-01001?-010000-1000N0-
00001100100000?N10000000???????????0001000?210000?0--0001-????000????????00000?01
Aristosuchus
???
????????????????01{0
1}??00000????0??????????????????????????????????00??0??????????????????????????00?1{0
1}???2?0?1?????????????????0?????????????1??????????
???????????????0???11??????????????????????????
???0????????????????????????0????????????????????????1
???0???
????010??0???0???????????????
???????????????????????????????0?
Bicentenaria
???????0????????1????00????????0??0?0??00??????????????????0?0????????????0??????????0100000??101?
???0????????????100?1???00??0??1??????????????????0??1????0?????????00?00?0?1???1???00??????????????
?????0{0 1}000?10??0??0?1100???0??0?0?????{1 2}?????????????1?????{0 1}????0{0 1
2}0?????????????????{1 2}0???????????????0??00???1???00??0?????0?{0 1 2}(0 1)????{1
2}1???0???????0??????????{1
2}?????0???1?????????????????????????0????????????????0?0?????????0?0?0????????????????????0???1???
????????0?????????????1000?0??00????????????????0??0???????0?0????????????1????????????????????????0???0
?????????0??0?????0?????????0?-???00????????????010?????????????????-?????????????1-
10?????101011100?????????????????0???????0??10?00??00?0????????????????1???0????????????1?0??1
Xinjiangovenator
???
???
?????????????02?001?1000???
??????????????????????????????????1??
???000?1???
???????0000000??0???
??0?????
0????????????????????????????0?????1

Zuolong ???211101??????????????????{1
2}010000????0010?200110?0110000010?????100?0???????????????0000?{0 1}101??1?1?11010000(0 1)????{0
1}??00{0 1}0000?????????????????0?????2{0 1}00?0????????00??0?1?????0001001???????????01{0 1}1101{0
1}000?10?1?0???????0001000????1002?0?{0
1}?0001???002?0?1?????100????0000?0?????0?????0????????1???????0?000??0100???010?101??{1 2}{1
2}?10000{1 2}0??00001??1010???0?0000????1?(1
2)???????1?1??????????00??0??00?10???210000?????0?00?00????0???????01000??0??01?000??????00???
????1??{1 2}0??0?0100????010--???00100000001?01????1001??????0010??1?0????????0?0?0?10??(0
1)0?0???10??000?????00????000-
?000???????010???10??00???????????????????????????1?000??10?000101????0??????????00???-
0??0000000??????????0--?001--??????0????????0???010?

Zuni
???201?????????????????????????0???{0
1}??1??0????????0?00?01????????????????1????{0 1}????????????{0 1}????????????????????????????0??{1
2}{1 2 3}????????????????????????????????????0????0????1?0?????0??{0 1}??0??????1?02???{0 1}???????{0
1}?01???1??????0??????????0?????????????0???{1 2}????{0 1}???????????0????????0????{0
1}????1?0??0??0?????????010??1??0????????????????????????1?0??????????1?{0 1}??????{1
2}??0??0?0???????0????????????????????????????????0??0??0
0??000????????????????????????????????????0??0????????????00??????????????????????????????0????0
??000??0??????????????0????????00????????{1
2}1-??????????????????????0??

Ornitholestes
?10201101 00???1??0000001???1?0??00001?00001000?00?0100000000?00001?0?0001(1 2)0000000{0
1}0000100101011111?1?00?100001011(0 1)0?100?000011100???????????????10000001?0????000?0{0
1}0??0100?00011102100001101?{0 1
2}110???01?00?1????????0000010??0?100201?100111001?0110?1?????100????00000???0???11????000100?
110?0?0?100110000100?10000-211?11??100000011010?(0 1)??2011002?00000000000(1 2)(1
2)10??????10?????????01??000?0000?1?10020??11??0?0000000?000???0?101000??0?0??0???2?0100?00????
00???10000000010??1001001-(0 1)?????????1?0??0?0?10001?0-
1?010?0??1?021?001??000?00100?00?00?1100000?00000000???00?-?000000----000?{0 1
2}0100?0???????????????????????????1-101000-001010000--?10???00?????000--000?0000010?????????00--
?010-0000-?-00??????10???010?

Sinocalliopteryx ?00110??10??-?1??-
1??0?????1????0000?0?01?11001?0000?000?0???0000???0?0000?1?0?10???0000(0 1)00101111?010100??(0
1)00?0??1?0????00??0{1 2}100-0000---000000?010000??00001000000000001010{0 1}00?{0 1}?1002200001{1
2}01?0?1010?0???001?0?????000000000100010020??11??????1?011?21?00001001110000?0?10?001{0
1}?1?01000?0?00?0000?000???0000?0?010?00??011?21?10000{0 1}01001?1(0 1)00101000{0 1}000?0?????10(1
2)110?10000000?0100200?000101?0?10?002100?001010?00?000?01?10???1????0?0100-
?01?0?????1?1?0?????????00?10??0???000?0{0 1}{0 1}??1-
??0??0?0000??01?0?01???11?11?????0??????101100?0?000?01?00-0000100?00??0?0?00????0????????00----
00{0 1}?00?00??0-0----?--???-00?000-00000?0?????????0???????????{0 1}-0100000{0 1}0000--
??000???0?1????????000--??01--0???1??????????????0000?

Huaxiagnathus
?00101?N???????????????????2??0????0???N??0???1?0??N??????????????????0?110?0-?????0?0001{0
1}01011???000?00??0??????{0 1}?0{0 1}????00?0?0{0 1}0-0NN0---0000N0002000{0 1}??1NNN100000000000111{0
1}00???0012100001 0011{0 1}?1??00{0 1}10?0??0000010NNNN0000020001002???1?0?????1-
021?21?00001?010??000?0?30??00{0 1}?1??1001??000?0{0 1}01?0?0???0{0 1}0??0001001103111 12?110000{1
2}01?????(0 1)?010110N{1 2}00010???-?10{1 2}11001001000?01100200?000001?0?10?00210??00?000?0????0-
01????0????0???01?0-????0???????010????????000?0????0??000?0?0??0-??????????????{0
1}?0?00???10??????000??1011???0N?0???00?00???-
00001??100?????????????????????????????0N??0?100??0?0----?--000-000?10-0100??-1??0N???0????????????{0
1}?N000000{0 1}0NNN--???0????????0?0????0?0--0?01-?-??????0000-?0?0?000100

Juravenator
?001010NN0?????????????????10????01?1??NN01000??0?1NN000???????00??00?0?11000-??0????010{0
1}001012???00??0???????????0?0?00??0???100-0NN0----000N0?011000???----
00001001000111000?0000???????????????1{0 1}?10111?00????????NN?N1?{0 1}?0001010020?0{0 1}0?1????0-
0????1?000?1?010100000??(1 2)000000?1???001??0?0?0000?0?0???0{0 1}00?100???1003(0
1)11121?1000010??010?1?000000N2000000??-?00{1 2}{1 2}?1?0000?00???1002N000000?1?0???000{1
2}10000000?0?0?0??0-00?00?0?1????0????0-?0?????????0?0???????00??00?100??????00?0?0--
??????????????0?1?0?000?100???????00??1?11??00N0?0000??01?0?-
0?0?1N??00??0?????????0??????????????0N??0?10?????0----?--N??-??????????1000??0?N??0???0??00??{0
1}?{0 1}?N0??0?0{0 1}0NNN??-?N??0??????????0010?0--0?{1 2}1-?-?????0??0?-?0?????00??

Aniksosaurus
???
???????0??????????{0 1}???????00???????????????????????2101?0?????????0??1?0?????1?0?01001?1??{0
1}???????????10001100{1
2}?000???0??0?00?010??1??????????????????2??1??????10???????0??0110??0????????????????00?????0???
?0????11??0???????1?21??????????????1??????????????????????2?1???????00???????????????1?001000?20
0000?????1?????0??????????00??0{1
2}???0???0?000??????????????????????0000??????01000????0?00?00????0??????????????????0????020??
????00?????????0?????1??10???1??????????????????????????????
1?0?????1??0??????????????????00??-0??????0000?????000???-???10-?????0??????????0???0???

Archaeornithoides
????1?1?????????????0???????0???1????????????????????????????????????0?0?000?1????????0?0N?1--
1??0???
??????????????????????????????????????0?{1 2 3}0???????????1-

???0???0??????????10????????0??11??????1?0??0?????????
0???????0????????0??0?????0???????????????????????????
?????????001(0
1)???1?????????????????????????????????????0??0????????
0??N00???0?0?????????????????0---
???
?????????????????????????????????0??
Scipionyx
?102001NN00??0?????0???1???1100?00001?0NN00100110?1N?001010??????100010000010-??0?0?0010{0
1}00101221?00?100000011?0?{0 1}?00?00?{0 1}?0??????0NN(0 1)---
1010N00020010???1NN10010000100000010O?{0 1}?1{0 1}?1?000?1{0 1}0111010?10001?0?{1
2}?0?????????????????????00200?00??11??1-02(0 1)???000002?010?00000??????????????0?00??{0
1}0010000?0???0???0?11??00?00031111{1 2}1?10000101?01011?000110N2101100?0-?001(1
2)1?01001001001010 2N0000201????????0???00?00??0?000?N0-01000??????0?0?00?0-
???0??????10000????000N000?000?????000?0?1??0??1???????10???1???000(0
1)1000????1010?0??011?0?N????00(0 1)101?(0 1)0-00?01NN000??00???00???00?-00???0?----0N0000?00N00?0--
--?--N10-01??00-010001-????N???0?00?100??????N00000000NNN-
??0N00???1????????????????????????0????00100000????0000?
Tugulusaurus
???
??????????????????????????0????????????????????????????????????0????00??1?0?????????????????????????
?????0{0 1}000?100?000001?00?{0 1}?0???????????????????????????????{1 2}??????0???????????????????{0
1}0?????0???0????{0 1}??????????????????????????????????{1
2}1??00????10?????????0??????????????????
????????????????????1?000??????????????????????????????0?????????????0?00100???00?0????????????????
?????????????????0??
??????????????????????????????????00??????????????????0?0?1??00000?????????1-
?????????????????1??0??0
Sinosauropteryx -
0030??NN0??????????????????1??0?0??0????N001?0??0?0??00??101??0???????0011000-?{0
1}???0?0001110011?1?000?0(0 1)??(0 1)00????0?00????00?1?0000(0 1)NN0---?000N0{0
1}02100100?NNN10001?2110?0010?00??00{0 1}11100001{0 1}0101?(0
1)??10000?001000??11NNNN00000100010020??{0 1}00??0??{0 1}-0(1 2)1??10?0?010021100000001 0?000{1
2}?11000111?{0 1}00??{0 1}0001?0?000000100010000?{2 3}1110?{1
2}100?00101?01??1N010100N1000?00?0??00(1
2)210?10?10000?000020010?0001?0?100002100?000100?0??NN0?00?00?010??0?0{1 2}00?0-
?1??000?1???010?0???????01?000?1??0?00?0?1--0-
000??1??00???11?0?1000?10?????1??0???011?1??N000?000?0??00-000?1NN000?????????????00?-?0?0???---
?0N000??00N0?-0----0--???-00?000-??0101-?000N1200?00??00--000?N000??0{0 1}0NNN---
0N0???00?0000????000?--??11-?0????-?0????????110?000
Santanaraptor
???
??????????????????????????????100??0???1110000?{0
1}0??????010001000?000????N?NN00000?0001???????????????????????????????????????0?10?0??0?0???0??
???????0?????0????????????100??????????1??
????????0010???3?0?????1??0????????????????????????????????1??
???0-0?0??1???????0??0????????????????????0???????00{0
1}?????????????0??
??????????????????????????NN--????????0??????????????--??11-????????????????????????
Haplocheirus ?102011NN00-
0?10100000?11100110100001?0NN00010?10?0N?0000010?1001?100100100110010100000 0(1
2)311012011000?00???-00111{0 1}0?0100?10?0??{0 1}0?0NN1????101N001210?1101NNN?000100(0
1)1000???0?00101{1 2}00100001{0 1}0112110?10101?000?0?10??NNNN?00????00{0 1}00101010010?000-
02?011??0?111010110?001?10000?0(0 1)???1?0110000110010010010 1?(0
1)00?1010?0012111?21?00000100001001?010100N20000000001?02?11?10?10101101102000101?0100010??02{0
1}010?00?000000NN000?0000??101001010?1????100??10??01(0 1)?0??0????020000001000001?{0 1}{0 1}--
?0001?0????00?000?0-10??10?110?01???100?{0 1}??10??1?{0 1}?0(0 1)?100?10-00001NN000?00?0??00???000-
?0?0?0?----0N0100100N00?0??????????-00??00-01010?-1?0??120?0000000--0?0?N000010?0NNN0--
0??0?000???????00?0???????{1 2}-?01???-0????????????0010?
Compsognathus ?002011010????0??000?0?????{0
1}??01?000??000?00011?0?000???00??01??01?00?00010{0 1}0-0100010000(1 2)1110(0
1)211?000?000?001000?0?00?00?00?1?000-0000---{0 1}01?00?121000??0?0010{0 1}000?1000?212????{0
1}???010000?210000?(1 2)0?10?1?1?0??00??010000?00?000001(0 1)03?0?0011?1??0-02(0 1)??(0 1)?000?1?01{0
2}??0?0?0?20000000???(0 1)00110100??0000?100??0?00?0?0100(0 1)0?31111111000?01?100(0
1)011?010100020000001 0-1002(1 2)10010(0 1)(0 1)0000?0010200000?001?011000?2{0 1}00??000?0?000000-
0?020???1??????00??-????0????01?0100??????0000???0??1??0?0???1???-
00????????0??0?0?0??000?????0??????0???0??11000(0 1)0(0 1)?00(0 1)100?0?-0000100?00??0?0{0 2}00??????0?-
?0?0000----00000010?00??0----?--010-00??1?-0?0(0 1)?1?????0??????1??????????01100?0?0000--
??00??0?0???00??0??000--0011-?0???1?00?00?10?01?0?00?
Sciurumimus ?000101N?0??????????????????0??0??00?1?0NN?0{0
1}0110??0N?000?0?????????????00100100?10?000000{0 1}1110121??00110(0 1)??00(0 1)?00?1??0?{0
1}0?000??100-0NN0---?001N00021?1{0 1}?00NNN?00100?(0 1)1000111{0 1}00?0?-201000201{0 1}0{1
2}?2?20?100?1??0??0?????N?NN000?00000100201?000?0???1-0(2 3)0?21?000?1?0211?00000?20?00?{0
1}?10?0?01?0??01000000?0????{0 1}0??000000000111101{0
1}?00000100?000?1?000000N2000?01?001001110?10010000011112N00001111?01000?02000?0010?000???N0000
?11?01???0?0?0??0-????1???????0?0?0???????00?100?????000?0{0 1}{0 1}??0-

????0??0?00??01?0?01?0?0001??000?00?????0010N0?10-00100000-00?01NN000????????????0????????0?----
0N0000?00N00?0?---?--N??-00?00?-0101010???0N???0??1??00--??0?N?0000000?NN--???0????0?{0
1}????????000--?001-?0???0-0??????0????201??
NGMC2124
???3???0??????????????????????0??0??1?00??121?????????????0????????????000?{0 1}1??{0
1}0?0000?01101?121???0?????????????{0 1}???????{0 1}??1?000-00?????????0{0 1}0??????????00?00(0 1)0{1
2}?1100?010{0 1}00?{0 1}?1{0 1}02?10002{1 2}0{0 1}??0?0?0?010??0???????00?0??{0
1}?010?0100?????????????1-020??1??0??1??21??00000??0?00???1????0???00010{0 1}000??????0{0
1}0??0??10????{1 2 3}??11?????000?0?????????1?10??2000??????1??{1 2}{0
1}??????0?????1000200?001001?0?????0200??0?100??????0??0?????????????????0???1?????????????????
????????????????00?0{0 1}1??0-??0??1???????0{1 2}?0??0??1?1?????????????21??????????0??0????-
?0????0?0??????????????0??????????---?????{0 1}????????0??????????-
?0??0?????0??????????????????????????0100??0{0 1}0000--??000???0??????????0??--??11-
?0?????0????????????????
Aorun ?1?3011NN1??????????????????110??000??0N??0{0
1}01??1?0??000?01???????????0?12001???0????0(0 1)0(1
2)20101211?0?1?00?0111??0?2?0??????1????????????????????????????1?1NNN?0{0 1}(0
1)01?11000???????????????????????1{0 1}010???????????010010NNNN0010010?01?0200??0???0??0-
?20????????????1?????????2000??{0 1}????1?0?1?210???0????0?????00???0???11?111????000000{1
2}0??010?1??{2
3}010?N2000101??0?002?1????????10?10?02000002????01100?03?0??0?????????NN000??0????????1?0?????
0??00??????00100????00???????????????00?????????1?????00?????10??000?1??????00??????0????10?0010
?00001?0?????0?NN?0???0?????????0?0??????0?----
0?0??010??N10?0?0{0
1}0???????????0????01?20000?????0?11--????0?0????????0?0?0100
Naze_dromaeosaur
??
??
?????????????0??????1?0000000????0?1??
???????????????????????????????????1??
???????0???{1 2
3}?0?????????1???1???
??????0???????????????????????????????1???
??0?--??1?-
???????????????????????0
Deinocheirus ?0011100000??????10000?????2110101001100000210-0?10000000?001000???0?20020{0 1}0010020001-1---------0-0??010000000112?11(1 2)10000010110?(0
1)??0???10001000200000 11?0013000001110001010100100121{0 1}???1001000000{0
1}0001?0020001?10110000101--0?100{1 2}--000-01???1-0(1 2)10?0?00002?021000000000?0(0 1)?-
?0???1?010100100{0 1}-00010?01-0--1100??00--0--1102110000000?-11111010110020100010?00--(1
2)01?000020100110002000110100 11?10--012110-10?000000?1000?0010?1110000000?0?0010000??0??0--
1??1???11000100011 1?0001012?0?-00100(0 1)001000?0-?0?00--100-1???0?01??0021??0-?000?0-0-
01?00?0011100000??0?????????0????0?0???---?000010?0?000?0???????000-00??00-
0100010101?0??0010010000?11{0 1}?00000?100000-000000??1?0{0 1}????????010----11-
???????00?00??0?0??00000
Hexing
?00?0??010????????????????1?2??0??00?1??000?100????0110??000111010???????0001?0?????001-1-0-???--
??-????0010???????????????????????????????00?10?1200?{0 1}?????0??10000(0
1)100?????????????????????????????100??????????????11?{0 1}00{0 1}?0{0 1}?01{0 1}102-
?00??00?0?1?????????0001002---??????10?0??20??????001?00??{0 1}1001?1?0???1????0?????-0?-
1??1??0?0000??-1??1?0101??02000?010?00-?2?????2?11?10??1000100110-
??1?0?10?102?010?00?1??0??01?00?0?0???????????0????????0?????1???-0????????01?00??1?-
??00??????????0??????????0-????0--?0?-
???????0??00????10?1020?0??01?0??0?101???00????????0????????0?????????00??????????????????????-
00??0?-??00-?-???00???0??0??0?????{0 1}?00????0{0 1}0??????????????0?????????????????11-
?????1???????????0????0??
Shenzhousaurus ?0031000??0?????????????1???2100?????1???00010{0
1}100000001?0????????????00001010???0??101-120---11-??-??????????????{0 1}??0?{0
1}0?00?0???0????1?????????????????????0??00000(0 1){0
1}0?00100?0?0100211001?100110110?00101?0????????????????????????103--01?01????0-0(2 3)1?21?????{1
2}0??10000000?????????????1???01?010001???????11010??0?1-?0-0{0 1 2}1-?011?100001 0??-
??????100100200000???00-0?{1 2}10????????????00?300???201????????0?0???????1??000??0000?{0
1}???????????????0?0?1??????????111????0?????????????????000000001-000??0????????0-?0??0--
?0001?0???1???0?20??1??????000-
0???0??0???0?000??0???????????0?????0?????????00???0100?00?0???????????????????????????????????????
?????????00????1??000-000?10?0?000??????????????????????????0????????????000?
Harpymimus
?002101011?????????????????210010?1?????000200000?100000010???????????00120100000?0101-120---11-
0?-00101010(0 1)000011?01100?0000011001???????00?0{0 1}?12100000100011010{0 1}1110?00{1
2}??00?0100?????{0 2}????{0 1}??110???0????0?0??1?110000001 0?????1003-001001?1??1-?2(0 1)??1????0{1
2}?02010?100??100???10????000?100101{0 1}1101?0?0110?0?100??00--11-1???10?00000?0-1??1??{2 3}01{1
2}002000101?0?0-0(1 2)11??10?{0 1}0100010003001 0?210???010???2{0
1}000?1010?10???1000??0000?00???010000??01?????1?1?1?1?1?00??1?000000011-
0?00001?000??0?000??????00-10?10--101-?????????000??1?1??0000??(0
1)10??00???0?100000?????????????0????????00----000010?00000????????????-?????????01?1-101000-
001000000--1?0-00000010000???0?00000????10?00000?01--?{0 1}10-?0???1-????????100??00?0

Nedcolbertia
???
??????????????????{1 2}???0{0 1}0???????????????????????????????????????0?1?(0
1)100?????????????0???0{0 2}0??00?0?10?0000??002?00101?0N00??1?0??0?1???????????????????????{1 2}{0
1}?????{1 2 3}0????????0?010?0??00???????????????{0
1}?????0??????????0??0????????121??0?????????????00??{1
2}100?10?0?2101?0?????(0 1)?????0?0?????????????????????????11?000?????????????????????????1???????{0
1}???0?0?000000000?00??????????????????????02????1?000???????????0????????????????????????????
???????????????????????????????????????-????????????????1???00????????????????NNN?-
?1????000?0????0?00??{0 1}--?001-?????????????????00?00?00
Arkansaurus
???
???
????????????????????????000010?????1???0????0????????????
?????????0?????????????0???
?????110???2?0????????1??
??????????????????????????????????????0????????010???
???
???1--?011-????????????????0???????
Archaeornithomimus_asiaticus
???
??1??01??0?00000011?01100?000?01{0 1}????????????1001021000001?00?3{0 1}10{1 2}21{0 1}000{0 1}0{0
2}0???0000(0 1)210000{0 1}000001(1 2)000000100010001011000000200?0??1????????????????2?01{0
1}??0?0?002?10?00000{0
1}00???00???1?0???10??00?1?1?0?0??0??1?0?100???????1211??????????????1?0??????????????????211??
?0??010?110003001002?1100110???20010?1??1?1????00?0????0?00000?010??0?0011000???????0000000???
10000011?000?0?10--0-001011000??00?00????????????0????????10???????010??(0
1)????00?00??1????????????????????????????????????00?010??00?????????????-?00000-0?0??1-1?1?0??{0
1}01000100--000?00000?000000---0????000001000000 0?01--?011--?????-????????000?00?00
GIN960910KD
???
?????0?010?100???1{0 1}?0?1{0 1}0??0??00?????????????1?????2{0 1}000001??N??0101111000{0
1}1?000?01000010?00{1 2}?0{0 1}0011001000100010000001NNNN00100?0??1?????????????????0(1
2)1?21?????20120000?000??0??0000???1??0??{0 1}0???0???1??????{0
1}???0??1?0???????1211???????????????????????1?????????????2?1?????{0
1}?10??100030010?221100?1?001210??00??0?1????N0?0????0??0??????????0???1100??1??????0?0??????0?0
??0?00??00???1???-
000001000??00?00?????????????????????????????????????00??0??1???????????????????????????????
???00??10???0?0??????????????????????1???1?10N???????????--100?N0????000NNN--
??N?00?00001??00?10001--0???-?????1-????????1?0??0?00
Beishanlong
???
???????0??????????????0??????0?101?0?????????0010000200?000???0??{0 1}00?11{0 1}0?0???????????021{0
1}???{0 1 2}????????0100011002000??11000000100?0001???????????????????0?0200{1
2}??0????001000?120??????0??{0
1}1???0?1?1?0?0????????0??0?????????21???????????????0????????????????????????11?1010?????????1
0????0?011000?2?01000??01??????????????0?0?0?0???????????0??000????????????????01?0000?0?0?0?????
???-
??0??1?0000?00?00???????????????1???1?????0?002???????????0???1???????????????????????????????
?????????????????????????-000000-010101-?1?000-?00010100--?00?0?????????0?--????????0??00000??0???-
-0{0 1}11-?????1-?????????00100??0
Garudimimus ?0031010100?1?1???01101??1?2100100101?00000200000000000(0
1)?0001000001?0011012010000020101-1---------?-?010???0?0??01{0 1}?01(1
2)00??00?????00?0??????????????????????????????????01000010100?????????0011100100011002000101
10000010010??1103-01000101001-01102?????????????0000???000010???1??11??01012?0?001?1-0--
1?0???00--1--?0211000000000-1001??2012002000101 1000--2(0
1)1???????????????????????0?11011000?210??0?0????0000100??000?????????????0??01?000?10001--
?0??0001???????????????001000??00?00100?00100-0??10--000-1??0100?0001010?1???0000-(0
1)101??0??010?00000?00000000?-000?-0000100----
0000?010??00?0??000??00000?00000100100000
0{0 1}--0011--?0????00????????01??100
Aepyornithomimus
???
???
?????????????0?1????01000000201--001??0010?-
00?????????????????????0?????????0??0??????????1???
??????????????????110--?2?0??-
?????1???
?????????????????????0?????1?0100??
???000???0----11-
1????0??????????100????0
Sinornithomimus ?00{1 2 3}1?1011??1?1????1101??102110101111?00010200000000000000(0
1)010?000????110020{0 1}00?00?0101-1---------?-01101011000001 0?01100000?001100?000----
0100000200000010001201000110000100001010002100001000001100000011001100??1?000000200??001003-
?000(0 1)111??1-02?0201-00?1002110000?0????01?00???1000?110?100-110?0?01-0--?1??10?0-01--
11111?0?00200?-1?01?0301{1 2}002000101??00--2??0-{0

1}0?10100110113001002011001112002000001010010000000000?0(0 1)???10?0??100?0-?01?000??00?1--
?0??1???-010000?11???000??0000-00010000?00100-00??0--10101??0100??001{0 1}1??1?001{0 1}?0-0?01-00-
00?01?0000?0????0?????000-00?0???---?000?1010?000?0-----------00??00-010101-??1000-{0 1}0?00??00--{0
1}?0?00000?010000--000????00000??00000000----01-?0????-00------????00???
Ornithomimus
??
???1?00?3{0 1
2}??0?0?0101100000020???001?????????????????????????
????2?????????011????0?????0???10?????????0??????????0??0??????????1??????????????????????????
?????????????????????11000?0?1????????110???2?0??????0?1????????????????????????????????1????1???
???????????????????????00?????????????????????0??0??????????????????????1?????-
??100???
??????????????????00000?010????????????????000000000?1--??01--????1??????????100????0
Cerro_del_Pueblo_orn
??
???????????????????????????0??
00?1{0 1}?{0
1}000?10???0???????00??2??????1?????????????????????????????0??????????0???????0???1??????????{0
1}?????????????????????????????2??
100??????{2
3}?0????????0?????0?????????????????????????000??0000000?
0?1?0????????????????????????????????????0????????0??
???0??0??0???????????1--????-
???????????????????????0?
Tototlmimus
??
??{0 1
2}???{0
1}00????????1??1?0???0????????????????????1??????????0???
???1?????????????0???
??
???????????????????????????{1
2}0???
???01-1????0??????????????????
mongoliensis
???2?1?0?????????????????????????0?????0?020????????0?0?0??????????????020?00?1?????1-1---------?-
0???1????0??01{0 1}00{0 1}?{0 1}?0????01{0 1}????????????0?00{0 1}?200?0???000130?11111000010??0?{0
1}???02?000?1000001100???011?0?000?0100?0000201--0010?{2 3}-???0??1???1-021??{0
1}??0??200200001100?01?0?-{0 1}????1?011?201?1?21?1?0?0?-{0 1}--11?01000??0--112211?000{0 1}0??-
11?1??3010?0?000?01??0?--2010?{0 1}??10100110003001002101001111--?2{1 2}000-
1??00?0?0?1?000?0?0??1??010{0 1}0??0?????00??1???0--?00?0001?0000?00?1?00000??1??0-00?????0?00000-
0??00--1???????????????0?0??0??001??-0?0?????00??1???0??????????????0???????????????0??0{0 1
2}010??0???????????????0??0?00101??-???????0?10101????????00000?0{0 1}0000--?000??0??0??00{0
1}0???00?----00-?????0??????????????00?00
MNAPI1762A
??
??????????????????????????0???0{0 1}{0
1}???2?????????0???0101100000201-
-0?1?????????????????02???1??????????????????0??1?-
??????????????????????0??????????????????????????1??
?????????????????0?11--?3?0??-
?????1????????????????????????????????????1???0??0
0????????????????????0???????010??0????
??{1 2}0????0{0 1}----11--
0???1????????????1?????0
sedens_holotype_only
??
??????????????????????1???00?????0??{0 1}??000?01??01?000?{0
1}10{0
1}??0??????????????10?0??????????????????
????????????????????1??10?????????{1
2}???????????????????????????????????????0?????????????????????10??????????0???????????????0??
?????????????????0???????????????????????1???????????????0010?00-
????????????????????????0???????????????1?????????????????0??0???????????????????????????????????
???????????00???111--
0?0?0???
Rativates ?00210?????????????1???????210110???????0???0{0
1}0????????????????????????{0 1}??2??0????????1-1---------?-??????????????????1{0 1}00?0?0??{0 1}{0 1
2}??????????????????????????????????????{0 1}1{1 2}00?1010{0 1}121000?1000?{0 1 2}120?{0
1}0001?00??????1010?000{2 3}01--001?03-?10????????1-?{1 2 3}??20???????1?????010000?1010-
00????????????00-????0???-0--?1001??00-?--?02{1 2}110000000?-??????{2 3}01000?0?0????0??--
?????????????????????????0{0 1}1?0111--?310??-??????0????0???????????????????0?0???00???????--
????????????????????????00{0 1}???0-?1??0??0000?0?-00???--?00-??????0???10?0??????110?-
??0??????00??00000??????????????????????0---
????????0??00???111-
00??00?00?1010??00000?1----11--????0?????????1?10??000

Struthiomimus
?00110101100111?010110?101021001011011000102000000000000000101000001?001100201000102010 1-1---------1-
001010100(0 1)(0 1)0010?01100000?0011001000----001(0
1)001200000010001301001110000110001010002100001000010100000011002000001?(0 1)00000201--001?03-
01000001101-03001(0 1)?-000(1 2)?02(0 1)??01000?010(0 1)0-00???(0 1)00?(0 1)1211100-11100101-0--11001000--
1--1022110000001?-11110030100020(0 1)01011000--2{1 2}?0-10110100110113001102001?0111--032000-(0 1)(0
1)10010000100000000?1??01?00010-0?11000{0 1}10001--????0??1-00?0000010000000?{0 1}10?-
?0000?00000?00-00-10--100-???01?0(0 1)0001??1?1-001(0 1)0?-0100-0?-001(0
1)100000??????0?0???0????00???0---?00(0 1)010?00??0-0-----------00??00-010001-??1000-{0 1}0?01?101--{0 1}1{0
1}00000?00{0 1}0000--0000?0?000010010000001----{0 1}1-?01101-00------110?00100
Gallimimus_bullatus ?002111011001?1001011011010211010 10-
110001020000001000000101000001?001100(1 2)010001020001-1---------1-001010100000011101120000000 1100(0
1)00????0110001200000?0001301000110000110001010002100001000001100000011002000 10?1000000201--
001002-0100001?001-0200(1 2)0??0002(0 1)020110000010 1000-00???100011211100-10110101-0--11001000--1--
1122110?000010-110100301200200010 10000--2110?10?1010011000200100101100111--030000-1010010000(0
1)000000000110000000??0?001?000{0 1}101?(0 1)--00000001?000000011000000010?00-00100100000000-00-10--
100-1100-001000101 1?0-?01(0 1)0?-0100?00-0010100000000?0{1 2}0000-000?-0000100----000010100000-
???????????-?0??00-010001-101?00-001010100--110?00000?0{0 1}0000--00001000000000000000001----11--00100-
00??????111100100
Timimus
??
??
?????00000110???0???????????????????????????????
???0?????????????????????????????2??
??1???
???1000???????0???
???0000???????
???????????????????????????????????
Anserimimus
??
??1?0??000?(0 1)?000010?01100?000011001??????0011000200100010001301 22?1(0 1)00?{0
1}1100010100021000010010001001000010010000011000000201--0?1??????????????????021?1{0 1}??0001(0
1)0210101000?0?00?-
00???1000??21??00?1?1?0?01?0??1100100??????10221?????????????1?0??????????????????(1 2){1
2}?0?10?101001(0 1)0112001001111?0111--031000-1??00?????10?0????00?11?0??{0
1}0??0??1??00??1??????0???00???00100001100?0000{0 1}00?0-
?00??000000?00?00???????0???????00???11??????010???(0
1)??????00??1????????????????????????????????????0?1??0???0????????????-01??0?-??0101-
?1110??000001???--{0 1}?0?0?000?000000--?000???00?{0 1}?0?00???00?----0{0 1}-??????-?????????00?00?00
Qiupalong
??
???0???01000???0{0
2}0???00010????????0?100?00100000020?????1??????????????????????????0?00?1?01???10???1
?????????1??????0??1?????1?0??0???????01?1???
????????0?00111???20?????????1??????????????????????????0???000?1???????????????????
0?{0 1}{0
1}000?00????000???0??0????????????????0????120??????100????????????0????????????????????????????????????
???1?0?-
00???0?????10?00000??{1 2 3}--??01-?????1??????????000???00
Dromiceiomimus ?00(1 2)001011?00?10010110110102100 10(0 1)11(0 1)10000(0
1)20000001000000(0 1)01000001?0??101(1 2)01000(0 1)0?0001-1---------1-0010111(0 1)000001(0
1)101100000000110 01000----0(0 1)1(0 1)00(0 1)2000000?000030(0 1)2(1 2)11100001100010000(0 1)(1
2)1000010000(0 1)11000000110 02000?010(0 1)00000(2 3)01--001103-0(0 1)(0 1)00011001-021?(1 2)11-001(1
2)002(0 1)1001000100000-001101000(0 1)1211100-11110101-0--11001100001--10(1 2)2100100(0 1)01?-
11010030110(0 1)20001011001--(1 2)110-1(0 1)0(0 1)01(0 1)01(0 1)0(0 1)(0 1)3001002101?011(0 1)--0(2 3)00(0 1)0-
(0 1)01001000(0 1)(0 1)000000000?(0 1)00011100?0-001?0001100?1--(0 1)0??100(0 1)-01000001100000 0010--0-0(0
1)000?000(0 1)0?00-00-10--10001000100(0 1)000(0 1)211111001 0?0-0101-00-0010100000???010?0???00?-
?0001?0---?000?101000(0 1)0-0-----------00??00-010101-10110??001001100--0???0000??0{0 1}0(0 1)(0 1)(0 1)---
00000?0000?00??0000?{0 1}----00-?0010?-00------?(0 1)?100100
Kinnareemimus
??
???????????????????{0
1}???{1 2
3}??????????????2?0?????N?NN0010?????1?????????????????3?????????????????????0100????0?????????
????????????0???????????0??0????????1?1???
?????(0
1)?100???3?0????????1?????0???????????????????000?????????????????????????????????0?????0
?????0000?1?????????????????????????01???????000????????????0????????????????????????????????
??????10???N?N???0???????????????000
??1???{0 1}11--????0??????????0???????
Fukuivenator ???{1 2}101010?-111010001000?0-
????00?0?????0?0?0?11110??01?????10001???0???0?0?0????????0?02?{1 2}--(0 1)????(0 1)10100010000001{0
1}00{1 2 3 4 5 6 7}(1 2)0??10?1011?00???????01000?000000101?00?(0 1){0
1}0?000111???????????????????????????0?{0 1}0011?0????0????000000000?00?1003?0??10???0?{0 1}-
011001??0002101?01??????201001(1 2)?????001??00???0{0 1}0?00000???(0 1)00??0??200??1{0 1}???{1
2}110100?0???1?01?01000?????000?1?????(1

2)11??1001011??10??1000?0???1?01?0?002?00000??101???00?0?010???01???01??????0??0000?01?????001
000??00??0?000?0000????????????0??00?????1?1-
?000?????0???1?01????????1?1120?0010??00?????1???0??0??01????-00????????????????0{0 2}0?01(0
1)??00?0??????????-000000-00000???000???01000100--0?0?00000?0{0 1}0???????????00001000?0??00?0--
?011-?00100-?0??????10??0?1??
Nqwebasaurus ???????0??????0????1???????{1 2}???10???????0??2?{0
1}1000?01??????00000??????????????????????02?1--1??0???0?0(0 1)?1(0 1)00010?{0
1}?????????????????????????0100002{0 1}1000?100011000?0110?0??????????????????????1{1
2}110?0?00000020000011000000000020001????????01?????-
031?????0001?011????????100??000?????01??00?????0?0?0???1?11???0??001???21??{1
2}1?0??0???????01?0{2 3}0???????000?1?????2{1
2}1??10?0010001100100000?0??1?01100012?01000???01????0??0????0????01?0000??????1000?10????11??00
0??????000?00??000???????????1?1?000000?1?0???0????????????1??????0???1?-
?001??00?0??0??0???1??????????0???????????????1????????00??011?????0??????????-00??00-0100??-
???00??????0??00????0?0000??000???????????00????0?20??0?0{0 1}--0001-????????0???????0??0???0
Pelecanimimus ?002111NN0?-
?1??1??1???1?112100101100??NN?01001?00?NN???0?????000???0?00020{1 2}00?10??1?000232--
02101100100011-000010????????????????(0 1)11?NN0-0??N0???00?{0
1}0?1NNN130002111000??103000000?????0-
?2????1?000{1
2}?021?????????????????0????0101001???0?0????0?1?01???????0102121????10000101?0??01?0{1
2}0120N2000101??0000(1
2)?0?????10?00100113001?2???????????1???00?1??0??000NN?00?00??0?1??0?0?000???????????0?0110?00?
0????0??000011??000???????????????????????0???0000?0?0???????????0???1?1?1?????0?101?0?N0?00{1 2
3}?N000??0?????0?????0-0??????---?0N0??010?????0-?0?2??????00??0?-??01?1-??1?????01?01?00?0???-
N00000000??0???????????000??
Patagonykus
???
??1????0??1?01??11{0 1}????2{0
1}10?00?????????????????0011?????2101?1????10??120?????1????111??0??{0
2}????1?1011?1100?00000001?110110??0?0????????????????????????2???0??0??{0 1}10??????0??0??????{1
2}0???0?11??0????0?0?????10????0??????????????1121?????????????????0??????????????????????1?1???????0?
0?1????????10?????0?1?????{1 2 3}1?20?????????????00?????????0??11110?????10?1100?1???????00010??0??{1
2}011?1??10?????1{1
2}101?01??1?10?0?10??0??????????????0???1?????21??????????????????00?00??????????????????????????????
?????????0??011????????????????-0?0???-010??10110??11101011100--
?11???00?????000??00??0?100101010000??01?-????????????-???????????1110?00
Xiyunykus
???????0???01111000001??11??????????????????????00??????????????1000??????0??0????1??1?0??????????
??1?????00??(0 1)1101100??2????1??00{0 1}????????????0101?0{0
1}1?12?0??001?0?0???000000{0
1}00?0?????????????????0?{0 1}?02???1??000{0 1}1??????????????????{1
2}??????????1??????01?01?11??????????????????????????1?????????????????00??????????0??1??0??2?1??200101
?????????????????1?0?1????{2
3}??21?????11???0?0???0??????10?????????????????01?0?????00010????01?00?1?0?0????????????????????00
1?0?????-?0???????11?????11??0??1?????0??????????0??????????????????00????-0?????????1???????00?{0 1
2}0?0??????????????????-?1??00-0101?1-?11????1?00111??--?0??????????????????????????0?????????????????--
????-0?010?-?????????????0????
Bonapartenykus
???
??1????0???0????01{0 1}?0???????0????????????????0?01{0
1}11?????????????????????????????????11????????????1?2101?100????????0??????0????????????????????????
???????11?0???000????????????1??????????????0???????????0?0?0???????????????????????????2{1
2}1??????????????(0 1)????????????????????????{0
1}1??21??0???????????????????1???????????????????0?????00??????000??????????????0??1?????????????0000
?0???????????????????{0
1}?????00??0??????????????????????????????1?????2????????????????????0??0????????????????????????????
??????????????010?????????????????-?00000-
01000??????????????????????????????????1?0?????????1???????????01?????????????????????????????????1??0
?
Bannykus
?????????????1????00????????????????????????????????1????????????????????????????????????2????????????????
????????00??(0 1)11?1?{0 1}????01???1?10{1 2 3}0?10???????0?0??011?02000?0011{0 1 2}(0
1)0?111100???10?010????????????????????1{0 1}0000?0010001?11000000{0
1}0?????1?????????0???0???0100?1?????01?2????0???1??0?0?????????11??00??1??1?11?010????????????0???
??????11????????????????????????????????????1??221?????{0 1}0001?00??100?00?{0 1}?1?0?11??0{2
3}??21?0????1????00?0?0??????10???????0???11??0?????????10000????0{1 2}?01?0?01100?00??--
????1?00?001?0?0????????????0??????????????0???????????????0?????0?-
1???1??????????????????????????????????00??0?1?????????????????????????-0100??-??1?0????10010??--{0
1}???0000???00?0???-???0??00?1?????????0?{0 1}--????-??????-??????????????10???
Alvarezsaurus
???
???????1?0?10000??010?(1 2){0 1}1?0?1?10?????????????0??N?00???????????????10??1?0??{0
1}1010011?11???????????????????0{0 1}1000??0?0????1?NNNN000????0?1????????????????????3?00{1
2}??0?????????????00???1000??{0 1}0??????????????10???0?0????0???100??0??????????{1
2}1??????????????1????????????????????????2??????1??-??????????????0??0?????00???2{1

2}0?????1??1????N0??????00???????????1??0?????????????0????00??0?????????????0011??????0??1????
??0??00???????0???????0????010?????0000??0????00???0??010?
???????????????-??????-01-???????????????????????????????????N??????N?00?????0??{0 1}?000??{0 1}--
??11-???????????????000120??1

Achillesaurus

???
??????????????????????2{0 1}1??01??1?0?11-
2???????????????????0??????0????1100?0??1??????1?????????????????????????????????????0??????????
??????????????10????????0?0???00???0???????0?{1
2}1???{1 2
3}2??????????1?????0???????????????????1??0{1 2}-
00??????????????????????????????????????10???
??00???0???00?????0??{
1 2}0000???--????-???????????????????1????0

Alnashetri

???
?????????????1????????2?1??????????????0?????0????????{0 1}?????????????12??????????{0
1}??????????????{1 2 3}????{1 2}?{1 2}?111??1?1?000110100??11?????1???????????????????????????{1
2}????????????????????1???????????????0?????0?????????????0?????????{1
2}1???-?????????????????1?01?0???{2
3}?0????????1?????????????????????????????0??????0??????????????????????????????1???????????????0
???0??1????????????????????????0???????1?0??0?????
??????-????????????????-??-???0??????10000001??0--?01?-
????????????????01121??0

Albertonykus

???
??200????????0??1?0?????????????????????????
????????????????001???0??0?0{2
3}???0???
???????1???0??????????0????????1????{1 2
3}?0?????????????????????????0101000?????????000??00
??????0????????????????????????????????????1?????????????0???
???--20??????????????????????????????????000???--????--
?????-?????????0???1???

Ceratonykus ???????0?-??0?1??00?00???????0???1?11?-
101?0001?0?0??001110??????????????{1 2}?{1 2}?0??0??1??????????????????00?0?-
1???????????????1??????????{2 3}0????00{0
1}0?????????1?1?????????????????????1?????????????????????????1???????0??101110?03??????1??????????
??0?0?????????0??????????????0??0??????????11?????0???00?000?????????0??0???0??????1???????1??1
--1??1?????2?0?001??????2???????????1???????????????1?0?11???3?0????0???-????000??0{0
1}????0?????????????0?0?1?????0?????1??????????2?10???????????????????????????0???0??0??00??-????0?--
???0??0????1?1??10????0-?1?????0??????????0???0???0?????1??1?????1?0???????????{0
1}????00?????0?00??01????0?????????0???001??00?0{0 1}--?011-
??1??????????????00?????0

Qiupanykus

???
???????0?1??-????????120???0??00{0 1}???2???21-
2????????????????{1 2}?{1 2}?01??1?{0 1}?1??????110??3???????{1 2}??????????????????????{0
1}??????1??????????????????????????????????1{0
1}????????????????0??????????????????????????????1??
1?011????3{1
2}0?????1????????0????????????????????????0??0???????????1?????????????????????????1???????1?????0?
???1??0????????????????????????1????????0????????1???
??0??00???01??0?00??????????????{0 1}--
??11-????????????????0???????

Albinykus

???
???1????????????????
??????????????01????001111?0300{1 2 3}0001??????????????????????????????????????0??0(0
1)101001???????????????1??????0??????????0??0???????????1??
??????????????????????1?011100?320??0?????-
???????????????????????????1??0??{0
1}??????????????00000??0??????????????????????0?0???0?1111???????????????????????????????????????
???
1010100000{0 1}--0?11--????0??????????101????0

Xixianykus

???
??-?????????????10{0 1}?022010??1??0100??111-2020--20{0
1}2?2??-
0201100000100001111111??30?????1?????????????????0300????????0?????010010??0??????????????????1
0????????0?0??-???--
0???????0221??21??????????????????????02100111???32?????????-
?????0????????????????????????00?000?-
???????????0?1???????????????0?2?100000001100000?1?0????????0??0???0?????10????????0??0??????
??1???010??0??0??
?????????????0000-01011?0001?01?01000001--????-????????????????101???01

Linhenykus
??
??-????0?1??011?101???2{0 1}??001??????????20?????1??0?????0?111013410--120--???0?????-
2??????????{1 2}?????0??01?10???0011?11??0?0300??0?1????????????????03???1????0{0 1 2}0?2---
??0??0100??001?????11??0-
???0???0?0?0????????0??0??????1?2?1??????????????1??????????????????????12???????????1001----100--
??1?0111?0-3?0000???-0-????00?1??????????1??1?0??0?001000????????100000???????????0??0-
0??21????1?1011?00???0??0????????0???????1????010????1?011??0????00?0?????????????????????????
????????????0??010??????1????00???-???????????????????????100?100??????--1000-{0 1}-
0?0???0?????0001?00??000???--?011--????1???????????1?0?0???
Heptasteornis
??
??
?????????????1?1?0111111??
??????????????????????????????????1???
???-
??0??0??
??
?????????????????????????????1001000???????????????????????????00????0
Bradycneme
??
??
?????????????1?1?0101111??
??????????????????????????????????1???
???-
??0??0??
??
?????????????????????????????10?00000??????????????????????????0?????1
Parvicursor
??
??-?????????????10{0 1}????????01???1???{1
2}????1?????{0 1}2?{1 2 3}??-12?2-
010?010100011?110000300?0001?????????????????03??0??????????????01?0001001??0????????????????10????
?0???????-??0??0?????????-
1?????????????????????????????????????1??????????????????????1100111???3?0??0?????-
?????0?????????????????????1?-0??000?-?????????????????????????????????????-
?0?01?0000001?0??????????????????????1?????0?1111????????0??1????????????????????????????????????
??????010???0???111-
?0???1?1000110010000?00--?011-?????0??????????11??????
Tugrik_parvicursorine
??????????0??????????????????????1???????????????????????????????????????1?0?????0???0??????????????
??-??010(0 1)01(0 1)01?11001?2201?{0 1}?10101?0????????????????????????111013{0 1}112112000?{1
2}?1?1111-2021--2?120213-02????1?001{0 1}?0001000?0000300200?1??????????????0??3?0(0
2)1????????2??00100001001000????-011??0???10?????0????0??-?10--0?????2?02?1???0?????????1??{1
2}????????0?????????2?1?????????1?????00?00???21?0111000320??0??1??-????00?0????1?????????????1?-
0?00?0?-??????1????011?????????????0???0?2?00-
00??1??0000?01?00???????0??????00?????100??1?1111???????00??1????000??????????????????????0?----
??0??010??00??10?0??0000--
01??10???0??001??0000{0 1}--?011--0??????????????11?0?1?1?
Shuvuuia ?00????01-0-0010100000111110 10?1010111-10101001-0?101--110100010?--11-
?0110100000201 0??0232--0210-?10111110011110{0 1}??2201?0??010{0 1}00100?301-
000010002002101 1101??11??120??110101011-2021--20020213-
02???0100???1?0010101000030????01103??1100011000-?2?00{0 1}?-0010?02???110000??01??001??-
011?10??0100100000001011-?1?--011-1?2?0??1?0?0?0010?1-01?010??0020001011100??(1 2)???-11??-
0010???????00??{0 1}11?0111???3?00???01?0-0-00000??0011?00???????????-0??0?01?01???01???????-
01?0?0?10-00??0022??0-?0?????00???0?{0 1}00-00??-00-11?--0000?1120??1?1?111????0--00-
010000??00010?00000?-0100-001010????-100???10??00???0????0----000?00-01-001-??000???0?00??00--{0
1}?????10?????0??--??0?1?1?0?{0 1}000????0?00--?011--0110?-?0------11??2101?
Mononykus ???????????-
?0??1????0?1111?????????????????????????01???0?????100????????????????????????2??--0???-????101??-
1111000(0 1){3 4 5 6 7}201??01??????????301?0000100011021011113{0 1 2 3}11??120?????1????1-
2??????????2?????0110001111100111110000300?0001???????????????-
021?2???0000102?????0??010?0?000?????11??00???0?1?0???001?1????0?????????2??121???0?????????1???
?????????????1?????(1 2)11??11?1-00110110???00????10?11?0??{2 3}101-01??00-????00???0??0?00?-
10000?????0010101??????01100001??010100010-0000??2210????1011000???01?0?-
??????0???????10????20???1111?1?00????1??0???0????????????????????????1?????????00??010??0???100
??00???-?00000-00-0010111000-10-000100--100?10100000?000??01?????001100000000?01--0?11--??1??-
?????????11121??1
Martharaptor
??
??????????????01??{1 2}????????????????????????0??0??0???????0?????{0 1 2}0000(0
1)1000??????????????{1 2}?{0
2}??????????????????????????????000?0?0?0??1?????????????????????????????1?????????????????1????
??0??0????????0???10?1????0????
????00??????0??011??0??0????11???????0??????????????1????????????????????????????0?????????????
??????0?0?????????????????????????????????0???????????????0????????????0??????????????????????????

???????????????10????????????????-??????????10??????????????????????{0
1}?????????0??0??????????????????????0??1???-???????????????????0?0?
Falcarius ??0?1??01??001110{0 1}0?011(0
1)010????10?????????????000?0?????001100000?????2?000?0????????0?002110021101???00010(0
1)00101101?11?1000100{0 1}00???????11010010110100011000000(0 1)00001011010101111-
2001200021110101000000010001000?01?000000000?00?1????????0??10?1(0 1)021021??0?02011110000001{1
2}000??11???1001??000?0000?01011111(0 1)01011100??0?1{0
1}11121?????2????1?01????1?00????1??10???0221?01??{0 1}011111(0 1)0020000001-10{0
1}1000001102000?10000???00?0?00?0000010?0100?0?10100001101?010110110(0 1)000201?011011100101100O-
0010001000110O?00-?0?0?0?0?10?0?0?1?0021???0?1020?00???000?00??1?00??????0{1 2}0??????0?-00?0???--
-?0000?010??0??????????0?0-?0???0-0101?1011100??000000000--{0 1}?0?000001000000--
000?0000?1100?00000?00--?011-?0?11?-?0100000101?00000
Jianchangosaurus
?103000N???????????????????0?00?0???1???N??0?0??0????000??????????0???0?02100?0{0 1}???001-
0021000211?-0010?111(0 1)0?001{0 1}000?00000?1?????0???????000N000110?000??NN?0{2 3 4}(0
1)00?0100?11{1 2}{1 2}01?11-201110001?01000000010?0?????0????????N????0??0??012--?{1 2}???????100(1
2)0?11?00?02001??0000010??????{0 1}11??0?00?1001?1{0 1}0000???0?1?(0 1)0110?10?100{0 1}01?1{1
2}2?00000200?-???1?010100N20000?1???0-02{0
1}1??10??010??01??000?11?001?????????1?1??00??0?0???N000???????0??0???00?0??00???0?????10001000
011?010100??01?000?0?20000001100??000??11????0--?001??????????0?1010???????000-
0??00?00101NN000????????0???0???0??????---???001010?N00?0????????0?-?00?00-
010?01010?0N???0??0??00??{0 1}?{0 1}?N0000?0??NNN0-00N???00??{0 1}?????001???--????-??????-
???0?????????000??
Beipiaosaurus
?00????NN0??????????????????0?001011???0?N000000?1?0NN????0?????????????0021{0
1}00010???00?0031000211??00?01??0?0??01{0 1}??01{0 1}0?0?0?00{0 1}0?0N?????{0
1}001N0?1?10?00?1NNN0100000(0 1)000?010100?11-20001?0?{0 1}{0
1}0?????0?00?0?1000???????NNNN0?0?0?0???002??011?10??0{0 1}00????1?0000200{0
1}??000001?????11??10???0001001??{0 1}00?0?0?1?0?(0
1)0?0??10?1000?110?21?0?00?010?1?0110101?0N200001??00(0 1)002{1
2}??010?0011110100200?0112?1?1???0?002?1000???1?0??NN0000?10??????0???00?0?????0?0?????0?0?????
???00{1 2}?110???11101?01110?1??11?1?00???10000??0???000????1??0??0?111?0????0??00?0?000-
000?1NN100????????????????????1?0?---?0N00{0 1}?01?1?01????????000-000?00-
0001010???0N???0??0??00????0?N0000??0?NN?000?N??00???????0?00?00?--?01?-
10??????1100??0????0?0??
Alxasaurus
???2?021?0???????????0310102
110???????0??00?1100101{1 2}0?01?10010?1????????0?00?1{0 1}10010??000010(0 1)00?0101?020101?{1 2}11{1
2}?1?1?0??20???????2-??1?0?{0
1}?0????????000000??00???????????????10010010??0?01?0????011?0?100???(1
2)1?????00??001?1?0??0???101110?000100???????0?-???????????????1??{2 3}?????????????0???0211(0
1)?1??1010101100?00?11?1??0????????201011??10?????00?0????00?000????0100??01?000?????0?011???01
1?01?110?10??10111?210?-
??111110?011?1??0???????0??????????????10???1?1??0??0????00?00??1?0??????????????????????????---
???0??000??{1 2}0????????????-?0??0?-??01?1?????0???0?00?00??????-00000100000?-
?0?01???????????????????--??11-?????1??????????????0????
Enigmosaurus
???
?????????????????????10000?0???????????????????????????0?????????????????????2??121-20?2{1 2}100{0
1}0?100001???0?????????0??????101????????????0??
???????1?????????0????1?0?1-
?????????11???0??????????????{1 2}-
??????????2?????????????????????????000???????0?0?????????????????????1???????????????122210O?0
0?????????????????????????????????????1???????????????????0????????????????????????????????????
??????????0???00-
0000??0??0?
zheziangensis
???
???
??????????????????????????????0??0???0?121??????????
?????????????????????0???
??????0??1???0??1???
???
???
????????{1 2}1?????????????????????????
Suzhousaurus
???
??1????????????00011?100000{0 1}00??????????????0000{0 1}002110???????????????????020101021-
20111100{0 1}1020000{0
1}02000100??????????????????????????????????????00102???0?010?????00001?????????????0?????????1
0?0?0???10?1??110?0-
????????112?????????????????????????????????????010?10?001???????????????11???????????2?20??????0?
?????0??0???001??????????0???0?????????????010?(0 1)(0
1)1?12010000010???1222100101111O???????1??????????0???0???1?????21???????????0?????0??0????????
???????????????????????????????010?000????????????-?0??10-??01010?0?000-{0
1}01000100???????????????0000-000?0010001??0?

Neimongosaurus
??2??2??0???????????0?1000?
?11?1??00010000012?01?2??1??0011001??????000110102100??????????????????{0 1}1?110111-
2????????????????02-
00100???0?????0?000001001??1???????????????0110?20??00011?????000??02?0?????????0??????1?1{0
1}0??1?0?101110?10???1????-?11?-1?????????????1??2?????????????1????1201?0{0 1}0??0(0
1)??????????????1?00200001?021201????01????00??????00???????????0??00?000?????0?0100?001?0120(0
1)(0
1)010010???101111????1111101????1??0???????0????????????021?????100???0???000?????1?00?????????
???????????????0---???00?0?0?0?0?????????000-?0??10-??01000101000-001000000????{0
1}??????????00???0???00??1?1?????00000{0 1}--??11-?????????110-10?0??0????
Erliansaurus
??
????????????0?0???{0
1}?????????????????????????1?????20000?0??00?0000000100????110?21??????????????????02-
01?0001001000000??10001??0?????????????????01??{0 1}0??1{0
1}???????10?????00?????????????-1??{0
1}????00?1110010101111??0???????1???20?0??1??????00?0????00?1?????10??0??01??00?0???????????????
0101011001010 0?0??11????1111101111?0????????0??????????????????????????????0??0???1?????????
?????????????????????????????0???????????????????????????1?00101000-001000100--{0 1}0??0000??0{0
1}0?0???0???00??11{1 2}?0?0???0????????????????-???????????0????0
Therizinosaurus_minus_hindlimb
??
??0101100210 0001?0001100000 01110??????????????????????
???000??0??????????????????????0???00
?????0?1???1????????0??????????????????????????????0???????????????????????101101010110 0100??1?
??????????0???21?0??10????????0????0?11100?010????????????????????????????1001111101110????????
??????????????????????????????????1?????????????????????????0???1????????????????????????????????
??????????????0??????????-00??00-0000010?0100??0?10000???0{0 1}?{0 1}?0000??0{0
1}0??0????
Nanchao_embryos
?00010?NN0??0??0??00?1?????01?0??11???0NN002001?010???????????????????{1 2}??11?00?1??0001-00{0
1 2 3}{0 1}000?11?-?1000???000???2?????????????????1??????1?10N1?02{0 1}000????????{0 1 2}??0??1???{0
1}2?10?????????????{0 1}{0 1}??0??01????0??0?1?0????????N??????????002-??00????0?00?1??????0??{1
2}0?????0000??????????????????1??0??{0 1}0000??????01000?????10?{0 1 2}-{1 2}?1???10000?0??-
1?01???0000N2101101??00-112???10?1000??1?0????????1?0?1??????11?{0
1}??00??0?0??11?00?000?????????????0??1???????0???000?0?????10{0
1}??????????0????2???????????11?????????00--?00??1????100???101?0??????-
0??11?0?????01???00????00????-0??????????0????0?00???0?????????????000-00???0-
0101???????0?????????????????N0???????NN?????N???1???????????????--????-
???10??10?????0????0?0??
Nanshiungosaurus_brevispinus
??
????10???0101000011100??0???020100121-20-22?0??0-
2??00???11????????????????0001???????????????????
???1?????????0?2???10?00???????11???????????????1????????????????????211????????????????????
?1???????????2??????????????1???0???????????????0????????????????1??????1???????????????112211
011????????????????????????1?????????????111??????????(0
1)????0???0??????????????????????????????????0030?0?0???
????????????????????????0-00010???
bohlini
??
????1?1?0000?000??{1
2}??
??
????????????????????????????????1????????????????????2??
??????????1???????????????????????????????????????1???
??0???0????????????
??
??????????????????
AMNH6368
??
??????????????????????????{0
1}???????????????????????1100??????????00??011??
????????????????????????????????0?????{0 1
2}??????????????????????????????0????????????1??
??????????????????1????????0??1????????????????{1
2}??2?101?1110??????????????????????
???0??
?????????????????????????10?01?0??00?0?1??0??????????????0?????????????????????????????????????
???????????????????????
Segnosaurus
???21121000002??????0110002
10?????0???0?0???????10?00?????????????????0??11?11100100??????{0 1
2}???0?????020200111110122100110201001?2-
0010000000110100000001011??1??????????????10???0????000{1

2}?0????00001???0?01?????0????1??1?1?01?0?0?1012(0 1)0110?0-0???0-??11-
1????????0????1?03?????????????000?1???0?101{0
1}010??????10?????21002?000??021101????01???01?0?????00?1010?0?0??0?010?100?????000???????1??10
10101100???12221100111111111101111?1?????????1???0???10???021??1???00??0??????0?-
0??1?00????????????????????????00----??0??????????0??????????-?0??0?-0100010??1?00-{0 1}?10001??--
?0???????????0000-000?0?1????10000??000?--0011-??????-?????????0000??00
Erlikosaurus ?1011100100--1101-
1001?001101101010011100001001110001000001000000??0102002100001021001-003100(0 1)201?-
?????????0??????????????????????????????????????110???
?????????????????????11?000101{0 1}003--0200011-
010????????????????????????20?1?121???????01??11??0?1?0?01?1?10???0???01-0-1?????-01000010-
001???3011002100001100 1-12?????????0??????????????????{1
2}00?01?0?1111?0????01001?0??011????????????????????????1?10001 0??????????001001001 0?????????????
????????????1??-00--00?-11?11???000????101?0???00?-00??????00?00000000000000?01000-0011100----
000???????????????????????????????????00?11?00-
0010000???0011-??1100?0(0 1)?????????????0??
Nothronychus_mckinleyi ???????????0-1000-
10011101 0????????????????????????????????00100??????????????????????0?1000???1????000001(0
1)00?1{1 2}1??????{0 1}??????????????????0?0??121011001??????????????????????????0121100?10{1 2
3}???????????????1?00????0??{0 1}1???0??00?????????????-??0???????????{1
2}?0????????1??????12??????????{0 1}0????{0 1}??00?0111?1?????0-{0
2}??????????1??????????????1??3?????????????1?????2???????{1 2}?10?????????0??????0?0????1?{0
1}?110?????????????1???0??10?1010101????????1?1?1?11???01????????01010001010?????????001?????111
011????1?-?????????10?????-????????1??????00????00?-0??1?????????00????01?????????????????(0
1)0?0?0???0????????????????????????1?10111100-001000100--01{0 1}???????????000-
????????????????00???????0?????010?-?????????????????
graffami
???
??1??????????000?120010000000?01{1 2}0??0???????00(0 1)1012001100??00?1{0 1 2}0000010?002{0 1}200121-
2012210011010000{0 1}02-
001?0?10001?110000000?011?01?????????????????100010??0?01001???01?01120000??????0?01??00??10?0?0
?0?11?2??1?0?0-0????????1-1??????????????1???????????????????????2010??0?{1 2}000??100?2{0
1}0?0???100200001002110 10??1?1?????1?00???11010?00020?????10?010?????????1100???1?0{0
1}010001?10101122?1100111111111101111?10???????????0????????02????0?000???0?????0--
0??1??????????????????????????????????00?0?0??00?0??????????-?0??00-??01?10111000-001000100--
0?0?0000??0{0 1}?0000-000??010001?002?0000?{0 1}--?011-?????0-?????????00000?01
Protarchaeopteryx
???????0???????????????????????0??10?0????????00020{1 2}--
(0 1)100?(0 1)????00???0????2???????0????0{1 2}??????00?????01??????00??100100000000000{0 1}{1
2}???1????????{0 1}00???????2?10?1?000?00{0 1}???????0000??{0 1}?0000?100{0 1}0??????????1-01??0{0
1}?00??2011{0 1}000??00?2001?0{1 2}?11???00??1????01????0?????1000?010??????1{1
2}1??2???????????0???1?03?????????????????0012???10???110?101020011000?1?0?100002?0{0
1}?00?100????????0???????1????0?00?0001??????????000????????1?????????????10?0???????????11??010?
0??00???00????????????0?????????010?0??(0 1)????000?00??100?0?????????????????????????---
???0????0???0??-0????0???-??00??-??????????????????0??00????{0 1}?0000??0{0
1}0000????????????????????0100{0 1}--0?11-?0???????010??0?????????
Similicaudipteryx 010{1 2
3}000NN0??????????????????1?00??100??0NN?1202100?011000?0?????????????20120200?20??0001020{0 1}--
020??(0 1)0??10??000010?2?001?0?0??0002??1111{1 2}N?00(0 1)0010020000?1?1NN100(0
1)01110001010201???0000?100?2{0 1}11??100?1??11?00???010????00000?0100010020-000??0???1-0(1
2)1?10??01?101100000000?2001000?110??0?00001?1{0
1}100??0?0?????000010?0??0121122?01?10211?110011130100N{0 1}0111?0??0000(1 2)110020-
10111?100020010011-1?0110000200000000101?0???(0 1)?000?????1???0???0??0???1?(0
1)?0?????0???11?0????01?000??0???00?0?0??0-000??0???00??0{0
1}?0?00??000???????00???021??0010001??0-
?0?0??00?01??000????????0???0???????????????0N01001000?020??????????-?0??0?-0?0(0 1)?1-
???00???0??0???0??????N000??0{0 1}000N--?000??1?0?{1 2}????????0?0--0?11-?0?????0????-??????0??0?
Ganzhousaurus
??2?12020??00????????-????---
?????????????????????????0???01???
???????????????????????00000?010??{0 1}???????????????1??????{0
1}????????????????????1????0???????????0??0???2????0??????-
??????????0?????????????????0???????3??????????????010?-??????????????????????????????????100??{1
2}?0??0?????????0??0??????????????????????????????????0-
???0?????????????????????????1????0????????????????
??00??????????????????????????00----
??
00--?000-?????????????????????????
Caudipteryx 0101010NN0?????????0???????1?11?00(0 1)01?(0
1)NN11202100011100000?0??????????2012020(0 1)1001000011-----0---
1010?110?10000011000100?0000012101111NN??0001100200(0 1)001?NNN102000-0(0 1)00-0102(0
1)0?110011010002{0 1}1101000011011?0000001011NNNN0011020001002--01(0 1)11?1??1-020?10100101011---
000000100000(0 1)01101000001111102000?0?00-0--0(0 1)0010000101-1122100000{1 2}(0
1)?111001N030100N2001100?000--2110?21-001(0 1)0000(0 1)0200100-1-1?011000-21000000101?0??1N0000?(0
1)(0 1)0?01??0?0?00?00000?100?0???0--1????????01?000??0??100?011000-000?1100000?00-
00?10000000??0??000?00020111110000000-00000N00?01NN00(0 1)???????0????00?-?0?0?00----0N0030?00N?0-

0-0??{1 2 3}?N???-000?00-010001-?10?N???0?00??0(0 1)0?010?N00001010NNN-000N0000?0?0?000?00100{0 1}--
0011--0???0100?????0?00000000
Shanyangosaurus
??
???00?0????00???
?????????1??????0?????0?00???1????????????????????????????????1?1???????????0??0???1?????????????
???1??????????????????????????????1???21-
??1?????????????????1?0???????{1
2}?00??????11??????????????????????????????0??000??????????????????{0
1}?000?0?????????????????1??1?00????0???????????????????0??????????????0????????????????????????
???0?1-
????0???0??0??00????????????????????????????0??????????0???--????-?????????????????????????
Kol
??
??
????????????????????????00003001000 1???100000(0
1)0?????????????????????0???????????0??0??
???????????????????11000?3?0??0?????-
??
???????????????0??????1?0110??
??000--0001-
?????0???????????1???????
Avimimus ??0????010??0?00??0001?1???2011??00?1000?????21-01?01000?001(0 1)01(0
1)0??0??21120200?0??0111-??-????---0-10111101(0
1)001011102100??0??0????1???1?0?1?0110021010???11100{0 1 2}?????{0 1}???(0 2)1(0 2)200111-20?0{1
2}000?0?10101100(0 1)00(0 1)11(0 1)10001010111100301--001002-?010-000002?020??0??00?10{1
2}????00000011000-001??1?0?11{0 1}100(0 1)(0 1)21000000??0??110???0??00-??1221?????????-
10110130??1021011001000--221??1?0001?1????0???????1?100111--?3(1 2)021-?0?00-
00111?0?0000???1??????0??0?000?000?-00?0-?00000001?00000000110???10(0 1)(0 1)100-
?0101000000101?0???0--00001100-11000002(0 1)??1110110??0??1?0???000{1 2 3}11000??0?00?110??0-0-
???00?0111?0100?010????-?-?????????-?0??0?-0100?1-101000?000001000??0???????00???(0 1)(0 1)1--0000(0
1)?00(0 1)(0 1)000000110001----11--?0??1?0?????????1(0 1)00??00
Machairasaurus
??
???11000100000(0
1)1111???0?????????????????????????????????????11
100????????????010?????01??0???????????????????????????????????1?????????????????????????????????????
???????????????1?10001000100?????????00??????????????????0??????????0?00?0??????0?????????????
??????????????????100??0??????
???0?0000
00010??
Caenagnathasia_dentaries_only
??2?10020????????????-????--
??
???1???0
???2???????????-??????????{0 1 2}??0???-
???0-
??
??10??????????????????????????0111???
??
Hagryphus
??
???0100?1000?0001?1???????????????????????
?????????????????????????{0
1}????0??????????????????????????????????????1101??????????????0?????0???10?????????????????????????
??????1??11110010001?0??????????0?????????0???
??????0?????????????????00??????1????????????????????????????????00??????????????????????????????????
??
??????????????????????????????????????-0100000{0
1}0??0???
Apatoraptor
??0??1??01?0?{0
1}0?011??????????????10???0??00?1???????????????????111?010?100001??{0 1}??0?0??0??0{0
1}001?0011?0?1???????{0
1}??1?1?0???1?0001?0110?????????????
??1????00?0???????010???????????????????????1??????????????????1?03??????????????01??21?????11011
???1000?0111???????????0??????0?????????1(0
1)??00???0001??????????0????00??????????1????????01??0??00???00??????????????0????????????????
?1????????????????1?1??????0????0?0????1?????????????????????????????????????3?????????-???20????-
10000?-??01?1???1?????????????--?????100?00??????????1????0?{0 1}????????????????????????-
????????????0????
Leptorhynchos_gaddisi
??2??0??0????????????-????--
??
???????????????????????10?02??????????????????????{0
1}????????????????????????????????0???????????0???2???

????????????????0???-
??1?????1????????????????????????????????0-
??
??10??????????????????????????0111??
????????????????????????????????0?{1 2 3}???????-??????????????????????
Elmisaurus_rarus ???1-
11?0??????????????????2?????0????????????-????--?0?????????0?01?1{1 2}???1{1
2}???????????????????1??0??1???????0?0??20000?0{0 1}1??????????????????{0 2}0???{1 2 3}???{0 1}??{1
2}?111??0??00????0?(0 1)100200????1????????-?????{0 1}??{1 2}?????????????{1
2}100?????0100000?0?????0???100??02??0?0??????-???0??2????????2?1?????????????1???{2
3}?????????0???0???-1????????????1????0000110??110111?002?0??0???(0
1)011??????0????????????????????010000?????0-
???????????????????0?0??????0?1?0?10000???01?0???0???????????????????0???????0010???????0???0??
??10??????????????????????????0111??0???010?????0??????????-
????????????????????????????????????0?00???10??0???????????????????00100111?0(0 1)1--
???????1???????1???0???
elegans
??2?10020????????????-????--
??
???????????????00??0?01110020???001???????????????1???????????????????????????1?0010?10????????????0
???2?????0??????????0??1???{0 1 2}??????1?????????????????????????????????0???-
????????????????????????????110???2?0????????1???????????????????????????????????00?????0-
??????????????????????????????????????100???0??0????????????????????????0????11??(0
1)10?????????????????10??????????????????????0?0111???
??0??1000100001?011--????1??????????(0 1)0??????
Chirostenotes_sensu_lato ???01-0????-1?101-
101110111????11????????????????????????????01010???0?21000200000201??1---???--?0??????0?0(0
1)001?1{1 2}?0112?(0 1)000?????????????????0011????????0????{1 2}{0 1}000001111{0 1}{1 2}?211?{0 1}010(0
1)001000{2 3}11{0 1}0{1 2}010011011100?1000?010(0 1)000020000001????-???????0?1??{0 1}?010??0??{1 2}(0
1)?1??0000001100100l1??????0?00000?10210?000?0-0?-?100100?0?0?-?1?21-?-00?0?0????1?0{2 3}?1-
?????????1001--2????????0???0????(0 1)001(0
1)1?101001100000210??00??0?1???10100?0????1??????????0??0010?01101?1-
?1??????1?????????????0?0?2?0--(0 1)-000011??0???00-0?-??????0?010???0?0??021?11??0001-?0-
0??0???0????10??????01{1 2}???-0???????1?0101110??0??010??00?0??????????-?00000-
000????????????????????????????01?0???{0 1}0000-0-?0?000000(0 1)1??000?1000--0011-
0?011???????????1?0?00000
Anzu ???----
0??????1?????01?????01111?000?1?0?????????????????00010000?00???21001200?0020111-1---------?-
1?1?101?0001?12001{0 1}20?0??0011?-1???010?10?11?0200100????0??{0 1 2}00??0{0 1}1??02{0 1}{1
2}00????011010001{0 1}1101010?2-1111?02000001101(0 1)0??2?0?0???002--???????0?1-
010020??00?1011??000000????1011????0??0-1?0??10200?0?00?-{0 1}--10??10?00?0--?12-1-1-0-?0??-?0?10120--
??{1 2}?0???00?01--20(0 1)0?1?1?000??1????0001??1-
1?????????10110000?1?10111100?0?????000??021??0000?10?0??00??--?0???0???0?0?00000?0??1?0?{0 1}??1-
000??1?0000??0-?0???--??0?0??01??00???10?-1?1????--0-?0?(0
1)??00??101?01???????????????????0?00?01???0?0?3??0?000-0-
?????0????0??????0000?1?11110??0?00111????{0 1}10?0???0??{0 1}?000-
0?00?0?0?0???010???10???????????0???????????????0100000
Luoyanggia
??{0 1}???02??????????????-
????---??0{1
2}???0?1???01?1?0?2{1 2}1??{1 2
3}??0???0?0????????????????????
????2????0????{0 1}?-?0??10????0???1???????????????????3?????????????????-
???????????????????????????1????????????1?????????????????????????????????????0??????????????-
????????????????????????????1??1-
0?????????????????????????????????????20???????1????????????0????1??????????????????????????0???
??000--
????00??
Incisivosaurus ?1010110100-11101{0
1}00011101?1100100001?0?011202100?0110000001100101001121120100?001011000201--0110?(0
1)??
????????????????????????????????????00100000001?001-???????????????????????????????????????-
0??10??1?0?0?0??0?11???????0--
011?????1000000101100???0100020011100l?001??1????0100
??0??000???????????????????????1?000100??1??-100000?-
?0?0-???000????11??????00?10???????00?0100000000{0 1}010--00?0??010?100--
000???
????????????110???0?????????????0??
Microvenator
??1?02020????????????-????---
(0 1)??011?01?100?01210?1{0 1}??01??01?????????????0N10?2001001??N???{0 1 2}001?0{0
1}11?0102??11000??????????11{0 1}100?0101110?00?01010NN??????0?0?????????????????2?020??{0
1}??0??100????0000?0?????0?????0?00??0?1?1020????00?0?-??????????{0 1}???1221??????????????1?0{2
3}??????????????0???-2(1 2)1??????011?0???????000?{0
1}?1??????????1?10???????????N0?0??????110000020????00??000?????0-?(0
1)101000???10000000000??0?11--

??00??1??000000??00??????????????01?????201?1????????0????00?00??2?N0????????????????????????0--
-???00?0(0 1)0????????????????-000000-010??1-?100N0-0000111000001 0???00?????NNN??-
0??00000000000020000????????????????1???????????000?00
Beibeilong
???100?N????????????????????????111?01?0?N?1202?00?????????????????1???2102?2?0?0???11??1---???--
??????????1??????2??1?????????????????????????????{0 1}??{0
1}?????N?????????????120200??110??1???????1??110????1???????????????????????????0??-????0?????{1
2}?????????????1?0????000???????????????????????1?112?0??????-??-?00?????000?-
?????10000000???0?1??{1 2 3}?101?200?1???01??-?????{1 2}??1??0??????????????1??0????????{0
1}0????0?????1?N??0?0?????????????????0?????0?0?????0--
???????N????????????????0???????0??????000???-
???00????0???????10?????????1???????????1????????0??N?????????????????????1????0????0N???????????
???????????????????????0???????????????????????????N????????NNN??00N?0?????{0
1}??????????????????????????0??????????????1??
Gigantoraptor
???2112120000?001????-????---
????????????????????????0100011??????????01?0???2000001??00?000???01???11{0
1}???????????????????1011000021?1011010001011001001000?000{0 1}???????????????1?0{1
2}???0?????1?011??0?00??{0 1}00000{1 2}????0?00-0000??02?00?0??????-???0??0???0???0?-
1??????????????1??3???????????????11?-
???0?2??1?10??1????0???????1?0????0??1?1?00?1?1?????1?100????????00??0?1??0???1?0?1?1???0-
??????????010?0???1?0??0???1?????0?01??000??0??00?????????0?????????????0??1?11?0?????????0?????
1?10????????????????????????0?00--
???????????????0???????????????????????0?1?101?0??0????0???0020??0000???0?00??????????11??1?00{1
2}0??00??--0???-?0????0???????????00???00
Tongtianlong
?10?0??01???????????????????20?0?101?1?10011202100??01000?0?1????????????10{1 2}0201???????1-1--------
-?-1??0???1?0??????????2??0?????{0 1}???1??{1 2
3}012?00011?2100????????????????????????????????00100????{0 1}0{0 1
2}?0??????0??0????????00100?0????????000-?010?00???1-0{0 1 2}(0 1)????10?01???????????0?{2 3}???0?{1
2}?????????????00??20???0???-?--????????0??0--???????0?{0 1}?{0 1}10?-10???021??102?000100?1?--{1 2}{0
1}1?021?{0 1}01??????????????????1?0??0???2?00???0??1?0????1????010?1?????????????0?????????????1--
????????0?1000001?????????????-?0???????????0?-???00--00?0???????????00????1?1?00??-
??00?0???01010?001?????????????0?0?????????????001?{0 1 2}??????????????????0?0?00??0?-0?00?1-
??0????????0??00???????????????????-??????????????????????00?--??11-????????0?100?00????0??0?
Oviraptor
?1??0?10100??????????????1???10??1?0011???001202??0?111000000??01??0???121020201?00??11??1---???--
??????1???????????2?0??????????????????1????210?111?20000???1110000000001111???201?{1
2}???0?{0 1}?0?10?011?01??{0 1}0????001?{1
2}?01100?0???????????????????001??000??2?0????00-??-?1?????0--0?-
11????0?00010??10?1??211????2?100101?1?--?0??020-
10111?1000200?1000??????????0?2?0??00?00?111???10??0000??0?0??11??0????0??????0??0--
00??????0010????10???00?0??????????????????????-???00??0?0-
???11??0????10??1?1????????1000???0?101???01??0????10???000-?11?????????00???0?0???????????{1 2 3 4
5}??010-??????-??000???00????????0??00????{0
1}?0000??1?000???????0???????????????????????????????00101111????0?0??
Banji ?1100-1NN01?????????????????{1 2}0?1??????1??NN?120{1
2}????0N100??0???11?0????21020201?10???11-1---------?-
??
???????????????????????????????????001-001?0??????2-
??0??0???2?????????-?--????????0--0--?????-0-1001?0-10?????11-
1N2111?10??1?--
???1??N??????01???????????????????????????1?0--
???-???10--?0?-??????????????????1??????--
?110???????1???100?????????1????0????1?0?{1
2}??????1??
??0??
Yulong
?111011NN0??????????0????????1101?10????0NN012021?0?1NN00000?1??11??????21020201?0020111-1---------
?-????0??00?????2????????0????{0 1}{1 2}?????????????1?N??0200?21????N?000000(0
1)100?020??0????????????????????0?????????????00???0????N?NN?0100{1 2}0??1001-00100110 0?2-?????{0
1}?????1?0???00????03?????{0 1}????0??000??00?{0 1}2?0??0???-?--???0???0000--????110010211?-1001?1{2
3}0101N2111010??11--?????????1?10??????0{0 1}0?00{0 1}{0
1}?1?0?100??2100?000????1??N?11000010??1????01???0??100??0??0??0--
????????1?0???0????????0?????????????1?????0?0???-???10--00?0????????????10??1?1???0???-
?1?0?0??0?1?2NN001????????1????0????1??????????0N?????1??????????????N?????????????????????1N?????
?0??0???{0 1}???N??????????????????????????0????2?000??{0 1}????01????????????????????0????00?
Huananansaurus ?1100-1011???????????????????{1
2}011?100???00??1202????111000?0011?010????121020201?00??111-1---------?-
?01?1??10??????????????????????????1???????????????2100????????0000001111??????????????????????????
?????????????0?????????{0 1}??{0 1 2}????00100{0 1}-00?0??10??2-????????????{1
2}??110????????10?0??2?????????10?000??2?0??????-?--???0???00-0--?????-0-1001??-10?1??{2 3}11-
1?2111?101??1--2??????????1????01?1{0 1}00010???????1???0{1
2}?00??0?????11111?10??10????????????????????00??????1?0--0??????????{1
2}??????1???10??????????????????????????-???10--0??-????10???0?0??1-10?100?--?100?0???????2{1

2}000??????????????0????1?0??0????000??????????0????????????????????????????1?0??????????0??????0
0????0??????????????????????????????????01-???????0??????????????0??
Wulatelong ?11????010?????????????????{1
2}??11?0101?00??120????????000?0?1????????????1?????1??????11-1??????????-???????????1??2????{1
2}??0???0{0 1}{0 1}??????010????1{0 1}1??{0 1}???????00?0{0 1}0?{0 1}0010??100??1???1{0 1}?0?{1
2}???2?12?2?0????01???0??????????00??1????002000-??20??????{1 2}?0{0 1}???{0 1}?????{1
2}??2?000000?????00????????0??0???010???????????????0????00--0?-?1??????{0 1}?011?-
?0?????1????2?1???0????-??0??????10????????0{0 1}?10??0-1?0?10???2{1
2}0????0????111????0???00?????????????0?????????????????????0????0?????????????0?{0
1}?1?????0????????0???-???1?--0??-
???????????020??111?0???????0?????010????0??????????????0???????????????0???{1 2}??0???0?0-
????00?????0??0?-??01????????????????????????????????????00????0??0???????????????0??--??01-
???????0????????0???????
Corythoraptor
?11????01???????????????????101111????????????0??????????????????????????2?02?20???0????1-1---------?-
1???1???0001??{1 2}???020??0???????(0 1)??0?????????????2{0 1}0?0????00?0000000{0 1}101111????11?{0
1}?0?{1 2}?0?2{1 2}1101?00?2-01??0?1?0???????10?0{0 1}?0{1 2}0001001-??{1 2}0??????2-
???????????11011000 1?0??11000?1????0?01??100??{0 1}2?????0???-0--?0?001{0 2}0000--111-????1?01??-
1??1??31??????11??0??0?--2???????{0 1}??1??100?0000100{1 2}-10010??00220??00??1??1???(0
1)1?00??1???10?0?????????????0000?????0--1?????????????0??0??0100?????0?001?1???00????-?????--???-
????????????0??0?101100??-??0??(0 1)???01?2{1 2}0001???????????????????????????---
??010???0?????0??????????????????????????1?????1?0?????0???????{0 1}?{0
1}?00000?0?0??0??0??1??00001?????????00--?011-1????????????????????100?
NXMV
?????????0????????????????????????????1??????1????????????????????????????2101?2?1?00?011????-????--
???????????00??012?0??
??2??01??0
???2?0??????????????????????0???????????????????0?1??{1 2}?????2?1???0??01?-{1
2}01??10?1????????????????????????????????????0-
??00?0??1????????0????0??????
??0????????????1??????????????????????0?1???10???
???
Nomingia
??
??1????1?1??00010?210312000?001120-???0102001111001110002{1
2}1100?001{1
2}??11100100010??00?????????????????????????????010000??????1?????000011???????????10?????????1{0
1}????????0?0???00?10????????02?1??????????????11??????????????????????20(0
1)0??????????????????????1-1?????????1??????0???????01??0????????????????????0??01?0?0?????????(0
1)01(0
1)000?????????????????00110010100??1??000?01??0???????0???????0?????20????????????0????00??0??????
?????????????????????????????????0301000001 0??
????0000-00000???001?00200?1????????????0??????????????????0????00
Rinchenia ?1121100101?01?-?100-2-
?01?1011110001000001202100?111000000100110000112102020100020111-1---------1-
1011001100010 12?0102000100001001?110102100011011000???100??{0 1 2}0???0{0
1}1??0102001111 0????????????????01101000?0???????0?00?01?0??00?000--000001?002-
01?0?0???10100????001??????0??{1 2}?????0??100?00102000?????-0--?00????00-0--?1?2??0?1?011?-
1001?131??0020110101?11--(1 2){0 1}10?21-{0
1}010??????????00??1?1???1?00??201?0?01?1?111001100000 0??1??????????0??01?0???????10--
?1??0??0?010000000?????001210????1??1???00??1-?0?00--000-(0 1)??11010?00?111?11??????-?-
00?00???10{1 2 3}000010001-211????0???0110010---00?003010??00??-?????????-0?????-??0101-
??000???0?00??00??2?{0 1}???????????00???0?0000?00?1????????????--???1-?00????0?????????0??0?1??
Nankangia
??2?02020???????????????-????--
????????????????0?2?0?120??00???????????????00?11?00100??????????????????????0102010{0 1}11011110002{1
2}1201?00?1101?100??00111?00??????????????????????????????2?010??{0
1}???1011?????01000??????????????1???????0?1021?0???????{0 1}?-
?1??10????0???1221??????????????????3???????????????0??-?01??21-10?????????????????101?????????????{0
1}??????1??????1??0???001???????????0??00?0?0?????0-??0000???1010010?10?????0{0 1}?2010-
100??0??0???01?00???????0???????1?????21??1?????????0?????0??0???00??????????????????????????---
??????000???????????????????-00????-0101?1-?0?00??00??????????????????????????100-
0000?0???0?0?002???1?????????????????????????????010??01
IVPPV9608
??
???{0
1}???????????000???11??1???001???????????????
?????????????{0
1}?01100???????0????10?????01????????????0?????????????0??
????????{1 2}????2????00??1?0010????1???????????????00??00??1?????????0??????????????{1
2}??????0??????????????????????????1???????????0??
?????????????00??????{1 2
3}??1???
?0?0???{0 1}0??
IGM10042 ?11(0 2)1110101?11?-0000-2-21102011110?11?000(0
1)1202000?111000000110011000001210202010 0020111-1---------1-

10111011000101210112000100001001111?0101100111011000??100000000000111101021 01{1
2}1110201000111101100?2-010100000000001000000100?0001001-00100010?02-010?(0
2)0100101111100010000?20000?1????0000100000112100000000-0--110?10010-0--11221101100110-100100311{0
1}102111(0 1)101011--2010021-
1011101001200000010100110 0?0210100011011111011100000000100?0??????0000??000{0 1}?0?00--
?0000001001000001 0???000012100-00001100000??1-00?00--000-??00?000??0021?11?110110-
0100000?00101100010?01-2110???000-0110?10---0000?3010?0?0-?-?0??00010-00??00-??0111-
1?000??00??0??00????{0 1}?0000??1{0 1}0000--000?00000?000?1000?000--??01--0?1???0110100010010?001
Citipati ?111(0 1)11010100?1-0000-2-
2?102011110111?00011202100?11100000?1100100000121020201000101111-1---------?-1?1????1?{0
2}?1??2100????0??000100(0 1)11?010210?11?0(1 2)10100??1001000000011110{0 1}02011?111?1?100?1(0
1)11??00{0 1}?211011001?0?????011000110100010 00-00100010002-1???01001?1101(0 1)10000?0110?(0
1)002??????0010?000112?000000?-0--?00010000-0--1122?100100110-101110311010211111010 11--20?002?-
00001??0?(0 1)(0 1)00(0 1)(0 1)00(0 1)-1?00100002{0 1}000000111?1110(0
1)110?0000??1??0?011??0??0????0010010--????000?(0 1)01000(0 1)000???00?0{0 1}{1 2}00?-1???????010?0?-
00-00--000-10?0100?0000211?11110(0 1)00-?10000000010(1 2)000010001-2110--0000-0?10010---
000?030??00?010-0011??010-00?01?-???0?1-??0000-00?000?00??1?0?00?0??0100?0-
?000000000000000?00?00?--0000-?001???01101(0 1)0011???00??
Anomalipes
???
???
??????{1
2}?001??????010??0?00?01?????0??0?????????????????????1?
?????????????????????????????21??1?0
??????2?0????????1????????????????????????????01???0??11
11??0????1?0??????????????????????0?????1??0???
??0??????001??1--
????-0????????????????1???????
Shixinggia
???
??????????????????2??3120??00???210100110-
2???????????????????1000000?0?0??????????????????????????????????????1102???????1??????00?????????0
??????????????10??????????0???00???????????1??01?????????
????????????0??0????????10???????????????1????????????????????0?????00???????????????00???????????
?????0{0 1}11--
????????00?????????????????0???????0?????21??????????????????0?????????????????????????????????????
?????????10??00???00???-
?000??0??
Khaan
?1111110101???1???00???2?102011110011?00011202100?11101000?1010100????21020201?00?0111-1---------1-
10101??10?{0 2}101210??20?0??00010?(0 1)11101?1100111111000???1000100001110110102001111001010001{1
2}11010000110101 00?00000010000001002000 1001-00100011??2-020?(0 1)0110101(0 1)011110000001000(0 1)0(1
2)??????0010000010200 0?00??-0--?1001000--0--1(0 1)121100100110-1011?1(2 3)1101020110101?11--2110020-
1011101000 1000(0 1)(0 1)00-1000100002201?001?0110?10(0 1)110(0 1)0000??100010111?00?01?000?100?0--
?000000010100 0?01001?00?0??000-00(0 1)110?00?0?01-00?00--000-???11000?000101?1110000?-
010000?00010100001??01????????000-00?01{1 2}0---?00??30?00??0-0-0????0010-00???0-0101??-1?1??0-
00?00??00???1{0 1}?00000?110000--000?00?000100001000000--?001--?0??1?0?1010(0 1)0000?00000
Conchoraptor ?1101-10101????-0??0-2-(1
2)11010111100 11?00011202100?11100000010111?000112102020101020111-1---------1-10101?1100?1012?0{2
3}120?01?0?0100011?0?01100(0 1)1?021010?0?1000100{0 1}01(0 1)11100102010{1 2}111010100011110?00{0
1}?11010100{0 1}000??000?0000{0 1}00?0002000-00100011002-0???2{0 1}??????1?(0
1)11000?1000??000?1?????00?100000112?0?00000-0--?00?10000-0--1122?-0-10011?-100110311-002011(0
1)101?11--2???0???{0 1}?1110?0?0(0 1)0100000?1?0?10???21010?00????011011(0
1)000000?????????????0??0?100?{0 1}?0010--???????000?0?00?????000000?0??-??????0???0???-???00--000-
1001100??000100?1???00?--?110?0?0?010(1 2)000010001-2110-??000-0110??0---?000???0?????{1 2}?-
??????000-?????????00??????00???0??1??00--??{0 1}?0000??0??000--?00?00000000000?00?000--?001-??01??-
00?01000?0???01??
Jiangxisaurus ???????NN?0????-????-2-
???????1?10?????N??12????????????????????????0??21??0201?0?????1-1---------?-????1???0?????{1
2}???????0?0????0??11?N1?{0 1}10?N1??211?{0
1}?0??NN?1?00???1?????????1???-??????????{1
2}-?1??10110?0{1 2}?10????????????????????????011???????20?0?????-?--??????????0--???????????????-
?0?1?1{2 3}?????2??1??0??1?--(1
2)??00?0??01????1?????00?????????????????1??0??????111N1?????????????????????????????????????--
1???????0?2??0?0????00????????????????????????-?????--????0?????????????????1?1?????-
??10?0?N????1NN0????????????????????0?????????0??????????0??-???2??000?00??0?-
0101?1?????????????0????--????N?00??0???-0??10??0????0????
Nemegtomaia ?1000-10101-00?-1100-2-0???10111100111000(0
1)1201100?11100?00?101111000112102020100 0?0111-1---------?-101110110000??21?312???????????(0
1)???????????????101????10001{0 1}012?1111?01020?101110????{0 2}0???{1
2}???0??11????????????????????????0?????001-0010000??12-?{1 2}?0???????1?01??????0?????1??{1
2}??????001000011?2?0?0????-0--???????00-0--1?22?-0-200110-1001??{2 3}11-1020110101?11--
211?????1??01?1000001000?1?1???????0?{1 2}010??0?0??1111(0 1)?1000100?????????1??0??0???????0?00--
0????00???????00?0?1?000?{1 2}??00?0?0???????????-??-00--100-1???????0?0?????1??????--
?100?0???0102100010?01-21?0???000-01100{0 1}0---

?000???10??0???????????????????????????????????????1001110????2?0000??(0 1){0 1}0000?-
??010????????????????????????????????0?????????????0??
Heyuannia
???????0??????????????????????11?10?1????001?0?????1??00100?1?1111?????2112020100?011??1---???--
???1011101?0{0 2}00?12?03020?0??0011??11??01?110?111?1100110?11100{0 1 2}00{1 2}?1{1
2}00?010201121110101000111101000?1101000000???00?010000100{0 1}0001?0????????????2?0?00?{0
1}?1???1100???000?0?01?0?01??????01100001102100?0??????-??0010????0-?10?21?????????-
10?10?3?????2?11??01??1--21?002??100010100?001011?0-1?0?10?002{1
2}01?00??01?0??011000?11?0?1100?0010?0??0?10?0?????0-?0???0???1010000?1???10000?1???-
?00??1?0010?????0???--0???????1??????110??111000???0???00??10?01{1 2}00???????????????????21????--
-?0?1?1?????0?-0-??????010-??????-010001-??010???0??0??00--{0 1}0{0 1}?0000000{0 1}0000--
?0000??00??????????0??--??0{0 1}-??????-0?101000?0??0??0?
yanshini
?11?0?0010?????????????1110101111???1??0001??2100?111?0000?10011??????2102020100020111-1---------?-
10101111(0 1)(0 1)????2??(2 3)(1 2)10?01?0001001??1(0 1)1011000110010000?1110200000(0 1)10110102001{1
2}1110101000111101000 11(0 1)01110(0 1)0000001101000000?00010?{0 1}--0{0 1}0001?002-
???000?10?0110121000000010000?11????0011000?11210000000-0--1100100???0--10221?0???????-
???1003?????????????1?11--2??0021-1011101000001001 01-100110000210110(0 1)??0110?10(0
1)1?0?0??00?0001?011??00001?00001???0--???????0101001000010000001 1000-00011100010001-00???--
??0???0??000???0101?1?10011?-?-??000??0??1?00???????????????????11????---?000????????0-?-
??1?00000-?0?000-010100-100000-001011000--??{0 1}?0100??010000--000?0?000?0000000?0?00--0011-00????-
??10(0 1)00010000???0
Scansoriopteryx
???????NN0????????????????????????10?1?1NN?????1?01?N100100????????????200?0100010?000???N1{0
1}--?21??????????0?2??0?{0 1}??0{0 1}0001?00{1 2}111??N??????111N11?200100?11NN100000001100122{1
2}?0?2?1200{0 1}--210?0111?1??0?010000?0?00010NNNN000?0300?1???????0?1?1??1-
010012??01130011000000 0?30?1001?10?0?00-021??100?0??1?0??????1?0--
0???01?11?21?????????1?10????1?????2??1101??01?0{0 1}111?10-001(0
1)1010002N010002?1?0?1001021000010100???0??0000??0??0?000?0?0??0??0011?0???????1???????N?00?00?
??1-?00010?-??0-101??1???0??10?00?00??0?0???????00???010??1??0020??0??0???-
00?11?????????????????000-???0???????0N2??010??01?0??????????-???0??-??00?1-?100N??{0
1}0??1?100??{0 1}?{0 1}?N00000100NNN--??N0?0?01?{1 2}?{0 1}0??0000?0--??10-00?????0??????0????00?1?
Yi
?00????01???????????????????2?????????????0??????????????????????????????12?2???00??1000?201--
02???????????0???????????????????????1????????1?????2?0?0???????0000001?1?0????????????????????
???????????????0??????????????{0 1}??????1003??00???1???1-
???????????3?01100??????????????1????00??110???{0 1}?1????????1?????????0-{0
1}1?1???????0000??01??1??{2
3}01????0???00??00??2?????????1???100020000?0????0?1????3?000?1??1??1?????0????????1???????????
??????1?????0????????????1??0???????0?????????????????0?0????????0?????-
?????????????????1????????????????????{1
2}??000??????????????????????????????0???????????0?????????????????????0?1???1?0??????0??????????
?000??1{0 1}0????????????????????????0???????????????????????????????????
Epidexipteryx -10????N???????????????????{1 2}?????1?????NN??00{1
2}??0???N0???0?????????????012???00{0 1}0?010000201--121??(0 1)0?????????0???{0
1}?00??0?1????03??1NN?{1 2 3}N1?01?N0?0?{0 1}??0????NN?0{0 1}1?01(0 1){0 1}?10{1 2}22??1?2???001--2?0{0
1}0011?1???????0?????010??N?NN??0?0????10010??0??1????1-0(1 2)(0 1)0(0 1 2)(0
1)??0??2?1????0???00????????11?0???????11-{0 1}1100???0???0??1??--000-
012??2?1???0?00??0???1N110??0?200110?????00?11??11?0?11???0??2?0??0{0
1}2?1?0?10??13??00?10??0?0???N?000??????1?????????0??0????0?????0?1?0???????0???0???0????01????
?0-?00??1??000??01???00?0?0?-????-??0??0??0??1?1?????00?10???-
00??1???00????????????0????0???????????N??0??0?N00-0??????N?????0??10-
??000????????????10101????????N0???????NNN--??N0?0?????????????1???--????-????????0?????????0???0?
Pedopenna
??
??
???????????????0?010??1??{0 1}??{1
2}0020001??????????????????????????????????????300?001?111?????????????????1??????????0??????
???????1???1??????????????????????{0
1}?10?0?3?0??0???1???
?????????????????????????????????????0???10??
??00{1
2}???10?0--????-?????0?????????????????0
Caihong
0103?11010??????????????????11?0??0????00???2?2???????0?1???????????????{0 1}0110?00?{0 1}0??00000(0
1)10101???????????????????{0 1}?00????1????111?0000---??1??{0 1}??2{0 1}0?0????00?000?0000??0{1
2}12??1?????00?200?3?1111?2?????1??????????????????1?0{1
2}00?100300?10??????0?021?02??00?1?011?01???0?30?10???1111?000??00???0?0??0?????0?????00?000111
1?2???0000200?000??001011?02000?00?00110{1 2}101?1??0011??00102{0
1}01?02121?0110?00300??0001?0??00???00??01??????????????0-
????0???????0??????????????????01???00??{0 1}???0-?1?????????????0?0??0??-
??0?????????????????1?01?1??00101???-00?0200000??0?????????0??????????---?0???{0 1 2}??000?0-0----?--
?0?-?0??0?-0?00???????0???????????????{0 1}?0000??1{0 1}????--
???0????????????????????????1??0?????0??0????????0?10?
Serikornis 0??????0???????????????????{0
1}????????????????20??????????????????????????{0 1}?10??1????????0?021{0 1}--(0 1)12?????????????????{0

1}???????1???{1 2}011?0???---011101?0200100???00?000000010111111??1??????0?{1 2}?0?3{1 2}111{1
2}???????1??????0????????0001?010?01103???????????0-?{2 3}??22?10??2?111011?1?1?30?101{0
1}?111??0?????1???00?0?1????{0 1}0??0?000?????1{0
1}??????1???????0???10120????????0???0???02??1021?2011??10111{0 1}010021-
1?0?10000200??00?1?1?????0??00????1?1?????10??0-????1???????0??????????00{0 1}?010?00???00?{0 1}{0
1}1???1?1???????0????1?0??0?0??0????????0???020???10100??0??0???0?00??10000???????????????????
???0---??????0?0???0-?----?--011-?0??00-??0101-
?1?00??0?????????????0?0000??0??00?0???0????????????????0??--1?01-?0???1????00-?0????0010?
Anchiornis 110(2 3)(0 1)(0 1)10100?1?0?0?00?{0 1}?????1100??0(0 1)(0 1)?10000?(1
2)021001(0 1)11001010?01???????000(0 1)(0 1)001-?10?0000(0 1)021(0 1)--(0 1)021?00?101??000000?01001?10(1
2)10010111(0 1)000---0111(0 1)11120000??010000000000(0 1)(0 1)(0 1)1(0 1)1111111001(0 1)(0 1)110013{1 2}111(1
2)0(1 2)00(1 2)?(0 1)(0 1)00001?00001?0?000(0 1)(0 1)00(1 2)00011(0 1)(2 3)0(0 1)(0 1)110111??0-0(1 2)(0
1)00211010(2 3)0(0 1)1(0 1)0110(0 1)00?(1 2 3)00(0 1)00(0 1)11110001(0 1)000(0 1)1(0 1)00100001???0000000(0
1)0011?311112(1 2)1(0 1)(0 1)000(0 1)01?01001011010002(0 1)0010110-0(0 1)0(1 2)1110(1 2)(0 1)-101(0 1)10100(0
1)2101(0 1)(0 1)(1 2)10100010000(2 3)000000010(0 1)?000000-0?01(0 1)0101??(0 1)?0??0?0-?01?1?0?00(0
1)?0???0??0??(0 1)1(0 1)(0 1)?010?00??0(0 1)010{0 1}1--0-(0 1)10??0?(0 1)0(0 1)0?00100?00?00?0-
?0?11000??0020?1(0 1)(0 1)010(0 1 2)0000100000-(0 1)00(0 1)(1 2)00?00?00?0??00???0???00?0?00----0000(0
1)0?00000-0----?--000-00??10-0?0(0 1)?1-??100??{0 1}0??0??0?????0?0000000{0 1}0000---?000?000???0?{0
1}???(0 1)000--(0 1)?1(0 1)-?0???(0 1)?00100-10?0??00100
Aurornis ?0?3011010?????????????????2?????10-
1?000??2021?0?1??0?1?10????????????000011-?{1 2}0??000?02{0 1}{1 2}--
002???00?0??????????????00???01??0{1 2}011?010?---0?1?01??2?0?0???10000010??0{0 1}1??011{0
1}000????02110003{1 2}0112?2??{1 2}????????????????0?000?10010001102?00{0 1}1?1?1??1-0{2
3}0?02?1???2?010??110?0?101000{0 1}?1??0?0100{1 2}10??{0 1}000????0??0(0 1)??1?000?10?31111?2?00000{0
1}0???1-01011011002100101?0-100{1 2}211021?1?101?10013{0 1}010??111?0?11?0?3100?000101?00??0?-
?0?10???1???????1?0-????1???????0?????????1101???0??????0?10?1??0-
0?????????????1?0?00??0?0????01?10??0?20??0001?????0101???000?02?0?00??0?????0???0?????????0?----
?0??0??0????-0----?--0?0-0?????-???1?0???1????????0??00????{0 1}?0000??0{0 1}0000--
??000???1??????????000--0?{0 1}{0 1}-?0???0?10?10-10???????0?
Eosinopteryx 0?0????NN00????????0???????0??0??1?0????N??20{1
2}??0?1??0???1????????0?1000000?1-?????0?000211--?02???0?????????????{0 1}?????0?1??01031?1NN1---
0111N1?12{0 1}0?0??01NN100(0 1)01100011111{0 1}11?{1 2}?0{0 1}01010003{1 2}1{1 2}1{1 2
3}?2????0????0???????????N000?0200010020?001?11???0-130??2??0112?01000111?0?20?10?(0
1)?110??000?{0 1}00?1{0 1}0100?0?0??00??1?000????31?11?{1 2}?????????1?01???N110???N2101101??-
?002211021-10101?10012{0 1}0?0002-1?0?10??03000?000101?000?N0-01?1????1??0????0?1-
????1???????0?0??????????{0 1}?0???????000??{0 1}2??0-
0?????????????????10???01???????????0020??0?N101??00??1??0-00?02NN000??0?????0???0??????????---
????00??00?0?-0----?--??0-?00?1?-0101??-???????????01???????{0 1}?N000??0?0NNN--
???0???????????????00?--1?11-?0??????0??0???????0????
Ostromia
???
??????????????????{0 1}???????????????????1????????????????????00?0{0 1
2}00?00???1??????????????????????111?2?????1??????0????????{0 1}????0{2 3}0??{0 1}?????????????????0{1
2}?????????{1 2 3}?{0 1}{1 2}??0??????{2 3}0????{1
2}?11?0?00?????????????0??????????0???
???1??????1?????1?0?1????{2
3}?0???0???????????????????????????????1???????????????????????????????0??????????1???????
????????????????????????????????????1?0???????????????{1 2
3}?????????????????????????????????????0?0?????0???
00?????????????????????0?{0 1}????????????????1{0 1}-?????0??????????????0?0?
Archaeopteryx 1102(0 1)11NN000?0110100001111100001?111110NN010001001(0
1)NN001010110011?110100010?1-01010000002(0 1)1--(0 1)111?0001011?001000?1?0(0 1){0 1}00?(1
2)100102110NN1---0111(0 1)111200000101NN000(0 1)00000(0 1)(0 1)(0 1)(1 2)221110100{0 1}(1 2)0(0 1)20(0 1)0(2
3)2111(1 2)1(1 2)1?111(0 1)0000{1 2}0001010NNN10010020001103(0 1)01100111000-02(0 1)0(1 2)21101130(0
1)110011101?(2 3)0010111110(0 1)000101(0 1)001001001010010(0 1)00(0 1)000(0 1)011131111(1 2)21(0
1)000010(0 1)001001N0101(0 1)0N2000101?0-0002(1 2)11011-10111010(0 1)0(1 2)(0 1)01(0 1)(0 1)2(1 2)-1?011(0
1)0(0 1)0(2 3)00100001011000NN0-010(0 1)(0 1)0111?0000??000-0010(0 1)0001010(0 1)?1?0?????(0 1)1(0 1)100(0
1)0(0 1)00-0010101(1 2)--(0 1)0(0 1)10??001000?0?110-000000(0 1)0?00??0(0 1)00?1(0 1)2(0 1)?10(0 1)N10(0 1
2)000(0 1)101100-0000(1 2)NN000000101100?-0000-0010000----0N0000100(0 1)00-0----?--000-0000(0 1)0-010101-
1000N0-00?0(0 1)(0 1)(0 1)0001010-N(0 1)00010(0 1)0NNN00-0N00000(0 1)01000{1 2}000000000010--0110(0
1)110100-10100000000
Shanag ?1?{1
2}010?????????????????????0????0?????????????????????????????????????0?000?0??????????0(1
2)10101020??
???{2 3}?-?????????{0 1}-
??0???0????????0?00???????11??11?????10110{1
2}0??????????010?????0????0???0???
???????????????????????1?0??1???????????????????????
???1???????10?-0???????????000?????????????????????0?---
???
???????????????????????????????1??
BYU2023
???
???
??????2-
110???1????

?????????????????????????-
???
??10???????0???????????????????
???
???
?
ISMDVP09
????0?1??01??001?
?0????????????????????1{1 2}??{0 1}1????{0 1}1?????????????????{0 1}?0{0 1}?0??????{0 1
2}???0?010??NNNN00100??001????0??????????-
?????1???????????0??????101?????????????????????????0???0?0????1??0??????????1???0??0?????????
0???1110??3?0??0?????1?????0??????0??1?0??????????
????????????0??????????????????????????????????0???0??0??????????????????????1?????0?1100?0
?1????0??0N??????
?????--{0 1}?????????????????????????????0??{1 2}0000????01?10-?????0-?????????0??????1
Halszkaraptor ???101?010?????????0???????10010110??1010?2020?0?10100101-
?0?001?????00010110?00??100002{1 2}1--(0 1)120?100?-0??010?01?0?011???10?-{1 2}0{0 1
2}1?1???00??????????{0 1}??????????00?{0 1}??????0{0 1}{1 2}22110{0 1}11{0 1}0?{0
1}??0??????????010?00??0{1 2}??0???????{0 1}??{0 1}?02000{0 1}1031?1?00100??0-031??0?????1?0{0
1}1??011???1100??(0
1)???????01???011?0?0??0???0000?1?1???1100110??2110100001?01001??1010??2?001001001002101????1?1
0???00?{1 2}0????0{0
1}?1?0?1?00?2001?0001??????01000??00????????0????00????0?????100?000????????????0??0???0?0??1??
????0??0????0?0?1?0?0000-?0?1??110?????0?????011100?0??01?0?????010000???0?????0-????????????00---
-00001?10?0?0????????0???????????????1?????????????????????{0 1}?{0 1}??0????1??????-
0??10??0?1??????00??????1?10-?0???0?10???????0???????
Mahakala
???????0??????1????????1110???????????????????1000??????0???0000110????????????????????02?1--
1??01??0?00?1000011?01011001010010{0 1}00??????????????????00??1?10000{0 1 2}0?100{0 1}?1101?111111-
2?????????????????12-100001?0?01011010001000?0001????????01?????-030011?????1?11???000??0?010?0{0
1}0????000??00??10{0 1}????0??0?00??001??0????????1?-
1????????????01??1??????????1??10????2?0???????10??00002{0
1}00?1?1?1?011?0?1210??00?10?1????10?0??{0 1}?00?111??1?1??0??01?000{0
1}??1?????????01??0????0??????00?00100????0011?00?0?00?00??0????0???????0????0101??0??100?0????
??0?0???{2 3}??????0?????0????????0?1?????????00?{0
1}010?000?????????????????????0?0???000???0?????????201?00??0??{0
1}000???0???00???1?00000000??0--0011-00???0??0??????0110????0
Ningyuansaurus
???????01???{0 1}??{0
1}???????????0?021?--??????00??????-?????0????????1???{0 1}{2 3}1??1??00???1?01??200?{0 1}???100?0{0
1 2}000?0{0 1}1??{0 1}10??1?????00?1?0?3{1 2}1??{1 2
3}?100??0????0???010??0??0??1?0?0111????????????????-
0????1?????1001???100?0?????0?2?1????01???0???{0 1}?????0????????0??10?????1??1?{1
2}1??????????1???0?3???????0??0????????????????1?101?10??20001??1-1?0?10?0?2{0
1}0??00?????????????0?????????????????00?00?1?????????0?????????????????0????0???1??0-
??0??1???????0????????????????????????02??????1?00?????0??1???0??1???0????????????????????????????
?0??????0?0??-0-?????0??????????????0?????????????????????????00??????0000--
??0????????????????10??--??{0 1}1-?????????????????????0???
Austroraptor ???{2 3}00001???????????????????????????1?????00110{1
2}010101???????????????????0010001?0?0??????0221--
11111??1?0011000111111?????????????????????????????????1100{0 1}1???0???{0 1
2}0000011?????????????????????????????????{1 2}1?1??0??0?01?1?0000111?0????20???0??00?????0-0{1
2}??????????1?1??????????21????{1 2}??????0???0?0??{0 1}0?????????1?00???(1 2)???01-
?11???210010000???1?01??0?11???0?00????0??02?1???????11?0????????11???1?0?1???0{1
2}?010???????????0??0??00?????001010?????0??000?????00110000?????{0
1}?010000?01?????????????????????????1???00?????-???????????1????????1??110-
0??0??0???1?00??????????????????????????0---?000??0000????0?????????????????????????-
??100??00???1?00????0???00?????????????????????????????????????0{0 1}-1????0??0????????????00??
Buitreraptor
?00301?010??????????????????????100???????????00?110???0111?1??????????110?1??00??????0221--
1110??0010?11?0001111101100?110000{1 2}111??????(0 1)1110110200 10?1?100000000?0111?12{1 2}(1
2)11111101021?01321111120?11100000{0 1}00010101100 10100{2 3}01110????0?1?1?1??0-0(1 2 3)1022?101120(0
1)111011000?1010?111???01001?00??(0 1)(0 1)01?0?0?0000000111112??01?{0 1}?1?2100000{1
2}0???10?1?1001000200010??001??211?121-
101110101020?0111101001 1000?3001000?1?110?000000??01010011???????1?0100010?1?????01001000?10{1
2}0010010?0010100?000-010?1001000000?00??0????????011?0??0012????1?11{0 1}11(0
1)0?01000??00?1???00????????????????0??0?0????-00000000?000?0???????01(0 1)-000000-000101-
?0000??00000100001111?0000??0?0000-00?0100000010001000 1??0--0011--0???01?01000(0 1)0101000000
Pamparaptor
???
???
?????????????????????????{0 1}10{1
2}0???111??301?????????????????????????0???????????1??0???
???110???2?0?????????????
???
???????????1?????0??10??

???11-
?????????????????1???????
Ornithodesmus
???
??????????????????????11101??
??2?0???
???
??1????????????????????????
???
????????????0???
??????????????????????????????????
Pyroraptor
???00??101?
?0?????????????????????1????????1?????????????????????????????0?1??????????????????????????????????????
??????????????????????????0000??0??11???????????????????1???????????????0?????????????1??????????????????
????{0
1}??????????00???1??0??1????????????????
??010???{1
2}?????????????????0??????????1??0?0????????????????????0??
???????????????????????????????????0??1???0??????0?0???0?
??00?10???-?01?-
?????0???????????1???????
Rahonavis
???
??1????????????{0 1}111{1 2}?01110?110010{1 2 3}11?????????11?0??0????011?????????????????122111011002-
1200?32011111102-010001{0 1}000101001001101O?0111?????????????????0210(1
2)2???1?301????011010200??1??11?01?????????11???0?0?00?0??0101111????????1121????????????????????
????????????????(0 1)11?21-1??0???????????????1-
10001001?200101??1?11?????0??0???01?01011101??1?100?010?????????0?1??01?1?????1?010???000100000
00?1000000?00?00???????0???0???10???020???0?10(0 1)0????????00?10??{1 2
3}??????????????????????????????????????001??00??????????????0???????????00???????????101000001110?
????????00000000?00000010001?000?00--1011-00???01????????1101???00
Unenlagia_sensu_lato
???
??1????????????{0 1}?112?011101???????1??????????10?0??0(0 1)(0 1)0(0 1)??????????00??0????121(1
2)11011002{0 1}1100?32?1(0 1)0121000111100?0001?1100?{0
1}11100?0111????????????????????011??????1120?????010000(1 2)010?1{1
2}1???1??????????10???0?0??0?0??010(1 2)?00????????1221??01??21-
1?1?????????????1??1?10011000?{2 3}00100????1??????????1???00????????????(0
1)?001?100???????????????00??010010111?????1001000-
000100111???00?0?????????0???0???11???121???1?11????0?????????0????????????????????????????????????
??????000??0??????????????-??????????10(0 1)-100000-001????00????0???????????000-
0000?000000100?10001???--0011-?????0???????????101?0?00
Dakotaraptor
???0??101?
???????????????????{1 2}?????????????{1 2}2???????????????????????2001?0???00?1{2 3
4}?????1???????????????????????????????????0101010?0??00101101000??0???111??????????????????1???????????
?100???????????????????111????00???0???0{0
1}???????????0????????????????????21???11??1???????????????1
?01?????{1
2}??0??0??????????????0??????????01?101?????01?0?0?1?????0????????????1???0?01????????????????0?01??0
???00?0?????????????????????????????????1???0??0??????0?1???2???
???????????????????????????????????1?0????????0??????0?{0 1}?????0??{0 1}???????????????00??00????0???-
-??{1 2}{0 1}-??????????????????01????0
Mei ?(0 1)0{1
2}0??010?????????0???????0100??101???0010102110110101?010001??110?????0?0(0 1)0{0 1}100?1?1100002{1 2
3}1--0?2?-001101111020100010O???01100201111011???11(0 1)101102001011?1000000000010110122111?10{0
1}111200031111?1000111100000100101?(0 1)100001(0 1)0200011020?(0 1)000110??(0 1)-01(0 1)1(0
1)2?100?100210010001?0?100?01????100?0000?110100?0?0000(0 1)?10111??1(0 1)0{0 1
2}1111221?0?00101?0111101201?002(0 1)01101??0?10201?021?1011??100020000001?1?0?11000300100001(0
1)110?0?00000?0?001101000210?0??01?001?0?1?0?00????001101001O1?(0 1)0??10101(0 1)(0
1)0??0?0??100000?00200?0000-0001?0?1000??0?211?1??1?00?00?01000-0000200000????????00?????0?-
00?0???---?000?{0 1}0100?00-0???0???000-000000-0?0001-1?0000-
00?00100101210?00000?1?0000000100100O??00002?11?000--0011--0????1101000111??0000?0
Xiaotingia
?0?01110???????????????????????1?????1?0????0?010???0????????001?????????????0?000?0??2??000??020{1 2}--
022???0110???1?{0 2}?????2(0 1)00??00?10??????10?0---?1110??120010???1000000000001111?2??1102?0{0
1}?222000?{1 2}-{1 2}??1{1 2}??????1??0?1???????????{0 1}??{1 2 3}00{2 3}0101????000????1??1-0{1
2}1?0??1?1?2?11210??1?101010?12?1????00-000??110?10?0?0?101??0?100???0?1111?{1
2}?00?????0????0?1??201000200?10??????0?{1 2}1?021-0?111000002{0 1}000121?10{0 1}?1?000{2
3}000000???0?0??100?0???????11??0???????0-?0????1??????0?1????????1001?110??0???101{0 1}{0
1}???000?0??1??000??0????10???0?0???1???0??0????????01?01-0?10?1??-
001?1??000??????????????????00??????????00??0????0?0?0----?--001?????????????1010??1?00-
?0?00?00???2?0?0000??1{0 1}0?000???00?0??0?1???????????????{0 1}11-0????0????00??0?????00??
Sinovenator ?10311101?0001010-(0
1)0000111110001?0?0?0000102021101?11001000110001110011001O1101(1 2)10100000(1

2)211?002011?0101?1110?001{0 1}?0010001100201111????????1110111?0000???10000?00000{0
1}00001?211011001-220003111121(1 2)0?1111000001001101?(0 1)0000110???1011020100?(0 1)000111-
021102??0101?01???10101010100?1????000?100001110000(0
1)00?00000010210010001121221000002000010011?1201000200010110010021(0 1)??21-10101????????000?2-
110111??030010?001?0100010000?0??0011010002???1?001?001000000?000000001?0??110??111?1010120?010
0001?000?0?0020?-100?1000100?10110??0101?11?1100?00101?0???000300000000?00000?-0000-00100?0---
?0000{0 1 2}0100?01?0??????????-000000-
0100010??100???0?00?1????????0????????00000010?0000010000000?0?0--?011-000100100????????0000?001
Jianianhualong ??0{1 2 3}1?00100??????????????????????000?1??001?20{1
2}??00011???0?????????1?0??{0 1}0010010021?1????01{1 2}11?002?????1????0??????0?0???0?11??{1 2}011(0
1)000?---??1?01??20000??11001000000001000????????????1-?200?3{1 2}1{1
2}??1?0???0????0?????????????00001?020??1????-??0?1?1??1-0{2 3}1??2??0??1?011001?101?1?0?10??110?(0
1)00??0?0???01?0?0?????0?00?{1 2}10?00?011111??00000{0
1}010?1??1?1201000200?1?0??00??0211??11?1011?1?00?2001112?-1?0?101002?0??0001?0?0???(0
1)?00??00????????????0??-
1??0????????0?????????????????????0??010???0???0??????????????01?0??0???0?1???????????1?1???1?0?000
00?-0??0?-00001??0????0????????????????????0????-?0?0???0?????0----?--0?0-00??00-
??0???????1?0????????????????{0 1}?0000?01{0 1}00?000???0??0?????????????0?0--??11-
?0??????01?????????000??
IGM100140
??
??????????????????????????????20??1??????????????????????1??100100000000000??????????????????????
?????????????????????????{0 1}00{1 2}?0??100??????????????????????{1
2}???????01000??????10???021????10???10???????????????????????1????????????????????????????????????
??????????????????????????1?1???2001112?????11??003?0??0??11??????????0??????????1001?0???????1??
?????????????????????????????000???????????????????????0?????????????????????0?????1?11?0??????
????1???
?????{0 1}?0000?0?{0 1}0??????????????????????????????011-00???1??????????01???0???
IGM100972_100974 ?002100NN??1-0001-11111011010010???1???N102021-
00???????????1?1001100000010000??1????0?0N11--
0020??
?????????????????????????????????0?????103?-10?0??????1-
???0???0?????????0?11????????000011?????000001010?1?0????
0100N210(0
1)1???10??0??0????0?0???0???0?????????????????????????0?
0??0?0???2??0?0???0?0????????????1????11???????10?-
0???????00?N?000??0001?0??-0??????1??0????-
?N??
????????????110??????????????????0??
Almas
?002010NN0?0??11?????011???1100??11?1??N01?202??0?1?1001????1??0??0???0?00??0??2??0?000021{1 2}-
-0020??????????????????????????{0 1}?1?{1 2}??1????1??11{1 2}????{0
1}???01?20002{1 2}1{1 2}?{1 2}001???{1 2}1?1010?00??1?N1?N??20?????11030-100?1????{0 1}-?????{1
2}??????0?????0??00?????0?????????????1??0?1?0?0????????0(0
1)11???010010?11??1?100000100?010????1010002000101?0??00???0??????????????????????????1-
11011????3???????01???00???0????00???????????????0?????00?1?01?1????????????????????????????????0-
00???????0???0?10?-10??-0?0???-??????0??????????????00?-01??0??001?NN000????0?0?0?-00???????0???---
?0N????????????0??NNN--
????0?00100?????01?0??--?????????1???10??????????????00?
Jinfengopteryx ?0?3?000?????????????????{1
2}??????0??????????0??102100??01????1???????0??????0??101?0??1?10?000211--(0
1)020??1????????????0?????????????1??0212110001---?111111?200?{0 1}???10000100000(0 1)000?1{1
2}??1???????0?2?0???11???10?????????0?????????????{0 1}??{0 1 2}?0??0010021-00???11??0-0?(0
1)??2?001?2011-0?0?101????????{1 2}?110?1011?{0 1}??????0?0?????????0?10???1?00-
0121?????001000??0??1101101000?00?10???0?00?(0 1 2)?1?21-0011???0002{0 1}000121?1?0?1???0{2
3}??????0?1?1?0?????000????????????????1??-
?0????1??????0?1??????????01?00???1???00????????1??????????0????1?????0???00-
???????0??????????1?01???0?0-0????-000?100?00??0??????0????????????????---??0?01??0?0?1-0----?--???-
000?0?-00010?-???00??????????????????{0 1}?0000??0{0 1}0000?????0??0??????????????1??????????-
?0???????0?0???0????0????
Liaoningvenator ??0200101-0????1???0000??11{1 2}0?01010?1?00???2?{1 2}0-
00011??10??110????0??000000010?{1
2}0??????0112100020??00111??00001??0?0??????1??020???0????????1110111210101????????0000?0{0
1}1??010211????{0 1}?121?0?{1 2 3}{1 2}11???1??01100??0???0????1?00??100????11?2?0?01-10???0-03(0
1)?02??0001?01{0 1}??0?-?0?111?00????????0????0?1001?0?0???0001?11{1 2}010???012?0?{1
2}1000?0?0???1??1??1011002?0110?1?0??0211??2111010???00?20000?{1 2}1-
1?0111??03100?000??1?00??1??00?10???1??????????0??????0????????0????????001?01?110??0???0010?{0
1}???-?????????10?0?200?00????0???????0?0???02-
101??100??00101?00?00?01??000?0??0???0????????????????????100?1??000?0?0???????????-?1??0?-
??01010?????????0??????0??????0??0???????000?-???00?0???1?1????????0??--??{0 1}1-
?????0?10???????10??001??
IGM1001128 ?1030010??0--10?1-1000111110?00101?01100010202010??11--
1010000000????0000000(0 1)0?11?0000?0232--
0220????????????????????0???011?020??1?????????????????????????1001000?0100011???2?11{1 2}11?{0
1}01100?{2 3}11112121?1?1?0001?????????11?00120020?02102?010?01011?0-
????02???????002100?10?000111??1????010010{0 1}0011?0?0?100????(0

1)11112100000011 1??2?00110101?0?10???101000210110110000 0???????????01?00002001?12?-
1?011??00300??0?010??0000?000?0?????????0?0??1????0??0??0?01?0?1???????1?????????????0010?110?-
????????010???1??-00???0?010?-??0?0020?01??1100?0?101??0?0001?00000??0?00?00?-0101-0010?00----
10??????????0???0?0000??02000?--000?0?000?{0
1}?????11????--0?11-0?1101?10???????1????00?
IGM10044 ???????????0-01?0-
1?????0????????????????????????????????0?01?0???????????????10?11???????1?10????????010???0??????
???10000{0 1 2}00000{0
1}000??{0 1}01{1 2 3}00{1 2}0001?????????????0?{0 1}-
???????????{1 2}0?1?????????110???{0
1}??????0??000????0?0?100??0?00???2??????0?????????????????????????????????????10???????????????
?101000?00?00???????1?001{2
3}?0??0???0?????0??00?0????????????????????0?????0????0????????????????????010?????????????????
????????-
????????1???1??????0????11?1100??01???????????????????????
??0000000{0 1}0?????????????????????????????????11-
0????0??????????0???????
Daliansaurus ?0?{1 2
3}??10?0???????????????????1??10??0???????????????????????????????????????0001???1??0?000111000020
?1???11??100?11??000???01???2011?01?????????????20??0????0??(0 1)0000?0100?0111011?1???0?2?{0
1}???1???????2?211?????10????0??00??0020??2103???0???????0-
0?1?01?????1?010??00????1100?02??????01??{0
1}???110?0??0????001?011???????111??21?0???????0???1??10????2?00????0??102??0??????111?10002001
1121?1?0110?0(0
1)3?0???0010??????1??0???????1?????????0?????0??????????????0001??2??10?00??10010???0?????????
??0???100????0??0????????????????00?1?00?0(0 1)?0??00?????1???00?????????????????????0????-
0???00??0??0???????????????????????????????0??0??????????{0 1}???0000001{0 1}??0???0?010???1?{0
1}???????????{0 1}--??01-?0???0?????????1????0???
Tochisaurus
??
??
?????????????????????????0001200{0
1}???2???0?????????????????????????0?????????????0??????
??111???3??????????????????
??
????????0????????10??
??--?001--
????????????????1???????
Hesperornithoides
???3111???????????????????????????10???01??0110????????????????1????????????0?{0 1}0?10?????????0(0
1)10100???1??1?01??000???1{1 2}1???????????11??1(0 1)??????0???????2{0 1}0000??10000{0
1}000001000?????1?1????????????????????????????1?0001100001?0000??100{1 2}0?01??????{0 1}???????{0
1}1?????{1 2}?????{1
2}00??????????1???0???1????10???0?0???0??????1?0?00?11???0???2?11???1100001?1?????1??101100{1
2}?00?0??00???2???0?????1010??00????10{1 2}??11{0 1}11?0?1{2
3}?01?0?01??10???1?00??????1???00021?????0??000?1?????00????00?102001????10000??????????????000
0000?110??????0?0???00?????0????11??????00?10?000?0?0?????0??????????????????0?0???????0?0???10
?????0???????000????0???01???1-1?1000-00?0?1???????0??00001??0????????????000{0 1}100200000?0--
00?1-?0???????0??-101????????
Sinusonasus
?0030110????????????????????1100?????????01021???????????????????????????0?000?0????????0001{1 2
3}11000??????????????????????0????21??21101??0(0
1)02?00?{1 2}1????0??2-?????0??0???11??10??2?02010?001??01???????{0 1}-
????01?????????????????0?011?00{0 1}1????0??????0???0????0?????0????110?11-?11???-
?0000000???????0?2010002000????????????0???????????????????????1?011?01?3?0??0?01???0????0?????
?????????????????????0???????0?0?????????????????????????????0-0????0???00????00??????0?-
??????????0??????0???0?00??0??????00????000???????????????????????????????????????0-
0??000--????????????{0 1}??????0??--00??-
?0???0????????????????0??
Xixiasaurus
?003011????????????????????111010???????00021????????????????????????0?1??{1 2}??0????????0002{1 2
3}1--00?0?1??{0 1 2
3}00??0?0???010000???????{0 1}-
?????????????0????????????????????????1?????1???{0 1}????????0?01???????100?11?????000{0
1}010??0???????01100?0?01???0??10???????????0???00??0?01??????????????????????0??0?????????????
??????????????????????????0?0???{1
2}?????10?0?0??????????0????????????00?10??????????00100??0????????????????????---
??00????0??????????????????????????
??????????????????????????????00??
Byronosaurus
?0030??????1?01?0??????1111010010???????01021?0???????????????1?100?????0000001????????000231--
002?10?0?01?110001010????{0
1}??????20???0?{1
2}?0?????????0??????????02??11?????1?0-
???????????????????????0?0?????????????????0???0???1?0??0?0????2????1-
111?????00000001?0??0????012002000???100?102??0????0????0???

0????0?????????????????????00?1?0??100????????????????????????????????????00??????2??0??00?0?-
??????????0????01??????10??0??0????00?00000??0???????????????1??00----
??0??010??1??????????
???????????1101????????????????0??
Sinornithoides ?003011010?1???00??1??1????0?001?00?1?0001?{1
2}?2??0?1??00101????????????00010?10?21?0000001{1 2 3}101002????0?011??0001??{1
2}??1??0?01?0211111011---?1?00110100000??10000000000101021221?011-2010200?211112110?2-
210??0?000???1010001{1 2}00201011020100???????0-1???1(1 2)??0011?0100010000?001(0 1)00(1
2)1???01011?{0 1}00?1101?0?0?000001?00210?0?-
0011?2210000000??010???120110?2000001?00?002??00111?011111000200(0 1)002?-
1?01110003001000010010???(0 1)000????00?0010?0?10?0-
?0??000?????0000?????????0100?0?0???10?0?2000-00??1?000?0?00200??0??0??-
???????????????10101?00?0?101?00-00??10000?????????????0??????1???0---?000??0?0???0?0----?--?1?-
000?01-010?01-?1100??0010?1?????{0 1}?0?0000??000000--00??000????000{1 2}?00?000--0002-
00?1?111??00??0??0?0011?
IVPPV11119
???0{0
1}???
????????????????????????????????0{0 1}00{1 2
3}????002???????????????????????????????????????10?????????????????????????0?????0???????????
???11????{2
3}?0??0??
?????????????????????????????0???????1???
???1?
-????????????????0???????
Gobivenator ?0?301101000-1100-11101000-
??00100001100010212100?11100100011110010000?0000010?1110000?02{2 3}1--0??01??010111100{0
2}1011?11100111?02011100??????01101012000?0????????????????{0
1}??1011011101110202111010000?1111000?1100?110010001200{2 3}0??20?{2 3}??1?0011??10-
120?01??0001?1????00000?01??00?????11??10???0110100100000?01?0??0000-
00?1?1221000001010?1001?1101100200010110000??2110?21?101???????????????1-
?001110??320100?01?110000(0
1)00??0000??????????????0?????0????0?0??00???0????0?0?00??0??????0?1100-01???????00?????0-
10??0000?1?????00010{0 1}10?1???10?000?01?0?-?010?0??001000010?0???00?-
0?10???????000?10?0?000?0????????????00??00-0101?????100??0??00????--??????????????000--
01??0???0??0001????00?--0?01-?00??1-00??????00100?000
Urbacodon_mandibular_only
??1?000?1????????????23{0 1}--
0020???
??1-
???0???0??????????01??????????????????????????????????
??????????????0???0??
??????????0?0??0
??????????????00?????????????????????????0---
???
???????????????????????????????????
Geminiraptor
???3001??????????????????????????1??0???????
?0??
???????????????????????????????????????1??
?????????????????0???????0-?????????00000{1
2}0????????????11???0?0???
???
???????????0??10???
???0??
Borogovia
???
???
??????????????????010?101?{0 1}00{1 2 3}0???102??0111??(0
1)0??????????????????????0???????????2???????????????1???
?????????????????????11????{2
3}?0???0???0
?0?0??0??????????????????????1?????0?1101??
???0?1?000???????11
--????1??????????????????0
Saurornithoides ?0031000??01-??00-111011?11010010???1??????{1
2}??????????????1?0???0???00110?00?101?1????000131000020???1?0??11?00101{0
1}??1100??1??20??0101000{2 3}011??11{0
1}?11110??????????????10?0{1 2 3}0010?020020-10?????1?1-
01?01???????????????????0?01???0???????????????0??10????00?00?01???{1
2}10?10?0?1??22?00000?01?0???1??1011002000?0?1?0?102?10???????????????????????-1?0?1?00?{2
3}00??0?0????000?000??0??????????????????????????1???0??00???????????????????????????{0 1}{1 2}??0-
00?01??????????2??0??00?0????0???????002???10???0??0?-0??0???010?00?00?0000??00?????????0?0?00----
?????010??00??000--
??????0????????????????--??0{0 1}--?0101??????????0????0??

Zanabazar ?003000010?1-1100-
11101001101101?01?0??0010012000?0110??????111001????1?0(0
1)0?1???1????00013100002?1??????????????????11100010?21?11??????????????????????????????????????
????????????????????????????????????0?0?010110110??20?????20020-00?000?111-
???001??????????????????????????????1??????00??0???1?1??0?01???????00-
?11????10000001?01?0???101100200010?000?10???0????????????????????????????1?1??????????01??10???
?0???000??????????????????????11010000???????1????????????????????????????????????0?20011000?0?-
?1??????0?0????1???????00?-0???0???1??00000????01{1 2}???-00??????1??00----
00????????00??001{1
2}000??00--??????0010???1??????0?00??0?0
Troodon_sensu_lato ??03?0001??1-1100-1110100110???10?0?????0002120(0
1)0001101?0??11(0 1)0001??0?1?0(0 1)001????????0?0(0 1)3101002011???01111(0 1)001011111(1
2)00??10021??11???????1110?1120?1001?100?1{0 1 2}0???0{0 1}???011211?0110000100 1?111011(0 1)?011{1
2}1000(0 1)10001?1(0 1)0(0 1)100(0 1)20020102?0??-??1(0 1)00?1110?1?0(0 1){1 2}??0??110?????(0
1)000?0101?011???000???00(0 1)?1(0 1)0??0101?000010(0 1)021000--?01?12-10?00000???1?0(0 1)??{0
1}0?1?????010?100??02{0 1}1??21?101110?????{0 1}??????1-1?011100?210200??1?01????(0
1)0??10000??011??0{1 2}1??0??????0(0 1)1100?0100??????1??2?010?1???0??1011100-
0?0?1???000?0??0?11000??0-?1????000??0{1 2}?????1?1000?00-???00??0????0(0 1)0?????01{1 2}???-
00????01??0?----0000?010???0?0??????????-?0????-0?0(0 1)??-??0?0???0??0??000001??0?00?1???(0 1)00--
010?0?0????10010001??0--100(0 1)--?01011?1???????(0 1)00???01
Talos
???
?????????????????1?{0 1}????{0 1}??????2????????????????????????01?????????????????????????????1{0
1}01?00{1 2}?????11{0 1}???11????1{0 1}1?01011001000{1 2}10?0002??????????????????1???{1
2}????????????????00?1111?0{0
1}???????????????1?????0???????????2100?????????21????????????????????????????????????01????????
1??????????????????11100?3?0??0?????1????????????????1?10?0{1
2}?????????????????????00???????????????????????????-
????????00??0??0?????????????0????????0?????1?1100??????????00???????????????????????????????????
??????010??0?1??????????????0-
0??????0????1??00??10?0--1001-?????1??????????10?????0
Tamarro_insperatus
???
???
?????????????????????????{0
1}0?2??0
???11????3????????????
???
???????????????0????????1??
???00--
?0???????????????????????????
Philovenator
???
???
?????0{1
2}01101?1?1?0101011100120020 1?1???01110000????????????????1?
????0???????????2??0?????????21???100
11100?3?0??0?????????????????????????????????????10?00??1???00
10?00???00?0?????????????????????0?????1?1000???
???0?0?00000000010--
0011--????1??????????000????0
Linhevenator
????0??0????????????????????????1?0?0???000?21??10??1101?????0?00??1????0??????????????0?01?{0
1}000????1??0?0??02101?????????????????????????????????1{1
2}?0??????????????10?10?????????????0??20020102????????01?????-01?????????10?????????0?1111?0{1
2}0???????????????1???0?0???0?0????1100??-
?0???????0?000?1???0????1???002?0?10?1??1??????????0?1??????????????????011011?{2 3}?0{1
2}00?1????00??????????0????????????????????????1???0?0?0?????0????0??100??????????-
???????????????????010?0?-
???????????0???????1?0?10??01?????01?????00?????????????0???????????????00?????1??????????????????
??????????0??????00????????1?00???????????????????-????????????????????????--100{0 1}-
?????1??1???????1????0??
Tianyuraptor ???{2 3}011010????????????????????0???00????0??{1
2}0???0?0?1?0?????????????????0110?0??????00000010 10111???0???0???0?????{0
1}?001?0?1??01211?????01?0?1?0111200?001?101000000000 1112111111{1 2}01{0 1}01?1?00311111?1{0 1}?2-{1
2}10??????010??0?00?11?????010???0?{0 1}???????{0 1}10{1 2}1002?000110012??0?000??0100?2????0?00??{0
1}00?1{0 1}0110?0?0??00??0??11??0??0?112-110?00?????1??1?110?00?{1 2}0?0?0?????00111?021?101(0
1)1?10?02{0 1}000112-1?0010??0200{0
1}0?01110????????01?0????????????10?00?01???0?????0?0?0??????001??1???0???00??{0 1}1--0-
??0??1??000??0?????0????0????????0??0010????1?0{1
2}???010??0??00?01??000????????????????????????????00??{0 1 2}?10?000?0???1{0 1 2}?0000-00??0?-
??01????????0?????00?0???1??{0 1}?0000??0??000---00???0?0?0???????10??--??10-??????????00-
?0?0??0?00?
Zhenyuanlong 10030110????????????????????1?00???????1001020{1
2}1100?11???????????????????0??00?00?11??0?0100?0101??0?000????10?0????{0
1}?01??0?1??0?2?1??11001???1?0???200?0???100000(0 1)000010100210?1??10001?{0 1}?0021111012{0

1}02??10??01?00?0??0?001?1?010101002?0?{0 1}?11????1?12??111????1?112?00010??1011?0(0
1)?11?1?0010010??{0 1}0?1??1????00?1011??10-2?1112-110100001?0??????101100{1
2}00010???0110?2?0????1?111?10102{0 1}010110-
1?0?100002000??0?1???0?????000??????1????0???010?????00?????0???????00???{0
1}???0?????010???1110?01??1???000?0?10???0???10-
??????????0?1??1?011?10100100???00001100000?????????????????0?0??0?????0??{0 1}??0?000?0-
???200???????????????0???????????????1??00????1-0000??0{0 1}0000?-010?0?0?0?????{1 2}?001?00--00{0 1}1-
?1???1?0??????????????10?
Acheroraptor ???2011????????????????????{1
2}???1?????????????????????????????????????0?100?0??????????0010101110???????????????????????
???
???????????????1?????????{0
1}1???????????????????????????????????????1???0????????0?00???????10???{0
1}???????10010??????????????1?????0????0???0??
????????????????????????????????000???1????????????
??????????????????????1(0 1)?00???????????00???????????????????????0---
???
????????????????????????????????0??
Bambiraptor ?002(0 1)11NN0?-1??111000002010{1
2}10010000110NN11202110?11N0100100?000?1100?00100?00?211100010(0 1)1010111(0
1)1??1100?1102011121001100110012111????N100111N{0
1}11200100101NN000000001110010211011001002000(2 3){0 1}111112002-
1100001000101?NNNN11100?0111002001110??100{0
1}101102211010210020100100130111011???11001100001101000000000001(0 1)0110?00?{0 1}011122110(0
1)001011010?0?110100N11001011001102011011-201111100020011111-
1001000002001000011010??NN00000110?1100010211?1000110001?0??0100???00N10101100?0100101001000-
010010?0000?00101-000?000?110110000?012011?1110(0 1)0000101100?000?1NN000?0??0000??-000?-0100100--
--0N0000000N00?0-0?0{2 3}0N000-?0?000-??000001110N0-00100100111{0 1}1{0 1}?N00000000NNN-
000N?000000110000001000--0?01-000110110100-10?0??0?(0 1)0?
Variraptor
???
??0???????????????12??01100?0??12011101111???????????
???0?0???????????????01??????????????????????
??1????????????0???10????????????0???01??????????????????????
1???????????0????????????????????????????????????1???????????????????????1????????????????100100?
?????????????????????????0???????1??????0??
???????000??(0
1)??00???0?0?00??????????????????????
????????????????????????
Sinornithosaurus ??01111010?????????0?0???1?{1
2}?00???001?00010201110101N0??0010??0????00?0110011??11?100010(0 1 2)101(0 1)111(0 1)?1???1?110(0
1)1{0 1}11?{0 1}100100?200012?1?(0 1)111010011101112001001?100100000001010{0
1}112?1110011022?0031111112001?110??0???0101101001010010101?03111{0 1}111?1??0(0 1)?{2
3}1022111112?2021111111?3011111111?0?0011100?100100?0??00000110211?0-01(0
1)11112111100101101011?11011002000101?0011011?1021-1011?010001101110{1 2}-
??01??000200000001111000000001?0100?10100?2?0?1?00??????1??000(0 1)0???????1110010001??0101001--
01010??0??????00101??00000?0??011??1?00?20?11011?001(0
1)0101000??000100000??000000????0?0?0100000----0000{0 1 2}010?000?0?1??3?0010-000?00-01010?-?01?00-
00??0????11????000000??000000-0??000????0001???100{0 1}--0011--1???1?01100-10?10000110
Microraptor 1002111010?????????????{1 2}???(0 1)10010(0 1)001?000102021?0??1100?0(0
1)1?0000??1???00(0 1)001(0 1)00?1?100010(0 1 2)10(0 1)0(0 1)1101101?01110?{0 2}0011(0 1)?001000210012(1
2)11111(0 1)(0 1)1?011111112001001?100(0 1)0000000111(0 1)112211110110(0 1)22000(2 3)112111(1 2)(0
1)?01111?00(0 1)000101?111(0 1)1010020101(0 1)0(2 3)10(0 1)(0 1)0?110??(0 1)(0 1)1(1 2)1122111112?(1 2)021(0
1)10011?30111111111101010?000?100100?(0 1)?00000011(0 1)2(0 1)100(0 1)001111(1 2)1110110{1 2}0(0
1)10101101101(0 1)002000101100?(0 1)02111011-(1 2)01(0 1)1010(0 1)0(0 1 2)1(0 1)11100-1?0110000300100001(0
1)01000000000?000111010002?(0 1)210001?101{0 1}1?1?(0 1)0(0 1)100?0??1(0 1)01?01000(0 1)??0(0 1)01000--(0
1)0010?1101000?0010??100000001??01?10??0020??1(0 1)111(0 1)?00(0 1)10100000000(1
2)00000????????????000-?????00----000000100000-0-000(1 2)00000-00000?-0?000(0 1)-?00110-00?010?001?{0
1}?{0 1}100(0 1)001000(0 1)0000-0000000???00020001000--00(0 1)(0 1 2)-?(0 1)?11111?100-10?100001(0 1)0
Zhongjianosaurus
???
??????????1??1?1??{0
1}?0?????2?0??211??11?01?0?111111200?0???11110?00??0?0??????????????????????????????????{1
2}11?00????????1111002??????1??????????????????31?(0
2)2?11??2020?????????????1??1??????01??00???1?????0??????????????1??????????????????????????0?????
?????????????????110????011??1???????11????1?0?10???3?000?0?????????10??????0111??1??{1
2}11??0?01?0?????????????00?0???1?0?11??????0?????????????0??1?0??0??0????????????????????????0????
??1?00???0??????10???2????????????????????????????????????0?0?0???0-?-???3?{0 1}010-????0?-
0?0??0?????1??????1???????????0??0??????????????????0?1?????????0?????11-??????????00-?0?0??00???
osmolskae ???2011?????????????????????????10???????0?1{1
2}??01?01????0???
??0?
?????????-???0????????00???{0
1}?????10100{0
1}?????????????11?????0???
??????????????????????????0???1???????????????????????

???1???????00?10??
??0??
NGMC91 ??02???NN???????????????????2??0??00?????N??20{1
2}??0?0??????0??????????????0100{0 1}00?{0 1}1??0?0?011?10111???0????????00??????{0 1 2}??????????2{2
3}1??11????1?1?N11?200?00??1NN10000000011?????????????????????????????????1???0????????N??N??{1
2 3}?010101002??10???????0-?2??{1
2}2?1?1?2?10201??????30110111110??001?1?0???0?0??0?????0????1???0??{1 2}1?1????10??0{0
1}01?01???N1101{0 1}?N2000?01??0?10{0 1}???021-
10111?10001100110??1?0?100003?0?0000101?0?????00???1???1???????0????0????0?????0??????????(0
1)?1??1???1???10?????????????????0?0?0??0???0??????????????0????????1111????0??010???0???1???00??
???????????????????????????0?{0 1}???????-0???????000-?0????-
??00?0?????N??????0???????1??N000??0?0??????????????????????????10??--????-?0????????00??0?????????
Changyuraptor
???00?0???????1????0000101011
1??????????????00????????????2????221??111???0?1??????00?00??100?00000000?1????????????????{1
2}00?2??{1 2}11?2??????????0????????0??{0 1}??1????00{1 2}???????????????{0 1}1??????2?1???20{0
1}02?????1??20111?1?111??00??0?0???0????0?????00???0???????0{0
1}1??????????????0????0?2?????????????????0????10?1???111?100???11?10??1?0?10??03?0???0?1?1??????
??0??????????????????????0??1????????????????????1?????????1???10??????????1???????00???1????????????
??????????????????11?0??0???????0?0??1?0?0??????????????????????????---?????{0 1}???????-
0???????0?0?????????????0??????????????????????????0000??0??0??????????????????????????0??????11-
?????0???100-?????????1?
Graciliraptor
???0(0
2)?0101??????????????????????????????????(1 2)2??1????????????????200?011?100100000?(0
1)0001??????????????????????????????????????0?0?0101011?{0 1}10{1
2}0???1?1??????????????????1?????2??????2?10200?????1301??????????101??00??????????0?????00???2??????
?1{0 1}1???1????????????????????1???????????????????????????????1110100011?011??1??01????{2 3}?0{0
1}??0?10??????????0????????110010210???????1?0?0?????0???????????????000101?10?????????????????00???
0?10???????????????????????0?????0?1000?0?????????????1??
??????????????????????????????????0?????0????11?{0 1}???0000??0{0
1}0??????????????????00000?00???????21-0????0??????????1110?????1
Yurgovuchia
???
??1???10000?-00111010?????{0 1}0???{1 2}???{0 1}1{0
1}????0??011?{1
2}00?2?1010100???1????????????????0?0????????????
?1??????????1?????????????{0 1}???0??1??????????12?????????????????0???????????????????????1{0
1}?????????????????????????1-
??????????0????????????????0??????????????????????0??????????????????0????00??????????????????{0
1}1???-
?0????????????????????????????????????201??????????0????10??0???????????????????????????????????
???0??010???0??000-
0?00?00???1??????0?
Deinonychus ?0020110100?1?1??00????{1 2}?10{1
2}10?10000011001120110111100100010000011100100100000121?1000000101(0 1)111011?110001100011121(0
1)11{0 1}00010012111111????1110111(1 2)10100101000(0 1)0000001011121211111(0 1)00011001311{2
3}0011011111000010001010000011110?01(0 1)1002010111100??{0 1}10(0
1)1011??0012?0111101100031101110???1(0 1)0(0
1)10000010010100?0000000002100001101111?211010010110101001{0 1}01000200010110000001(0
1)1??11?1011111000(1 2)100112(1 2)010(0
1)1000001101000010010000000001000001111010111?0??010000?1?1?00000000(0 1)01?010100000100101001(0
1)(0 1)1-010?000000000101?100010001?011110?0102110?1?1000000100?0000000100000?0???000???00?-
?0??0?0---?0000000(0 1)00?0-0??????????-0000(0 1)0-0001010110000-00?00100011111?010001010000--
0?0?0000101000200000000--0011-000??0101??????1?0000000
Utahraptor ???????????????????????????{1
2}?00????????00??????????????????????????????????0?????0?2?????01?0??101??011???00011?0{0
1}1111?0??????1??0{1 2}???1?????????101????????????????{0 1 2 3}?????????{0 1}??2???{1
2}?011110000???10?????1111000?{0 1}?00101?00001001???11{0
1}?02??????????????0?0???1??0?????????????1?00??0???{0 1}1??????????????11????????????0?????100???{0 1
2}0????11??????????0???0?1??????????????????10?00???????0???????????????????00????????{0
1}??????10?????00??????????1?????????????????001???????0???????????????????????????{0 1}{0 1}???-
??????000????0??????00????????????????{1
2}1???1??????0?????00??0???0??00??????????????????????????????0??0000???????????????-0000?0-
000?????????????????????????????????????000-0???????000?000??000?00--????-0???????????????1???0????
Achillobator ???{2
3}1?1??????????????????????10??00?{0
1}101???1??1?0?0??000?11210?????????12??1?????????1?101?0??????????0??{0 1 2}00??0{0
1}0??111210?01010000?00111100010111{1 2}00?0???001???0?00?10???????0???1??????????0001??{0
1}??001{1
2}0?????01100??10?????????10?1??0???11?1?0????0000?110210?00???1?1121??100?0??????0?01?1{1
2}??????????????????000??11??0?????????????01??01011??0???11??????100?????01??1???0?1????????????1?000
?000?????????00?????0??1??????????????0??0121?0-
000010000???00101???????0???????10???021?0????0???0010??00??0??????????????????????????????????????
???0??000??????????????????-?00000-01000????????????????????????????0????????000--
000?0000000???????0??{0 1}--???1-?0???0??????????10?00000?

Yixianosaurus
0???
???????????????????????????????????????1??????1?010?210?00??10000000001011?????????????????????
???110?0{1 2
3}?11111??????????????11???01??0??????0?0??????????????????????1???????????????????1???????????
????????????0???1?111?10002000112??????????0???{0
1}??0???0????????0??????01??????110??????????????????????????001??0000???100???????????????????
??0???0???1??
???????1???10-00000??0?00?0???1?0??0?1??????0?{0 1}0?-
?00001??0??00??0????0????
Pneumatoraptor
???
??1?11211??
??01??????????????????????????????????
?????-
?0???1????????????????????????11100????????????????????
???????????????0?????????????00????????????????????????????????????1?????????????????????????
???????????????????????????0??
????????????????????-?01?0?-
00000??0???
?
Saurornitholestes_langstoni ???20110???????????????????{1
2}???10?????1???????1111?1??100??0?????11???0?10000?1?1?10?010(0 1)101(0 1)111011?11000110(0
1)01112?101101?10012?11111?010??11011120010???1?0??000000011111211?{0
1}1011002?00?11??????????1??00{1
2}0000010??001000???1110?2?0???1????011?0?00?1?00020??0?0?00001(2
3)110??0?????0????0?0?0101?000??00000?10210000??0{0 1}11?21111001 0110??10?11?10?????000??1?1?0(1
2)????211101?1?????0???1111?100?10??02101????10010???(0
1)0?010??0011?????0???0??0??000?1???0000??????1?0{0 1}??1??10???0010{0 1}1100-??0??0010?0?00{0
1}0???000??0???01?00?????2111?11??0?00010??0?0?0??10000???????????????????0??0---
??0000000??0??0-1?010?0??-00???1-0001?1?????0??????0????????1?0????????000-00?0????0??{0
1}??01?000???--??01-?????0??0????????00000?0
Atrociraptor ???2001????????????????????{1
2}?00???????????????????????????????????????0?10000????????0100101(0
1)1110?1??
???00101?????????{0 1}(0
1)??0???0????????0?00???????00??0{0
1}???????1?00?0??0????????0?????????????1??00??
??????????????????????????????????000??1?????0??????
???????????????????????0?10??????????00?00????????????????????????---
???
???????????????????????????????????0??
IGM10022_10023 ???{1 2
3}01??????????????????????2??10??0????????01001???1?{0
1}0???1???????0?{1
2}?0???{2
3}?????????????0??{0
1}?????????????????????0??????????11???
?????????????????????????0??
??0??????????????????????????????
???
???????????????????????????????????
Itemirus_holotype_only ???????????-
1111100000001010???????????????????????????????????11??
???
???????????????????????????????????0??0?1?
???0??
????????????????????0???????????????????????????0?00???
??????-?????????1???00????-
0???
??????????????????????????011????????????????????
Linheraptor ?003001010??1?1??00?00?1???200???0001?10010202110?110010?01?10001(0
1)10??0?100000?21??000?011010?11???0110???1-?????????????0??0?2{0
1}1?011?2?0??0?????2????????00?00??????????????????????????????{1
2}?2?10????????0???010101100??0?02???100201100010?0?1-
0???21?????1??12?????0???1???0?????1?0?11??10?{0 1}0?0?000????0????{1
2}???1100?1??2?100100101?01000??10100010000010?0100{1
2}??0????1?1???10??2?0???1??1?0?10?0?2?0???001???000?(0
1)000?010?????????????1????????0???00000???????????????????????0????0??????????????????00?0010??10??10?
0?1?110??1100??111?1100?10?100?100?00010??00?00?00{0 1}???-
00?0?????????????00?????????0?01??????????????????????1???????????????????????????0000???????0??????
????????00????000?--??{0 1}{0 1}-???????01???????0000?0?0
Tsaagan ?0020010100-
1111100000020102100100001?1001120210 1?11001000001000 0100??0010000012111000101101011 10?10(0
1)100?1100???????????????????????(0
1)???????1?10111??

??002011000111001-????????001????????????????????????????11??00??01000?0??0?00???????0-
0101?????0100010110100 1??{0 1 2}010001000001 1?0100(1
2)????211?0???????????????????????????????0??1?00000?0??011????????????????????????0?00?0000???
??1??-100010?010?111?0010???011??????10?101?(0
1)????00?00000{0 1}???00000?-000?-0000000----00??????????????????????-?0????-
000?0???00-
??00??????????0?0??
Dromaeosaurus ????0?10??0-
110010000001010???0100001110???2??1011???01000001000110001001100001211100000000101110?1????????
???0???
?????????????{0
1}11??0?????0?200???00?10010????????????????????????31???????????0?110?00??0?0?0??0?00???{1
2}??????201??????1?{0
1}???110?01????0????1000000110100???????????????????????????????1??0????0??0?0?????000??0??0????
????????????????????0?010000????????????????????0????????????????????????1??-
10001???10?11???10??????0???????0?100???????0??010?{0 1}0??0000??-000?-0000000----
00??00?????????????????????????????????
????0?1?-??00-??0??????????????0??
Hulsanpes
???
???
?????????????????????????N?100?????1???1?????????????????
?????????0??????????2??0??
?????11????2?0????????1???
??1????????000???
???
????--?0{0 1}1-0???????????????0???????
IGM100980
???
??1?01?0001000011121 1??????1??{1 2}{1
2}1??1?1111000100121 13?{1 2}?10????0??0????0?01???001?1?0{1 2}010{0 1}?????????????????{0 1}0?{0
1}???????????????1100??1???0(0 1)???????????????1{0 1}??????????0?????{1
2}102???????12?1??????????????0??????????????????????1110??????????????????????1?1????0???{1
2}10??0?????????00??0????????????????0???1?00??1??????0001000??????????????????????0-
000?????0?0?????????????0???????0?????????????1?????0????0???0???????????????????????????????????
???01000000?0??000--
00?1????0?????????????--????-???????????????????????
Velociraptor_mongoliensis ?0020110100-
11111000100201021001000001100112021110110010001000001110010010000012111100010(0
1)1010111011011000 1000{0 1}1111?11110(0 1)0100120110111010110111112001001?10000000000111(0
1)01121111101000100121120111011110000010001000011011110101010020110110100001011021100012001210(0
1)00000311010011 1?0101110000110100001000000101210101010111112110(0 1)0010110101101101 0001000(0
1)01110100(1 2)010011110111010002000 1121-1010100002100000011010000(0 1)000001(0
1)00110100021??0000100000101000 00???00100{0 1}0010000100001011100-00001000000?00101-
1000100011011010010(0 1)20(0 1)11(0 1)11100(0 1)001000000?0001000000000000000?-000?-0000000----
00000000000-0-?00{1 2 3}00010-000010-010(0 1)01-?1100???010000001210?00000?010000--
010100000110000000000--0001-010110100000-10100100000
Luanchuanraptor
???????0???????????????????????????????????????1111????????????????????????????????????0?0(0 1)?{0
1}101????????0????10011?{1
2}????????0??21??1????????1011112001???????????0????0??0112111100??????????102110????0???????
???????????????????????????0??????1????2?????1????????100?????????????01????????1?{0
1}1?0???0??00??00????????0??11????????????????1?1??????????0??????0?1????11?201???????????????0??
?????????1?10???1?0?????00??????0?1??????????0?0?????????????00????????0100000?110????01200??00
??????????????1??0????0???????1?????20??????????0?????0???0????????????????????????????????????
00??0?0????????????????-?0?010-010100-111000-
001001000????????????????000??0?0?000????????????????????????0??????0??????????0??0?
Adasaurus
???????010?????????0???2?????????000????01?2?2?10?01????0010??000??0?????????0?2???1???????????
????0(0 1)100010?0{0 1}1112??11101???012?1?1???0???1?11111??????????????????????01021?1{1
2}110??2{1 2}?01???1?1110100000000{1
2}000????0110111 1010??1???????1?011????00???1???0????????0?10??21?0?101???0???1????011??00000?0
?0??11?2??1???{0 1 2}???122??1????????1??1??{0 1 2}?????1????01??01??(1
2)?1?????10???????????????0?1?001000?210??0?01?11?0??0?0?1011????????????????001?00????1???0????
?????0??????????????1?02000?010010000000000?0??10????0?1?01101????020????1110????????0???0???????
?????????????00?-000????????000?{0 1 2}0?0?00?????????????-0000??-
??010??????????????????????????????????000??0?0?0000000000{1 2}000?00{0 1}--0001-
?0?110??0??????10?????00
DGBU_78
???
???
?????11?10000??0????????????????????????????????
???1??????????????????????????????2???
???1??
???1?11????????0??

???0000???????
??????????????????????????????????

Zhongornis

???????N??????????????????????????1?????N????????00????????????????????0???????????????1-1---------?-
0?1???????0???{0 1}??{0 1}????1????03???N?1---{0 1}01?N1??210?00??NNN10{0 1}00{0 1}111001??????0{1
2}???00?--2???1???????2-110??1??00111?N??N000?010001103-??{1 2}??1?????-0???1(0
1)?101?3?01?000???0?3001-00?11???01??10????2????0???-?--???0--?????--1??-1?????????-
????N110????21?110?????--???1021-1110??10102N0?001?-1?0?11?002?0{1
2}?000??1?0??????0??????01??????????-?0??1???-????--????????10{1 2}?000?????00010????0-
0????????00???-????0--????1??????0???0?????1N0?0??-???10??-0??01?N?01????????????????????????---
??N??0?00?N00-0---0?--?1???0??0?0???1??-?00?N??0???00?00??????N0000?0{0 1}0?NN--
???00??0????0??0??0-0{0 1}--1{0 1}00-??????????01??0?01?0????

Hesperonychus_holotype_only

??
???2110{0
1}11???????????11021??01?????
????????0?????????1??????????????010????????????12??
???????????????????????????0???????????????????????????????????1?0?????????????????????????????
?????????001200??01??????????????????????0???????0?????20?????????????????????????????????????
??100??00
??001???1?

Balaur

??
??????????????0?1?2?1{2 3}10???0???????????0???1?11110{0 1}001011111100{2 3}000-0{0 1}00-{0
1}??11?0110{0 1}2?2??{0 2}1{0 1 2}2?2?2021????????0110?????1111000101011??????????????????2?02{1
2}??011{1 2 3}?01??-
??10?13001012????0?01??21??1??0?0?0?0?????????1??0??????112??????????????????1???????????????????
??1??11-?011111000200001-2????0?001-
1101000??00??????0?0????00?10100020???????0000???????????????1??100000?010?001?12000??1????1000
00?????????????1?????????????0?0???11101?????????0?00??1??????????????????????????????????????010?
?00????????????-000??0-010?0?-111000-10100110001{0 1}1{0
1}?01000002011?110????0???????0?000000000010?1????10?????????00?01???

Bauxitornis

??
??
????????????????????????11000??????1??0???????????????
?????????0?????????????0??
?????000???2??
??????????????????????????????????????0????????000??
??
??1?00?010-????????????????0???????

Sapeornis 1001000010?0??0?0??0?0?{1 2}?10(1 2)10110(0 1)(0 1)01?000?1(1 2)021001(0
1)0(0 1)00100000010??0?0?0(0 1)120{0 1}0-(0 1)102100000201--(0 1)--0-0101?01??120000{0 1}?021?00(0 1)0?---31-
1000---21110(1 2)11(0 1)001001?10(0 1)102000-01(0 1)(0 1)-222211120-2(1 2)01--20(2 3)101111(1 2)1?2-
110000??0???1?11(0 1)100000(1 2)00(0 1)10031-0(0 1)00(0 1)11??1-021010?101030(1 2)0---(0 1)1100?(2 3)00101(1
2)1111(0 1)-0000(0 1)(0 1)011111(0 1)00(0 1)01?1010(0 1)(0 1)00--00(0 1)0312112-?000001010010011(0 1)(1
2)010002(0 1)0110010-0012(1 2)10021-211(0 1)1?10(0 1)00(1 2)(0 1)111-1-1?011(0 1)11-20010(0 1)00-011000100-
00001010(0 1)??11?1?021-00101?0??0??1?100?????110(1 2)?0(0 1)0100?00(0 1)01201--0-000??(0 1)??0?0??000--
000(0 1)-0001??--?00??0020??000(0 1)0(0 1)00(0 1)0-1000?-100(0 1)2(0 1)0(0 1)0(0 1)??0?????1???0???0010010---
00000201(0 1)000010----?--0(0 1)0-0000100010(0 1)01-000010-00101010001010?(0 1)(0 1)0001010000-0-
0010000001000(0 1 2)000(0 1)00000001(0 1)-0-0??(0 1)10010(0 1)011-00?00000

Zhongjianornis ?-0----0?-??-????-10?{0 1}?????0??0?01?-
1???0??00???01??0?????????????????{0 1}002?200??0??0?1-1---------?-?????????????????{0 1 2 3}????{0
1}?????????1??0??011?02-111010???10100{0 1 2 3}(0 1)00?(0 1)100?{1 2}12210??0??12{1 2}--201??2???0?02-{1
2}10?01??0????011111?0?02000?113--?{1 2}????1???1-?210????1?1?3110???01??1?30?1-1{0
1}??????0010211?102-00?0?1?-{0 1}--?000--0--00--1?2-?-0-212??0-?-???100--002001?0???00--{1 2}111021-
21111?0000110100?1-1?0?10?0?2{0 1}000100??1?0?01?0000??????1??1?????11??01???0??????1--
0???????001??0?110??010?{0 1}{0 1}?--000?0??1??0????0-????0--???0???????0????????1111?0?--1---
0??010?0200001????0????????????????0?----10?0??11???0??-?????00100000?0?00??10???10?00-
1?00001??0000{0 1}100000?1?000010-001000??11?0000??01??001{1 2}11-?????0??0?01-?0????0?0?0

jianchangensis

?00????0?????????????????????0??0?0??????????????????1?000????1??????????0???????????????1-1---------?-
1????1???0???????{2 3 4}????2??---3?-???????????????????????????????????????021??1??11101?--2?1?111{2
3}?2???2??????1??????1?11110?0?010??0101-??{0 1}????1???{0 1}-11100-??????0?????01101??0?1-1{0 1}????--
???0????102????0???-0--?000--{0 1}11??--?1?-????0??0??-1???1?10??????0??00????--
211????????????????????????1-1?0?1??0?{2 3}{0 1}0????0-???????????0???????????????????0??0???-????--
??0?0????????????????????{0 1}??0?00?0????????????-?-??0--??0?1?????1????0????????00??-
0?1?????????0??1?01?????????????0???????????????00030?1?0??00??????????????????????????????????????
????????????????????0?00-00????0?0?1?0?????110?????11-?-?????????????-00???010

Jeholornis 110{1 2
3}?0001????0??????????1???0?00?01?1???00?12021?0???1???0100????????0?0012000-00021001-0201--(0 1)--?-
-0???0???1200102?01??0021101111110100100111021101000111?1010000010010(0 1)01222?1?101020{0 1}--
202211111(1 2)1?2-110001??00101000110000020000110(2 3)--01??111??1-0(1
2)1022110113?1020001100?30010121110(0 1)100110010101-00?0???10100(0 1)00--11??31211(1 2)-1?1?10{0
1}0?1-?0?10110??00200110??1-0-02(1 2)110(1 2)1-21111?100011111001-1?0?101102000000010(0 1)?0????0-
01???1111??11????021?00??1?0?-???0?1?0?????11020000000?1?10101(1 2)--0-00?????0000?10?10?00--

??0???0????0??0020??001100???0-00100-10?0201001?00?????????0???00?00000---000010000000-0-
?0????0102000?(0 1)0000110(0 1)-010000-001011100010101 0(0 1)0101(0 1){0 1}0000-0-00?010000{0
1}0000?001000001011-00????1?0100-11??010??(0 1)0
Jixiangornis ?10????0100????????????????{0
1}?00????11???001?0??00??00????????0100???1?00120000?{0 1}0?0001-1---------?-
00?0?????000??2??2???02???11111?11?2100111021111 00001010(0 1)00{0 1}0000(0 1)1000?{1 2}22110{1 2}1-
2201--2?{2 3}0111{0 1 2}120?2-?1?11101???011111100100(2 3)0001003-??1?01????1-0{1
2}??12110113110?00011000300101(1 2)?11???0010000??12-00?0?1?-0--0000--0???1--112-?0?0{0 1}1{1 2}0??-
?0??0110??00{1 2}?0110??100--{1 2}??1121-21111?1000(0 2)11(0 1)0(0 1)?{1 2}-1?0?10100210(0
2)000010110?01?0000?????11??1?0??0?0??00???0???1?0--???????1102?01?0?0?0010?0{0 1}2??0-
??0??1??000??0-?0??0--??0????????1???020??0111000?-???11??0(0 1)0002?0001????????????????????0?00----
?00?{0 1}10?00?0-000?120?00021000(0 1)00001101-?10000-0010110????11?000000(0 1){0 1}0000-
10001000?00100?{0 1}??0100000?011-00???0??0101-111?010?100
Yangavis
???????0?????????????????????0??0?????1???????0?????????????0????????????{0 1}011?201?10?10?1-
1??????????-?????????{0 2}????{0 1}??2{0 1}?0???0??????111?N1?{0 1}?1?121?110?0???1010000000010000?{1
2}???02???20???{0 2}?2{1 2}?{2 3}12?21????1??????????1?1111??0?020011113-??1???1???1?02?01??1?0?3?{0
1}1000??100?30?10?2????-?00???0??1??-0??0?1??0?????????2?1?0?-1?2???0?2??0??-
?????100????2100?1???0?-?{1 2}???02?1?0111?100?0{1
2}001?0??1?0?100112?000?01????????11?00????00?0???????????0?????????????1??0????????1?1??0???0??000
??{0 1}2??0-000?????0????0-????0--????????????0???0?0??1?11?????????0??000?02{1
2}00?1????????1?????????????{1 2}?---????0{0 1 2}?10???0?0-???{1 2 3 4 5}00000-?0????????0??1???0?1??{0
1}??01?0????{0 1}???010?100{0 1}?000-????0?????????{0 1}00???100?100?11-????????????0??1??0?0?010
Eoconfuciusornis 110-1-00????????????????????0?00?01??????00??0?-
??????????0?????????????00110100020??001-1---------?-0????0????????{0 1}??2100?{1 2}??---3?-
10N?11?0?1?N2??111?00??10N100(0 1)010(0 1)1010???2????1?1??????????{1 2 3}?{0 1 2}??1?2-
?????1???010??N1?1??1?0{1 2}0001113---10??10??0-0{1 2}101-?10??3001000??10??30?1012?1100-
00??110???2100?0???-0--0??0???0100--1??-1-0-21000?-1?-?1110--002100??1??10--?{1
2}?0010?2?101?10101200?00??1?0?101012?00?000-01?-??10?00??????1???1???10???00??1???-???1--
0???????101?01??0???010??????????0??1??0???0?-0-?10--?0?????????0????????1?10?0?--?-??1??-1?-
01100?1?????????1???0???????0{1 2}?---?0???{0 1}010??0000-??????000??0?00?001?101-?00?N0-
00?00??00????{0 1}?0001??010????????0??????????0??0?000????1{0 1}-?-???????1?0-11-????00??
Proornis
???
???11??0????N??0000{0
1}0011???
01000???????????????1????00????????????????????????????????????1?????????????????????????????????
????????????????10??1000{0 1}2000?{0
1}???????????1??????0?????????????0??{1
2}??0???0???00??0???{2
3}???
N001??0?0???
Confuciusornis_sanctus 110-0-00??0?-??0?-1?01?????0100101??1???000?02-0-
?00000?1000?01???1?1000110(1 2)0(0 1)020?0001-1-----------00?010?0020110??12{0 1}101100---3?-1(0 1)10(1
2)1101101211(0 1)1(0 1)0001?101(0 1)000000(0 1)1(0 1)(0 1)10222110210121 1--2022121312112-
210?110?00??111111010102000 1113---100111??0-0110(0 1)-1100130110000110003001012111 0--00100000112-
0000010-0--0000--11100--1?2-?-0-210000-1?-111(0 1)0--002100-111?00--201?021111111010(0 1)01(1 2)00(0 1)002-
1?011(0 1)10121010000-11?-0010?00100(0 1)001100111?111?001?0110?-0??1--00??0??1101?00000011?(0
1)010{0 1}?100-?01??0?0000??1-?-?10--?000110??0?0???0?0??1010001--?--?(0 1)0?000-(0 1)11(0 1)001?0010{0
2}??11??00?-011002011110000{0 1}00100000000013000(0 1)0-00000001-010(0 1)-000010-001011001012101010(0
1)00010010-1000000001010010?100000101010-0-?1011??100-11-00?00010
dui 1??-???0?????????????????????0??0??1??????0???0{1 2}-
???0??????????????????????01102?0010??0?1-1---------?-0???????????????????????{1 2}??---3?-
??1?11?????????011?0????010000100(0 1)1000??????0???????????????{1 2
3}????????????????????????11??{0 1}?020011113---10??1???1-0????-1????3?1100?0?????30?1?12?11??-
00???010??2????0???-0--???0????1?0--1????-0-{1 2}??00?-1?-?1?10?-0?2100??0??10--
??????????11??1?0?2101010??1?0?111012?01?000-1??-??1??00??01???1???????1??0?????????????1--
??????????1?000??????001??????????????????????-?-?10--????????????????0????10100 1??-?--???????-
01??0?1?????????????0???????????????0???{0 1}??1????00-??{0 1}2????????????????????1-
??011???0??0?????????{0 1}?0100??0{0 1}????????000??????????????????1??11-?-???0?????????-????????
chuonzhous
???
???
?????????????????????1?N??1??{0 1}?0{1
2}?00???30????2?0??
??10???2?0????????????????
???
???
??0????????-
????????????????????????
Elopteryx
???
???
?????12-
2?0???1????
?????????????????????????-

???
?????????????????0???1011????????1??????????????????
???
???
?

Changchengornis 1?0????0????????????????????100?????????0???0?-
??????????0????????????0??????0????0001-???????????-?????????2??????021{0 1}0?{1 2}0?---3?-
???011?0?1?121?011?0????0??0000001001?2?2??0???1??????????{1 2 3}????1?2-
??0??1????????1112??0?0{2 3}0001113-?-1????1?????210?-?100?(2 3)?1{0 1}0000??0??30?1001?110?-
00????????{1 2}-???0????0??0??0?????0{0 1 2}-????-??????????-??-?1110??00?101?????0?--
?0??02?02111??100?02010?0??1?0?111113?0??00?-01?-??1??00????0??10?????????001??????-????-
?????????1?{1 2}??00?00????0?????????????????000?????-??0--?0?01??????????????????11?0???????1???0?-
?211??1??????????????????????0????????{0 2}???11??0000-?????{0 1}000-
00???????0??0???0?0??????0????????{0 1}?0000??0?0????????????0???????????000000??11-?-???1???100-
11-???00???

Chongmingia
1??
?????????????????????{2 3}1?0??????????????1???0?1?1{1 2}-?0{0 1}000???11100?0{0
1}??10?????????????????????????{1 2 3}?3??1?2-?????1????????111110?0?0001?????????????????2?0(0
1)???00?3000??????0??30?10?{1 2}?11???00??10???{0 1}?????0???????0???????????????-
?????????????????????????????????????211?0??1?1111?10000101 1????1?0?11?1?3?0???0??0?????????0???
?1?11??0?0??11??01????0?-
??????????????0?1?01??10???1????????????0??1??0????0??????????????????????????????1?0?0??????????
?0???2???00?0???????000-?1??1????1??0-
??000???0???????????{0 1}?0100??0?0?????????????0??????0???0000??1011-??????????00-11??0?0????

Yandangornis
???????0??????????????????????0???0?????????????0?1-
1??????????-0????????0?????????????2????{0 1}{2 3}?????1?10???1?????2000{0 1}????????{0 1}??{1 2}{1
2}???????????????????????????{1 2 3}1?????2-01?101??????0?1112??{0 1}?020001???-?????????????1???1{0
1}?????2?1??????????30?1-0{0 1}???????????????{0 1}??????1?0?????????????????-???-??????????-
????0?3????????1???????-?{1 2}?????????1???????0{0 1}????{0
1}??1?0?11?003?0001??????????????0??????????????????0?00???1?-?????????????????1?0?0??0-
???0???????????0??1??-????0-?????--????????????????????????11?1???????????????{1 2
3}???1????????????????????????????????0???1????1-0-???1?1????????????????0-
?1?10??001001????????????????????????????????1?0?{0 1}?{1 2}?0?0100????11-???????????????????1?????

Jinguofortis 1??{1 2}??????????????????????????{0
1}1????????1?????????????????????1?????????????{0 1}0120000?20??100?0202--011?-
??1?010?0?2???02??2????2??---3?-?0??{1 2 3}??0?1?12-?210?0???111002000-0100-222??1?2???21?--2?3?1{1
2}12?21??????0?????????????11000?020?0{0 1}003???????1???1-03(0 1)0(0 1 2)-?1?0?3?20---0?1?0?30?1?0{1
2}?1100-00??101?1?0-1??0????000?1?1--??1?11{1 2}1????00??0??????????1?20?00?2?01?????0?0?{1
2}11011112 1111?100?010110-2-1?0?1?1?-2?001100-
00????????00???????1??1????010?????0????????1???0??????101?01000???010???????-?1???????????????-
???????????????????0????????1111?0???(0 1)?0?0???00?02{0 1}0001??????????????????????0?----??{0
2}03??000?100???{0 1}???010-?????????11?0-100000-10??1??10??{0 1}?{0 1}?0000010{0 1}00??-
???00?000?????????????00?00?11-?-???1????00-10-???0??1?

Wyleyia
??
???21?0???
??
??1????????????????
???0??0?00?????????????
??
???0?11000????????1??0???
????????????????????????????????????

Protopteryx 110{1 2
3}???NN0??????????????????01?0??1??????N0?20???0?1?????0100??????????00010????????0?0-120---0?-
??00?????????0??????021???{1 2}??---3?-1N1?{2 3}1?211?N2-?210?01??1N1002000-0100-?22??112????0?--
2?22131{0 1 2}?21?2?2????1??0?????N?N{1 2 3}110?020001?03-?01????1??{0 1}-0?0??-1101?3?11---
??100?30?1?1{1 2}1110?-00??0?01??1-0??0?????10???0--?11??1-1?????0?00????-???11100??0N?101?01????-
02???111-21111?101012?010-{1 2}-1?0?1011-3?000000-
00?0??????00???1111??1?????0?0????1?0????????1???????1011?10??0????10???????-?0????????????0-?-
??0???????????????????0??????1100?????0?10?-10??2{1 2}N?01?????????10??1????0???00----
0?0?10110N00000??1{2 3}0N01011100?001-?1000?100N??00??1??????0?0-N000??1{0 1}0N??-
0??N?000?????????????100001?1{0 1}-?-????????00110-???0?0??

Paraprotopteryx
??
??????????????????????????????????????1?{1 2 3}1?2?1?N2??210?0?????1102000-111?-
????????????????????????????????????1???010??N1?1??0?0200?{0 1}?????????????????????????1???3?20---
???????0???0{0 1}?110??00?????????-
????????????????????????????1??????????????11?????????????????????????121?2?101?100?01110?-
??1?0?11?1-3?0???0???0????????0???????????1????02?0????????-
??????????????1?1??0??00???10??????????????????????0??0??????????????????1???0???????1??{0
1}????????????1???2???0???????010?01??0?0???1?0??????0?
????????????????N00???1?0????????????????????????0??????????????????????10??0?????????

Orienantius
1???????????????????????????1?0??{0 1}??2?10????????00020{1

2}--0??0??0????????0???????2????2??---3?-??1?{1 2 3}1?2?1?N2??20010????NN002000-1100-??2??0?????20?-
-2?1?1{1 2 3}?{2 3}??1?{1 2}?010??????0????N?N{1 2 3}010?0200001030??????????0-0{1 2 3}10(1 2)-?10??3?10--
-??100?30?1-1(1 2)?110?-00??{1 2}11??{0 1}0-?0?0?????(0 1)0???0--?????1?1??-
1?????????0???11110????2???????0??0?????111?2?111?1010011100-2-1?0?10?(0 1)-3?00?00?-
?0????????0????????????1????0??1????1???????1??????????101?100??????10???{1 2}???-???????????????2?-
?????????????????0???0?????01000??0????1?0?10??2?N001???????????????????????---
?????2111??0000??????1010?00??0?10??1?10?00?N??{0 1}???1??0???????N000??1{0 1}0???-
????1?????????????????10???1?11-?-????????01010-?????0??

Eopengornis

1??????N???????????????????0?0??????????????????0???????0??????????????{0 1}??1?00????????00021{1
2}--00??????1??0???2?????????????{1 2}??---3?-1?10{1 2 3}1?201?N2?1200?0???1NN002000-011?-
??????????????????????12?21????????1??00?0??N??N??0?020000??????{0 1}??1????{0 1}-?????-1101?3?00--
-??????30?10?2?1100-00??{1 2}?0???0-
?0?0?????10???0???11??111???1?0???????0???11100??0?2????0??0???0????121-1?1(0 1)1?10000{0 1}111?-
??1?0?1001-2?01?100-?1?????N??0??????0????????0??0?????????????1??????????00{0
1}?1??00????10???????????????????00???1?-
??????????????0?0??00?????01?00??0????1??????02NN?00????????????????????????---
??N0???1????010???12?N00010????00??10110100?N??????1??00??????N001??100????????????0??0??????00
10??--1?21-?-???1????011?0-???????1?

Pengornis ?1001-001-

?????????????????01001010?1?000101021?000?N??10001?????????1?{0 1}00(0 1)001-?10?000000211--
00?1??11101110?(0 2)??????03??0020?---3?-111?{1 2 3}1?201002-121000?1?1N1002000-010(0 1)-12(1
2)??1?2???02{1 2}--2?2{1 2}1211?(1 2){0 1}?2-?10?010?00?0??1111?00?0200(0 1)11031-?200110??0-?2100-
1101030(0 1)0---0?001?3001012?1100-00?0{0 1}?0?100-00?0?0??{0 1}(0 1)0?(0 1)00--(0 1)11-311112-1-0-00001101-
0111100-002100101?1-?002111(0 1)(1 2)1-211(0 1)1?(0 1)0(0 1)000(0 1)100-2-1?0?101(0 1)-21010100-0(0
1)?0??11?-00?00?1(0 1)1??1????(0 1)(1 2)00?0????0?-???0?1(0 1)??????10?1?11000?00?101{0 1}?1??0000(0
1)??1?0000?0010-?00?0?00-1???10?0??00{0 1}0???011000-00-0-110N1000200000????????????0???0??0?00----
10(0 1)?{0 1 2}011??00(0 1)0?00?3?0000001000?000101(0 1)0100101000001?101?0{0 1}1210001111{0 1}0NNN0-
?10??0000?{0 1}10?10001(0 1)000010{1 2}{0 1}-0-?????00?00?10-?0?0?0(0 1)0

Elsornis

???
??????????????01??010??????????????????210211002-
020000?1?11100??0??00??????????????
??????????1011??2????????????????010?????00??2??????-
?0???0??????????????????????????????????????1?1????????????????????????????111-
21111?1000????????????????????010?0???0?????0???????1010011?001???0????????????????0100?0??01101
0001100?1??1?????????11??????0???11??1???1?????????????
?????????????????????0??010??????0??1?000?0001000001-11010101001010110110000112?100?10?{0
1}??00010????0????

Otogornis

???
??002-
121000?????1?????????0???
???????????010??20?????????????????11????0?????????-
?0???1???????????????????????????????????????1?????????????????????????21-
?1101????0???01??????????????????10?????1???????????????????0???????????????????????????????????01?1
0000??0?????????????????????????????3?????????????
???00?0????0010000?1010102000111111?0{0 1}1{0
1}???0????

Yungavolucris

???
???
????????????????????????111101?????0??0????????????????
?????????0??????????????2??
?????010???2???
??????????????????????????????????????0????????02???
???
??0000?020-0?????????????????0???????

Noguerornis

???
???2????????????0???11100????????????????????????10?--
2??10??31????????????1?????????0??????????11???
00??2??????????????0?????????--
???????1???1??1?????101?1010????????????????????????1??0?????
????????????????1????????0??????????????????????????????10?{0 1}????????????1????????-
??0????????????2???????????????????????????????
?????????1??0???????????010???????????????1?101????0???1??00????{0 1}??001??1???00-
???00?10?????????

Iberomesornis

???
???????0????0{0 2}01?????3100?2?0---3--?01?21?2?1?02???00?0???????????????????{1 2}2{1 2}2?1?{1
2}??020{1 2}--20{1 2}{1 2}?{1 2}??02????{1 2}????????0?????11110?0?030001??????????????????0(1 2)00?-
11???311????110?0?30?1-12?????-???????????-???0?0??0?????0--
????????12??????????????????111????????????????????2211121-2111??????????????-1?0?11?1?320100??-
?1?????10????????11??????????10????1????????????0???????0?{0 1}??1?0??????????10??0-001??1??0????0??-
?????????????????????011???01110???0???110-?0??2??002(0

1)01?00001{0 1}???{1 2 3 4 5}?{0 1}010?000?0101????1?11???????10001?????????????????000-
????0??00??????1????100011?10-?-???0???101110-???0????
LP_4450_IEI ?1?----
NN0??????????????????0?????10?1?0NN?0202??0?0??011010??????????????00??00?20?1000?0N01--
0120???1?????0?2??????????????????????1??????2?1?N2-1?{0
1}0?0????N1?0?000?0000???1030-
?10??11??{0 1}-???????10?0??10??????????????????1????00????00??0-00???????1????????11?111?????-0-
01201?01????1{0 1}0--
??2101101??0?002???1?????11??10100N0?11????????????????10?00?0????????00??01??1???1?????????????
???????0??0???????001?10???????10???????????????????????1???-0??-??????--??????????-???????-0?-
01????1??02??00??????????????0??????0???????1?0???????????????????0?0?00??0?0??10?00????N???0??1?
?00??????N00???1?0??1??00??0??????1??
Concornis
1???
??-?????????????00??0?{0 1}?0???0?????????1?21?2?1?02??210?0?1???1?0??00??????????????????20?--
21211{1 2 3}????10??2100????0???1?11110?00030??{0 1}??????????????????030?1?1101?3010??????0???0?1-
1{1 2}?11????0???????{0 1}?-???0????????0??0--
????????????????????????????1??????????????????????211121-2?1???100?011?????-
1000101??3?0??00???0?????????0????1??0???????????{1
2}?0????0?????????????????0?1??0??000??10??????0?0??????00???????????????????????????1???0???????1?0
0???0???1????0??{1
2}?????????????????????????????????????111???0?00??1?01010001??00100??00?110?0102????0???1121??
10????1?0??????????????00?1??0?00???10?000?10-??????1??100110??0??????
Cruralispennia
1?0????0????????????????????0??0????0??????????0?0?11???1
10???????????????????????????---3?-??1?{2 3}1?????02??200?0???????0??????1????????1?????21?--
2?3?1{1 2}1{2 3}?20????1???11???????1111??0?0{2 3}010{0 1}103???2????????1??(2 3)1??-??01?2?{0
1}0????????0?{2 3}0?1?12?1100-01????????{0 1}-???????????0????--?11???{1
2}?12???0????0???????1110??0?2100?????????1?11??11-??10???????0{0 1}1?????-1?0?1??1?3?0{0 1}?100-
?0?????????0??????10??1??????00????????-????00?1???????01??0??00????0???????0-?0???????000?????-
?????????1??????0???0?????01?0??????????????10??2?0?0???????????????????????????---??0?????1???110{1
2}?????1????01??0?0???1?0?1?0?0????????????????????{0 1}??????00?-??????????????????????0?0??????2{0 1}-
?-??????????????-???????1?
Bohaiornis ?10----
0?????????????????????0??0?01??1??00??2021?0?????????????????????????11000-?20??00000201--
0120??1?????????????????020?0?20?---3?-111?{2 3}1?201?02-120010?11111003000-01?0-022??1?2???201--202{1
2}12?1121????1?001{1 2}?00?01?11110?0?0200001030-?1????1??0-?200(0 1)-1101?3010---11100?30?1-12?11??-
00-?210?-?0-?0?0?????(0 1)000?0--?(0 1)(0 1)?31211{1 2}?1-0-01200?0?-??1110--00210110??0-10022??1(1 2)1-
211(0 1)1?10(0 1)00211(0 1)(0 1)-2-1?0?1110-210{0 1}?000-00?0??NN?-0????111???1????0002?0??1???-
???0??0????????0?1?1??000???101?{0 1}2??0-??1?11?100???(0 1)1?-??0?0?0??1???????1??00?0?-0110?0?-(0
1)0-011???1?00210001???????11?????????????000----?02??111??00000??12?1000001??0001-
?00001?010102???1??10??1?2-000(0 1)101?00??-1?10??0001???0?0???(0 1)10?00(0 1){0 1}{1 2}1-0-
???0??0?001?0-00?0?100
Parabohaiornis ??0----
N????????????????????0?????????????N?0202??0???0??????????????????????11000-??0????010201--012?-
??????0????{0 2}1?0??021?0?200---3?-1110{1 2 3}1?2?1?N2?12{0 1}000???1NN003000-0100-021??102???201--
2?2{1 2}0{1 2 3}121210???1??011?00101011NN010?0{2 3}00011031-?{1 2}??1????0-?(1 2)10(1 2)-1101?3010---
0?100?30?1-120????-00??210?-?0-?0?0?1??0(0 1)0???0--211?3?2112?1-0-01?0??0????1110--0N210010??0-
?00221?111-2?111?1010010110-2-1?0?1110-2{1 2}000000-00?0????0-0?????1?0??1????01?1?0?????1?-
???0?1??????0?001?10?00???0101{0 1}?2??0-10??1??1000?0?00-??0???????????????1??0020?-?(0 1)100(0 1)?-
?0-011??N10??2?N001??????????????????????????---??N?021110N0000???1401000?01??00000?100?110?N0-
101011000????20N001111{0 1}0NNN-??0?10?0?01?0011???010?001010??-???1??0?01110-000??000
Longusunguis ?10----
??????????????????????01?0??1??1??N???00?1???????????????????????????11000-?{0 1}0????00020{1 2}--0020-
??????????2?1?0??0{2 3}???01??---3?-??1?{1 2 3}1?201?N2?120000???1NN103000-11?0-?????10????20{0
1}??{0 2}?2??{1 2 3}12?2??2-210?011?0??011N1NN010?0{1 2}000{0 1}1031-?1????????0-?2(0 1)??-?10103?10---
????0?30?1??2?11?0-00??210??{0 1}0-?0?0??????(0 1)0??00???1103?21?2-1-0-01?0??0???11110--0N2?0??????0-
?00{1 2}11?111-211(0 1)1?10(0 1)001?111-??1?0?1?1?-2?010101-00?0?0???-0???????1???1????01?1?????????-
???0???00?0??1011?11000????0010?????-?1??1????00?0?-0-??????0??????????1??0????-0110?0?-?0-
??????10?02{1 2}N001??????????????????????0?00----???021?1???000??????{0
1}00?0?1?00?1??110001?00N???0??0??00????21N001??1{0 1}0?NN-????????0?10?00?20?00??0??????1?0-
???1?????????-01?0?01?
Shenqiornis ?1?----
NN0????1??????????????0??0??1?????N???2??1?0?0???????1010?00???????0?11000-??????0000201--
0210???0??01?1?2??1????1{1 2 3 4 5 6 7}?????0????????1?1?{2 3}1?2?1?N2??210?0?1??NN?02000-(0 1)100-
?????1?????21?--21220{1 2 3}12?(1 2)10?????????????010??N1NN??0?0{2 3}0??{0 1}1030-?{1 2}0?1??0?0-
031???110??3?{1 2}0---????0?30??????11?0?00?02?0???0-00000??1?(0 1)0????--?11-{1 2 3}111?2??1-0-
0100??01?1??110--0?21?1?011???00{1 2}11?121?2110??1010021111-?-??0?1?1?-{2
3}?00?000?00?0???N??0?000????????????0?01?????????00?1?1??????0??001?10??????010???????0-
000??0?????????1????0?????-???????????0????????1??0?-00-01????10002NN001????????????????????01?0---
?0N0??101??00?0???13?N000?01??0?0?0?100?11??N??0???1?????1???{0 1}?N001111?0NNN-
??0N????????????????????0?101?{0 1}0-0?0????????001?0????0000?
Sulcavis
?10??0?NN0?????????????????????0010????????????????0?111????????0100???????{0 1}??10?0?????????00020{0
1}--0110?10????1???{0 2}?1??????{2 3}????2??---3--1N1????2?1?N2??210?0???1NN003000-0100-?2{1
2}??102??1????????????{1 2 3}??????????1?0?1???0101?N11N010?020001???1??{1 2}1??????0-0??01-110??3010--

-??1???30?1-12?1???-00??{1 2}?0??{0 1}0-???0????0(0 1)0?0?0??010??1{1
2}1???1???0?????01???1110???N?????0??0??002??01???{1 2}?111?1010011101-??1?0?11?1-2{1 2}00?00?-
0??????1??0??11???1??1????010??0?0??0?-?0?0?00???????0?1??0??????0101?2???????0??0??00??0?10-
??000????????????????0?????0?000??(0 1)??0?1??-1???2NN?01????????????????????????0?----
0N0?11?1??0000???2{2 3}??000??1??00?????01?1???N??????1??????{0
1}??1N000??1?0NN????0N01???????0?00??110?00??10-?-???1???00?10-?0?????0
Zhouornis
?0010??0????0?1???0?10?????0??0?0???1???0??20?1000?00?????010?00??????011(1 2)000-0{0
1}0??00000201--0120-101100?00?2??00??121{0 1}??{1 2}??---3?-111?{2 3}1?2?1?02-120010?1?111003000-0(0
1)00-{0 1}{1 2}???(0 1)?2????1?????{1 2}??{1 2 3}12?210??21?011??0???111111110?02000(0 1)1030?00?01??0?(0
1)-?3101-110113110---??10??3001-11?11?0-00??210?-{0 1}0-?0001???0(0
1)00000???11031211???00000?00?0??0111100{1 2}00210?(0 1)0?10-000211?111-211(0 1)1?1010011111-
2?1?0?1(0 1)1(0 1)-(2 3)001000?-00?0???00-0?0????10??1????0100?0??1?0?-
0??0??00100?0?001?11000??0010?????????00??0?000???0(1 2)?-??0?0?0?011???0?10?00?0???0100(0
2)0?0??0?1??01?102{0 1}?001????0???10??????0??0?0????-?00??111??00000??(0 1)30100000(0
1)??0000?110101?000???00?10?000?(1 2)?210001001{0 1}0000???00??0001?1?0?0?1?0100001{0 1}10-0-0??(0
1)??0?101?0-00?00(0 1)00
Fortunguavis
?????????????????????????????0???01?????-
?1?????0?0??1?????????0???????0???????{0 1}2{0 1}0?2??---3?-??1031?2?1?02-1200?????11100210?-(0 1)100-
????????????02--202{1 2}1{1 2}1102??2-2??0?10?????11111010?0?0001???????????????1?021?1-?10103010---
??100?30?1-12?11?1-00-0(1 2)(0 1)0??{0 1}?-00?0??????????0--0???31?1?2-
????????????????111?????2????????-0??{1 2}11??21-211?1?1000011100-?-1?0?10?1-2001??0?-0?????1??-
0??????1???1???102?2?0????0?-???0??000?0????010100?0????10??????0-000??0??0????0??-
?????????1??????????0?0???110?00?0????1???10??2?00?????????????????????????---
???0???110??0001?????10?00000?0000011?101??10??????1??00????220000001{0 1}0000-
??0????01????01??10?100?00?11-0-???????1?????-?0?00?00
Dunhuangia
??
?????????????????????????????????????1?{1 2 3}1?2?1?02-?210?0???111003000-1{0 1}?0-
???1?01?{1 2 3}?10---
?????????????????????00??1??????-
????????????????????????1???????????????????1????????????????????????121-211?1?10000{1 2}1011-
??????????-
???1??0??01????????0??????11??1?????1?0??????????????????????011??0??????010??????????????????
?????????????????????????????????1??????????????????{1 2
3}???0?0??4?10?0001?00101011???110?0??{1
2}???1??00????{0 1}?000110100???1??????0????
Yuanjiawaornis
??
???????????????????????2100?20?---3?-??1?{1 2 3}1?211?N2-120000?1??NN?0?00??0?1??121?11??10{0 1}200--
202{1 2}1212?2?????????10???????111110?0{2 3}0??0??????????????????2100-
110113?10???011?00???????????0-00??1???1{0 1}?-?0?0????{0 1}???00?--
????????12??????????????????11?????????????????????111121-?111??1???????1??2-1?0?110??2100??0?-
00?????????0?????10??1????0?02?0????1?-??????????????0?1?1?0?????0???0?21?0-?00??0??00???0??-
???????0???????11?????0????1??10??0???1???10??2??1?1??0000??
?12?10000?0000?0?010000??00N???0??1??0011??2?N001??1??NNN-100N???0???{0 1}???0??01?0{0 1}??1110-
1-????????010?0-???0??0?
Eoenantiornis
?101?0?0?????????????????0??0?0???????0?????1?0??00?????????????????????????????00010202--
0??0??1????????????????????0?0?---3?-?1??{1 2 3}102?1?02??200?0???111003000-0000-
0?2??????????02?????2{1 2}131{2 3}?2??2-210????????????1?11??0?020001?03???1??1????{0 1}-????1-
?101?3?10---??????300?-00?1100-00?0{0 1}0???00-???0????{0 1}00?????????11???{1 2}1??-
?0?000?0??0????1110?{0 1}00???1????0?000{1 2}???111-?1101?1010011010-??1?0?11??-3?00?00?-?0?0?????-
00?????11????????1?1????0??????????????????10?1?100?0????101??????-?0??????????????-
??0???00?1????????????????0?1?????0????1??0?0??21??00??????????????????????????????0????1???00{0
1}0??01??1000001??0?00011000??0?0??????1????????200001??110???????0???0?????????????100????10-?-
????????0?110-???0?00?
Huoshanornis
???????0???01?????????????????????
????????????????????????2????2??---3?-????{1 2 3}1?2?1??2??200?0????????0{2 3}0{1 2}0-(0 1)1?0-
?????????????0?????{2 3}{1 2}?{1 2 3}????1????10????????????1111??0?0{2 3}00011?3????????1???{0 1}?????2-
?10??3?00---???0??{1 2 3}0?1-1{0 1}?????-?0?????????-
???0????????????0??????????????????????????????1110??????0???????????{1 2}???1??????1(0 1)??10000{0
1}1???-??1?0?11?1-3?01?00?-
??????????0??????1?????????????0????0????????????????????0?1????????????10????????????????????????-
??????????????????????0??????11?0????????1???10??2???11???00???
????10?0?0???0?0?????0?????????????????????????0????1?0??????????????????????????10???1?10-?-
?????????????0-????????
Grabauornis ?1?----
??????????????????????0???????????????????????????????0?0??????????????01?0??????????000?02???12??????
???????????????3???02??---3?-??1??1?201?02-1?10?0???11100?0?0??1???{0
1}22?1?0??????????????????????2-210??1????????1111?1{0 1 2}?0{2 3}00?01030-0{1 2}???????0??1??0-
?001?3?10???1?????3001-1{1 2}?110?-00????0??00-?0?0????0????00???11???2???-?-?-01{0 1}0??0????1110--
??????????????00{1 2}???111-21111?101001????????1?0?1??1?{2 3}?01?10?-?0?0??????00???????????????10{1
2}?0????1???-??????????????1??{1 2}???????????101{0 1}???????????????????????-

?????????????1??1???????-?111?1?-??-0?1???1???2?0?00??????????????????????---
???????11???000???1??10?0001??0?1??11?0?????????????????????0??001???{0
1}0???????0?0?????????????1100??0{0 1}1{0 1}-?-????????????0????0????

Jibeinia

??2?0?????????0?0202--
0?2????????????{0 2}???????3????-??---3?-
????21???1?N2??210?0????NN?01?????1?????????????????????????12?21???????????0?????NNN{0 1}??{0
1}?0{2 3}00?{0 1}1?????0???????{0 1}-????0-?10??3010---??????30?1-1{0 1}?1100-0?????????0-
?????????0?????????????1?1?2???????????????111{0 1}0????????????????0?????{1 2}1?{1
2}?11??1010010???-??1?0?10?00{2 3}?00??0?-
?0????????0??????????????????0??????0????????????????0?1??0???????10??????????1??????????0??-
?????????1??????1???0??????1100???????????????2???????????????????????????????????0?????????00??
?1??1????0???0?0???1?0???0????????1??????????N0????0??N???????????????????????1??????{0 1}{0 1}-?-
????????0???0-??????0?

Hebeiornis ???----

NN???0?????10?????????0????????0N????????0?????????0??0?????????012000-0{0 1}0??????0202--
?11?????????????0???????31?????????????1?1?{2 3}1?2?1?N2??200?0???1N10031??-?(0 1)??-
0?1????????0????2????{1 2 3}12???1???10?1???????111?NN0?0?020001????-????1??0?0-???0???10??2?10---
????0?30?1-?{1 2}????0?00-??10???0-??101????(0 1)0???0??011?3-?1????-0-01???0?????1110--??2?01?0?10-
??02???1{1 2}1?2?111?10?00110??-?-1?0?1010-3?010000?00?????N?-
010????????1????12?0???01????0??0??0???????0?1??0?10??0110??????0-
0??????0????????????0??????????????10??0???-??1001?-??-??0???1???2?N00?????0?????01?????????00----
??2???1???0?000?????1000001??0?0?0?100?100?N112?101?1???????1N001111?0NNN--????????00??{0 1}?{0
1}0???10?001?1{0 1}-?????1??0?0(0 1)110?0????0??

Gobipipus

?10????????????????0???????????00?????????N???0????????????1???11????????????1?{0 1}1?????????1-1--------
-?-
?????????0?????????????????????????????????11?N2?0201?000??NN?0??????????????01????????????????????
???2-{1 2}10??0???1?0??N?????????????103-??????????0-???????101?3?{1
2}0??????????????????????00???10?1{0 1}2-?01?0??-?--?0?????????--???-????2?????-
???1?100??0N???????0???--?{1 2}????1(0 1)-2111??1000?N???????1?????????????2??0??0(0
1)??????????0??00?10?1?????10??0???????00?1--????????0(0 1){0 1}0??0??????1??0????????0??1??????0?-
0????--?00????????1??1???????1?????-0????????1???2{1 2}N0?0????????????????????????---
???0????????????????????????10??0?0??101????10N???0??1??00????{0 1}?N000??1{0
1}?NN????????????????0?????0??????????????????????0??0????0????

Gobipteryx ?1?----0??0????1??00?0?????010000?????????????-

?0?????????0???????101?00110?0-?10?1101-1---------?-?010?0??-111??0??{2 3 4
5}10?0?0???????????????20?102-1?{0 1}???0???????3???-????-
2222??02010?????????????13??1????1???10110101111110000020001?03--00????1??1-???0-????113?1?---
101????00???21???-???00?10?1?2-10?1??0-0--0??0??11103--?1??1-0-0120??-??-111{1 2}0--00?1?10???1-0--
2???121-?111???00??{1 2}????-1?1?01101?-210??00??000-??1??-???????1??0110???????0??0?1??0?01--
0??????10?????0?0????0?1011--????????11000?0?-0???0--?00?10???0100??0110-?1??020--?-0?110?1?-?21(0
1)(0
1)0100010011?????120???00?0??????0???10??011????????0?00010?000001?0??????????0???0?0???212?00?
???10000???-?000?0????00101110?00?01020-0????0???1??1?0?00?0???0

Nanantius

??
??
???????????????00???1111???0???????????????????
??
???0???101???????????????????????????????????????
???????110???
??10101?0
0???????????????????????????0?????0

Gurilynia

??
???002-
??
????????????1??-
???-
?1????????????????????????????10??111
1?1???
???00?0???0??1??0011?0012201??1?0???????????????????????
???0????

Holbotia

???????N??????????????????????0???????????????????????????????????????0???01?1????0????0??20{1 2}--
?220??????????????????????????0?????????1????2???{1
2}??????????????????????0?1???????NNNN100?0?0001?03????????????0-
?2????1?????0???????????0?01???1?11?????????0???0????1?????(0
1)0???????11??????2???????????0???????{0 1 2}0??0?????????0???1????1??????????????1{1
2}?????????0?10???3?0??0???0?????0?0????????1??????????????
????????0???????0????????????????????????1???1?20???01???????????1????0???NN?00??????????????????
????00----??????11???0??????{1 2 3 4
5}??0?0???0N?N???0????????{0
1}?????11???0??0?11?????????????0??0?1???????

Evgenavis
??
??
?????????????????????????110000?????1???1?0??????????????
?????????0?????????????2??
?????111???3???
?????????????????????????????????????0????????000??
??
?000?0?011-0????????????????1???????
Enantiophoenix
??
???????????????????????????????????????{1 2
3}???2?1?02??????0???????????????????????????????2???????????????????????????????????????11000?0??0?1??
??????????????????0???10??3?0???????0???0???12?11???????????????-
???0???????????0??1????????2??????????????????1??????????????????????????110?{1
2}1??????????????????????10?0?2{0
1}0??1????1?????????????????????1???????????????????????????????????1????????????????{0
1}???????????????????????????????????????0???0??????1?0?????????????1???{1 2
3}???0?0?01??00000?1?????????????????????????
?????????1??????????0?????????????0?00??1{0 2}-??????????????0????0????
Parvavis
??1?10????????????????????????????????
??????????????????????????????---3?-
???????????????200?0??1??????????????NN000?0200
01?????????????????????(1 2)-?????3?2??????????00?0-10?1???-?????????{0
1}?????0??????????0????????????????????????????1?????????????????????????????????11???????????????
1?0?10?0?2?01?0??-
?????????????????????1?????????????1?????0???????????????1?10?????????????????????????????????-
????????????????????0?????1?1000??????????1???2???00???
????????????????????00110?0??{1 2}???1??10????{0 1}???????????????????????????????????100--1?11-?-
???1?????????-????????
Monoenantiornis
???????N????????????????????0??0??????????????????0??N???0??0??????????{0 1}?120?0??{0
1}?????0?0201--022??1????????????????????21??????---3?-?11?{2 3}1?2?1?N2??200?0????NN003(0 1){0 1}0-11??-
{0 1}?1???????????????????{1 2 3}????10?????0?1{0 1}?0010?0N1NN0?0?0{0 1 2}000{0 1}1131??1??1????0-
???0?-110??3020---1??0??30?0-11?110?-00????0???1-???0?????0???????{0 2}11??111???1?????????0?????1{1
2}0??0??1???0????1?0{1 2}???111?2?11??100?1N0???-??1?0???1?-2?00??0?-0?????????00??????1???????1{0
1}?0????????-???0???????????1?1??0??0????10????????????????0???0???-
????1????????????1???0?????11000??1???????N????2?N00?????????????????????????????N????11????000
??1{4 5}?{1 2}000?01??0?001?100???0?0??1???1??????????N000??1??0??????????00?1??0?????0?0???1?{1
2}1??-????????00??0-?0???1?1
chabuensis
??
??????????????????????????????????????{2 3}1???1?02?1210?0????????{2 3}??--?-?--
???????????????????????????????1???1??0?????1111??{0 1}?0200????????????????????{1 2}??????0??3?1?-
--??????30??-12?1?????0?????????-
???1???1?????????????????????????21?2?10??10100{0 1}1??--
??1?0?1??0-2?00?00??00????????1??????11??1???????0???????0?-
???????????????02?10??0????00???????????????00?0??????????????????????????????????1?????????????
?1???2??1???????0?????1????01??0?????0?0??10????2???1??????
??2?00????1?0?????????????????????????1????????-?????????????????????????
Junornis
?10????0????????????????????0??0??1???????????????0?????????????????????{0 1}00200?-??0????0?020{1
2}--112???0???????????????1?2????1??---3?-101?{1 2 3}1?211?02-1200?0???111002000-0111-021???0????20?--
2122121{2 3}?21????1???1??0???101111000?0300011031??{1 2}??1????0-0{2 3}1?(1 2)-110103?10---??1?0?30?1-
12?1100-00??{1 2}00???{0 1}-?0?0?1???100??0--?11?3?211????0?0??0?00????1100??00210??0??0-?00?{1
2}11111-2?101?101001?110-{1 2}-1?0?11?0-3?010000-00?0?????-0??????11??1???101?0????????-
???0??????????1101?100??????101??{1 2}??0-?0????????0???-?-
?????????????????1??00????011100??00???1??-1??02{0 1}000?????????????????????????0?----
?0???111???000????5?1010?010?0?00?11000??0?????0??1????????22000110110???-
1??0?????????????0???111???1?10-?-???0????01?10-?1?00?00
Eocathayornis
??0????0?????????????????????0??0??????????0???0???0???0?0??????????????02000???0???000?20{1 2}--
001????1?0?????(0 2)????{0 1}??????????---3?-1?1?21??1?002-121000??01110020{0 1}0-110?-
???103???1??1????0-??0???11010{2 3}?00---
????????????????????00??100???0-?0???1????0???????1???-?1?????0?0?????0??0?11{0 1 2}0??0?{1
2}1?110??????02{1 2}???11-?11(0 1)1?1001011?01-??????????-???10?0?-
?0????????0?????111??1?1?101?1?????????????0??0??????????1?1000???0?10???????????????????????-
??0??????1??????0??1???????1??????0????????????2?0?0???????????????????1?????---??0(0
1)??110???0??0??1{1 2 3 4 5}01???00100000??100001100012201 11?011?1{0 1}?120000111{0
1}0????????????????????????????????????-??????0??????-???001??
Houornis ??0????0???????????????????{0
1}?0???????????????????1?0????????????????????????{0 1}?02?0??????????0???0{1 2}???--
?????????????????????3???0{1 2}??---3?-????{2 3}1??????2???2{0 1}0?0???1?100?0?0??{0
1}?0?220??1??????????????????212?21?2?{1 2}????10?????101111110?0{1 2}00?1?????????11???0????00-
?????3?10???1010?????1-11????0-00???10???1????0????0??00????1???????1?-??????????????0?11{0

1}0???02????0?????????????????111?10000200?1?2?1?0?11???310???0?-???0??????00???????????????{1
2}00?0??1???-?????????????1??1?100??????101?{1 2}2?????01??1?0?00??0??-
??????0?????????????00?0???11?00????????????????2{0 1}0?01?????????????????????????---
??0???????????000?????1?????0??0???????00?????????????????????210000101?0000????0????????????????1
10?001?10-?-?????????????-?0????00
aberransis
???????0?????????????????????????????????????0???0?????????????????????1??????????????0?01???{
0 1}??????????????????????1????0?---3?-????{2 3}1?????02-
??00?0????01??????????????????????????????{1 2
3}???????1????????1???????????????????????????????1????{0 1}??????-??0??3?{1
2}???????0??????????????-??????0???0-???????????0?????????????????????????????????11{0
1}??????????0??????0??????1???111?10???????????????????????????0?-
??????????0??????????????????????????-?????????????????1??????????0???????????????????????????-
??0???????????????????????1???????????????1???{1
2}??????????????????????????????????0?????????0000??????????0???0??????????????????0??????????????
00????1??????????????????????????????????????-??????0??????-????????
Cathayornis_yandica
?102???0???????????????????0??0??1??1??0??????0?00??????????????????????12?00???0????00020{1 2}--
0--?????????????2????????3????2??---3?-1???210201?02-120000??11111030{0 1}0-010?-?22?11??????02????{0 1
2}??{1 2 3}????2{0 1}?2-21??110?0?????11111?0?0?0??01031??{1 2}??11???0-0310?-11?1?3110---
11100???????0?????-00??200?1{0 1}1-?0???1???10?0?????11??-1?1?-?0???0????0??11110?{0
1}0020?1?0?????0??11??11-211110100001010 1-2?1?0?1???-{2 3}1010?00-
00?0???1??0????1111??1?1??0110?0?1?10?-???0???????????001?10000??0010???2??????0??100000??0-?-
??0???0??????????0??0??1?1??1??????0?0?1???1??02{0 1}0?01?????????????????????????0?----
??0??111??000?0??{1 2}??10?0001??000??11000110001 0201?10?01??2?210000011{0
1}0000????0???00??1?0?00?0???0????{1 2}?-?-????????01??0-???0????
Sinornis
????1???0?0??????????????????????????????????????
?????????????????????????0?200---31-???????2????{1 2}??????0??01??003000-0000-{0 1}{1 2}??11??10{0
1}202--201{1 2}1112121???210??1?????????1111??0?0{2 3}0000?????????????????03??(1 2)-11???3?20---
??100?3001-1{0 1}????1-00??1???1??????0?????????1?0--?1?????111???0??????????????1?{0
1}?????????????????????11??????11?1000010?01-?-1?0?1010-220??10?-
0???????0?0???????????1?????11?0??11???-???????????????0????0???0??010?{0 1}?2100-00???????0??????-
???????0?????1?0???0?21???010??0???????????10??2???0?????????1???????????????????????021?1???00???
?????0?0??????????????????????????????????????10100111{0 1}0000-100????????????????????10???1011-0-
????????00110-????0?0?
Linyiornis
?00????0????????????????0?????0???????????????????1??1??0???????0?10??????0???????????????000?????0?
?0?0????????-2???????2????2?0---3?-10??{1 2 3}1?2?1?02-
1210?0?1010100?0?0?00?0?1222110?111211???03?121{1 2}?2?02-???0?10?0???101111000?0??001102???{0
1}?????1?{0 1}????0?-?101130{1 2}0???01100?30?1?1{1 2}?????-001?11??1{0 1}?-
?0?0?0??0?0?000??0???????12-???????0??0????11{0 1}0??00?1?110???????{1 2}???111-
21111?1010020??0?2?1?0111?0?22000?0?-00?0??010?0?0???111??1?0??010???001?0?-
??????0???????1010?0?????0?101{0 1}{0 1}2?00-0?0??0?00????0??-
??0???0101?????01??0020???010000?????????-100?210?0?????0??????????????????????---
??0003111??000????????00000100000 0?1100??1??0102?00101??10{0 1}1?200?0001{0 1}?000-
1000?0??0????010?100?0?00?011-?-1???????10?00-0100?0?1
Gracilornis
?1??????????????????????????0??????????????0??20??????????????????????????????????2??0???0??????0???????
????1???(0 1)????{0 2}???????{3 4}0?0?2??---3?-?01?2??2?1?02??200?0???1?10030?{0 1}-?{0 1}??-{0
1}2?????21?020???{0 2}????{1 2 3}0{1 2}?21????1?0?12?0???011111000?02000{1 2}113???{0 1}???????0?0{1 2
3}?01-?10??3?00?????10??30??-12????0-00????0?1{0 1}?-
???0????????????0??111????112???0?0??0????????1100???02?01?0??0????{1 2}???12??2?1(0
1)1?10??010??0???1?0?1101?3{0 1}01?00?-?1?????????0????????0??1?????2?????????1?-
???????????????0?1??0??0?00?10???{1 2}000??0???1??0???????-
???????0??????????????0?????01?01?????0????-1???2?0?00????????????????????????0---
??00???11???00?0??1???0?0?01??0?0?????0??10?0102???11???0??1?20001?01?0000???0??0?10????0?0??00
10???0?10-?-???1??????110-00???000
Alethoalaornis ???????????????????????????{0
1}?1?????????????????????????????????1????????????12?0??????????0-
???????????????????????2?????????????????---3--????{1 2 3}1?2?1?02??200?0???1?1?03???-?0??-
????????????????????????????1?????????????????????{2 3}????0{1 2}?00?????????????????0??????-?101?2?{0
1}0---??????{2 3}??1?0??11??-00????0????-
?????????????????????????1?????0????????0????111???0??1?1?0????????????1?1-2?1????01?{0 1}{1 2}0???-
??1?{0 1}?10?1-2?01??0?-
??????????0??1?1?10?????????0??????????????????????????-
?????????????????????????????1??0????????1????????2???00??????????????????????????????????0{0
2}?????????0?????????0?0?01??0??????????????0???1??1????????????1?0???1????????????????????????????0
?????11??-?????????1???0-????????
Cuspirostrisornis ??????????????????????{0 1}?????{0
1}?0??????????????????????????????????????{0 1}?01????????????00??0{1
2}?????????????????????????{2 3 4}2????????--3--????{2
3}10?????????200?0????11????????????????????1?????????????????{1 2 3}1{1 2}?21?????????1??1??????1?11??{0
1}?0{2 3}0?0{0 1}???????????????{0 1}??????-?????3?0???????????30??-???????-
??????????1????0???????????0?????????????????????????0????1?1???????????0??????0???????????111?10?0?
????????1?0?11???{2 3}?0{1 2}??0?-0??????????????????0????????????????1?0?-????????????????{0

1}?10???????1??????????????0?0????????-???????????????????0??????1?0{0
1}0??????????1???220?0???????????????????????---??0{0
2}?????????0?01?????????????????????1?????0???0??1???????????0????1???????????????????{0 1}?{0
1}???0100??1??{0 1}-?-?????????????-??????0?

Dapingfangornis

???????0????????????????????????0????????1??????0???0?????????????????0002000-
??0????000?01?????0????????????2???????{3 4 5}???????---3?-??1?{2 3}1?2?1?02??200?0???11100{2 3}???-
?0??-??????????????????{2 3}{1 2}??????1????????1??????1?1111??0?02000{0 1}?03??????1????0?0????-
?10??20{1 2}0---???0??30?1-?2?110?-00????0???0-
?????1???1????0??111?3??1???????1??0??0???11110????2?0??0??0-??0????02??2?101?100?010???-
??1?0?11?1-{2 3}?000?00-?????????-0???????1??????????0?0????0?-
???0???????????001?????????010??????????????????00??????-
??????????????????????0????0?1100??0????????????03?0?0???????????????????????0?----
?00?????????000??0??10?0?00??0?0???1?0???0?010{1 2}?1?1??????????01011?1?0?????????????????{0
1}?0???1?0?????{1 2}{0 1}-?-???0????101?0-?0???0?0

Largirostrornis

????????????????????0??????????0???00{0 1}????????????00??0{1
2}???11?????????????{0 2}?????????????????????????210????02-
?200?0????11???????????????????1?????????????{1 2 3}????{2 3}??1???????????????????1?11??0??????{0
1}???????????????{0 1}??{1 2 3}?????????3?{1 2}???????0?????????????????????????????1-
???0?????0??????????????????????????????0?????11???0??????????????0?{0
1}????????10????0???????????1?0?11???3?00??0???????????????????????1?????????0??????????????????
?????{1 2}?10????????????????????0??1??0?????????????????????????????0??????1?0{0
1}????????????1???2?0?0??????????????????????????---
???0????????????01????????????????????????????????0??1???????????1????????0?????????????????{0 1}?{1
2}???1?0?????{0 1}{0 1}-??????????????0?????????

Longchengornis

??
?????????????{0 2}???????{3 4 5}???????---3?-????2??2?1?02-?201?0????0100{0 1 2}??????????{1
2}???1?????2{0 1}?--2?3?1212?21????????1????????1?11??{0 1}?0??00{0 1}?????????????????{2 3}???-
?10??3?{1 2}0???????0???????2?????-00?????????-???????????????--
???????1?????????????????????11????????????0????????????121?21111?100??{0 1}??????-1?0?11?1?{2
3}?01??0?-?1?????????????????11?????????1??????????-??????0???????0????0???????1????????-
?0??????0???????-
???????????????????????????1??????????????00??1???????????????????????????????????00????1????0???
?????0?0?01?00?1??10?1???0????????1?????????1000???1?????????????????????????????????????-?-
????????00?10-????????

Microenantiornis

???????NN???{1 2}?0?-?{0
1}?????????????????????????????0????????????????---3?-????{1 2 3}1?2?1?N2??200?0????NN00{2 3}???-?{0
1}??-??1????????1111??0?0{2 3}00?11??????0??????{0 1}?0????-
?10??3?1?---??????30?1-12?????-0??????????-
???0????????????0??????3??????????????????1???1110?????????0???-???????111?2?111??001120???-
??1?0?1??0-{2 3}?0{0 1}?00?-?????????-0??00????????????1??1????????-
??????0???????101??0???????10?????????????????????????-
???????????????????????0??????1100???????00??????21N??1?????????????????????????---
??N0????1????00???{1 2}??{0 1}0?0?00??0?0???0?0?1?0?N??{1
2}???111????2???N00???1????????????????????????????10???1?{0 1}{0 1}-?-???1?????0?10-????????

Piscivorenantiornis ???????0???????0???010?????{0 1}?0?????????????????1-
00??????0???0?00??????{0 1}0?2000????????00?20?--?12??0???0??1?-201??{0 1}??{2
3}1?0??0???????1???210211?02?120000???10100?00??0?0??122??1?2???20?200?2{1 2}011101?02-
??????0???????111101{0 1 2}?0?0??1????????-?????{0 1}-
01102??10??3?10???0?1?0???0?????????1?00???10?1{0 1}0-?0?0?1??{0 1}(0 1)??0??10?????1??12-
?????????00???1?110?????????1???????0221?111??1111?1010???11??101?0?110??2{1
2}01010??00?????1???0??????0??1????01?10?????????0??0??0???????101?1001???001???????0-
?00??0???0???1?????000????????1??1?????????0?1??1??00????10?10??210?00????0????????????????0?0?----
?00??110???0?00????01000?01??00??????00??00010{1 2}0??10?0???2?2201011?1{0 1}?111-
1??0??000???10???????00???011--???????0?00110????00?0?

Pterygornis ?10----0????????????????????0??????1?-
??000??20???0??00???0?00?????????1??0(0 1)(1 2)000-?{0 1}?????00020{1 2}--??2????????????-201????030{0
1}1?20?---3?-111?{2 3}1021??02???2(0 1)0?0?1?11100300?-0?0?-112??1??111{1 2}?1??{0 2}0{2 3}??11{2 3}?2?02-
210?011???????1111110?0{1 2}0?01103?-?{1 2}???11??1-?30?(0 1)-1?0??30{1 2}0---1?10?0300??12?1???-
00??210?1{0 1}0-???0?1??{0 1}10?000???11?-???12??-?-0120(0 1)00?-011110--0?210?0???0-???2?1?111?{1
2}111101000?1?11?-2?1?0?1111-3001010(0 1)-00?0??010-?0???1??0??1?1?10{1 2}1{0 1}?00????1?-
?????10???????001?10000?10?1????{1 2}?00??00??1??00???0??-??0????0?????1?0???0????-0?1??0?-??-
01?10010?02{0 1}0?00?????????????????00?0?0?----?0???111??00000??04010(0
1)0?01??00000?1?00100001010(0 1)0101000000220(0 1)001?1{0 1}0111?001?????0100???{0 1}?10??0000(0
1)010-1-???0?00?00110-???0000?

Dalingheornis

???????N???????????????????????0???2???????0????0?0???????
????0?????????0???????3????????{1 2}{0 1}{2 3}???N1?N1?1?1?N2??20000??0NNN00??????{0
1}???111??0?2????????????2???12?2{0 1}????1??????0?????N??{1 2 3}000?0{2 3}000{0
1}103???0???1???0?02???0110??3?10???0?????{1 2 3}??1????11?-?00?????????-
???0?????????0????????????1?2???0????????????N100????2101?0????????11?021?2?10101?10{1 2
3}??????1?1?0?1000?3?00?00???0?0???????????????1??1????0?0??0??????????????????????1?0?00??????01
?1?????????0?????????????????0??????????????1???0??????110{0 1}0????0????-

0???2???0?????????????????????????????N0?2??1????-?????2??0?0??1??0?????0?1-
??0?N???0??1??????????N00?001??NNN????N0?0?????????????0???????11-?1????????????10?0?????0?
GMV2158
???????N?????????????????????????????????????0????????1???????????????01?0?-?20??100?0201--
02{0 1}???01?0???1???????1?1?????0????????N1?{1 2 3}N?211?N2-
1200?0???1NN10??????????????????????212--20{1 2}?1{1 2}12?2??2-210?01??0?????NNNN00{0 1
2}?020?011????????111??{0 1}-02??0?1101131{0 1}???????00?10??????11?0?00????????{0 1}-
00?0?0???1????0--????31????-1?0???????0???1?11?????21?110???-?0??{1 2}10121-21101?100?????????-
1001100??2?00?00??01???????-0??????11??1???????0??????0???????????????N(0
1)00?100?1????10??????0-00??????00??0?????????????????????1??????????1??0??0????1?1-
10??2?N?0?????????????????????????---????031???N?1?0????{2 3
4}?N0100??????00?10??0??0?N???0??1??00????0?N001??1{0 1}??NN-1?????????0??????001??0--1?21-
????????0?00?10????0??0?
GMV2159
???????N?????????????????????????????1???N??2?2??0???N?????????????????{0
1}?11??0???0????????01???2?????????0????{0 2}???????????{1 2}??---3?-??1?{1 2
3}N?2???N2??200?0?????N?0?000??0?0????????????????????????1???10{1 2}??1??0???0?????N??N??{0 1
2}?02000?1?3??????1????0?0???0-1?0??3?0{0 1}?????????10???0??110?-?0???00???0-
???0?0????????0???11?3????????0?????????????1100???N210110???-??0{1
2}???1?????11??10??0N1?00???1?0?1??0?2?000000-0??0?????-
0???????1???????02?0????1???????0??000?????1?1?10?11????10?????????????1???????????-
??0????????????????????????01??????????1????????2??00????????????????????????????????{2
3}??1??0?0?-???{1 2 3 4
5}?N000?01??0?0?????0?110?N??2???1??????????2N0??0?1?0?????????????0??????????????0--1???-?-
???0??0?00?10-?????0??
Liaoxiornis
???????N???????????????????????????1?????NN???????0????????1???????????????00000????????0???0{1
2}???11???0??????1??0??????1????1??---3?-?NN?{2 3}N?2?1?N2??200?0???1NN00??????{0 1}???0{1
2}2??10???????????????{1 2 3}????1????1????????????????N??0?020??{0 1}103??0????????0?0??0(0 1)-
?101?3?1????01?0????????????11??-00????0???1-?0??????0??00????????????1?????0???????0????11{0
1}0????210?10??????0{1 2}???121-11111?1001?N?????1?1?0?10???3?01?00?-
0??0?????????????????1???????0?00??????????????0??0???????101?000?????01?10???????????????0???????-
??0???????????????0???????????1?????????????-
0???2??0????????????????????????????????N??2?0???010?1??1??N010?00??0?0???0?0-
?10?N??00??1??00??{0 1}?2?N0011?100???????N00???????????????1???--???{0 1}-?-??????0?01??0-????????
Avisaurus
???
???
?????????????????????????110101?????1???0?????????????????
?????????0???????????????1???
?????110???2??
??0????????020???
???
??0000?01110???????????????0???????
Gettyia
???
???
?????????????????????????13110??????1??
?????????0???????????????1???
??????10???2???
??0????????010???
???
??00?0?{0 1}1{0 1}10?????????????????1???????
Mirarce
???
?????010???0????0???0????????---3?-????3??2??002-?1101????1?1??????????????????1???1??????????????{2
3}???{0 1}?02-21?011??0???1?111111010??0?1??????????????????21??-?1?1?30????????????2000?12?1???-
?????????0?-???0?1????????0??1?????????-???????????????111????????????????????211?1??-?11?1??????{0
1}????????00110?1?2?0101??-
????????????????1?1?????????????0??0??0???????????0????0??11101100?10?????????11????11110?????1??-
???????????????1?????0?????111000??0???1???1??2??111????0?{0
1}????0{0 1}00000?00?100111?0010010102010100000????{0
1}?0???????0?11??0?????00110???0?????0000?010-0-???0????101?0-10?0???0
Soroavisaurus
???
???
????????????????00???1111100010?0??1??1??1??1???????????????
?????????0???????????????1???
???0?1100??2?0??0???????????????????????????????????????110??
???????100?????????????????????????????0????????010???
???0000000
0?00101021-1???????????????00?????0
Neuquenornis ???????????????0???????????????????????????????????-
???01???????10???1?3101?1?02
-
?20?00?1?11110??21??1???0????????10100020?00????????0?1?0

???03?????101?3?1??????????30???1{0 1}??????0???21???0?-
??000???????????????????????????????????????1?????????????1??????{0 1}??121-
??111?1000?1???????1?011001?3?0{0
1}?00??01??????????0????10??1????????????1????01?????00?????0?{1
2}???0?10???1???1??????1?00??????1???1???3?????????
????????????????????????0???111??????1??1???0??001??00?0011?????0?????0??10??1?1{0
1}?2?0001??10?????????????0?1???????????0??1010-????????????????0???????
Intiornis
???
???
????????????????????11?111010?030?00???0?11????????????
?????????0???
??????100??3?0??0??
??????????????????????????????????????0???????000??
???0???
??0?10??11-?????????????????0?????0
Enantiornis_pectoral_wing_only
???
???01102-
1100011?11100??
???????????010??2???????????????????????0???21?????-
?0???1??????????????????????????????????????1????????????????????????11-
2111111000?????????????????????2?????00?????????????1101011101?2???????????????????????????1101
00?101??1??0?????????????????????????1???2?????????????
???001000000010000110101120??0???11{0
1}12100011010???0????????????0????
Elbretornis_holotype_only
???
??11?2??20000?1??????????????????????????????????????
??0????{0
1}??????????????????????????????????-
?????0??????????????????????????????????????1?????????????????????????0???1????????????????????
????????10?????????????????????1?1???????????????????????????????????10110000-
???{0 1
2}???01??0000????00100101221101100010??2?
???
Martinavis_cruzyensis
???
??2100???
???
???????????1??1??????????????
??????????????20??1?1000?100?????????
???
???????????????????????????????????????001101011{1
2}01010100???
vincei
???
??2000???
???
???????????1??1??????????????
??????????????20??1010010?00?????????
???
???????????????????????????????????????001001010201011100??????????????????????????????????????
???????????????????????????????????
saltariensis
???
??2000???
???
???????????1??1??????????????
??????????????20??101000??01?????????
???
???????????????????????????????????????001001012101110000??????????????????????????????????????
???????????????????????????????????
minor
???
??2000???
???
??1??????????????
??10110??0???????????
???
???????????????????????????????????????001?010122??
???????????????????????????????????
whetstonei
???
??2010???
???

??1??????????????
???10100??0???????????
??
???????????????????????????????????????0?1?0001210??
????????????????????????????????????
Cratoavis
1??????N????????????????????????????????N??20???0???????????????????????????????????????0??{1
2}????????????????-????????????????---3?-??1?????11?N2-121010?????100?0?2??{0
1}?0????2?0??1?????????????2????{0 1}02?2?????????????????000000300011????0???1???????31?0-
??0??3?{0 1}0?????????20???10?11??-?0??21???0?-
???1??????0?10??????????1????????????????1111??????1???0???????21(0
1)?????21111?100001 1????????0?1001?2?00?10?-
0??0??????0????1????????????0????????????????????????????1?00??0????10??????????0??????????0??-
??0??????????????????0???????102????????11?0???2???0?????????????????????????????N????01???00???
????????001??0?00?0?01-1???N???0??1?????1??2?N0????1{0 1}0???????????00????????????00??1?{0 1}0-
???????????????-????????
Qiliania
??
??{1 2}?1?21??201--
201?1{2 3}13?0{0 1}???21??01?10010111111??0?020001????????????????????????????0???????1000300 1?1(1
2)????0?????????1??????0????????0?0--
1????????2?1???-10011100?3{0
1}0????????1????????????????????????????????110?-??????????????????????????????{0 1}{0 1}??00-
?0??1?0000000??0?????????????0???0??????????0????????????????0????????????????????????????????????
??111-
10?????0110?10100000?00?01?{1 2}{0 1}-?????1???????????00???00
Feitianius
??
????????????????????0??0??2??---30-??111221--202{1
2}1111020????1?0?1????????1111000?020011?????????????????021?0-??????????????100?3011-12?1100-
???????????????0??????????0--
1???????1??????????????????1??????????????????????211??????????????????????-100?1111?310?????-
??1?????0?????????????????????????1?0?-?????????????1??????????????????2100-?0??????0???????-
???????0???????0????010???1?100???0????????0??1?10?0?00??
???000-10?????0?????????0??100001010-?-
???0?????????-0?????0?
CAGS_IG_02_0901
??
??1?02??20000??01?10030?0-11?0-
??01???10---
???????????????????01??{1 2}??????-
?0??????????????????????1????????????????????1??????????????????????????11-??111?100001???1-
??????????-
???1??0???0????????0??????????????101??????????????????????????11?10??????010?????????????????
???????????????????????1?????????????????????????1???2??
?????????????????1??0?0???0??01?010???0??????????1?20001101?0??????????????????????????????????
????????????????????????
CAGS_IG_04_CM_007
??
???1????????0???{
1 2 3}1{0 1}1211??{1 2}???11????????111100{0 1}?0300?{0
1}???????????????????????????????0???????10?03011-100???0??????????{0
1}?????0??????????0???????????2??
1?0?10?0?2?0??1???????????????????????????????????00???0?-???????????????????????????????{1
2}??0??00??1?10????1?????????????????????????0?????1?20???
??1???????????0?01??
???11?100????11-0?????????????????????0?
CAGS_IG_04_CM_023
??
??{0 1}??0?0?11100{2 3}000-(0 1)000-
???10---
????????????????????00??2????????????1??????????????????1??
??????????111?1000010?00-??????????-???{1 2}0????0?????????0??????????1????0{1
2}?????0????????????????????????????0?010?10???
??????????????1???{1 2
3}???10111??--
21??0001101?0???-?????????????????
Boluochia
??????????????????????????????0???01????????
??????????????????????????????????????1?{1 2}1???1?????21?--
2?110{1 2 3}1{0 1 2}?2???????????????????N11N010?0?0001?13???{1 2}???????{0
1}??????????????????????1?0??0??-?2????1???????0??{0 1}{1 2}????1?????0??0?0--
?????????2???????????1?????1?{0 1 2}0????????????????0??????????????????????????-??0?111??3?0??1??-
??????????????????????????????1?0??????????0??????????????????????????????0-
00??????????0????????????????????????????????01??00???????????0????????????????????????????????????

?????????????0?{0 1}???{1 2 3}?{0 1}???-
???????????????0?????00000?11-?-?????????????-??????0?
Longipteryx ??0----
0????????????????????01?0?????????00?00???0???????010???????????0?12000-?{0 1}0????001(1 2)0-0002-
0??0010?0?0?2??????03????2??---3?-?11021?201?02?1210?0???1NN002000-0000-?????1021??212--
2012?311?21?2-210??10?0?????N?1N000?010001103--02??1?1??0-0110{1 2}-110103010---010?0?30?1-02?1?00-
00??2?00101-00?0???-000?000--111-31-111-?-1-0100??0????1100--00??00101?0-?-0211?111-21111?1010010111-
1-1?0?10?0-2200?10?-00?0?0?1?-0??????11??1????0100?1?0??0?-???0??0???????001?100?00?0?10100???0-
?0??????000???-?-??00??00-?????0?0??00?0????1100?-0?-0?1??N100?21N111????????10????????1????0---
???0?{0 1 2}0110000000??1301010001000000??1000?1000N??20?11??00??0?{0 1}?N000101{0 1}0NNN-
1?0N?000010????{0 1}???1100000?10-0-???1??0110010-???00000
Shengjingornis
???????0???????????????????????0??????????0???????0????????????????????????02?01????????0?1?0-??0--
?????????????????????3????2??---3?-??1?21?2???02??210?0???101?0200?-?{0 1}??-????????????20?--2?1{1
2}?212?2?01?{1 2}10?11??0?????1111?00?02000{0 1}1?{2 3}-???????????0????00-110??3?10---??0?0?30?1-1(1
2)????0-00????0??{0 1}1-???0?1?-??????0--??????-1120????????????0????11{0 1}0????????010?????-
0????111????11??101?0{0 1}111?-?-1?0?1??1-3{0 1}00000?-00?0??????0????????1??1????0??0????????-?????-
????????0?2??0??????010??????0-?0??11????????-?-
??0???????????????1???010????1000????????????10??2??00??????????1?????????????????????00???1????0000?
?{1 2}4?1000?00??0?0?????0?1?0?0??{1 2}??01?0?????????0001??1?0000-??0????????????????????10?????11-?-
??????0?00?10-0?????0?
Shanweiniao ???????0?-
??????????????????01?0??1???????????????????????????????????????02000-?10????0?1?0-???--
???0?????????2?????????1??????---3?-101?{1 2 3}1?2?1?02??210?0?????????{2 3}??--?2?--
?????1?21?????????????{1 2 3}????102-???????????????1111000?020001103-??????1???0??????-1101?3?10---
??????30?1-01?11??-?0????0???1-???0???-????1?0??1?1?3?-???-????{1 2}???????-??1100????2?00????0-?-
0{1 2}???111-2111??????011??--??1?0?10??-2?000??0-?0???????-1???????1?????????????1?0?1000??????1?-
????????001??00???????0???????????0??0?100???1-?-?0????????????????1???0????001001??????-???-1??02{0
1}0?01??????????????????????????---?1????????????00???{0 1}{1 2 3 4
5}?10?0?00??0?0???000?????0??????1????????????????????0000?????????000??0?0???010?00??01-?-
????????00?10-0??0?0??
Longirostravis 1???????????????????????????01?0????????????????-
?????????0?00?????????????02?{0 1}?????????01120---0--??????????????2????????2?????2??---3?-
??1?21?2?1?02??200?0????11?02??--?-?--?{1 2}{1 2}2????????22?--20{2 3}?0?13?2??2-
2????10?0?????1111?00?030??{0 1}103-??{1 2}???????0-0??0?-1101?30{0 1}?---011?0?????-???1100-
00????0??{0 1}1-???????-0(0 1)0??0?????1?3--??2-???????????0????1100??0??0?0????0??-0????121-
21111?100?0{1 2}???--??1?0?11???3?0{0 1}??0?-???0???????1????????????1???1?0?2?0??1???-???1?-
????????0?1?1?????????1010?????????????1?0??????-?-
?????????????????????0??0??0?1????????????1???1???2{0 1}0?01???????????????????01?0?1?---
?100?????????000???13?10?0000??0?01??00001?000??{1 2}0??1?1???????2-0001??1{0
1}0000????0?0?0???2?????????10?00?{0 1}?1-0-??????????0?10-???0????
Rapaxavis ??0----
N?????????????????????01?0????????0N?????????0???N???0?00???????????{0 1}102000-?{0 1}0??1?01120-???--
0??0??0???????????????21?0?200---3?-??1?{2 3}1?2???N2??200?0???????002---------022??1?2?-220?--
20111213?210???10??1??001011N1NN000?020001113--?2??11???0-?3102-1101?3?20---01?01?30?1-02?????0-
001?{0 2}10?1{0 1}1-0??0???-?00?0?0--2?1031-112?1-0-1120??0????1100--0N?00010??0-0-0211?111-??111?100(0
1)011?---2-1?0?1010-3{1 2}01010?-00?0?????-10?????00??1?????001?0?0??0?-???0?-
????????0?1??1?0?????10?{0 1}0???0000??1???0?0???-?-??0???0?0????????0?000{0 1}0???01001?-?1-
??????10??2NN?01?????????????????????????0---
??N???111??00000??1301000?00??000????00?100?N??1?111??????11?-
N001001?0NNN00?0N??????????0?00???10?000?10-?-???0?00?00010-?1??-000
Halimornis
???
??-??????????????0????????????---3?-
????????1??0??0???1????????????????????????
????????????????????2???-
???1?30??????????????????????????????????????0??1????????
???????????????1??01-
??????????????????????????????????????0???????0???????1???{1
2}011???????????????????????????????????-
?????????????????1???111????0???
????????0?????????????000010???1120???????????????????????????????????????0100??????????????????????????
?????????????????
Flexomornis
???
???0??0??0????????101????????????????????????????????????
?????????????????0???1???????????????1????????????????????
???????0??21-
2???1???00???????????0??????????????????1????????????????1??????????????????100????????????????????1???
????????1??????????????????100??????????????????????????1??
??0???????????10???????????????????????????0??????1????????
???
Alexornis
???
???11002-0????{0
1}?1???0???1???

????????1031??????????0????????????????????????-
?1???1??0?-
?1????????????????????0??????????10?????0?????????????????00??????????0????1?????????????????????
??0??11?????????????????????????????????????1???
???0??????0??1?????????????10110001??????????????????
??????00?1????????????????????????1?????????????0????
Xiangornis
??
?????????????????????????????????????0??2???02??2{0
1}0?????111?0??
?????????10????00?????????????????????00??11?????-
???1????????????????????????1????11?10?00??????????????
???????????0??0?????????????????1???????????????????????????????????????0?1??0???????1???????????????
??????????????????????????????????????1??????????????1???
????????????????0?0?01??1?1??1??1??10?0?????????????????10001?1????????????????????????????????
???????????????00?10????0????
Eoalulavis
??
?????????0???001????1??????????????10??2012?1?02-121010?1???0?0300?-0{0 1}??-
021??1????????????????????????2-2?0???21????10103?10---
??????????????11????0??????10?-?0???0??{0 1}???0?????????????1??-
??????????????1?1?????????????????????21??111-2110??1000010?0?-1?????????-
???00?1??00?????1??0??????1??????????02???????????????0????(0 1)(0 1)?012?10000????1??{0
1}????????????????????????????????0????????1??????????1??????(0 1)???1??-
????2????????????????????????????????????0?3?1100????1??1?10010001??00000100101101010201011000 1?1
12?00????1?1???????01????????????????????????????????????00?10????0????
Liaoningornis
??
????????????????????????????????????1?30?2????{1
2}??????0??2-???0?1??000????112010?02000{0
1}??????????????????????1????{2 3}01??????????30?1?01?1?????????????{0
1}?????0??????????0??1?????????-
1????????????????1???1?0?1001?2?00?0?????????????????
????1??????????2?0??1?????????????????????????0??????????????????0??1??0?0?01???????????????????
????0??????1100??00??1{1 2 3 4
5}000???????0???????0?????????????1??????{0 1}?????????1????????????????????{0 1}?2????1?00??1211-
0????1???????????0?????0
Vorona
??
??
?????12-
0100111000000111121101020??1??????????????????????????????0???????????1??0?0?????????????????0????
?0??????????????2?????????-
1??100010???2???????????????????????
?????????????????10?100???001110000001?0?????????????????
????0????????010???
??0110101011010001 0?100-
0???????????????000????0
Lectavis
??
??
??????????????1?000??111111??00?????0???1?????????????????
????????????????????????2???????????1???
?????111???3??????????1???????????????????????????????011??
???????110???0?????????????????????????0??
??10000?1
0?1100??{0 1}0-?????????????????00????0
Hollanda
??
??
???????????11111000111111010002 0??0???0??0??????????????????
????????????????????????2??????????1???
???0?1110??3?0??1?????1??????????????????????????????0??010??????????????????????????????????????
???????000?0????0?????????????????????0????????021???
??10101012001
0?11001000-0???????????????001????0
Aberratiodontus
???????0??{0 1}0010{0
1}??0?0????0?0?{1 2}{0 1}??011???101??????????????????????---3?-????{1 2
3}1?001?02??200?0????????????????????112??1??????????????????12?21?????????1???????????1????0{1
2}00?010{2 3}0??2???????0?0????-110??3?{0 1}????0100??30?1-?{1 2}?1??0-??????0?1?0-???0????{0 1}(0
1)??0?0????1???1?1??????????????????11?10????2?00?0??????02????121?{1
2}111???00???1????1?1?0?1????{2 3}200?001-01????????00?????????????????10?0??????-
???00?????????100??0????????0??{1 2}1?????01??1???????02?-
??0???????????????????10????10?0??0??1??0??0??02?0?01????????????????????????---
????????1????000??1{1 2 3 4

5}?0010????????????1?1???0?0??????0??????????00????1??00?????????0???????????110?????{0 1}1-?-
????????00-10-???0????
Bellulornis
???????N???0?????????????????????{0 1}?00???-??0???????21{1
2}--021?????????????2???????0{4 5}??01??????????1N??{2 3}1?011?N2?121010?1??NN00{0 1 2}00??(0 1)100??2{1
2}21?1?1?????????????{1 2 3}?31?1?2-?10?01??0?????11N{2 3}000?0??0011?{2 3}??????1????0-
021????101?3?(0 1)1???1??0??2001?00?11???00??100??00-?0?0????0(0 1)?0?00??0????1?1??-
???????????????111?????2?01?0??0???0211?120?2111??10(0 1)0020011???1?0?10?0?{2
3}?00??00?01?????1??0??????11??????11??200??????-
???0??00?????1101001000???01000????????????????0?????????0??????????????0?????????01??0??00??????
-????2?N???????????????????????????---??N2??011????{1 2}01?01??10002100000001101-
0?0?N1010?01??000?1??111000?0{0 1}0???????N01?0?10110?00??0?0000??00?0????1???100-?0??1?00???
Archaeorhynchus 1101?000--?????????0???????0100?01--??0NN???12-001-
?N???10000??????11??01(0 2)001-??0??001-1---------?-01?0011??0{0 1}??????2110?1?0---3?-111?{2 3}1?(0
1)01?02?1210?0?1011100200?-11(0 1)?-021???02??20(0 1)0--20(2 3){1 2}(0 1){2 3}1212{0 1}?2-
?110?10?1000111111000?020001113-?0{1 2}-01?1??0-???0(0 1)-1101030(0 1)0---0?0001(1 2)0?0-?0?1100-
0010110?1{0 1}2-00?0?1?-?--???0--00103--112-1000002?0----11110010N2101101?0-0--2???121-
21111?10000211(0 1)?-1-100?10??-21010000-01?-??N1?-00?-?0??1??1?0?10000?1?0000?-?0?1--(0
1)???????101010?0??100101?02??0-000??0?1000?0?-0-?00--?0?01??????0??0010??1010100?-
??011??N100021N001??????????0??????0010?00----1N2???0???01101??1201010210??1001-
?101?000?0??1?10110??011?1?1(0 1)0010010NNN--?0N??00010{1 2}?012010010010?010-0-???0?00?00-10-?(0
1)00?0(0 1)0
Patagopteryx ?11{0 1}0??0--??????????????????01?0??1-?????0??102-?0?-
??????10????????11??002?21-??0??101-1---------?-1???11???{0 2}????????{3 4}????1??---3?-111?{2
3}101?1?02??010?0???10100{0 1 2 3}01??110???212????1-200?--21{2 3}?1{1 2 3}12??1????1???1????????111{2
3}100?020001?3-?02-??11??0-???02-?101?31{0 1}0???0??01?10?0-01????0-001?{0 1}10??{0 1}2-???0?1?-0--
???0--21103--1?????0?0020??--?-?11101?002101?0???-0--2???121-21111?100001010???-1?0?1010?3?010000-
01?-???1?-0??--??11?????????11?0?1??0????????1--?????????0?10?0?11??0?10??????00??????????????-?-?-0--
?0?01??????0??00????0?1100??-??01???110-020?001??0????11???????00?0???????10{1 2}???(0 1)1??00{0
1}01??1???010210??0?01-?101?010?0101?10110????11?11000??1{0 1}0?0?0-
???????1?0?10?21???10000011?0?0-???1???1000?1-?1?0?0?0
PMO228.582
??
??
??????2-
2???0??1??????????????????????????????????????0????
?????????????????????????-
??
???????????????????0??1010???????1???????????????????
??
???1??
?
Hongshanornis
1??1?1????????????????{0 1}?-?{0
1}0??10??????????????00101??0-{1 2}01?????{4 5}??0?2??---31-111?31?0?1?02-?200?0???11100200?-?1??-
?21??1???-202?--20321{1 2 3}?2?21?2-0???110?1?????1113111?0{1 2}000{0 1}????????????1????0{2 3}1??-
?10??30{0 1}0---??101?20?1-?{0 1}?11??-0010{0 1}??????-0??0????0?????0--0???????1?2-
??????????????111?????????????????0??2111111?2?111?100?02110?-?-1?0?10?0-3?0??00?-
?????0?1??00???????????????1{0 1}0-?0??00??-???????0?????1101?????00???101?{1
2}????0??0????000???0??-??????????????0?0?????????0?11?{0 1}???????1110?0??2?????????????1??????1{0
1}?10????????????{0 2}11011102010l????02010??1??1?01-11?1???1?0????????????????{0 1}?1000??0{0
1}00000-?0001?1100???????1010{0 1}100010?1-???????00-?0-????????
Archaeornithura ???????N????????????????????{0
1}??0???????????????????0?????????????????????{0 1}?02?00-?{0 1}0????0-0201--111?????????????????????{4
5 6 7}????{1 2}??---3?-?11?3101???N2??210?0???1N1002000-110?-{0 1}2{1 2}???????????????????{1 2
3}?????????1???1????????111{2 3}??1?02?00?0131??0???1???0-0????-1101?3?{0 1}0---???0??20?0-?{1
2}?110?-00???10???1-???0????010???0????1?3-21?????0????0??-????11{0 1}0??0??101?0???-000????1?1-
2?111?100?021?1?-??1?0?11?0-3?0??00?-???0?????-0????????????????00?0?0????0?-??????0????????1?{1
2}??0??0???0101??????????????????????1?-
??0??????????????????????0?10?0????????0??N1???21N?01?????????????????????????---
??N????1????0101??22?1000210??1?0?????1?0?0?0?????0?1??????????11001?0?0???????0????????????????0
1??????11??-??????0??0??0-????????
Jianchangornis
1???
??????????????????????{4 5}??0?2??---31-?1??31?1?1?02-?210?0??????1?0{2 3}0?{0 1}-1100-1?????????????2?--
202{1 2}1{1 2}12?21???01???11???????1112??1?010001?????????????????????0-?10??3?00---????0?20?0-
00?1100-00??{1 2}??????-???0?0????0???0--???????1?2??????????????????11????????????????????{1 2}??1121-
2111??100?120110-?-1?0?11?1-3?01000?-?1?????????0??????1???1?????????????????-
???????????????1?1?1001?????10???????0-?0???????0?0??????-
??????????????????0??????????011?0????????0??010??2??1{1
2}0{1 2}??1{1 2 3}??000210??1?001?1010??000???010000??????0?01{1 2 3}0??0{0 1}0???--??????????0????{1
2}???010??10?{0 1}1-?-???1???00?10-???0??0?
Schizooura
??
???????????????????{0 1}?0{3 4 5}????2??---30-??1?{2 3}102?1?02?1111?0??0111003122-1000-021??1?21-
2022??20???2?{2 3}?11?2-?1???1??1?????111{2 3}000?0{1 2}0002??????????????????{2 3}?0?-110??3?10---

01?0??10?0-01?11??-00??20??10?-?0?0????{0 1}????????2??????112-
??????????????111???????????????????1110121?2?111?100002??10-1-1?0?1100-2100000?-
1???????0?00??????1??0???1010???0???0?-??????????????1?10?0??????010????1?0??00??1??0????0??-
?????????????????0???0?0???01000???1???0?0?00??2???????????????????????????????????????010????1?1?
??{2 3 4 5}0?010?11??10001?1?1???0?0??1???0??????2???1000100?0111?-000??00?????010???110?100?01??-
????????00??0-?1?????0

Chaoyangia

??
???????????????????????0{4 5 6}1??????????????1????????????????????????????????????021{1 2}??0????011--
21210212121???01??0???0???????1{1 2
3}??????????????????????????020?????????0?????01101?????????????????????1{0 1}???????????0??0?0?--
?????????2?????????????????????????????????????11???????????????????????1-
1?????????1?????????????????????????????????????0?000?0?0?????????????????????????????????0????00?
?0??1??00???1???????????0???????????????????????????0??????0?0?????????????????????????????????????
????{0 1 2}0110?0??1110-
?10?0?0??01???1?

Changmaornis

??
?????????????????????161???{0 1}?????????????1?--212{1
2}1{1 2 3}1{2 3}0?1??????????????????1?1{2 3}0?{0 1 2}?02000{0
1}???????????????????100???????????????????01?20?0?00?????????????????????????0????0??0??0--
2???{1 2}?????????????????????????-????1?01?{2
3}?0??0??0?????????
???????????????????????????????????0?0?????????????????0??11
???-??????1?????01???????1??1{0
1}{0 1}{0 1}??????????????????0???????

guozhangi ???????0--??-?0??-1??0??111???????-???????????-?0?-
110?10001??????????????????????2??000????????????-?1?10?1?1-2(1
2)1000041010?0???????10????0?101021121000?1-?11?0??-{1 2}??0-0?02?210001-2110--21{0 1}2?213-
02???00?01101000?111113?10002000 1???????-0110????1300???010111-???0??01?1001?00????-?0-
102??010?-10?000??0??-112--0???3???12?1??????????-??1??{0 1 2}???00?????00??-0??2?1??11-2111?-
1?001??--??1-1?011001?2201100???0?-??110-0??--??010????{1 2}??????0?-000?-?1????1000001??{1
2}01?0?101?-0012????0??0(0 1)??1?0000?01?0??-0????0?1????0010??0?00??0100?????????1--00-
?1????1????011?????0-0-01111??????1(0 1)2020000000?????0??????2000?1001-
01010101?0???00001100012 10?10?00??0?1110000010000 10??01101?01011000(0 1)010?0??11?0???????0010-
?00

Changzuiornis 100????0--??????????????????2100?01-?????0??202-???-??-
?????????????????00(0 1)1001-??0???00021{1 2}--011????010???0?2?1????04100?2??---3?-111?31?001?02-
12100001?111103(0 1)00-(0 1)100-0222?1?2??{1 2}02?--20321212?2112-010011???????11113001?0200011031?-1-
??10??0-01(0 1)0?-?10103010---(0 1)1101?(1 2)000-0(0 1)?1100-00??{0 1}10?100-00?0?0?00(0 1)001?0--
2110311112-??0???{0 1}0??0-?-111(0 1)0??00210010??0-?00211?121-21111?1000020(0 1)(0 1)(0 1)-1-1?0?11?0-
20010000-00?-???1?-00?--1?010?1??11(0 1)01-?00?00??-???0?00???????101?1000????0(0 1)010{0 1}{1
2}??0000???1?100???01?-?-0???00??????0?0???1?0???11(0
1)00??00?010??010?0210001????????10??1?????1???00----102?{1 2
3}011????101?????2010210?0000??11010?10000-10000?0001021{0 1}-1010000{0 1}0???0-000010011???01{1
2}01?010{0 1}10(0 1)?11?0-???0??0100-10-00?00110

Yanornis_martini

?00????0????????????????????11?0?0???????0??20??????????????????????????{0 1}0110?1-
???????000?11???21???1?1????0?2???????4????2??---3?-111?{1 2 3}???01?02-120000???111?03(0 1)00-1100-
?????????????0?--2?321{2 3}?????1?2-010??1????????????{2 3}??{0 1 2}?0200011?3??-1???????0??????-
??0??3?00---0??01?20?0-0(0 1)?????-00????0??{0 1}0-?0?0????0(0 1)?0??0--?1103?01?2-
??0???000?0???11?10??00??0?????0-?002????21?2?111??00002?001-?-1?0?11?0-2?00?00?-01?-?????-
0???????????????1????0???????-???0????????????1?0???????????10??????00????????00?????1?-
??0???0?????????0??0??????111?0?????0????000??2??001????????1??????????????????????{0
2}?????????10?????????0???0??0?0??11010??010???0??0??00????{0 1}?1010??0{0 1}0????-
?0??????1????????1?110?????11??-??????????????-???0????

Yixianornis 1??????0???????????????1?????{0 1}10???1??1????1????-
?0???????0?0??1????????0011011????????0??2?1--0????01????????(0 2)01????04{0 1}00?2??---3?-
?11?310000002-1210000??111003000-0100-010??1???-2022100?{2 3}?0213121?2-010?11????????111300{0 1
2}0020001????????????????0-020?0-?10103?00---1??0111011-01?1100-00??110?1{0 1}1-
?0?0????00?01?010?????1?1??-??0????????0?--?111???0?21???0???????{1 2}11?121-11111?100?02011(0 1)-0-
1?0?1100-3?0{0 1}?100-00?????1??0???????1???1????100-????0?0?-?????????0?????101?100??0??1101{0 1}?{1
2}??00?0?????101??????-???????0?11?????0??????????111?00??0??-
????00?0210001????01??1?????????1?????---?1000301100??101??{1 2}{1 2 3 4
5}?2011210001010?11010??010???0??0??000121{0 1}?112010010?110-??01??0?101?????11010{0 1}??0?11??-
???1??0000-?0-???0??0?

Songlingornis ????????????????????????????{0
1}??01?????????????0?0?{0
1}1??021?????????????????????0??????????????????{1 2
3}100???02??????????????0???11
3????????????0?????????10????????????????????????????0??????0???0-
?????????1?????????01???1???????0??????????????110????????1?????????0????1????????1??0????????????????
???-
?????????????0???????????1??????????????????????????0??1??1????
????0???????????????01???20?1?10??1?1?????????????????
?????????????????????0???00-?0?????????

Tianyuornis
?1?????0???????????????????0??0?????????????????00????????????????????{0 1}001101-
??0????0?0??2???????????????1???2?1????????????????????????1?31?101?02-121010?1?111002(0 1)01-0100-?{1
2}?{1 2}11??1??011??{0 2}0???3???21????1??11??1???101113??1?020?0?113???1???1???0??3?????101?3?00--
-0110??2011-10?11???00???10??{0 1}?-?0?0?1??0?0???0??211?3??112???0????0????????100???0?10010??0-
???{1 2}???120-?111101000120?01-2-1?0?11??-3100000??01???????-0??????10??1????0000????????-
???0??0???????101?10011?00010???2?00??0??????0?0?????????????0???????10???0??????1101???????0??
?10??210001?????????????????????0?----?00??0?1??????1?????0010210??1001-
1101000010111010101?00000??{0 1}-10??110?0111?-00????0??0?0{0 1}?0011010010?001?0???????0?00-
10????00??0
Parahongshanornis ??0????0???????????????????0??0?01??????0??{1 2}0?-
???????????????????????{0 1}?0200?-???????0?02{1 2 3}{1 2}--0?????????1????{0 2}???????{4 5 6}???0{1
2}??---3?-111?{1 2 3}1?2?1?02?121000?1?11100200{0 1}-1100-1{1 2}1????????01?--2?2?1{1 2
3}12?21????1???1???01?101113011?020001113???0???1???{0 1}-0??0?-1101?3?10---011?0?10?1-?0?1100-
00???10??{0 1}0-?0?0?????10???0--211?3-?1???1???????????-11100??0?2100?0??0-0??{1 2}???121-
21111?1000120010-1?100?1110-3{1 2}00?00?-?1?0?????-00???1?10??1????0{1 2}?0????000?-
??????????????101?10011????001??{1 2}?????0?????00?0?????-??0?????????????0???0????011100??0???-
1??01?-?2{0 1}0?01????????1??????????????0---?1?0?{0 1}?11??0?{1 2}0???22?1010210?01001-
1100?0?0?012?0??(0 1)??00?????111{1 2}0??0{0 1}0????-??01??1?1?{0 1}?1?20?0010010101001?0-
???0???100?10-?0?0?000
Longicrusavis
??
?????????????2?????????????????????????1?31?1?1?02??210?0???101?02000-?1?0-{0 1}?1????????00?--20{2
3}{1 2}1{1 2 3}12021????1???1??0???????1300{0 1}?020001??????????????????????1101?3?00---??1?0?{1
2}0??-?1?1??0?00?????????-???0?1?????????0--2??????1?2??????????????????1???????????????????{1
2}???11?-2?11??1000220???-?-1?0?1110-3?00000???0????????0???????????????00?0????????-
??????0????????1?{1 2}?10??1?00000????2???-
?0??1??????????????????????????????0???0?0????1101???????1???10??1????????????????????????????????
???0????????????1??12?1010?10??000???1?1?0?0?0??{0 1}?1?1?1?1???????-0100??0?00?0--
??????1??0????2???010???1{0 1}{0 1}{0 1}???????????00010????????0?
Iteravis 100????0??????????????????11001?1??1???0?????-
?0??????????????????????000(1 2)02(0 1)-?{0 1}1?????1-0211--0{0 1}10?-1????1???????????{4 5}{0 1}01?2??---
31-111?{2 3}10011?02-1211?0???111003(0 1)01-110(0 1)-{0 1}12?11??1??002--2?2{1
2}1212?21???0????1??1???1011130010020000?103-?-2??111??0-0{1 2}???-110??3010---01101?20?0-00?1100-
000?110?100-?0?0?1??{0 1}(0 1)0?1?0--211?3-{1 2}112-??0?0??0??-?--11100??00210??0??0-0-1{1
2}??112??2?111?100?(0 1)2(0 1)0(0 1)1-2-1?0?110(0 1)-310{0 1}000?-00?-???1?-00??????0??1???10{1 2}1-
?0000?1?-???0?10???????101??0????00?10?0?{1 2}1?00?10??1?00?0??0??-??0--??0????????1???0????0(0
1)1100??0????????00-?210001????????11??????0????00----10?0{2 3}011?1?0101??2302010210??1(0
1)00?11?1??00?0101?1001???0121?1111010010?110-0?0???01????1100??11001010{0 1}{0 1}??-???1???000-10-
000000(0 1)0
Gansus
1??????0???
??-??????1???{0 2}01?0??05{0 1}{0 1}002??---31-?11-310001002-120100?10111003100-1100-(1 2)12211021-2022--
202{1 2}121{2 3}12112-010??1?11???(0 1)01113011002000????????????????02(0 1)00-110103?00---011011(1
2)01(0 1)-00?11?0-00??{0 1}1??10?-?0?0?1??0??0000--2??????112-
???????????????11???????????????????111111-21111010000(1 2)1110-2-10011110-3201000?-?0?????(0
1)0?00?????11??1?1?1011-???0000?-???????1??0??1101?1001?000?(0 1)010{0 1}{1 2}1000?0??1??0010?????-
???????0???????00???0?0???111000??0???0?1010??2??????????????????????????????????{0
2}0301100?0111??0402000210001011-11?1000010121010001000021?21110110101110-
0101100110111121110101101011110-???10??000-?0-00000?10
Ambiortus
??
????1????0???2?0????????????????????1?1?{2 3}10101?02-12{0 1}??????11100?1?0?1{0
1}?0??1101?{1 2
3}?{0 1}???????????????????11???0?????????-
?0??1???????????????????{1 2}???111-?1??1???0?0{1
2}???0??????????????????????0??????1??0????????????????????????????????????0???????101?01????????0
??0???????????1??????????0???????{1 2
3}???????????????????????????????????2????1???????{1 2}???{1 2 3 4 5}?{1 2}00?2100?101??1101-
1?0001210???????????????0101??0??0??10????0????
Yumenornis
??
??????????????????????????????????????1?{1 2 3}10????02??21010???1?1003010-1110-
??1101???10---
??????????????????00?????????-
???????????????????????1???????????????????1????????????????????????11-2111??100?021110-
??????????-
???0??0??00????????0??????11??1??????1???????????????????????????01?100??????00?????????????????
?????????????????????????0??????????1??????????????0???2??
????????12?{1 2}???210?01?1???1?10??010??????0??00??2?{0
1}1?110??0??0????0????
Dingavis ?0?????0???????????????????01?0?????????0???0?-
??????????1?????????????{0 1}?01?01-??0??0?1-?2?2--1????-1????????2????????????{1 2}??---3?-
1??????0?1?02-?110?0????1100?00??0{0 1}0??????????????????????????????11???????1????????111{2
3}????01000?1?3-?-2???11??0-?{2 3}???-?101?3?20---???0??30?(0 1)-?1?11??-00???10???0-
???0?0???1???????{1 2}?1?3??1?????0?{0 1}010??-??-?1100??0?2100?0???-?-12{1 2}??121-{1

2}?11??101012111?-??1?0?11?0-3?000?00-?1?-???1?-0????????????1???{1 2}???1??00??-
???1??????????1?1??0?????0000??????????0????0????????-??0--
?????????????????????????1?????0???????0??02{0 1}0?01?????????1?????????????00----
?02?3??1????10???????000?10??10???11?????0?0????100?1?????1??10301?0?00?0?????????0???????20??01
0???1?{0 2}1??-??????0?01-10-????????
Juehuaornis ??0????0????????????????????0??0??????????0?00?{1
2}????????????1?????????????{0 1}??1000-??0???01-???????????-?????????2????????????02??---3?-
??1?????01?0???11000????(0 1)0003(0 1)01-(0 1)100-{0 1}?{1 2}?1?0????02?--2?321{1 2 3}???21?2-
010??1??????????1?{2 3}0???020?0?1?3-??2???1???0??????-???1?3?{0 1}0---0??01?2010-0??1???-00??{1
2}?0??0??00?0????0??????--2?1?3??1??-??0???????-???11?00????2100?0???-?-?2????11-2?111?1010021110-
?-1?0?1111-3?00?00?-01???????-0???????????????0???101?1???-
??????????????1??1?100?0????0010????00??0??1?0?????0??-??0--
?????????????????????????1??0????????????00??100?01??????????????????????0?----
??2?????????10??????1???????????????1?10??010??{0 1}0??1????????1{0 1}?0130??0{0 1}0????-
??0??????????1???101100??1?00??-????????0????-????0?1?
Piscivoravis
???????0?????????????????????0??0??????????0???????????????????????????????{0 1}?01????????????1-0??{1
2}???????-??????????????????6????{1 2}??---3?-??1?{1 2}1?0?1??2??110?0????11?02?0?-?10?-
???????????????????3?1{1 2 3}?{2 3}?21?????????1???????????1{1 2 3}????02000?1??-??2???1???0?0????-
?????3?{1 2}0---???0??{2 3}0?0-???110?-00????0????-???0????0????????????????1?????????????-
????110????????0??0?????-?2?????1?{1 2}?111??0??0211??-??1?0?11??-3?00??0?-
??????????0???????????????????????????-???????????????1?1??0??00???10???????????????????????-
???--????????????????????????1??0????????????????1????1??????????????????????????????????3???????{1
2}01??????0???10?????????1???????0????????????????{0
1}?11????0???????????????0?????????????1????1?????-??????????-??-??????0?
Mystiornis
??
??
???????????????????????1301000{2
3}0??0??1?????????????????????????0??????????????2??????
??111???3??????????????????
??
????????0????????000??
???00?0?{1
2}1101????????????????0???????
Ichthyornis ?10----0--?--10?1-1????2?111100101-011?0??12?{1 2}-?0?-01--
10101?1??????1?0001000011010001-0211--1100--?11011111001?0?11(5 6)1000201---3?-????310001002-
11111001?11100{2 3}?-?-?0-?-02{0 2}{1 2}???21-2001{1 2}001?203????212-010?11?11???(0 1)0111301100{2
3}0???113---2-01100?0-?2?0(0 1 2)-1?0103000---?11?11?0??????????-00-020001{0 1}0-001001?00(0 1)0-??0102-
1?210??2-?-0-212?10--0-?1100--00210000-?100-0221??10-?1111?1000020?-?-{1 2}0110111??-31000?00-01?-
??11000?0--011(0 1)0011101?2?0?00?0100-???00(0 1)00000??1111111011000?0?1?{0 1}{1
2}1100??0111000????11?---0---00?1??-10?0?10??10-1?1??00-0?-?-001?1000220001????????10{0 1}1112101101{0
1}0---?110??0111?102?1102300001210001110111010010101110100100000211211301101111?-010(0
1)1?11100?1100110?1{1 2}11?11100-?1?100-000-?0-0000-??0
Gargantuavis
??
???????0111?-2???????51000??{0 1}??2????1-
2??????????????????21010??0????????????????11??????????
???????????????1?????????????????????????????-
???????????????1??????????????????????2???1??????
???????????????????0?????????????0??????1????????????????1?1200????1111???????1??????????0???????1
?????2(0
1)?????????????????1??2???????????????????????????????????
?????????????????????????????????0??????01??
Potamornis
???0?10?????????????????????????????????????
??
??0??
??
??
????????????????????????????????1??1100110?????
??
???????????????????????????????????
Enaliornis ???????????--01?1-10???2011??????????????????????????10--
?1??????00???????????????????????????????-??????1??-22??0???{2 3 4 5 6 7}1{0
1}?0??1?????????????3?????12-
010011?11???10111201100 2???1??????????????????2???????????1???????1??0??0???????????????????0????
?0??????????????????????1?-
?????????????????????????????????????1???????????????????????????0?111???31?????????1???????????
??????????????????0?0101-00????0??????1??????????????????{1 2}2??????001110??????0???-
??????0??1?????????01???????000???????1???0??2???1??????????
??0?????1?????1100111(0 1)011??01???1(0 1)1-
1?01?????????????000????0
Chupkaornis
??
?????????11??221?0{0

1}10??01????
??1???
??????????????????????????????1?????????????????????2??
??????????1???????????????????????????????????????00000???
???1???21?011???????
??10?0?????????????????????????
??????????????????
Pasquiaornis
??0?????????????????????????????2??--(0
1)?????????????-221?0{0 1}?????????????????????????????2???????????????????????????????????1???????{0
2}0???3?????12-0??111????????1112?01??????????????????????-?3?????????{2
3}1???????1??????????????????????????{0 1}{0 1}-?????(0 1)???0???????0???????12-
???????????????????????????????????1??????11?????????????????????0?11???32010??????????????????
??????????????????0?????-?????????????????????1???1??????{1
2}100???1111???????1??????????0????????????0?0??????0?0?0?????1???0??????????????????????????1??
?????????2??011?????????????????1?????????????????????0(0 1)0(0 1)1(0
1)1???211??????????111??0?0????11?0?1???????011??1?1?1????????????????0???????
Baptornis
?????????????????????????????00????????????????????????????0???????????????????{0 1}????01-
????????????1??????1-221?????510002?0---3??????????1?002-
0????101?????????????????????????1??????????????????12-0???11??1000(0 1)01112001?020??11?3-
????????????03?0-0???10310??????????1?000??{0 1}?????-??10?????{0 1}?-?0?0?0????????0??2?????????-
1?????????????111{0 1}????????????????-?2????21-?111?????????????????1?0111???3?0000??-
?1????100???????111001?001?????0??0?0?-??????(0 1)??????1??????00?0-0??????????????1??1?01???01?0-
???--????1??????0???0????????000???????1-?0???0{1 2}?????????????????11001?01???????0213?1?100(0
1)2???????????21?000?0011?0???????????00111000011??????????111??110????11???11{1
2}0111?01110111-0-???11????????-0000???0
Bmongoliensis
??
??
???????????????????????1{2 3}??{1
2}00?0??1????????????????????????????????????0??0???
???111???{2
3}???
??
??0111?1?{
0 1}?????????????????????????
baileyi
??
??
???????????????????????2??{0 1
2}?0?0??0??2???
??11????2???????????????
??
???????????0??????????0???
??11?1{0 1}1-
????????????????????????
varneri
??
??-????0?1??-221?00??1??00{1 2}--20{0
1}??3???2????????11??1???10111{2
3}001?0?0??1?????????????????????????????0???????1??0???1????????????????????????????0????????????--
?????????2???2?????????????????????????-
?0??10???2???????????????0????????????????????????????011?-?????????????????????????????????100-
?0????1010?0???????????????????????????0????????000???????1???0?????????????????????????????????????
???2??0???11--
????0?011???1?20????0111?111??????????????????00?????0
Fumicollis
??
??-????0????-22100001???????????????11?{1 2}?0????0?-?2?0-???????????????????2{0 1}221?021-2011--20{0
1}2?3??-0212-010111011???00111200100{1 2
3}0??1??????????????????030????1??1?31?????011001??1??????????????????10?????0????0??-?0?--
2?????????2-??????????????11?????????????????????211????-?11???????????????1-
100010???220?????????????0???0?????????????????1?-00?010?-??????0(0 1)0(0 1)000???????0??????????{0
1}{1 2}100-
?01111001000?1???????????0???????0????0???????1?000??0?????1?0?1???????????????????????????????????
???213010?0??1???013?????2??????????1???????-0???????????????????????????111--
110?0001100111201111?0111?111-1???????????????011????0
Parahesperornis ??0????0???????????????????1100?????????010102--
1??11?????????????????????11???????????01-0??????????-10??0???-221?0{0 1}?0{5
6}???????????????11???????1?02????0??????????????????????{1 2}02??1??1??00?--??1?0313?2212-
0??111??1?????111200100{2 3}00?1103-?-2?-110??{0
1}?031????1?01?30?????01??01?001????1100????0??0??{0 1}{0 1}-0??0?0?????-1??--2?10??????2-??????20??-
??-?1100??00?00000?????-?211???????1???????????????1-10?111???3?0?0???????-
??1??????0????????????????1?-???011?-???0?????????0????????0?0-??????{0 1}?????-??110100100??1?????0--
?0??1???1?????00????0?11000??0?0??1?0?00??10?11?????????????01?001101??---??02?30110?????????{2 3

4}??????10??0??01?????????????0????1?0????????????????111--?1010??11?0111{1 2}???0?0111?111-
1??????????????0??0??10
Hesperornis -10----0--?--01?1-10-1?21111100101-01?-0000202--0?-11--
1010001100??0110102001-?{0 1}1?1101-02{2 3}1--(0 1)020--10100110-221000016100000?003--111-110-
0100111200--------------------102201021-2002--20120312-2212-01-111{0 1}11???00111301100{1 2}0??1113---2--
1?00?0-0210(0 1 2)01101?30-----0110012001-000???-----0--011000000000?1001-110--2110311-12-?-0-112010----
111000-00200000-11-0-0(1 2)01-020-101--------------1-1101111?-320001-0--1?-101(0 1)0--00--000----------10-00-0112-
0??00(0 1)(0 1)00000(0 1)11010110001-0---00{0 1}{1 2}100-?11101001000?1??---0---00011?-1?00?000?00-0011000-
(0 1)0-0-01-0-000-10011?01?022111??01100110000----10213010000(0 1)21-000(2 3)00110210000?10101-10010-00-
10-----10---------------111--110100011001112011111111111101-?101-0-100-10-0010-000
Xinghaiornis ?0?{1 2
3}???010??????????????????????0??11?????0??20??????????????????????????02?21????????1-1---------?-
????????????????{3 4 5}???????---3?-????{2 3}1?2?1?02?12{0 1}010????????{1 2}???-????-?2?????2??????--
20???{2 3}????????0????1{1 2}???????111{0 1}????0{1 2}000?113-??2???????0-?????-?1?1?3?1?---0?1??????1?-
0{0 1}?11??-??????0?1?2-?0?????-?--????--?11-3--??????0?0?00??-????1100????2?0?10?????--
2???121?2111???0??21????-1-1?0???00-{2 3}{0 1}00?000-?1????????00??????1??????????????????-????--
???????10?1?100?0????101?????????????????????-?-?00--???-??????????????????1??{0 1}??-
??1??????1???2{0
1}???1???01????1???0?0?10??1?0???1?100?0?012?0?????01??2?{0
1}?01?????1{0 1}0111????0??????0??????101100??0?{0 1}?-?-????????0???0-???0????
Horezmavis
??
??
????????????????????????1{2
3}??10???0???2
???111???3????????????
??
??
???0{1 2
3}11??????????????????????????????
Maaqwi
??
??02-
??
???????????0????0??????????????????????????????????-
???1?????????????????????????????11????????????????????
????????????????????????????????0???
???{1 2
3}??10001?10?1?????????????????????????{0
1}???0????
Jiuquanornis
??
???????????????????????????????????????1?{1 2
3}100???
????????????????????11???
?????????????????????????????????????1???
???????????0????????????????????????1???
???????1??????????0???13?1000????????????
???110-10?????????
Eogranivora
??0?????????????????????????0???0???????????????1-
1??????????-???????????????????????2??---3?-??1?{2 3}1?{0 1}?1?02??20100???????02(0 1)01-1100-
021??1?????00?--{1 2}?{0 1}?1{2 3}??????????1???1????????1112??0?1--001113-?-????????{0 1}?0???0-
11???3?00---011?1?30?0--0?110?-00??{1 2}??????-???0????{0 1}???1?0--??????-11?????????0??-
????1110??00?101???????-?{1 2}???1?????111??00?021010-{1 2}-1?1?11---2101?-0?-0??-
??????0????0?????1????020?????????-??????????????1?10?00??????101?{1 2}{1 2}???0??????????0???-?-
???--??0?????????????0?0????1?02???????0???1?-?2?1?01????????????????????????????????????1?????1{1
2}?1???{3 4 5}01000?10??1?1?????1???000??????1????????{0 1}21030??0{0 1}0?1?0-
???1??????????????010???--11??-???0????0?-00-????0???
Qinornis
??
??
?????????????1??????001112??1?0{2 3}0001???1?10??{0
1}?????????????????????0??????????????{1
2}??01???3?0????????
?????????????????????????????????????-
???0?????????????????????????????????????0?{0
1}???
???121{1 2}0??0?0210??11-?????????????????0?????0
Iaceornis
??
???????????????????????????????????????{2 3}?0?1?002-1????????11100{0 1 2 3}??-????-?122?11??1??01(0
2){0 1}0?012?31{2 3}?0?12-
01??11?1????0011????????????????????????????????????1010????????1101?????????????0?00??20??1{0 1}?-
?0??????0???1??10????????12-?????????????????1????????????????????????21-21??1?000021??-
?221??????????1???????01????????11???0?1?????????21??00?????-????????????????0???????????00?0?{1

2}1000?00??0????0??1??????????0???????01??????????1???????????????0???????????????????????????
?????????????????????1?02{3 4}1??1?21000?1101111??????????????????????11130110101110-
01?????1100121001 1??????????????????????0??????0?0??00
Palintropus
???
??-???????????21?0?????{0 1}?0?????????????????0?002-
1??2-
01001???3???????10?0?????????????????????????????????0?-
?0?????????????????????????-??????????????????????????????????????1??21-
?1????????????????????????????????????1???????????????1??????????????0???????????????????1?????
????????????????????0?10???????1??????????????????1??
??????????????????????????011?????????????????20?0???1001???
??????111-?????????????????????????????????????0????
Apsaravis
???????0???0?????????????0002020-?{0 1}0??1?????-
????--???1???0????2?01?{0 1}??5100?200---3?-1???{2 3}?0?00002-?110?001?11100??????????1222??021-201?--
2112131311212-?11??1?0????01113001?1--??1???????????????1?0{1 2 3}?0(0 1)-??0103?1????0?1?1?101?--
1????--0???2???1{0 1}2-0??0?1??0??-??0--2???3??1?2-??????????????1111??????????????-0?-2????21-
211?1???0??2?????{1 2}-??0111--?32010-0?-?1?????10-?????111?101????010?-0??????-???0-
???????????1?01?0???001????1{1 2}?000?00?????????????-
????????????????0???0?0????1111????????????10??1?00?????????????????????????---???2030100101{0
1}?{1 2}??1??????2010?101001111-0?0101020111?101012?00?12011??01110-?001101110?0012011?10110--010?-
???10????????-0000??00
Lithornis 110----0---?-?00?-1010?2?110100101-011-1000202--00-01001000(0 1)00111--01-0(0
1)021110?1021001-1---------?-111001?0-221??2?161000100---3--111-300011002-120000?1?111003011-110?-(0
1)21211021-2011--21220213-2212-0110011?1???01111300100200 0?113---2--111000-02100-110103010---
0110112011-10?110--00-01000102-000001?-0---100--2--01--112-?-0-01200?----111100-002101(0 1)0-1010--211-111-
21111?100?02101?-1-1?011110-32010000-00?--01101000--0?(0 1)(0 1)??1?1?10210-0?00?0{1 2}-01-0--0??00(0
1)0110101(0 1)01?001?1000?21000?00?11?0000??0-?-?-0---00011?-10010000201-1110?10--1-0-01-
0100012111100000111110101100110?111?0-10203010001111111231001 1210(0 1)011101111100(0 1)11012(0
1)1101(0 1)00(0 1)012111113011010 1110-01001011100011101101121101 0111-01?1??-?01-?0-01000000
Palaeotis ??????????-?-????-10?0???????0?????????1?????????????????0????????--01-
0?????????0???1???1???1111101????0???11100???????
???112?100?1-2??????????3???2??????0?????1?????1??3011?1--
0?1?????????????????????????0002?0????010???0??0?-{0
1}?11???0???00??1??1?0?0????0???00????????????1???????????????????11???????????????????2????1???01
1101?0????????1?1?0?11--?3{1 2}0??-1???0??-0?1???0?????????????????0??0??0????0?-
?????????????????0???????????0???0????00??????????????????????0?1??????0???0?0?????001?????????????
??1??1???????1{0 1}?1???0?{0 1}??????2??????0?????????1????????????-?0??01-
??0?0????????????0??0????????{0 1}???3011??????0?1???1???1???1????????1{1 2 3}??--
00???????????????????0??0????
Limenavis
???
???{0 1}?1?11100{0 1 2
3}??
???{0
1}????????????????????????00??20????????????1???
?????????????????1?1??0???{1 2}????????????????????20????0??????????????????10?1?0??0{1
2}????????????????????????????????????1??11???
???????????????1??
????101010101 11??1?3011010??0?????????????????
Struthio 010----0-----1001-1010121110110101-111-1010202--01-00001010100110--11-
0001020--1020001-1-----------10101110-2210000(0 1)71000100---3--111-110-111101120000?101110030-1-10-0-(0
1)0(0 1)21(0 1)021-20(1 2)2--21120(2 3)0201102-010111{1 2}?100000111301101----2103---2--111100-03102-1-
1003100---011001--00--0?110--00?021001021000-0?0-0--010---2--03--112-1-0-2120??----111200-00210110-10-0--
201--1110010101000 0{1 2}1?-0-1-1?0111---32010-10-001--0110-010--0?0???1?0?10{1 2}10000?0100-00-1--(0 1)1(0
1)10(0 1)(0 1)1-01?0000?1??0100112100-010?11?0000?01-0---0---00011?-11100-10110-0-11000--1----1-
10000021111000001011101011001111 10110-11213010102121-111400----011001-000101-101(0 1)00-
10001000100101?103001000111--01100111110?11?011011211--0000-010-10--------0000-000
Tingmiatornis
???
??10??0?1???????????????????????????????????????
???
???????????1???11?????????????
??????????????10??????????????????????01???????????????????????????????????1010011001?????????
???
???????????????????????????????????????100?1??120?001111000 21???????????????????????????????????
??????????????????1?????????????????
Chauna 110----0---?-????-10?1??????100101-01?-1000102--1?-01--1110100??0--11-
10120{0 1}1-?{0 1}?21101-1---------?-1??00??1?221??2??7???02??---3--101-31?001002-0200?0?10111003-0?-120?-
222211 0?1-2011--21121212-2212-010111??1???0011130?1?02000?113---2--1110?{0 1}-020?1-1?01?3010---
01101?10?1-0{0 1}?110--10?01001102-1010???-0---100--2??03--1?2-?-0-?12?-?----11110?-00210110-11-0--2111110-
211110100?021?0?-2-1?0?1111-3?0??00-00?--011?-?00--0?0?0?111010{1 2}10-0000???-00-1--?1?0000110{1
2}?10?1?????10?1?{1 2}?????10??1???00??1-?-?-0---00000?-10?0000????-??10??0--1---0??-100?321011{1
2}111022110111121111010011111121301010201111115 0(0 1)01121001011(0

1)1110100110?1201000101101111211301001011111-?1111111100?21201101121102{0 1}{0 1}11-1??1?1-?01-11-
?1100110
Polarornis ?000?-?0???-
?0??1??????2?11?1?0?0???1???01??0???????????1000???????????????????????????1????????????????110
-2???????????????????????1??12-
01?11???1???????????????????1?3-??1????1??????????1?????0????????????????????????????????{0
1}??????????????????????0??-???-?-0-?0?????????1??00?-?02?0?1??1???-
?2??????????????????????????1??????????0????0????????1????0??????????????????00?00?0?0?????1???
?????????????????????????????0??0?00??1?1-??-?0???1?0????1?????0????-??1????-
???0??1????0??2??112??1????10???121?110?????????2???????????????{2 3 4
5}???1000???????????????????11????
?????????????1??
Vegavis
???1???????????0??100?????????
?????0?00??0?22????107??????????????????1??11002-12101????111003?-?-?0-?-2{0 1}2??????-2?1???{0
2}1{0 1}??3???1?12-0101110?1???0?1113??1???????????????????????01??????0103010---
011?1??????????????00?1{1 2}1???1?-00???1???????????????????12-
??????????????1?1????????????????0??2????20-111?1?100?021?-?-2?100?????-{2 3}{1
2}02??0???1????11??00?????1?????1???21??00?000?????????????001?01?110??????1????{1
2}??0???001010000??0????????????0??????0?????0????1??????1????????0??1??????1????21????????????
0????????2??0111020????????1???210000?10111?10011101000?001011????1211{1
2}01?0101111??1???0111?0?2?0???0?121?????????????????0???0????0-???
Anatalavis ?0?????0????-?1??-10?????????101?1?1??-
1???????????01???????1001???1???????????{0 1}???101-1??????????-
????????0?????0????????????????????3?0011001-1?{0 1}0????1111003???-?1??-
????????1??0?1??2???3-??0??11????????????1010???0---
???????????????????0???11?????-1????1?????????????????-??????????????-?-???1??????2???10?1???-
?????120-211?1??00?021???-??????????-
???10?00?00?0??1???0??????0???????????????0?????00????????????011?100110?0?10????10???????????
????-?????---?0?00?-???1???????????1?????????-00???????{1
2}??11?1????211???11?????????????11?????1??????{1 2}1?{1
2}???0102110101100011?10?0?1?120?0011101??????113011011?1??-
0??????????????????????????????0??????100-?0????0????
Presbyornis ?00----0--?--?1?1-1??{0 1}?2?11?110101-???-10??102-11?-0?--
1110110????????01000??-?{0 1}??1101-1---------?-???????????1??0??61100?????????????310(0 1)11?02-
120000???11100??????????122?1???1??02?--211{1 2}1213?0?12-0101?1?1????00111301100{2 3}000?103---1-
0?00-?0-?2?0???10103110???01101?00?0??0????-?00??1101102-10-001?-0--?1?0--2--03--?12-?-0-212?-?----
1?1000-002101?0-??-?--2?1?121-211110?001?2?????2-1?0111???32010?00??0?-?0???-?01--0?11??1?1???{1
2}10?00?00??-???1--???????1101?1(0 1)01?010?0??0?{1 2}?00??10011?00????1-??--0---000???-1?01???????-
??11000--?---
0???10??221011????12?110?1111111101??????112????1?02???1??24?1000210001110?11010?11101200100101
1??1???1130110101111-000??1?11?0021201?1?1211?111?0??101?1-100-10?0000?010
Teviornis
??
??111003???-????-
??---
???????????????????0???11???????????1???
????????????101001?2????-??????????-
????0????1??????????????????????????2??????????????????????????????????1?????1???????????????????
??
???????????????????????????????????????00??01???????1130110l????????????????????????????????????
????????????????????????
Anas 100----0---?-?10?-100012?1??110101-011-1000102-11?-01--1100110110--01-
1101100-10021101-1---------?-1010(0 1)1?0?2210?1??720?02?0---3--111-310010002-12000001?111003?00-00?0-
21121?021-2021210112?313-0212?010111??1???00111300100200011 13---1-01100?0-0{1 2}?00-1101?3110---
01101?10?0-00?1101-00?02101102-100001?-0---100--2--03--112-?-0-2120-0----11100?-00210?10-11-1--2{0 1}?-121-
21111?1001021?00-2-1?011110-31010000-?1?--0110-000--0?01??1?1??0{1 2}12-?0?001?-01-1--
0???0??1101?1001100??1000?2?00??10??1?0000??1-?-?-0---00010?-10?1001???1-?110000--(0 1)---
00?110011210?1111112211021112111101001111112130101020111111510000210001111-
1101001110121100111110111121130110101111-0111101110{0 1}?21?011?11211110110-11011?-110-10-0000?100
Gallinuloides ?10----0--???????????????????1?0??1-?1?-?00?002-?0?-????1?1?????????????{0
1}??2??0??????0?1-1??????????-1????????????????{4 5 6 7}1????2?0---3--111-310210002-?200?0??1111003??--
?2?--{0 1}1?2???????01?--2?121313-12?2-??01?1??1???1011130?1?02000{0 1}103---1-?1?0??0?0{1 2}?0?-
?101?3010---0?101?10?0-0{0 1}????--00????01???-?0?0?1??0??-??0--2--0??-1?2-?-0-2120-?--?-?1110?-00210100-
????-?2???111-21111?100?021??--?-100?11?0-3?010000-?0?-?0????1??--??10??1?????2??-??00?0?-
???1??????0???112?10?1???1?10??????0??10??1?00?0??0-?-?-0---0?0???-1??0??00???-?01001?-??--
1????100021?011?????????1???????1????????????11??????1?1??{1
2}11???21101020001111??11?10???00??1?10111????{0 1}?111(0 1)30110{0 1}01111-
?1?????1????2120??011???0?01??-???0?1-100110-00?0?010
Paraortygoides ???????0??-?????????????????10010??????0?1??????0????????????????--
11-1??????-???????1-1---------?-?????1???{0 2}{0 2}???{0 1}?0{5 6 7}1?002??---3--1???310210002-
120000??0111003??--?2?--?{1 2}{1 2}?1?0????????????{1 2 3}??{1 2 3}?????12-010??12???????111{2 3}?1{0 1
2}?010000???-?????1????{0 1}-03???-?101?3?20---0????11011-0{0 1}1110?-00??21????2-?0?0?0?-0--
???0???????3--1??-??????????-????111?????2????0?1?-?--211?121-21111010000210??-??110111?0-3?00000?-
?1??-??1?-?????1?10??1?????1????0?0???-??-?--0??????1101?1001?????101?????????0??0?0000??1-?-???--
????1??????1?????????110?00?-????0???1???1{1 2}100???????{1 2}?????????????????????????3011?1??{0

1}?1??1???0102100?1?1??11?10??000??0?000?011?0{0
1}?11113011010??????0?1???????12???1??1?2???11111-???1???00??0-1??0????
Sylviornis ?00----0---?-?10?-1000???1?1100101-011-1000--2-100-01001000110101--11-
00???????0???101-1-----------1000011102211000{6 7}?0????????????1???{1 2 3}?0?01002-1200001?11100{0 1 2
3}??????????????????1??001???1?????????02-010111??1???10111301100?00?{1 2}003---0-01000?1-
02?0????010110??????1?100000???11????00?011?0?02-100001?-?--???0??2??0?--???-?-1-?120-?----1?011?-
0?211100-0??0--(1 2){0 1}1??21-01111??00????????????00?11?0?2{1 2}010001?00?1-011????0--1?10101?1???{1
2}???00?000?-01-1--?1000011?0101001?1?1?1????{1 2}?0????1?11000????1-???-0---0?010?-1001001???0-
??10?10--0---?0??1?11121011-11-02{1 2}?1011012111101?0101?11213010112????1??2?????101?10111-
11?10?11?0121000000010001{0 1}?11301101?111?-???????11000212011?1311?10111?0???10-
???????0010?0?0
Meleagris 110----0-----10-1-10-2-20111100101-111-1010202-000-01001100110110--11-
0112020--0021101-1-----------101011?0-221000(0 1)171000200---3--111-310211002-1200001111003---------
112211021-2011--21{1 2}20313-0212-010111111???001113(0 1)11002000?113---1-0110000-02102-110103(0 1)10---
0110111011-(0 1)111100-00001001102-100001?-0---100--2--03--112-?-0-2120-1----111100-00210110-10-0--211-121-
1111101000(0 1)210---1-10011110-32010000-11?--0110-100--??100110101?2?1-?0?0101-01-1--(0
1)11100111110100 11??0?100012100?010011100000?1-?---0---0001??-10010000100-0011000--1---01-
1100011101121110211101101211110100110-11213010112001111121001011001(0 1)111-11011011100-
1100110110021111(0 1)30111201111-1111111111002(0 1)1011111311020110-010000-?10010-0000-010
Cerebavis ???????0???--?1?1-
11?10?????????????????????????0???1?????????0??1???
??
???????????????????????????11???
??0?1???
???????0???????????????????????????????2?01??
-
?0???????0???0??11?01?????????????????1??????????????
???10???
????????????????
Foro ?0?----
0?????????????????????0??1??1???0??10???????????????????????????10?????02?001-1??????????-
101????????????{0 1}??7???02??---3--?11-{1 2 3}10001?02-120010???111003??--?2?--?{1 2}?21?0?1???(0
1)?????{1 2 3}{1 2}1{2 3}13-22?2-01?????????????111{2 3}???002000?103-??1???1???1?1{2 3}1?(0 2)-11?102?20---
011???2011-0(0 1)1???--10?1200??0{0 2}-10?0?0??0??-?00???0000--1??-?-0-2?2?-?-???11110?-??2101?0???00-
?21?0?11-2?111?100?011??--??1?0?1110-3?00?000-?0???0???01?????1?0??1?1?102??-???0???????0-
??0??0??1111?1001000??100??2?0???0??????0?????-?-??0--?????????101???????-1011?00-?1--
????11??01?1001?????????1?{1
2}1??????????0?????????3??0?0?011?1124?100?210??0????11110?0010??00?011?01??01101130110{0
1}01????01?0????0???2??????103??0111?0-???1????01-?0-?0?0?000
Columba 100----0-----?1???00?{0 1}???1??100101-11?-1010202100?-01-01111100???--11-
00010?0-?{0 1}?20001-1---------?-101001?10221???106???02??---3--111-310010002-120000111111003---------
212210021-2021--2(0 1)121213-2212-010111?11???10111311100{2 3}000?113--02-01100?1-021?(0 1 2)-010103010-
--0110101011-?21110--00?0??01102-100001?-0---100--2??03--112-?-0-?1?0-?---011110?-10211100-1?-0--2201111-
21111?100?1200---1-11011110-3201000?-00?0-01(0 1)?-100--1?00001???0?{1 2}1--010000?-01-1--
0????01110111001101 0?1010020000?10?11110001?1-?-?-0---010100-10?00-00??0-0110010--?---0(0
1)11100?2110?121?1012110211110001 0??0????112?31?011?00111?1212010210001?11-
11010011101201001100000 01{0 1}?1030110111111-100111?11001202011111301121{0 1}?0-0??00?-110-10-0010-
010

;

END;
BEGIN ASSUMPTIONS;
OPTIONS DEFTYPE = UNORD POLYTCOUNT = MINSTEPS;
TYPESET * UNTITLED = unord: 1- 3 5- 21 23 27 29- 43 45 47- 70 72- 73 75 77- 79 81 83- 88 92- 94 97- 113 115- 116 118 120- 121 123- 125 129 131- 134 136- 137 139- 142 144- 145 148- 149 151- 157 160 164 166- 168 173- 175 177- 178 184 186-189\3 193-196\3 198 200- 204 206- 215 217- 218 220- 221 223- 225 227- 228 230- 232 234- 240 242- 243 245- 246 249- 253 255 259- 266 268- 271 274- 283 285- 289 291- 299 301- 305 307- 308 310- 312 314 316- 317 320- 323 325 327- 336 338- 339 341- 342 344- 355 358- 360 362- 363 365- 373 376- 379 383- 384 386- 391 394 396- 413 415- 424 426- 427 429 431- 437 439- 455 457- 469 473- 490 492- 495 497- 514 516- 523 525- 539 542- 545 547- 551 553- 555 557- 559 561- 566 568- 576 578- 582 584 586 588- 589 592 594- 596 598- 617 620- 629 631- 632 634- 635 637- 640 642- 657 659 661 663 665- 668 670- 672 676- 700, ord: 4 22 24- 26 28 44 46 71-74\3 76 80 82 89- 91 95- 96 114-117\3 119-122\3 126- 128 130 135-138\3 143-146\3 147-150\3 158- 159 161- 163 165 169- 172 176-179\3 180- 183 185 187- 188 190- 192 194- 195 197 199 205 216-222\3 226-229\3 233 241-247\3 248 254 256- 258 267 272- 273 284 290 300 306-309\3 313 315-318\3 319 324 326 337-343\3 356- 357 361-364\3 374- 375 380- 382 385 392- 393 395 414 425-428\3 430 438 456 470- 472 491 496 515 524 540- 541 546 552 556 560 567 577 583 585 587-590\3 591 593 597 618- 619 630-636\3 641 658 660 662 664 669 673- 675;
TYPESET default = unord: 1- 3 5- 21 23 27 29- 43 45 47- 70 72- 73 75 77- 79 81 83- 88 92- 94 97- 113 115- 116 118 120- 121 123- 125 129 131- 134 136- 137 139- 142 144- 145 148- 149 151- 157 160 164 166- 168 173- 175 177- 178 184 186-189\3 193-196\3 198 200- 204 206- 215 217- 218 220- 221 223- 225 227- 228 230- 232 234- 240 242- 243 245- 246 249- 253 255 259- 266 268- 271 274- 283 285- 289 291- 299 301- 305 307- 308 310- 312 314 316- 317 320- 323 325 327- 336 338- 339 341- 342 344- 355 358- 360 362- 363 365- 373 376- 379 383- 384 386- 391 394 396- 413 415- 424 426- 427 429 431- 437 439- 455 457- 469 473- 490 492- 495 497- 514 516- 523 525- 539 542- 545 547- 551 553- 555 557- 559 561- 566 568- 576 578- 582 584 586 588- 589 592 594- 596 598- 617 620- 629 631- 632 634- 635 637- 640 642- 657 659 661 663 665- 668 670- 672 676- 700, ord: 4 22 24- 26 28 44 46 71-74\3 76 80 82 89- 91 95- 96 114-117\3 119-122\3 126- 128 130 135-138\3 143-146\3 147-150\3 158- 159 161- 163 165 169- 172 176-179\3 180-

```
183 185 187- 188 190- 192 194- 195 197 199 205 216-222\3 226-229\3 233 241-247\3 248 254 256- 258 267 272- 273
284 290 300 306-309\3 313 315-318\3 319 324 326 337-343\3 356- 357 361-364\3 374- 375 380- 382 385 392- 393
395 414 425-428\3 430 438 456 470- 472 491 496 515 524 540- 541 546 552 556 560 567 577 583 585 587-590\3 591
593 597 618- 619 630-636\3 641 658 660 662 664 669 673- 675;

END;

BEGIN PAUP;
	pset msTaxa = variable opt = deltran;

END;
BEGIN MESQUITECHARMODELS;
	ProbModelSet * UNTITLED   =  'Mk1 (est.)':  1- 700;
END;

BEGIN NOTES;

	SU  C = 219 N = color I = 5;
	SU  T = 1 C = 219 N = color I = 5;
	SU  T = 2 C = 219 N = color I = 5;
	SU  T = 3 C = 219 N = color I = 5;
	SU  T = 4 C = 219 N = color I = 5;
	SU  T = 5 C = 219 N = color I = 5;
	SU  T = 6 C = 219 N = color I = 5;
	SU  T = 7 C = 219 N = color I = 5;
	SU  T = 8 C = 219 N = color I = 5;
	SU  T = 9 C = 219 N = color I = 5;
	SU  T = 10 C = 219 N = color I = 5;
	SU  T = 11 C = 219 N = color I = 5;
	SU  T = 12 C = 219 N = color I = 5;
	SU  T = 13 C = 219 N = color I = 5;
	SU  T = 14 C = 219 N = color I = 5;
	SU  T = 15 C = 219 N = color I = 5;
	SU  T = 16 C = 219 N = color I = 5;
	SU  T = 17 C = 219 N = color I = 5;
	SU  T = 18 C = 219 N = color I = 5;
	SU  T = 19 C = 219 N = color I = 5;
	SU  T = 20 C = 219 N = color I = 5;
	SU  T = 21 C = 219 N = color I = 5;
	SU  T = 22 C = 219 N = color I = 5;
	SU  T = 23 C = 219 N = color I = 5;
	SU  T = 24 C = 219 N = color I = 5;
	SU  T = 25 C = 219 N = color I = 5;
	SU  T = 26 C = 219 N = color I = 5;
	SU  T = 27 C = 219 N = color I = 5;
	SU  T = 28 C = 219 N = color I = 5;
	SU  T = 29 C = 219 N = color I = 5;
	SU  T = 30 C = 219 N = color I = 5;
	SU  T = 31 C = 219 N = color I = 5;
	SU  T = 32 C = 219 N = color I = 5;
	SU  T = 33 C = 219 N = color I = 5;
	SU  T = 34 C = 219 N = color I = 5;
	SU  T = 35 C = 219 N = color I = 5;
	SU  T = 36 C = 219 N = color I = 5;
	SU  T = 37 C = 219 N = color I = 5;
	SU  T = 38 C = 219 N = color I = 5;
	SU  T = 39 C = 219 N = color I = 5;
	SU  T = 40 C = 219 N = color I = 5;
	SU  T = 41 C = 219 N = color I = 5;
	SU  T = 42 C = 219 N = color I = 5;
	SU  T = 43 C = 219 N = color I = 5;
	SU  T = 44 C = 219 N = color I = 5;
	SU  T = 45 C = 219 N = color I = 5;
	SU  T = 46 C = 219 N = color I = 5;
	SU  T = 47 C = 219 N = color I = 5;
	SU  T = 48 C = 219 N = color I = 5;
	SU  T = 49 C = 219 N = color I = 5;
	SU  T = 50 C = 219 N = color I = 5;
	SU  T = 51 C = 219 N = color I = 5;
	SU  T = 52 C = 219 N = color I = 5;
	SU  T = 53 C = 219 N = color I = 5;
	SU  T = 54 C = 219 N = color I = 5;
	SU  T = 55 C = 219 N = color I = 5;
	SU  T = 56 C = 219 N = color I = 5;
	SU  T = 57 C = 219 N = color I = 5;
	SU  T = 58 C = 219 N = color I = 5;
```

SU T = 59 C = 219 N = color I = 5;
SU T = 60 C = 219 N = color I = 5;
SU T = 61 C = 219 N = color I = 5;
SU T = 62 C = 219 N = color I = 5;
SU T = 63 C = 219 N = color I = 5;
SU T = 64 C = 219 N = color I = 5;
SU T = 65 C = 219 N = color I = 5;
SU T = 66 C = 219 N = color I = 5;
SU T = 67 C = 219 N = color I = 5;
SU T = 68 C = 219 N = color I = 5;
SU T = 69 C = 219 N = color I = 5;
SU T = 70 C = 219 N = color I = 5;
SU T = 71 C = 219 N = color I = 5;
SU T = 72 C = 219 N = color I = 5;
SU T = 73 C = 219 N = color I = 5;
SU T = 74 C = 219 N = color I = 5;
SU T = 75 C = 219 N = color I = 5;
SU T = 76 C = 219 N = color I = 5;
SU T = 77 C = 219 N = color I = 5;
SU T = 78 C = 219 N = color I = 5;
SU T = 79 C = 219 N = color I = 5;
SU T = 80 C = 219 N = color I = 5;
SU T = 81 C = 219 N = color I = 5;
SU T = 82 C = 219 N = color I = 5;
SU T = 83 C = 219 N = color I = 5;
SU T = 84 C = 219 N = color I = 5;
SU T = 85 C = 219 N = color I = 5;
SU T = 86 C = 219 N = color I = 5;
SU T = 87 C = 219 N = color I = 5;
SU T = 88 C = 219 N = color I = 5;
SU T = 89 C = 219 N = color I = 5;
SU T = 90 C = 219 N = color I = 5;
SU T = 91 C = 219 N = color I = 5;
SU T = 92 C = 219 N = color I = 5;
SU T = 93 C = 219 N = color I = 5;
SU T = 94 C = 219 N = color I = 5;
SU T = 95 C = 219 N = color I = 5;
SU T = 96 C = 219 N = color I = 5;
SU T = 97 C = 219 N = color I = 5;
SU T = 98 C = 219 N = color I = 5;
SU T = 99 C = 219 N = color I = 5;
SU T = 100 C = 219 N = color I = 5;
SU T = 101 C = 219 N = color I = 5;
SU T = 102 C = 219 N = color I = 5;
SU T = 103 C = 219 N = color I = 5;
SU T = 104 C = 219 N = color I = 5;
SU T = 105 C = 219 N = color I = 5;
SU T = 106 C = 219 N = color I = 5;
SU T = 107 C = 219 N = color I = 5;
SU T = 108 C = 219 N = color I = 5;
SU T = 109 C = 219 N = color I = 5;
SU T = 110 C = 219 N = color I = 5;
SU T = 111 C = 219 N = color I = 5;
SU T = 112 C = 219 N = color I = 5;
SU T = 113 C = 219 N = color I = 5;
SU T = 114 C = 219 N = color I = 5;
SU T = 115 C = 219 N = color I = 5;
SU T = 116 C = 219 N = color I = 5;
SU T = 117 C = 219 N = color I = 5;
SU T = 118 C = 219 N = color I = 5;
SU T = 119 C = 219 N = color I = 5;
SU T = 120 C = 219 N = color I = 5;
SU T = 121 C = 219 N = color I = 5;
SU T = 122 C = 219 N = color I = 5;
SU T = 123 C = 219 N = color I = 5;
SU T = 124 C = 219 N = color I = 5;
SU T = 125 C = 219 N = color I = 5;
SU T = 126 C = 219 N = color I = 5;
SU T = 127 C = 219 N = color I = 5;
SU T = 128 C = 219 N = color I = 5;
SU T = 129 C = 219 N = color I = 5;
SU T = 130 C = 219 N = color I = 5;
SU T = 131 C = 219 N = color I = 5;
SU T = 132 C = 219 N = color I = 5;
SU T = 133 C = 219 N = color I = 5;
SU T = 134 C = 219 N = color I = 5;

SU T = 135 C = 219 N = color I = 5;
SU T = 136 C = 219 N = color I = 5;
SU T = 137 C = 219 N = color I = 5;
SU T = 138 C = 219 N = color I = 5;
SU T = 139 C = 219 N = color I = 5;
SU T = 140 C = 219 N = color I = 5;
SU T = 141 C = 219 N = color I = 5;
SU T = 142 C = 219 N = color I = 5;
SU T = 143 C = 219 N = color I = 5;
SU T = 144 C = 219 N = color I = 5;
SU T = 145 C = 219 N = color I = 5;
SU T = 146 C = 219 N = color I = 5;
SU T = 147 C = 219 N = color I = 5;
SU T = 148 C = 219 N = color I = 5;
SU T = 149 C = 219 N = color I = 5;
SU T = 150 C = 219 N = color I = 5;
SU T = 151 C = 219 N = color I = 5;
SU T = 152 C = 219 N = color I = 5;
SU T = 153 C = 219 N = color I = 5;
SU T = 154 C = 219 N = color I = 5;
SU T = 155 C = 219 N = color I = 5;
SU T = 156 C = 219 N = color I = 5;
SU T = 157 C = 219 N = color I = 5;
SU T = 158 C = 219 N = color I = 5;
SU T = 159 C = 219 N = color I = 5;
SU T = 160 C = 219 N = color I = 5;
SU T = 161 C = 219 N = color I = 5;
SU T = 162 C = 219 N = color I = 5;
SU T = 163 C = 219 N = color I = 5;
SU T = 164 C = 219 N = color I = 5;
SU T = 165 C = 219 N = color I = 5;
SU T = 166 C = 219 N = color I = 5;
SU T = 167 C = 219 N = color I = 5;
SU T = 168 C = 219 N = color I = 5;
SU T = 169 C = 219 N = color I = 5;
SU T = 170 C = 219 N = color I = 5;
SU T = 171 C = 219 N = color I = 5;
SU T = 172 C = 219 N = color I = 5;
SU T = 173 C = 219 N = color I = 5;
SU T = 174 C = 219 N = color I = 5;
SU T = 175 C = 219 N = color I = 5;
SU T = 176 C = 219 N = color I = 5;
SU T = 177 C = 219 N = color I = 5;
SU T = 178 C = 219 N = color I = 5;
SU T = 179 C = 219 N = color I = 5;
SU T = 180 C = 219 N = color I = 5;
SU T = 181 C = 219 N = color I = 5;
SU T = 182 C = 219 N = color I = 5;
SU T = 183 C = 219 N = color I = 5;
SU T = 184 C = 219 N = color I = 5;
SU T = 185 C = 219 N = color I = 5;
SU T = 186 C = 219 N = color I = 5;
SU T = 187 C = 219 N = color I = 5;
SU T = 188 C = 219 N = color I = 5;
SU T = 189 C = 219 N = color I = 5;
SU T = 190 C = 219 N = color I = 5;
SU T = 191 C = 219 N = color I = 5;
SU T = 192 C = 219 N = color I = 5;
SU T = 193 C = 219 N = color I = 5;
SU T = 194 C = 219 N = color I = 5;
SU T = 195 C = 219 N = color I = 5;
SU T = 196 C = 219 N = color I = 5;
SU T = 197 C = 219 N = color I = 5;
SU T = 198 C = 219 N = color I = 5;
SU T = 199 C = 219 N = color I = 5;
SU T = 200 C = 219 N = color I = 5;
SU T = 201 C = 219 N = color I = 5;
SU T = 202 C = 219 N = color I = 5;
SU T = 203 C = 219 N = color I = 5;
SU T = 204 C = 219 N = color I = 5;
SU T = 205 C = 219 N = color I = 5;
SU T = 206 C = 219 N = color I = 5;
SU T = 207 C = 219 N = color I = 5;
SU T = 208 C = 219 N = color I = 5;
SU T = 209 C = 219 N = color I = 5;
SU T = 210 C = 219 N = color I = 5;

SU T = 211 C = 219 N = color I = 5;
SU T = 212 C = 219 N = color I = 5;
SU T = 213 C = 219 N = color I = 5;
SU T = 214 C = 219 N = color I = 5;
SU T = 215 C = 219 N = color I = 5;
SU T = 216 C = 219 N = color I = 5;
SU T = 217 C = 219 N = color I = 5;
SU T = 218 C = 219 N = color I = 5;
SU T = 219 C = 219 N = color I = 5;
SU T = 220 C = 219 N = color I = 5;
SU T = 221 C = 219 N = color I = 5;
SU T = 222 C = 219 N = color I = 5;
SU T = 223 C = 219 N = color I = 5;
SU T = 224 C = 219 N = color I = 5;
SU T = 225 C = 219 N = color I = 5;
SU T = 226 C = 219 N = color I = 5;
SU T = 227 C = 219 N = color I = 5;
SU T = 228 C = 219 N = color I = 5;
SU T = 229 C = 219 N = color I = 5;
SU T = 230 C = 219 N = color I = 5;
SU T = 231 C = 219 N = color I = 5;
SU T = 232 C = 219 N = color I = 5;
SU T = 233 C = 219 N = color I = 5;
SU T = 234 C = 219 N = color I = 5;
SU T = 235 C = 219 N = color I = 5;
SU T = 236 C = 219 N = color I = 5;
SU T = 237 C = 219 N = color I = 5;
SU T = 238 C = 219 N = color I = 5;
SU T = 239 C = 219 N = color I = 5;
SU T = 240 C = 219 N = color I = 5;
SU T = 241 C = 219 N = color I = 5;
SU T = 242 C = 219 N = color I = 5;
SU T = 243 C = 219 N = color I = 5;
SU T = 244 C = 219 N = color I = 5;
SU T = 245 C = 219 N = color I = 5;
SU T = 246 C = 219 N = color I = 5;
SU T = 247 C = 219 N = color I = 5;
SU T = 248 C = 219 N = color I = 5;
SU T = 249 C = 219 N = color I = 5;
SU T = 250 C = 219 N = color I = 5;
SU T = 251 C = 219 N = color I = 5;
SU T = 252 C = 219 N = color I = 5;
SU T = 253 C = 219 N = color I = 5;
SU T = 254 C = 219 N = color I = 5;
SU T = 255 C = 219 N = color I = 5;
SU T = 256 C = 219 N = color I = 5;
SU T = 257 C = 219 N = color I = 5;
SU T = 258 C = 219 N = color I = 5;
SU T = 259 C = 219 N = color I = 5;
SU T = 260 C = 219 N = color I = 5;
SU T = 261 C = 219 N = color I = 5;
SU T = 262 C = 219 N = color I = 5;
SU T = 263 C = 219 N = color I = 5;
SU T = 264 C = 219 N = color I = 5;
SU T = 265 C = 219 N = color I = 5;
SU T = 266 C = 219 N = color I = 5;
SU T = 267 C = 219 N = color I = 5;
SU T = 268 C = 219 N = color I = 5;
SU T = 269 C = 219 N = color I = 5;
SU T = 270 C = 219 N = color I = 5;
SU T = 271 C = 219 N = color I = 5;
SU T = 272 C = 219 N = color I = 5;
SU T = 273 C = 219 N = color I = 5;
SU T = 274 C = 219 N = color I = 5;
SU T = 275 C = 219 N = color I = 5;
SU T = 276 C = 219 N = color I = 5;
SU T = 277 C = 219 N = color I = 5;
SU T = 278 C = 219 N = color I = 5;
SU T = 279 C = 219 N = color I = 5;
SU T = 280 C = 219 N = color I = 5;
SU T = 281 C = 219 N = color I = 5;
SU T = 282 C = 219 N = color I = 5;
SU T = 283 C = 219 N = color I = 5;
SU T = 284 C = 219 N = color I = 5;
SU T = 285 C = 219 N = color I = 5;
SU T = 286 C = 219 N = color I = 5;

SU T = 287 C = 219 N = color I = 5;
SU T = 288 C = 219 N = color I = 5;
SU T = 289 C = 219 N = color I = 5;
SU T = 290 C = 219 N = color I = 5;
SU T = 291 C = 219 N = color I = 5;
SU T = 292 C = 219 N = color I = 5;
SU T = 293 C = 219 N = color I = 5;
SU T = 294 C = 219 N = color I = 5;
SU T = 295 C = 219 N = color I = 5;
SU T = 296 C = 219 N = color I = 5;
SU T = 297 C = 219 N = color I = 5;
SU T = 298 C = 219 N = color I = 5;
SU T = 299 C = 219 N = color I = 5;
SU T = 300 C = 219 N = color I = 5;
SU T = 301 C = 219 N = color I = 5;
SU T = 302 C = 219 N = color I = 5;
SU T = 303 C = 219 N = color I = 5;
SU T = 304 C = 219 N = color I = 5;
SU T = 305 C = 219 N = color I = 5;
SU T = 306 C = 219 N = color I = 5;
SU T = 307 C = 219 N = color I = 5;
SU T = 308 C = 219 N = color I = 5;
SU T = 309 C = 219 N = color I = 5;
SU T = 310 C = 219 N = color I = 5;
SU T = 311 C = 219 N = color I = 5;
SU T = 312 C = 219 N = color I = 5;
SU T = 313 C = 219 N = color I = 5;
SU T = 314 C = 219 N = color I = 5;
SU T = 315 C = 219 N = color I = 5;
SU T = 316 C = 219 N = color I = 5;
SU T = 317 C = 219 N = color I = 5;
SU T = 318 C = 219 N = color I = 5;
SU T = 319 C = 219 N = color I = 5;
SU T = 320 C = 219 N = color I = 5;
SU T = 321 C = 219 N = color I = 5;
SU T = 322 C = 219 N = color I = 5;
SU T = 323 C = 219 N = color I = 5;
SU T = 324 C = 219 N = color I = 5;
SU T = 325 C = 219 N = color I = 5;
SU T = 326 C = 219 N = color I = 5;
SU T = 327 C = 219 N = color I = 5;
SU T = 328 C = 219 N = color I = 5;
SU T = 329 C = 219 N = color I = 5;
SU T = 330 C = 219 N = color I = 5;
SU T = 331 C = 219 N = color I = 5;
SU T = 332 C = 219 N = color I = 5;
SU T = 333 C = 219 N = color I = 5;
SU T = 334 C = 219 N = color I = 5;
SU T = 335 C = 219 N = color I = 5;
SU T = 336 C = 219 N = color I = 5;
SU T = 337 C = 219 N = color I = 5;
SU T = 338 C = 219 N = color I = 5;
SU T = 339 C = 219 N = color I = 5;
SU T = 340 C = 219 N = color I = 5;
SU T = 341 C = 219 N = color I = 5;
SU T = 342 C = 219 N = color I = 5;
SU T = 343 C = 219 N = color I = 5;
SU T = 344 C = 219 N = color I = 5;
SU T = 345 C = 219 N = color I = 5;
SU T = 346 C = 219 N = color I = 5;
SU T = 347 C = 219 N = color I = 5;
SU T = 348 C = 219 N = color I = 5;
SU T = 349 C = 219 N = color I = 5;
SU T = 350 C = 219 N = color I = 5;
SU T = 351 C = 219 N = color I = 5;
SU T = 352 C = 219 N = color I = 5;
SU T = 353 C = 219 N = color I = 5;
SU T = 354 C = 219 N = color I = 5;
SU T = 355 C = 219 N = color I = 5;
SU T = 356 C = 219 N = color I = 5;
SU T = 357 C = 219 N = color I = 5;
SU T = 358 C = 219 N = color I = 5;
SU T = 359 C = 219 N = color I = 5;
SU T = 360 C = 219 N = color I = 5;
SU T = 361 C = 219 N = color I = 5;
SU T = 362 C = 219 N = color I = 5;

SU T = 363 C = 219 N = color I = 5;
SU T = 364 C = 219 N = color I = 5;
SU T = 365 C = 219 N = color I = 5;
SU T = 366 C = 219 N = color I = 5;
SU T = 367 C = 219 N = color I = 5;
SU T = 368 C = 219 N = color I = 5;
SU T = 369 C = 219 N = color I = 5;
SU T = 370 C = 219 N = color I = 5;
SU T = 371 C = 219 N = color I = 5;
SU T = 372 C = 219 N = color I = 5;
SU T = 373 C = 219 N = color I = 5;
SU T = 374 C = 219 N = color I = 5;
SU T = 375 C = 219 N = color I = 5;
SU T = 376 C = 219 N = color I = 5;
SU T = 377 C = 219 N = color I = 5;
SU T = 378 C = 219 N = color I = 5;
SU T = 379 C = 219 N = color I = 5;
SU T = 380 C = 219 N = color I = 5;
SU T = 381 C = 219 N = color I = 5;
SU T = 382 C = 219 N = color I = 5;
SU T = 383 C = 219 N = color I = 5;
SU T = 384 C = 219 N = color I = 5;
SU T = 385 C = 219 N = color I = 5;
SU T = 386 C = 219 N = color I = 5;
SU T = 387 C = 219 N = color I = 5;
SU T = 388 C = 219 N = color I = 5;
SU T = 389 C = 219 N = color I = 5;
SU T = 390 C = 219 N = color I = 5;
SU T = 391 C = 219 N = color I = 5;
SU T = 392 C = 219 N = color I = 5;
SU T = 393 C = 219 N = color I = 5;
SU T = 394 C = 219 N = color I = 5;
SU T = 395 C = 219 N = color I = 5;
SU T = 396 C = 219 N = color I = 5;
SU T = 397 C = 219 N = color I = 5;
SU T = 398 C = 219 N = color I = 5;
SU T = 399 C = 219 N = color I = 5;
SU T = 400 C = 219 N = color I = 5;
SU T = 401 C = 219 N = color I = 5;
SU T = 402 C = 219 N = color I = 5;
SU T = 403 C = 219 N = color I = 5;
SU T = 404 C = 219 N = color I = 5;
SU T = 405 C = 219 N = color I = 5;
SU T = 406 C = 219 N = color I = 5;
SU T = 407 C = 219 N = color I = 5;
SU T = 408 C = 219 N = color I = 5;
SU T = 409 C = 219 N = color I = 5;
SU T = 410 C = 219 N = color I = 5;
SU T = 411 C = 219 N = color I = 5;
SU T = 412 C = 219 N = color I = 5;
SU T = 413 C = 219 N = color I = 5;
SU T = 414 C = 219 N = color I = 5;
SU T = 415 C = 219 N = color I = 5;
SU T = 416 C = 219 N = color I = 5;
SU T = 417 C = 219 N = color I = 5;
SU T = 418 C = 219 N = color I = 5;
SU T = 419 C = 219 N = color I = 5;
SU T = 420 C = 219 N = color I = 5;
SU T = 421 C = 219 N = color I = 5;
SU T = 422 C = 219 N = color I = 5;
SU T = 423 C = 219 N = color I = 5;
SU T = 424 C = 219 N = color I = 5;
SU T = 425 C = 219 N = color I = 5;
SU T = 426 C = 219 N = color I = 5;
SU T = 427 C = 219 N = color I = 5;
SU T = 428 C = 219 N = color I = 5;
SU T = 429 C = 219 N = color I = 5;
SU T = 430 C = 219 N = color I = 5;
SU T = 431 C = 219 N = color I = 5;
SU T = 432 C = 219 N = color I = 5;
SU T = 433 C = 219 N = color I = 5;
SU T = 434 C = 219 N = color I = 5;
SU T = 435 C = 219 N = color I = 5;
SU T = 436 C = 219 N = color I = 5;
SU T = 437 C = 219 N = color I = 5;
SU T = 438 C = 219 N = color I = 5;

```
		SU  T = 439 C = 219 N = color I = 5;
		SU  T = 440 C = 219 N = color I = 5;
		SU  T = 441 C = 219 N = color I = 5;
		SU  T = 442 C = 219 N = color I = 5;
		SU  T = 443 C = 219 N = color I = 5;
		SU  T = 444 C = 219 N = color I = 5;
		SU  T = 445 C = 219 N = color I = 5;
		SU  T = 446 C = 219 N = color I = 5;
		SU  T = 447 C = 219 N = color I = 5;
		SU  T = 448 C = 219 N = color I = 5;
		SU  T = 449 C = 219 N = color I = 5;
		SU  T = 450 C = 219 N = color I = 5;
		SU  T = 451 C = 219 N = color I = 5;
		SU  T = 452 C = 219 N = color I = 5;
		SU  T = 453 C = 219 N = color I = 5;
		SU  T = 454 C = 219 N = color I = 5;
		SU  T = 455 C = 219 N = color I = 5;
		SU  T = 456 C = 219 N = color I = 5;
		SU  T = 457 C = 219 N = color I = 5;
		SU  T = 458 C = 219 N = color I = 5;
		SU  T = 459 C = 219 N = color I = 5;
		SU  T = 460 C = 219 N = color I = 5;
		SU  T = 461 C = 219 N = color I = 5;
		SU  T = 462 C = 219 N = color I = 5;
		SU  T = 463 C = 219 N = color I = 5;
		SU  T = 464 C = 219 N = color I = 5;
		SU  T = 465 C = 219 N = color I = 5;
		SU  T = 466 C = 219 N = color I = 5;
		SU  T = 467 C = 219 N = color I = 5;
		SU  T = 468 C = 219 N = color I = 5;
		SU  T = 469 C = 219 N = color I = 5;
		SU  T = 470 C = 219 N = color I = 5;
		SU  T = 471 C = 219 N = color I = 5;
		SU  T = 472 C = 219 N = color I = 5;
		SU  T = 473 C = 219 N = color I = 5;
		SU  T = 474 C = 219 N = color I = 5;
		SU  T = 475 C = 219 N = color I = 5;
		SU  T = 476 C = 219 N = color I = 5;
		SU  T = 477 C = 219 N = color I = 5;
		SU  T = 478 C = 219 N = color I = 5;
		SU  T = 479 C = 219 N = color I = 5;
		SU  T = 480 C = 219 N = color I = 5;
		SU  T = 481 C = 219 N = color I = 5;
		SU  T = 482 C = 219 N = color I = 5;
		SU  T = 483 C = 219 N = color I = 5;
		SU  T = 484 C = 219 N = color I = 5;
		SU  T = 485 C = 219 N = color I = 5;
		SU  T = 486 C = 219 N = color I = 5;
		SU  T = 487 C = 219 N = color I = 5;
		SU  T = 488 C = 219 N = color I = 5;
		SU  T = 489 C = 219 N = color I = 5;
		SU  T = 490 C = 219 N = color I = 5;
		SU  T = 491 C = 219 N = color I = 5;
		SU  T = 492 C = 219 N = color I = 5;
		SU  T = 493 C = 219 N = color I = 5;
		SU  T = 494 C = 219 N = color I = 5;
		SU  T = 495 C = 219 N = color I = 5;
		SU  T = 496 C = 219 N = color I = 5;
		SU  T = 497 C = 219 N = color I = 5;
		SU  T = 498 C = 219 N = color I = 5;
		SU  T = 499 C = 219 N = color I = 5;
		SU  T = 500 C = 219 N = color I = 5;
		SU  T = 501 C = 219 N = color I = 5;
		SU  T = 502 C = 219 N = color I = 5;

		TEXT   TAXON = 289 CHARACTER = 223 TEXT = 0;

END;

Begin MESQUITE;
			MESQUITESCRIPTVERSION 2;
			TITLE AUTO;
			tell ProjectCoordinator;
			timeSaved 1605990840904;
			getEmployee #mesquite.minimal.ManageTaxa.ManageTaxa;
			tell It;
```

```
				setID 0 4680078835126459546;
				tell It;
					setDefaultOrder  0 1 2 3 4 5 6 7 8 9 10 11 12 13 14 15 16 17 18 19 20 21 22 23
24 25 26 27 28 29 30 31 32 33 34 35 36 37 38 39 40 41 42 43 44 45 46 47 48 49 50 51 52 53 54 55 56 57 58 59 60 61
62 63 64 65 66 67 68 69 70 71 72 73 74 75 76 77 78 79 80 81 82 83 84 85 86 87 88 89 90 91 92 93 94 95 96 97 98 99
100 101 102 103 104 105 106 107 108 109 110 111 112 113 114 115 116 117 118 119 120 121 122 123 124 125 126
127 128 129 130 131 132 133 134 135 136 137 138 139 140 141 142 143 144 145 146 147 148 149 150 151 152 153
154 155 156 157 158 159 160 161 162 163 164 165 166 167 168 169 170 171 172 173 174 175 176 177 178 179 180
181 182 183 184 185 186 187 188 189 190 191 192 193 194 195 196 197 198 199 200 201 202 203 204 205 206 207
208 209 210 211 212 213 214 215 216 217 218 219 220 221 222 223 224 225 226 227 228 229 230 231 232 233 234
235 236 237 238 239 240 241 242 243 244 245 246 247 248 249 250 251 252 253 254 255 256 257 258 259 260 261
262 263 264 265 266 267 269 270 271 272 273 274 275 276 277 278 279 280 281 282 283 284 285 286 287 288 501
268 289 290 291 292 293 294 295 296 297 298 299 300 301 302 303 304 305 306 307 308 309 310 311 312 313 314
315 316 317 318 319 320 321 322 323 324 325 326 327 328 329 330 331 332 333 334 335 336 337 338 339 340 341
342 343 344 345 346 347 348 349 350 351 352 353 354 355 356 357 358 359 360 361 362 363 364 365 366 367 368
369 370 371 372 373 374 375 376 377 378 379 380 381 382 383 384 385 386 387 388 389 390 391 392 393 394 395
396 397 398 399 400 401 402 403 404 405 406 407 408 409 410 411 412 413 414 415 416 417 418 419 420 421 422
423 424 425 426 427 428 429 430 431 432 433 434 435 436 437 438 439 440 441 442 443 444 445 446 447 448 449
450 451 452 453 454 455 456 457 458 459 460 461 462 463 464 465 466 467 468 469 470 471 472 473 474 475 476
477 478 479 480 481 482 483 484 485 486 487 488 489 490 491 492 493 494 495 496 497 498 499 500;
					attachments ;
				endTell;
			endTell;
			getEmployee #mesquite.charMatrices.ManageCharacters.ManageCharacters;
			tell It;
				setID 0 8922971771042771389;
				mqVersion 361;
				checksumv 0 3 714075737 null  getNumChars 700 numChars 700 getNumTaxa 502
numTaxa 502   short true   bits 2305843009213695231   states 1279   sumSquaresStatesOnly 1.036131643E9
sumSquares -1.9784133019052957E22 longCompressibleToShort false usingShortMatrix true   NumFiles 1
NumMatrices 1;
				mqVersion;
			endTell;
			getWindow;
			tell It;
				suppress;
				setResourcesState false false 100;
				setPopoutState 300;
				setExplanationSize 0;
				setAnnotationSize 0;
				setFontIncAnnot 0;
				setFontIncExp 0;
				setSize 1315 541;
				setLocation 31 49;
				setFont SanSerif;
				setFontSize 10;
				getToolPalette;
				tell It;
				endTell;
				desuppress;
			endTell;
			getEmployee  #mesquite.charMatrices.BasicDataWindowCoord.BasicDataWindowCoord;
			tell It;
				showDataWindow #8922971771042771389
#mesquite.charMatrices.BasicDataWindowMaker.BasicDataWindowMaker;
				tell It;
					getWindow;
					tell It;
						setExplanationSize 30;
						setAnnotationSize 20;
						setFontIncAnnot 0;
						setFontIncExp 0;
						setSize 1215 469;
						setLocation 31 49;
						setFont SanSerif;
						setFontSize 10;
						getToolPalette;
						tell It;
							setTool
mesquite.charMatrices.BasicDataWindowMaker.BasicDataWindow.ibeam;
						endTell;
						setActive;
						setTool
mesquite.charMatrices.BasicDataWindowMaker.BasicDataWindow.ibeam;
						colorCells  #mesquite.charMatrices.NoColor.NoColor;
```

```
					colorRowNames
#mesquite.charMatrices.TaxonGroupColor.TaxonGroupColor;
					colorColumnNames
#mesquite.charMatrices.CharGroupColor.CharGroupColor;
					colorText  #mesquite.charMatrices.NoColor.NoColor;
					setBackground White;
					toggleShowNames on;
					toggleShowTaxonNames on;
					toggleTight off;
					toggleThinRows off;
					toggleShowChanges on;
					toggleSeparateLines off;
					toggleShowStates on;
					toggleReduceCellBorders off;
					toggleAutoWCharNames on;
					toggleAutoTaxonNames off;
					toggleShowDefaultCharNames off;
					toggleConstrainCW on;
					toggleBirdsEye off;
					toggleShowPaleGrid off;
					toggleShowPaleCellColors off;
					toggleShowPaleExcluded off;
					togglePaleInapplicable on;
					togglePaleMissing off;
					toggleShowBoldCellText off;
					toggleAllowAutosize on;
					toggleColorsPanel off;
					toggleDiagonal on;
					setDiagonalHeight 80;
					toggleLinkedScrolling on;
					toggleScrollLinkedTables off;
				endTell;
				showWindow;
				getWindow;
				tell It;
					forceAutosize;
				endTell;
				getEmployee #mesquite.charMatrices.AlterData.AlterData;
				tell It;
					toggleBySubmenus off;
				endTell;
				getEmployee #mesquite.charMatrices.ColorByState.ColorByState;
				tell It;
					setStateLimit 9;
					toggleUniformMaximum on;
				endTell;
				getEmployee #mesquite.charMatrices.ColorCells.ColorCells;
				tell It;
					setColor Red;
					removeColor off;
				endTell;
				getEmployee #mesquite.categ.StateNamesStrip.StateNamesStrip;
				tell It;
					showStrip off;
				endTell;
				getEmployee #mesquite.charMatrices.AnnotPanel.AnnotPanel;
				tell It;
					togglePanel off;
				endTell;
				getEmployee #mesquite.charMatrices.CharReferenceStrip.CharReferenceStrip;
				tell It;
					showStrip off;
				endTell;
				getEmployee #mesquite.charMatrices.QuickKeySelector.QuickKeySelector;
				tell It;
					autotabOff;
				endTell;
				getEmployee #mesquite.charMatrices.SelSummaryStrip.SelSummaryStrip;
				tell It;
					showStrip off;
				endTell;
				getEmployee
#mesquite.categ.SmallStateNamesEditor.SmallStateNamesEditor;
				tell It;
					panelOpen true;
				endTell;
```

```
                endTell;
            endTell;
            endTell;
end;
```
